# Mapping global prevalence of depression among postpartum women

**DOI:** 10.1038/s41398-021-01663-6

**Published:** 2021-10-20

**Authors:** Ziyi Wang, Jiaye Liu, Huan Shuai, Zhongxiang Cai, Xia Fu, Yang Liu, Xiong Xiao, Wenhao Zhang, Elise Krabbendam, Shuo Liu, Zhongchun Liu, Zhihui Li, Bing Xiang Yang

**Affiliations:** 1 School of Nursing, Wuhan University, Wuhan, Hubei China; 2 Department of Thyroid and Parathyroid Surgery, West China Hospital, Sichuan University, Chengdu, Sichuan China; 3 Department of Orthopedics, Chengkou People’s Hospital, Chongqing, China; 4 Department of Nursing, Renmin Hospital of Wuhan University, Wuhan, Hubei China; 5 Department of outpatients, West China Hospital, Sichuan University, Chengdu, Sichuan China; 6 Department of Obstetrics and Gynecology, Second Affiliated Hospital, Chongqing Medical University, Chongqing, China; 7 Chengdu Women’s and Children’s Central Hospital, School of Medicine, University of Electronic Science and Technology of China, Chengdu, China; 8 Department of Obstetrics and Gynecology, Key Laboratory of Birth Defects and Related Diseases of Women and Children, Ministry of Education, West China Second University Hospital, Sichuan University, Chengdu, China; 9 Department of Medical Library, Erasmus MC-University Medical Center, Rotterdam, The Netherlands; 10 Department of Psychiatry, Renmin Hospital of Wuhan University, Wuhan, Hubei China

**Keywords:** Depression, Human behaviour

## Abstract

Postpartum depression (PPD) is the most common psychological condition following childbirth, and may have a detrimental effect on the social and cognitive health of spouses, infants, and children. The aim of this study was to complete a comprehensive overview of the current literature on the global epidemiology of PPD. A total of 565 studies from 80 different countries or regions were included in the final analysis. Postpartum depression was found in 17.22% (95% CI 16.00–18.51) of the world’s population. Meta-regression analysis showed that study size, country or region development, and country or region income were the causes of heterogeneity. Multivariable meta-regression analysis found that study size and country or area development were the most important predictors. Varied prevalence rates were noted in geographic regions with the highest rate found in Southern Africa (39.96%). Of interested was a significantly lower rate of PPD in developed countries or high-income countries or areas. Furthermore, the findings showed that there was a substantial difference in rates of PPD when marital status, educational level, social support, spouse care, violence, gestational age, breast feeding, child mortality, pregnancy plan, financial difficulties, partnership, life stress, smoking, alcohol intake, and living conditions were considered in the pooled estimates. Our results indicated that one out of every five women experiences PPD which is linked to income and geographic development. It is triggered by a variety of causes that necessitate the attention and committed intervention of primary care providers, clinicians, health authorities, and the general population.

## Introduction

Postpartum depression (PPD), the onset of depressive episodes after childbirth, develops at a critical moment in a woman’s life and can continue for long periods [1, 2]. The likelihood of depressive episodes can be twice as high as during other periods of a woman’s life [3], and they often go undetected and untreated [4], wreaking havoc on partners as well as the emotional and cognitive growth of infants and adolescents [5, 6]. Desperation, sadness, nausea, changes in sleep and eating habits, decreased libido, crying spells, anxiety, irritability, feelings of isolation, mental liability, thoughts of hurting oneself and/or the infant, and even thoughts of suicide are common signs of this form of depression [7]. Postpartum depression can start at any time within the first year after delivery and continue for several years.

In Western countries, the prevalence of PPD varies from 10 to 15% during the first year after birth [8]. According to a systematic review of 47 studies from 18 low and lower-middle income countries, the prevalence is 18.6% (95% CI 18.0–19.2). Moreover, another review involving 143 studies from 40 countries found a broader variety of PPD prevalence rates, ranging from 0.5% to around 60% [9]. Cultural variations, diverse reporting practices, different viewpoints on mental health issues and stigma, socioeconomic class, poverty, poor social services, deficient nutrition, elevated stress, and biological factors can all be related to this broader continuum.

Despite the increasing number of longitudinal studies on postpartum depression around the world, there is a paucity of rigorous systemic data that explores not only the general burden of PPD, but also risk factors associated with it. The current understanding of the epidemiology of PPD is based mostly on a few geographic surveys and very little national evidence. Therefore, the present study aims to close this void by presenting an updated global estimate of postpartum depression epidemiology, synthesizing critical risk factors, and providing evidence-based data for maternal mental health treatment prioritization.

## Methods

### Search strategy and selection criteria

A systematic review and meta-analysis were done in accordance with PRISMA guidelines. Articles from inception to July 2021 were reviewed using the following databases: Medline, Embase, Web of Science, Cochrane, PsycInfo and Google Scholar. Searches were tailored database functionality, but generally used the following terms: “postnatal depression”, “postpartum depression”, “depression” and “depressive symptoms”. For relevant citations, reference lists of retrieved papers, as well as review articles found during the initial search, were screened. In order to uncover additional reports, forward citation checks were performed. Full details of the search strategy are provided in the Supplementary method.

Pre-defined decision rules were used to screen studies. Two reviewers independently screened titles and abstracts, with 10% of studies randomly reviewed by another investigator. Two investigators reviewed the complete texts of theoretically qualifying papers, with any inconsistencies settled through agreement or by another reviewer; consensus was found in all cases and agreement was reached.

Datasets from studies that met the following criteria were deemed eligible: (1) studies identified postpartum depressive disorder or depressive episode; (2) participants were women who completed assessments at least one week after giving birth in order to avoid baby blues being confused with PPD; (3) prevalence or incidence of depression was determined using standardized validated instruments, self-reported questionnaires, or clinically structured interviews; (4) participants were not recruited if they were receiving psychiatric assessment or care, or if they were identified as having possible depression because screening seeks to identify women with otherwise unrecognized major depression; (5) sufficient information was available for authors to calculate the aggregated prevalence of depression in the selected group of participants; and, (6) study undertaken from January, 2000 to July 2021.

### Data analysis

Data extracted included the first author of the study, country or region, geographic region, time of publication, study period, income of country or region as assess by the World Bank, the level of country development, study category, study source, diagnostic technique, sample size, study quality and prevalence of disease (Supplementary Table 1). In addition, a comparison was made of the prevalence of postpartum depression stratified by marital status (married/cohabiting or single/divorced/widowed), employment (unemployed or paid employment), sex of infant (male or female), parity (primiparous or multiparous), educational level (less than 12 years or more than 12 years), social support from friends/family/parents (YES/NO), support from partner (YES/NO), violence (YES/NO), maternal age (adolescent or adult), residence (urban or rural/suburb), religion (YES/NO), mode of delivery (spontaneous or instrumental/cesarean delivery), ethnicity (indigenous or nonindigenous/immigrant), location of delivery (home or health facility), gestational age (term or pre-/post-term), breast feeding (YES/NO), infant death (YES/NO), sleep (satisfactory or unsatisfactory), pregnancy planned (YES/NO), gender preference (YES/NO), financial problems (YES/NO), partnership (intimate/satisfactory or unsatisfied), life stress (YES/NO), smoker (YES/NO), alcohol use (YES/NO), family type (nuclear or extended), living condition (satisfactory/good or unsatisfactory) and number of children (1 or less/2 or more, Supplementary Table 2).

Socio-demographic characteristics of participants were identified and compared with the prevalence of postpartum depression to determine the pool estimates of risk factors for PPD. Data were cross-checked for accuracy against the original source. Researchers reviewed and extracted data from included studies by using a data extraction form specifically designed for the current study. When duplicate data were identified, the duplicate with the smallest sample size or shortest duration of follow-up was excluded.

The Joanna Briggs Institute-Checklist for Prevalence Studies was used to assess the methodological consistency of the qualifying studies (Supplementary Table 1). Based on quality score, studies were not omitted in order to improve clarity and to ensure that available data in this field was reported. After checking for consistency, the “Meta” and “Metafor” modules in the R-4.0.0 statistical software package were used for meta-analysis. A 95% confidence interval (95% CI) was estimated using the Wilson score method, and pooled prevalence was calculated with the DerSimonian-Laird random effects model with Logit transformation. Heterogeneity across included studies was assessed using Cochran Q and I^2^ statistics. Estimates with a p value lower than 0.05 for the Q-statistic and I² of 50% or greater were considered to have moderate heterogeneity. A random-effects approach was used to pool the prevalence of postpartum depression as global evidence was assumed to be heterogeneous. A series of leave-one out diagnostic tests was performed by sensitivity analysis and findings were further checked using a build-in feature in Metafor. Using a Mixed-Effects Model, meta-regression was then conducted after eliminating the outliers. Covariates considered were geographic regions, development of countries or region, income of countries or regions, diagnostic techniques, publication time, study size and study quality score. Consequently, multivariable meta-regression (multi-model inference) was performed using a “dmetar” package to examine which possible predictor combinations provide the best fit, and which predictors are the most important ones overall. To assess the potential confounding effect of heterogeneity, subgroup analyses were performed. The P value was used to compare the difference between the groups. A *P* value <0.05 was considered as significant difference. The study protocol was registered at PROSPERO (https://www.crd.york.ac.uk/prospero) as CRD42020211478.

## Results

### Literature search results and study characteristics

**Fig. 1:**
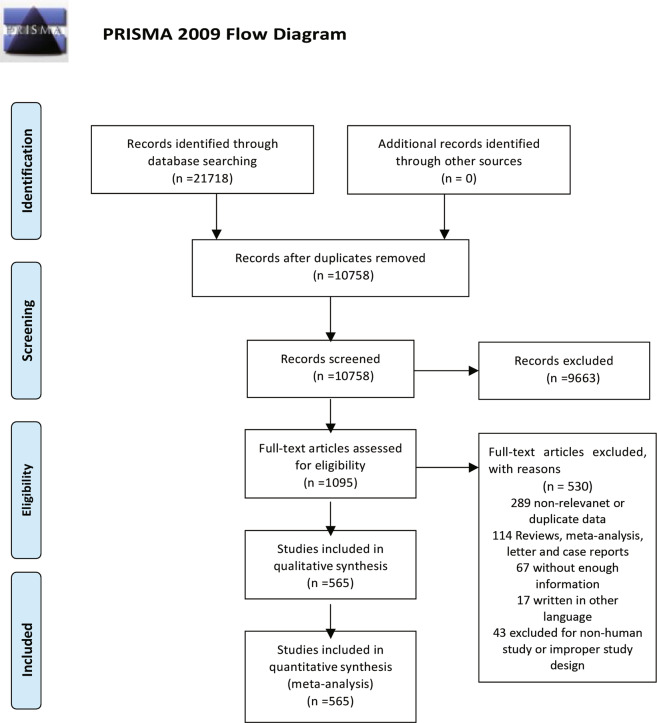
Study selection. (Description of the searching methodology).

The search identified 21,718 records, of which 10,758 records were retained after removing duplicates. Titles and abstracts were screened, resulting in the exclusion of 9,663 ineligible records. Full texts of the remaining 1,095 records were assessed for eligibility, of which 530 were excluded. Overall, 565 eligible studies involving 1,236,365 women were included in the final analysis (Fig. 1, Supplementary Table 1). The quality assessment scores of included studies are displayed in Supplementary Table 1. The majority of included studies had a cross-sectional design. The mean or median age of participants ranged from 17.76 to 37.70 years.

**Fig. 2:**
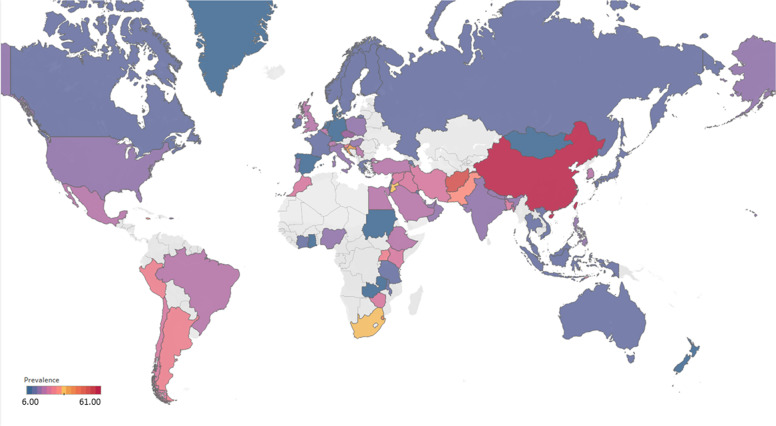
Global prevalence of postpartum depression. (Unbalanced prevalence of postpartum depression was observed between different continents, countries and regions).

Participants included 172,342 women from 80 countries, having a diagnosis of postpartum depression. There was a high degree of heterogeneity among documented results with an overall prevalence rate of 17.22% (95% CI 16.00–18.51, *I*^2^ = 99.70%, Table 1, Fig. 2).

**Table 1:** Subgroup analysis for PPD prevalence among women.

Subgroup	Studies	Prevalence	PPD	Participants	*P* value	*I*^2^
Country					**<0.01**	
South Africa	6	38.79 (25.71–53.72)	1076	3144		98.00%
Malawi	3	13.57 (8.94–20.07)	116	830		77.50%
Zambia	1	9.71 (6.74–13.79)	27	278		
Ghana	4	8.00 (1.90–28.13)	606	4294		99.30%
Cote d’Ivoire	1	14.13 (10.90–18.11)	51	361		
Egypt	6	22.99 (12.99–37.38)	472	2218		97.50%
Sudan	2	7.56 (5.47–10.34)	35	467		0.00%
Zimbabwe	2	27.22 (16.52–41.41)	281	1137		94.80%
Ethiopia	13	22.79 (18.07–28.33)	2666	11534		97.50%
Tanzania	3	12.30 (11.22–13.49)	407	3310		98.00%
Nigeria	7	17.93 (10.19–29.59)	1008	10659		0.00%
Israel	12	12.90 (9.38–17.50)	1188	9158		96.60%
Japan	27	13.30 (12.25–14.41)	25035	199089		96.20%
India	14	18.81 (13.59–25.44)	1817	10554		97.60%
Turkey	26	21.87 (18.12–26.15)	3038	12370		96.30%
Malaysia	6	12.64 (6.97–21.86)	1021	10402		98.80%
China	42	17.98 (15.32–20.99)	5946	31517		97.10%
Taiwan	17	21.65 (17.56–26.38)	661	3141		88.00%
Vietnam	8	13.77 (8.54–21.47)	1009	7314		98.20%
Pakistan	3	35.45 (18.47–57.10)	440	1326		97.60%
United Arab Emirates	1	18.31 (10.94–29.03)	13	71		—
Nepal	11	16.41 (12.07–21.91)	538	2443		92.80%
Thailand	8	12.52 (8.02–19.01)	514	4547		96.10%
Singapore	6	14.24 (10.10–19.71)	402	2602		98.40%
Bangladesh	10	26.65 (20.00–34.56)	1329	4423		98.80%
Qatar	3	18.00 (16.90–19.16)	795	4417		91.70%
USA	66	18.56 (16.91–20.34)	34380	242105		96.70%
Canada	25	13.89 (11.43–16.79)	13421	118968		98.60%
Australia	23	11.22 (9.23–13.56)	6928	82680		0.00%
Argentina	2	29.88 (19.30–43.14)	165	625		83.00%
France	8	14.63 (10.01–20.89)	493	3203		93.80%
Oman	2	16.38 (9.15–27.58)	160	874		90.60%
Brazil	31	20.51 (18.53–22.65)	19960	88955		98.00%
Poland	5	17.91 (9.19–31.97)	365	2013		97.60%
Spain	5	9.09 (6.97–11.78)	360	3704		84.10%
UK	13	21.50 (17.63–25.94)	25786	219769		98.40%
Sweden	14	12.18 (9.41–15.63)	2057	18189		97.20%
Kuwait	1	11.72 (10.11–13.55)	158	1348		—
Norway	12	11.24 (8.31–15.03)	1711	12557		97.00%
Iran	18	24.41 (17.18–33.43)	5834	15753		99.20%
Kenya	3	25.20 (11.50–46.63)	70	391		90.50%
Jordan	4	39.78 (21.43–61.54)	539	1333		98.20%
Uganda	1	32.67 (27.60–38.18)	98	300		—
Indonesia	2	11.76 (3.73–31.43)	51	440		93.90%
Eswatini	1	47.37 (38.39–56.52)	54	114		—
Syria	1	28.24 (25.66–30.96)	312	1105		—
Saudi Arabia	7	20.08 (14.17–27.65)	457	2305		93.40%
New Zealand	5	10.58 (5.62–19.01)	681	8057		98.30%
Multi	3	20.21 (10.16–36.19)	233	1474		96.60%
Morocco	1	27.00 (19.22–36.51)	27	100		—
Portugal	4	18.28 (12.57–25.81)	115	663		77.90%
Italy	14	16.79 (11.63–23.64)	1283	10800		97.70%
Netherlands	7	10.69 (5.76–18.98)	1222	13069		99.10%
Ireland	3	11.14 (10.13–12.25)	1275	11694		19.10%
Iraq	1	28.40 (25.69–31.28)	284	1000		—
Greece	4	12.26 (8.02–18.30)	155	1259		83.40%
Hungary	1	16.54 (12.54–21.50)	44	266		—
Lebanon	1	12.75 (8.28–19.13)	19	149		—
Korea	4	22.50 (12.01–38.17)	246	1263		96.10%
Finland	3	14.62 (9.83–21.20)	320	2513		92.20%
Denmark	2	6.48 (5.70–7.36)	219	3381		0.00%
Czech Republic	2	15.82 (7.08–31.66)	59	408		90.30%
Belgium	1	23.94 (15.44–35.19)	17	71		—
Philippines	1	16.36 (11.47–22.81)	27	165		—
Mexico	1	20.00 (15.13–25.96)	42	210		—
Hong Kong	4	16.96 (13.77–20.70)	321	1860		71.90%
Greenland	1	8.62 (5.26–13.81)	15	174		—
Germany	3	9.76 (5.26–17.40)	161	1760		92.90%
Bahrain	1	37.13 (31.21–43.46)	88	237		—
Serbia	3	23.66 (10.41–45.27)	134	573		95.60%
Peru	1	29.97 (28.00–32.02)	597	1992		—
Armenia	2	14.08 (11.48–17.14)	82	583		0.00%
Mongolia	1	9.10 (7.50–11.00)	95	1044		—
Russia	2	13.54 (1.71–58.45)	205	820		97.80%
Switzerland	1	21.93 (17.39–27.27)	59	269		—
Croatia	1	45.03 (37.74–52.54)	77	171		—
Slovenia	1	31.82 (19.84–46.81)	14	44		—
Afghanistan	1	60.93 (54.25–67.22)	131	215		—
Chile	3	28.27 (14.87–47.08)	177	629		95.10%
Timor-Leste	1	25.29 (19.33–32.36)	43	170		—
Jamaica	1	34.25 (24.31–45.79)	25	73		—
Continent					**<0.01**	
Southern Africa	7	39.96 (27.81–53.48)	1130	3258		97.70%
Eastern Africa	26	20.21 (16.86–24.04)	3665	17780		97.00%
Western Africa	12	13.62 (8.27–21.62)	1665	25314		99.00%
Northern Africa	9	18.75 (11.40–29.26)	534	2785		96.90%
Western Asia	62	19.83 (17.33–22.58)	7133	34950		97.20%
Eastern Asia	96	17.39 (16.09–18.77)	32429	238273		97.70%
Southern Asia	56	22.32 (18.48–26.70)	9964	35227		98.80%
South-eastern Asia	33	13.53 (11.00–16.52)	3208	26677		97.30%
Northern America	92	17.01 (15.68–18.44)	47816	361247		98.70%
Oceania	28	11.11 (9.27–13.25)	7609	90737		98.30%
South America	37	21.71 (19.78–23.76)	20899	92201		97.80%
Western Europe	20	12.91 (9.44–17.40)	1952	18372		97.90%
Eastern Europe	10	16.62 (10.95–24.43)	673	3507		96.50%
Northern Europe	47	13.78 (12.47–15.21)	31368	268103		97.20%
Southern Europe	31	16.34 (12.90–20.48)	1997	16177		96.60%
Central America	1	20.00 (15.13–25.96)	42	210		96.70%
Caribbean	1	34.25 (24.31–45.79)	25	73		—
Multiple	3	20.21 (10.16–36.19)	233	1474		—
Development					**<0.01**	
Developing	295	19.99 (18.76–21.27)	54145	266334		98.30%
Developed	276	14.85 (14.22–15.51	118197	970031		98.20%
Country or regional income					**<0.01**	
High	314	15.54 (14.90–16.20)	121333	985634		98.20%
Upper-middle	178	19.68 (18.26–21.19)	41217	186768		98.40%
Lower-middle	56	20.14 (16.39–24.50)	5769	39108		98.50%
Low	23	20.02 (15.32–25.73)	4023	24855		98.70%
Publication date					**0.58**	
Before 2010	145	17.94 (15.79–20.30)	19417	120264		98.80%
After 2010	426	17.28 (16.54-18.05)	152925	1116101		98.80%
Study size					**<0.01**	
<1000	421	19.44 (18.40–20.51)	27433	137934		95.70%
>1000	150	12.97 (12.03–13.96)	144909	1098431		99.50%
Diagnostic technique					**<0.01**	
DSM-IV	7	12.94 (9.14–18.00)	378	3086		91.50%
SRQ	12	25.90 (21.16–31.27)	3681	12948		97.70%
PHQ-9	19	18.59 (12.21–27.44)	2475	23846		99.10%
EPDS	464	16.86 (16.04–17.72)	110611	775287		98.70%
CES-D	13	25.06 (19.55–31.50)	2763	19702		97.80%
BDI	10	29.70 (23.07–37.31)	553	1767		89.30%
DASS	4	18.47 (16.43–20.70)	1234	6863		72.40%
SCID	5	10.11 (3.75–24.48)	1712	6360		98.90%
PDSS	5	37.23 (21.47–56.27)	471	1522		97.50%
SDS	4	28.17 (21.30–36.24)	1231	4248		95.90%
PHQ-2	5	18.47 (14.42–23.34)	14461	105902		99.30%
Others	23	14.94 (13.06–17.03)				96.10%
Study quality					**0.41**	
>8	542	17.52 (16.80–18.27)	163375	1184434		98.80%
<8	29	16.03 (12.98–19.63)	8967	51931		98.90%
Study period					**0.56**	
4 weeks–3 months	342	15.35 (14.10–16.70)	55359	918858		98.20%
3 months–6 months	89	15.92 (14.03–18.02)	26031	277293		99.10%
6 months–12 months	67	17.89 (13.32–23.59)	55092	1560464		99.90%
Longer than 12 months	15	17.95 (13.80–23.01)	1781	11374		96.60%

**Fig. 3:**
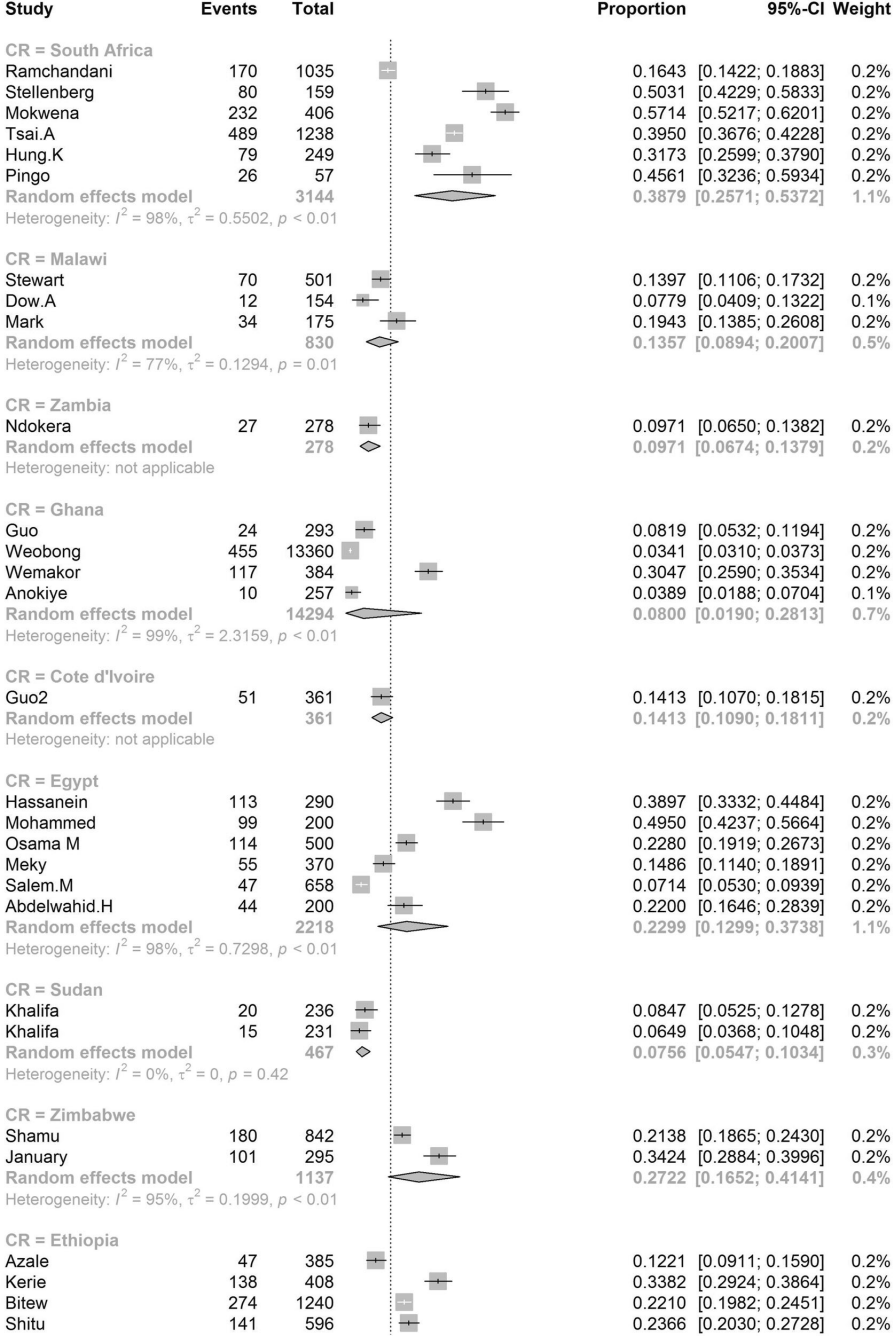

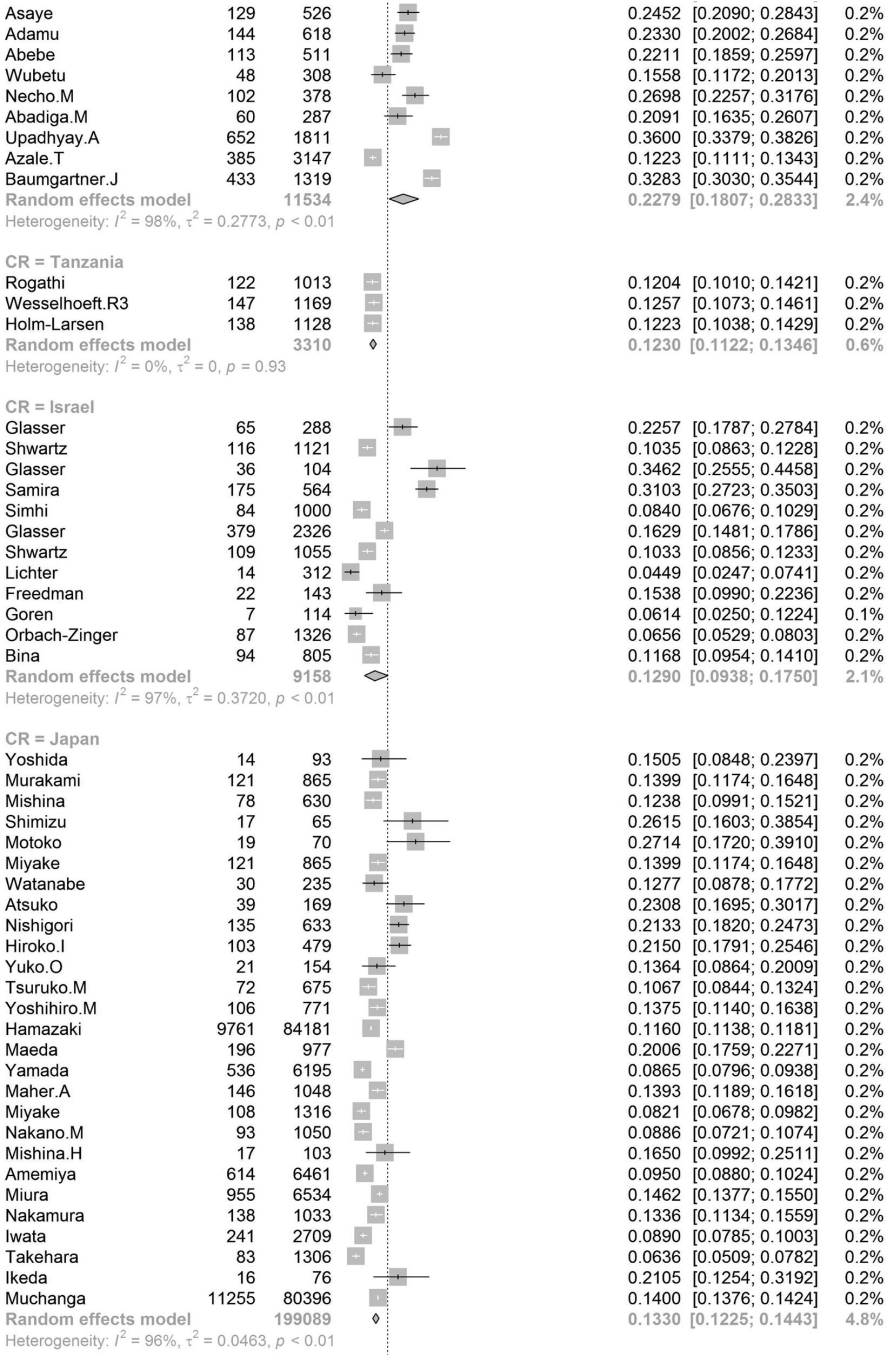

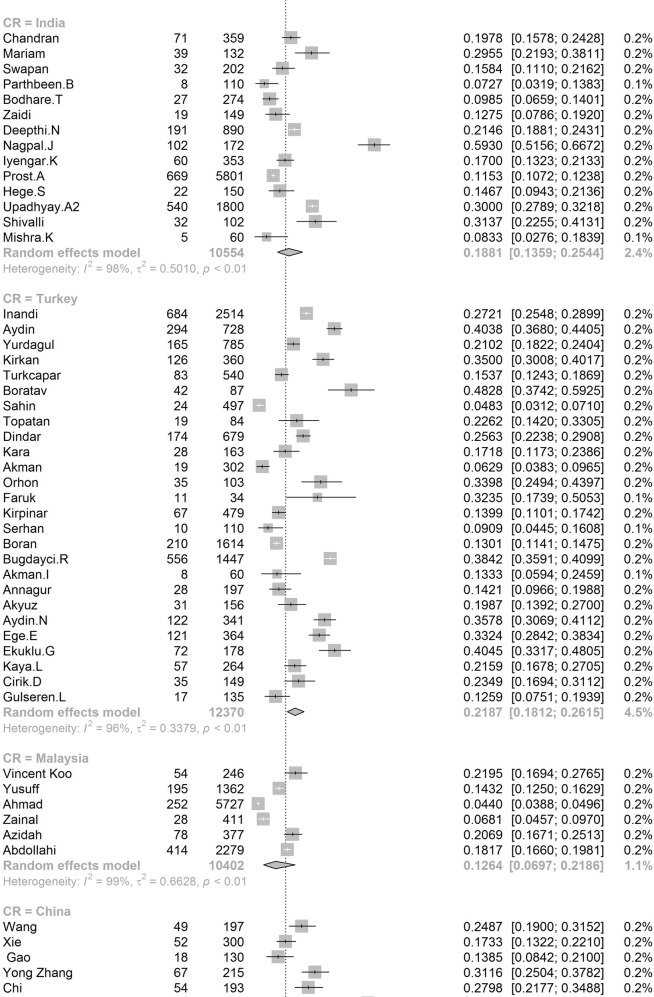

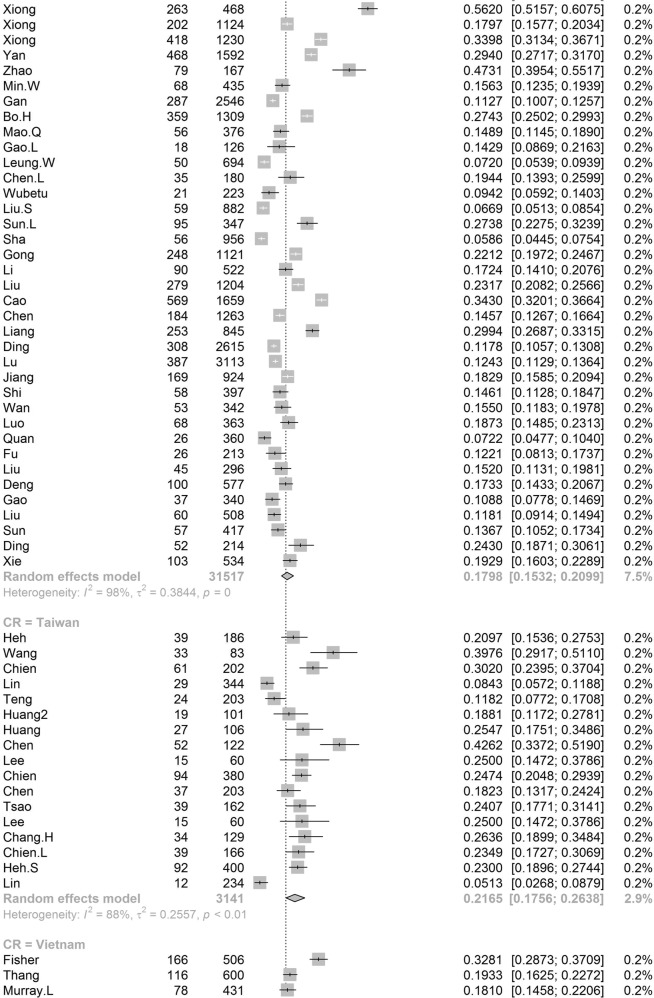

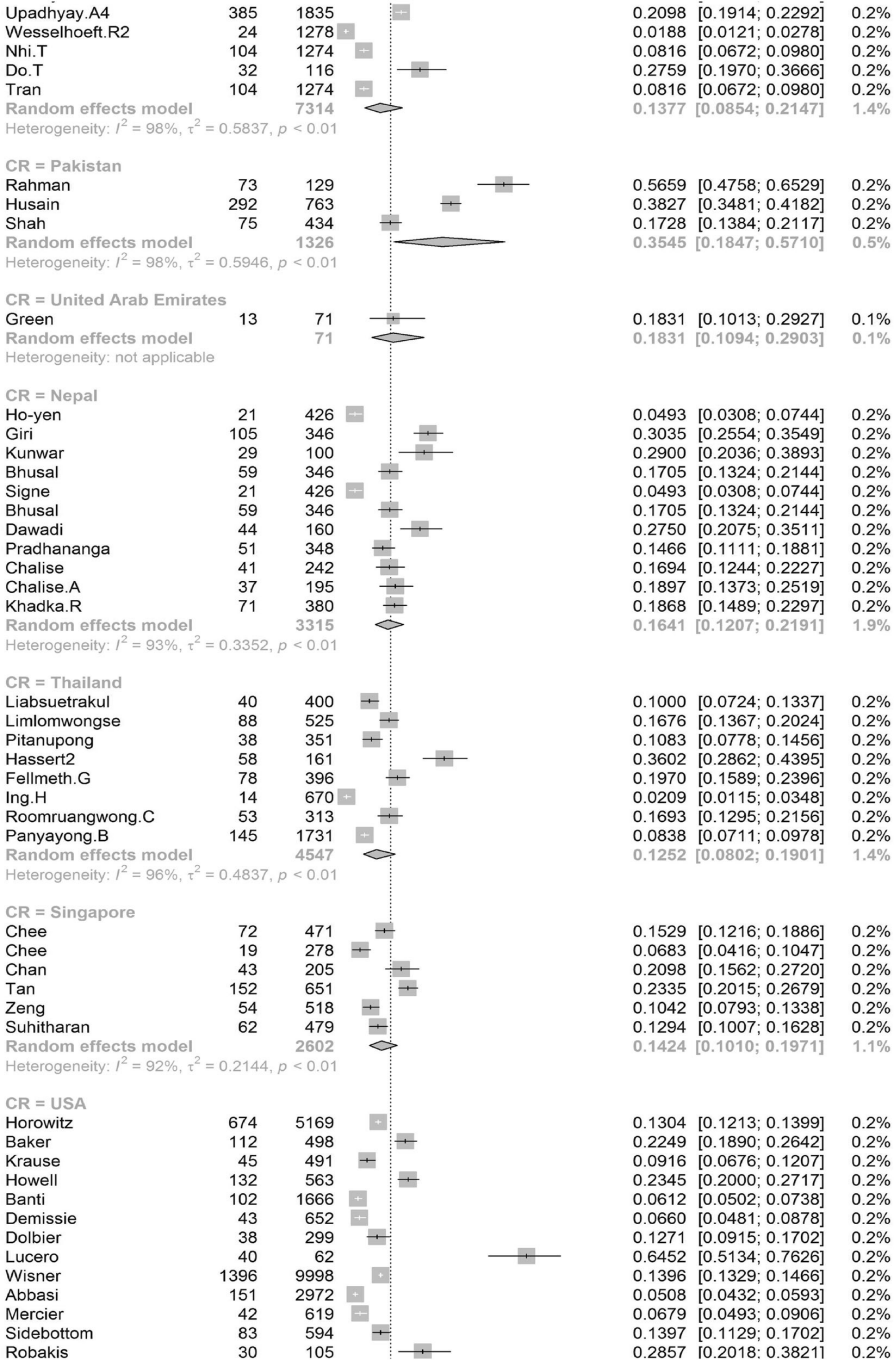

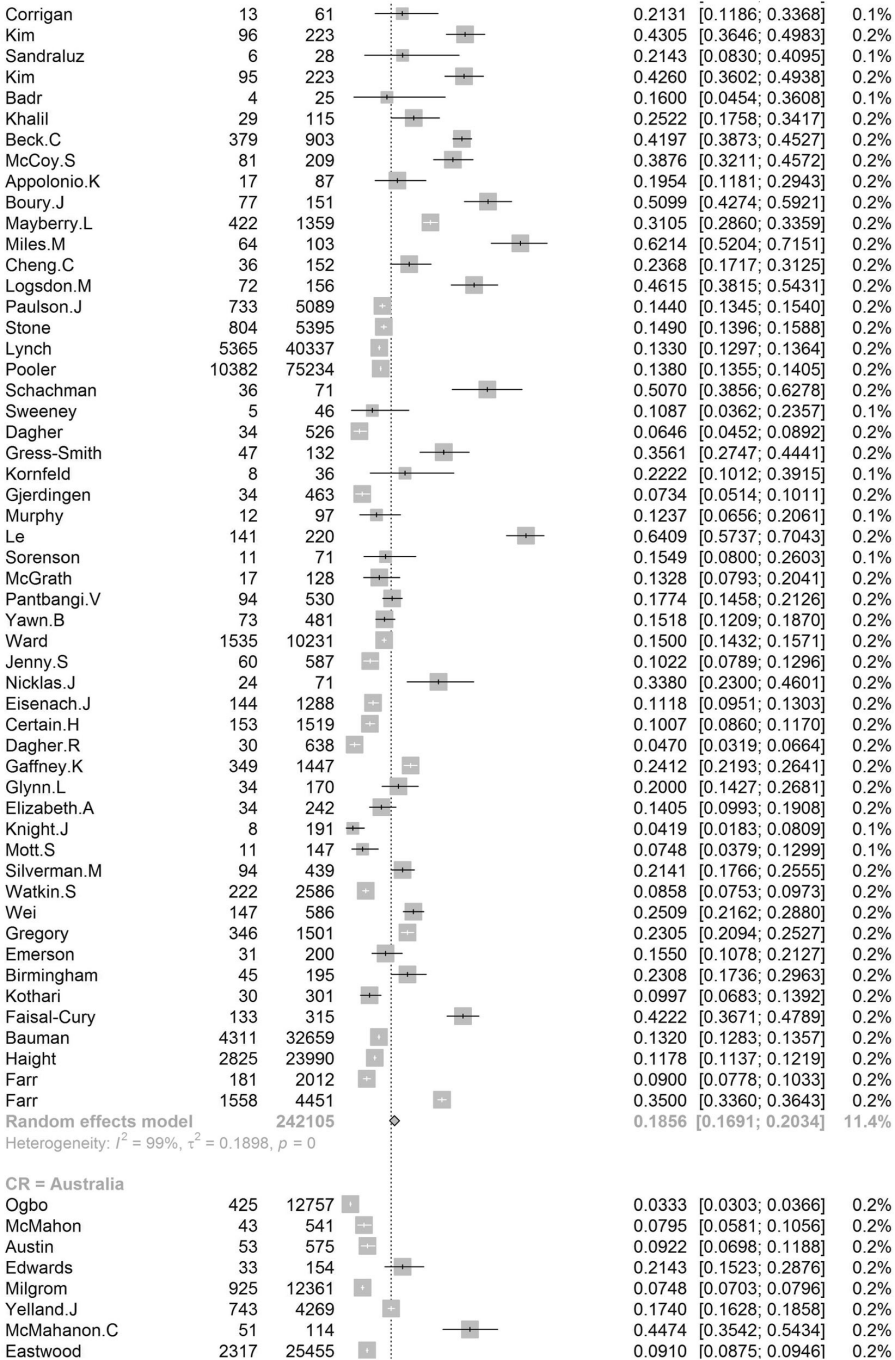

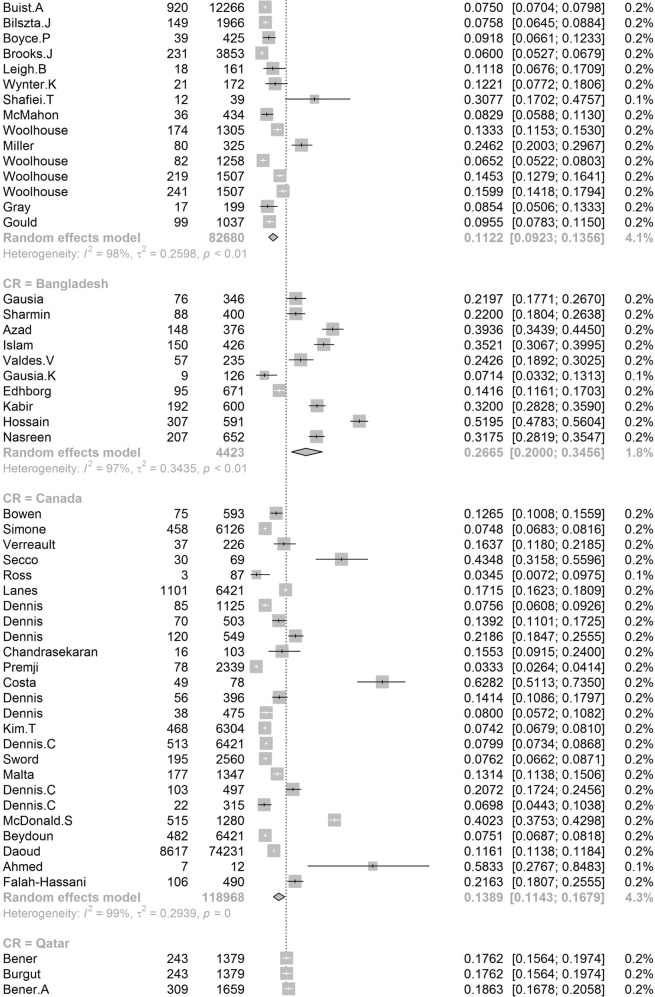

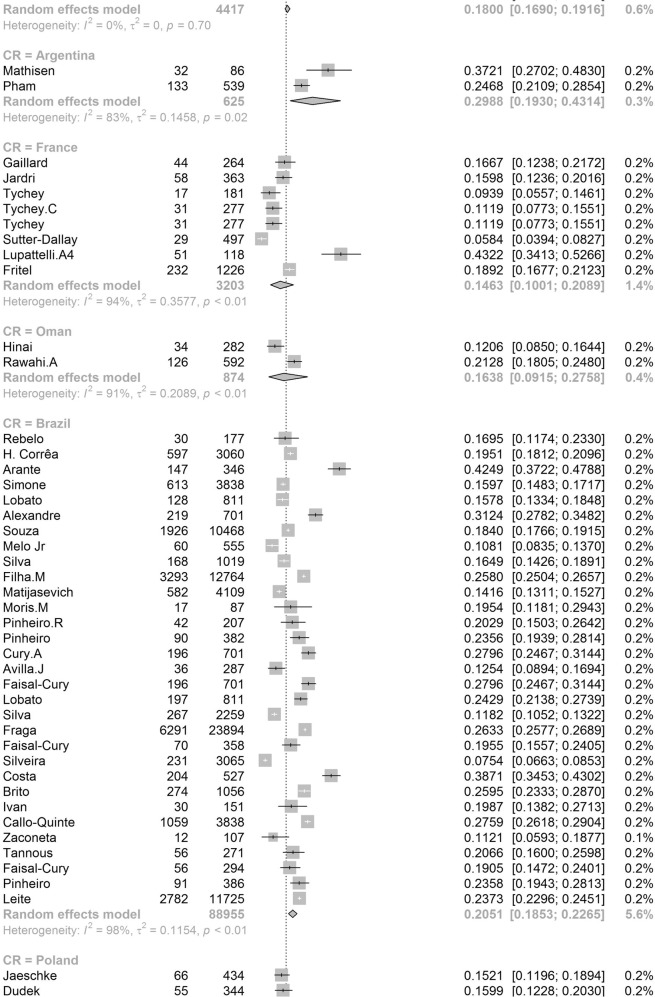

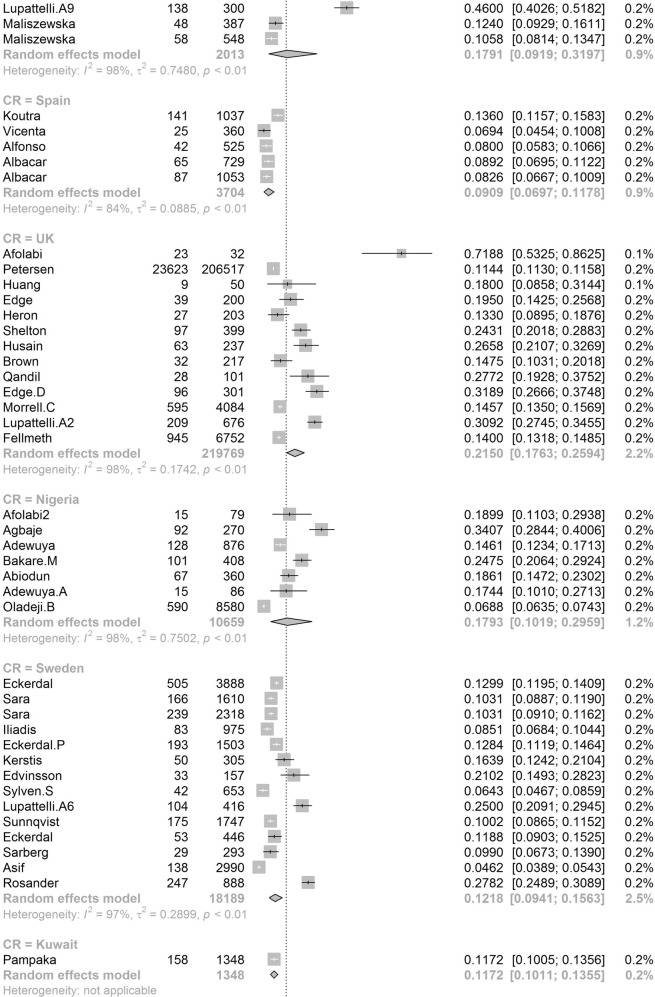

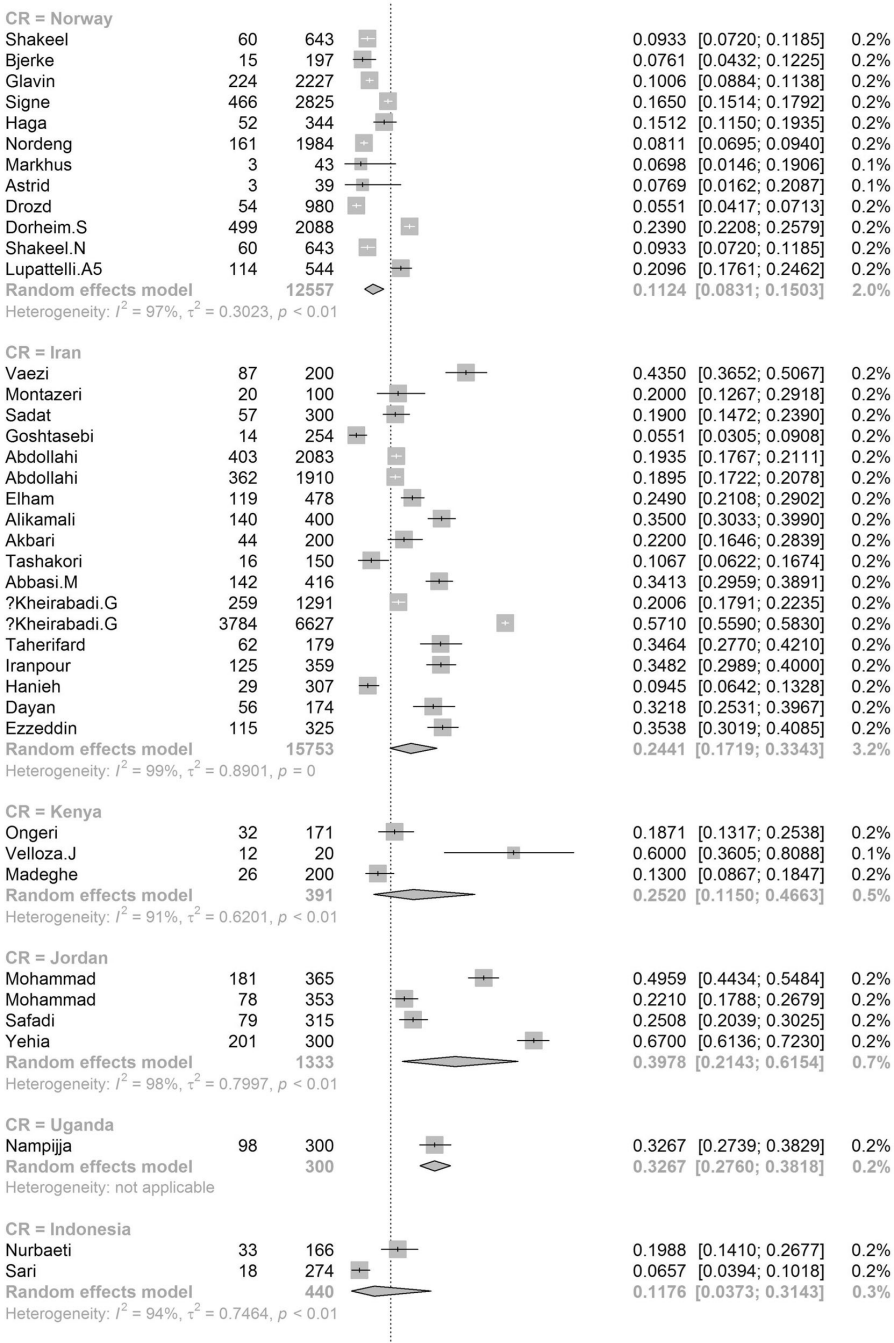

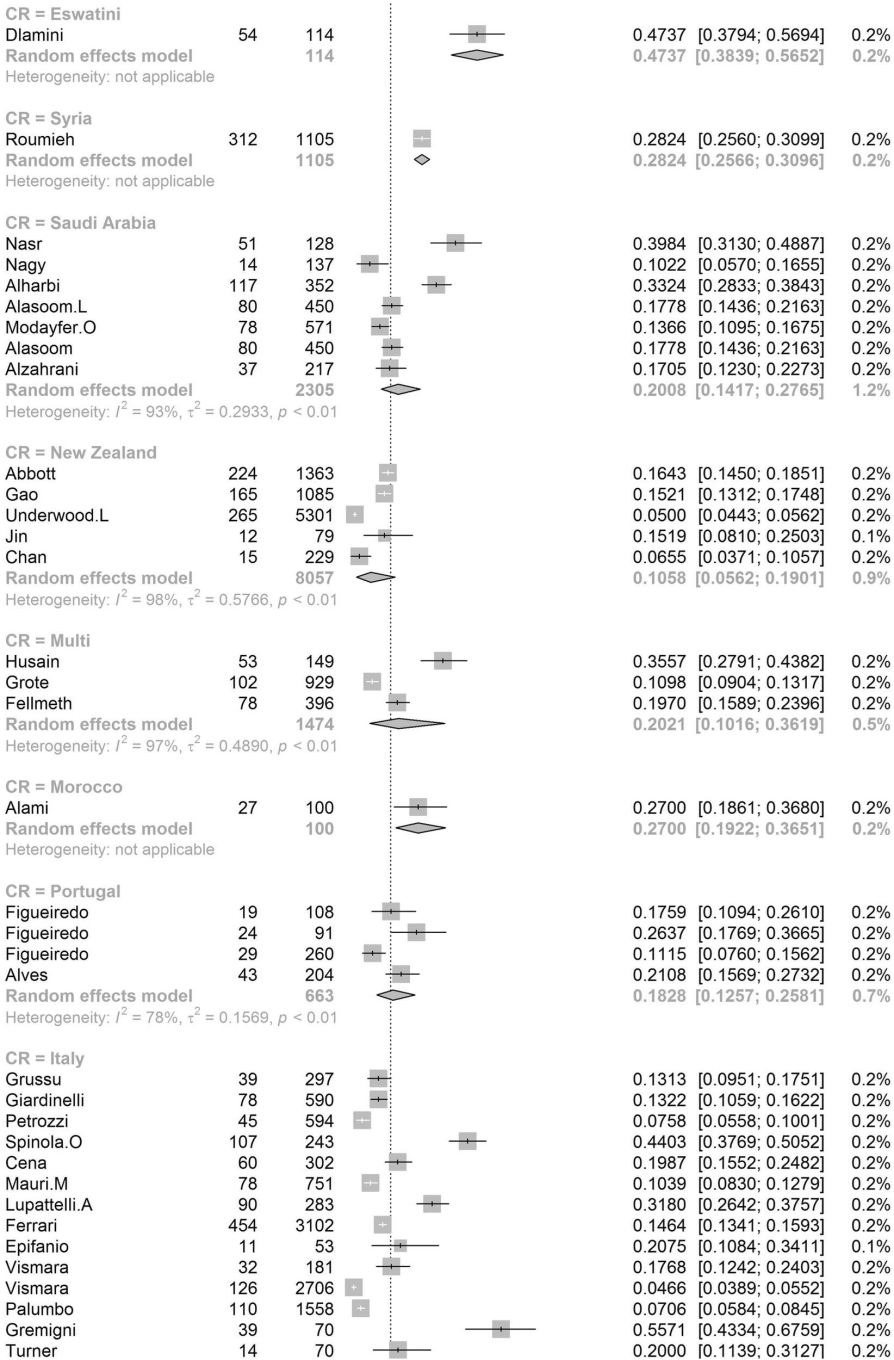

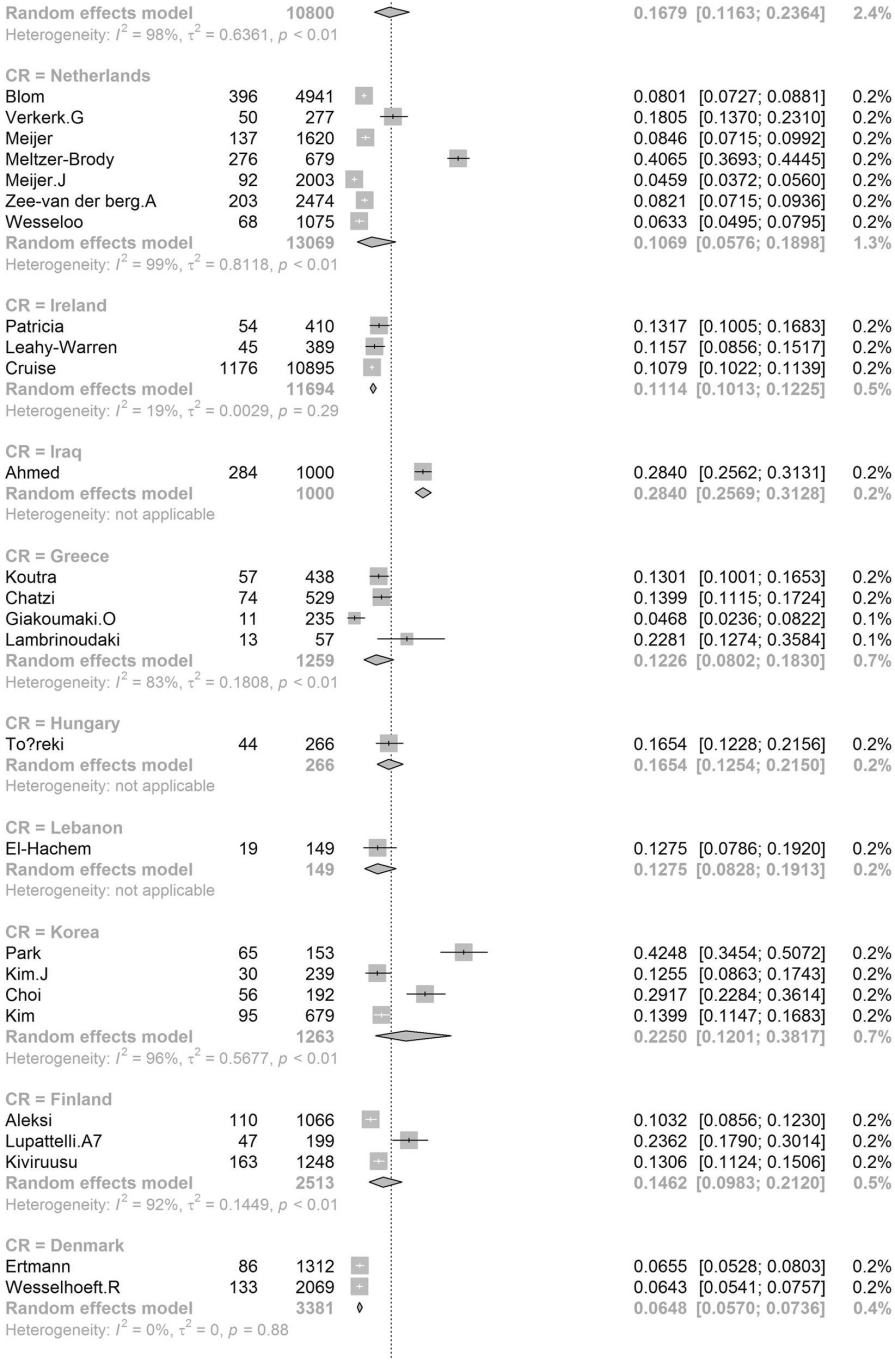

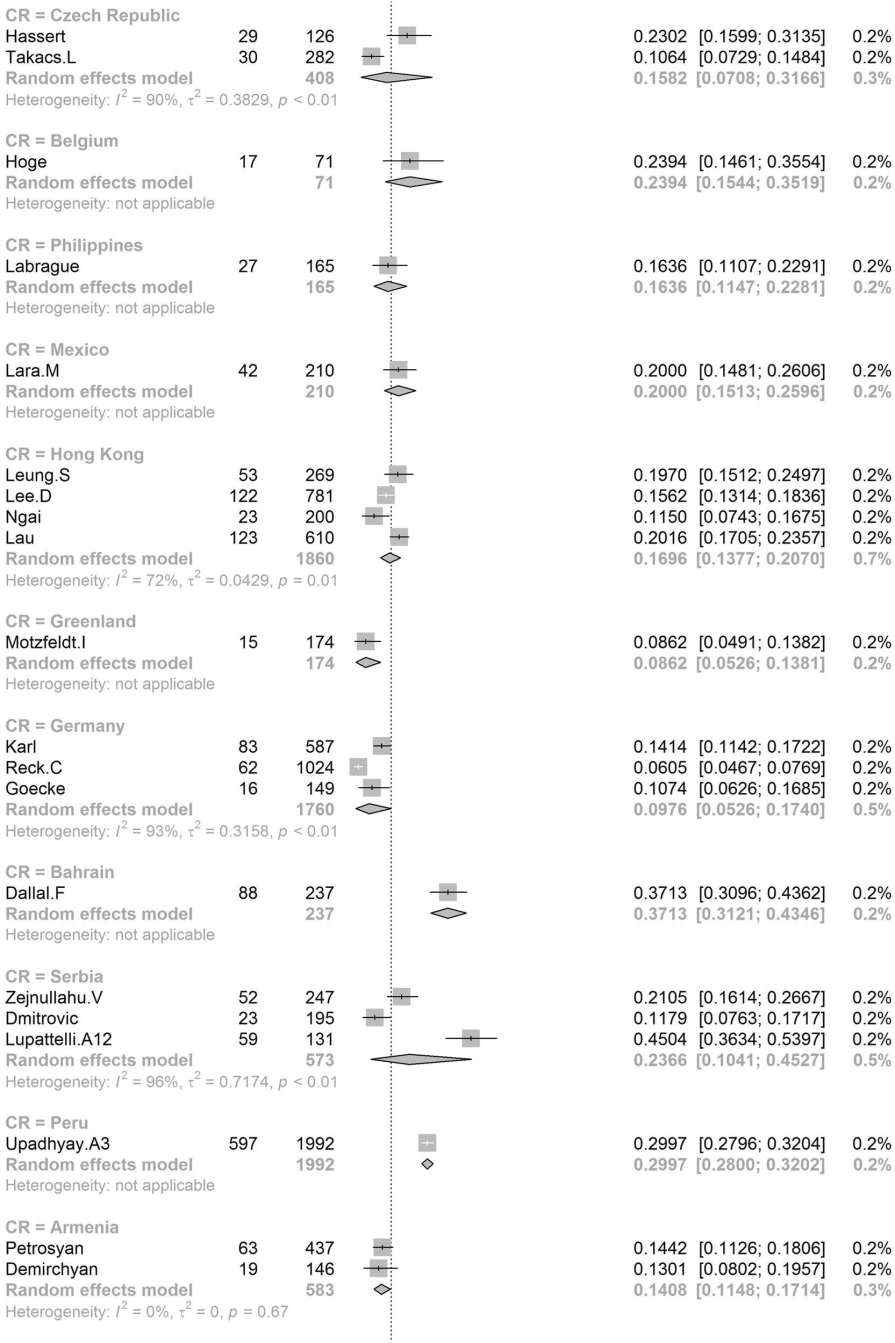

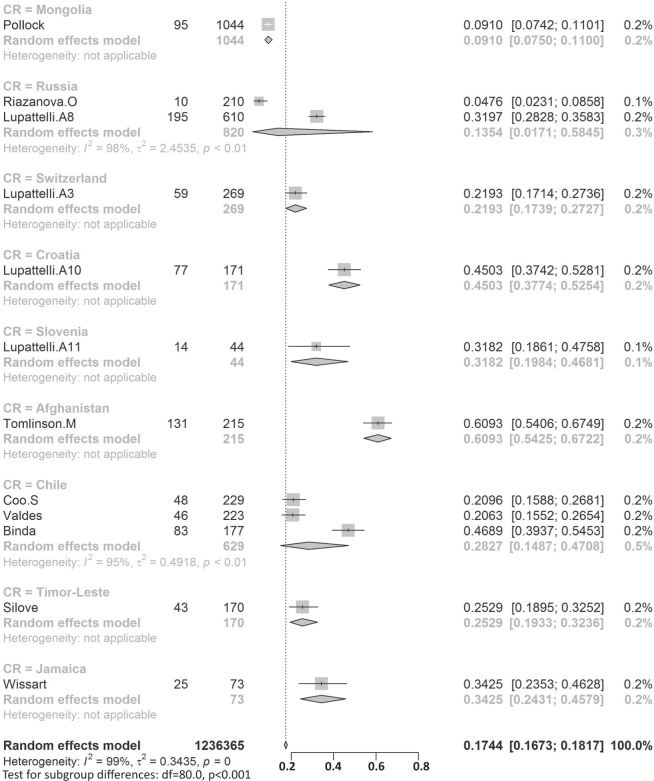
Forest plot of prevalence of postpartum depression stratified by countries or regions. (Postpartum depression prevalence varied substantially between different countries and regions, with the lowest prevalence observed in Denmark and highest one in Afghanistan).

**Fig. 4:**
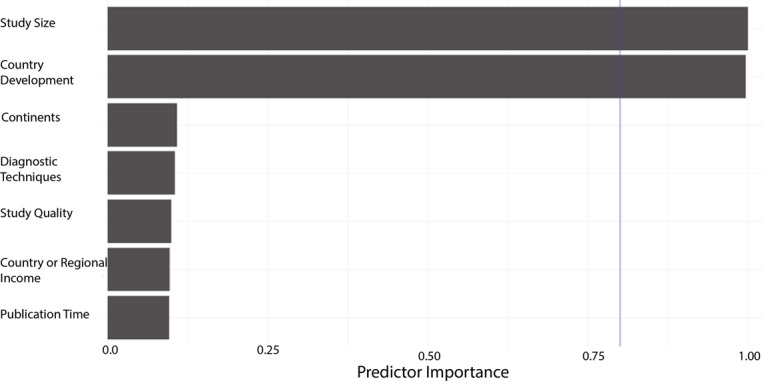
Multi-variable meta-regression for postpartum depression prevalence. (Multi-variable meta-regression was performed in order to find out factors with the highest predictor importance).

In order to further understand the heterogeneity, sensitivity analysis was conducted by performing a set of leave-one out diagnostic tests (Supplementary Table 3) and the results were further verified by using a build-in function in Metafor (Supplementary Table 4 and Supplementary Fig. 1). As a result, four studies were identified as outliers for estimating pooled prevalence. After removing the outliers, the pooled prevalence of postpartum depression increased to 17.44% (95% CI 16.73–18.17, Fig. 3) stratified by countries or regions. To further explore the source of heterogeneity, meta-regression analysis was performed. The univariate meta-regression model indicated that geographical regions (*R*^2^ = 0, *p* = 0.31), diagnostic scales (*R*^2^ = 0, *p* = 0.37), publication year (*R*^2^ = 0, *p* = 0.65), and quality score of the study (*R*^2^ = 0, *p* = 0.55) were not significantly associated with heterogeneity. The source of heterogeneity across the studies, identified by meta-regression analyses, was study size (*R*^2^ = 0.03, *p* < 0.01), development of countries or regions (*R*^2^ = 0.02, *p* < 0.01) and income of countries or regions (*R*^2^ = 0, p < 0.01, Supplementary Table 4). By performing multivariable meta-regression, it was found that the study size and country or region development with the highest predictor importance of 99.99% (Fig. 4, Supplementary Table 5).

To confirm results of the meta-regression, subgroup analysis was performed after removing the outliers. Among geographic regions that had at least five studies, Southern Africa had the highest prevalence rate (39.96%, 95% CI 27.81–53.48), followed by Southern Asia (22.32%, 95% CI 18.48–26.70), South America (21.71%, 95% CI 19.78–23.76), Western Asia (19.83%, 95% CI 17.33–22.58), Northern Africa (18.75%, 95% CI 11.40–29.26), Eastern Asia (17.39%, 95% CI 16.09–18.77), Northern America (17.01%, 95% 15.68–18.44), Eastern Europe (16.62%, 95% CI 10.95–24.43), Southern Europe (16.34%, 95% CI 12.90-20.48), Northern Europe (13.78%, 95% CI 12.47–15.21), Western Africa (13.62%, 95% CI 8.27–21.62), South-Eastern Asia (13.53%, 95% CI 11.00–16.52), and Oceania (11.11%, 95% CI 9.27–13.25, *p* < 0.01, Table 1). Postpartum depression prevalence varied substantially among countries and regions, from 6.48% (Denmark, 95% CI 5.70-7.36) to 60.93% (Afghanistan, 95% CI 54.25-67.22, Fig. 2). Among countries or regions containing at least five relevant studies, South Africa had the highest (38.79%, 95% CI 25.71–53.72) and Spain had the lowest (9.09%, 95% CI 6.97–11.08) postpartum depression prevalence among women (Table 1). Considering the income of countries or regions, those with high incomes had a significantly lower prevalence of 15.54% (95% CI 14.90–16.20, *p* < 0.01). Besides, developed countries (14.85%, 95% CI 14.22–15.51) shared a significantly lower postpartum depression prevalence than that of developing countries (19.99%, 95% CI 18.76–21.27, *p* < 0.01, Table 1). With regard to diagnostic scales, use of the Postpartum Depression Screening Scale (PDSS) resulted in the highest postpartum depression prevalence rate (37.23%, 95% CI 21.47–56.27) and the Structured Clinical Interview for DSM Disorders (SCID) with the lowest prevalence rate (10.11%, 95% CI 3.75–24.48). More than 80% of the studies used the Edinburgh Postnatal Depression Scale (EPDS) as their diagnostic tool with a prevalence rate of 16.86% (95% CI 16.04–17.72, Table 1). Interestingly, a pattern of increased prevalence rates was found in studies having more than 1,000 participants (12.97%, 95% CI 18.40–20.51) than those with less than 1,000 participants (19.44%, 95% CI 18.40–20.51, *p* < 0.01, Table 1). To be noted, postpartum depression appears to be universally prevalent in 1–3 months (17.70%, 95% CI 15.95-19.60), 3–6 months (15.31%, 95% CI 14.31–16.36), 6–12 months (18.19%, 95% CI 13.56–23.96) and greater than 12 months (17.95%, 95% CI 13.80–23.01, *p* = 0.08, Table 1) after birth.

Moreover, results indicated that a significant difference in PPD prevalence was found in the pooled estimate among marital status (married/cohabiting or single/divorced/widowed, 16.37% vs. 28.14%, *p* < 0.01), educational level (less than 12 years or more than 12 years, 19.84% vs. 15.66%, *p* < 0.01), social support from friends/family/parents (YES/NO, 15.15% vs. 32.03%, *p* < 0.01), support from partner (YES/NO, 15.40% vs. 35.99%, p < 0.01), violence (YES/NO, 40.40% vs. 15.65%, *p* < 0.01), gestational age (term or pre-/post-term, 15.48% vs. 22.04%, *p* = 0.01), breast feeding (YES/NO, 16.51% vs. 25.02%, *p* < 0.01), infant death (YES/NO, 43.33% vs. 18.50%, *p* < 0.01), planned pregnancy (YES/NO, 17.36% vs. 28.12%, *p* < 0.01), financial problems (YES/NO, 31.82% vs. 15.92%, *p* < 0.01), partnership (intimate/satisfied or unsatisfied, 17.11% vs. 39.96%, *p* < 0.01), life stress (YES/NO, 28.81% vs. 14.53%, *p* < 0.01), smoker (YES/NO, 25.17% vs. 15.74%, *p* < 0.01), alcohol use (YES/NO, 20.97% vs. 14.49%, *p* = 0.02) and living conditions (satisfied/well or unsatisfied, 20.35% vs. 38.82%, *p* = 0.02). However, minimal differences were observed among number of children (1 or less/2 or more, 17.74% vs. 19.38%, *p* = 0.68), employment (unemployed or paid employment, 21.08% vs. 18.29%, *p* = 0.11), sex of infant (male or female, 17.97% vs. 20.77%, *p* = 0.13), parity (primiparous or multiparous, 17.39% vs. 17.63%, *p* = 0.92), maternal age (adolescent or adult, 23.34% vs. 20.35, *p* = 0.52), residence (urban or rural/suburb, 12.10% vs. 14.46%, *p* = 0.73), religion (YES/NO, 15.11% vs. 17.47%, *p* = 0.77), mode of delivery (spontaneous or instrumental/cesarean delivery, 18.31% vs. 21.01%, *p* = 0.40), ethnicity (indigenous or nonindigenous/immigrant, 14.76% vs. 17.06%, *p* = 0.27), location of delivery (home or health facility, 14.88% vs. 11.08%, *p* = 0.49), sleep (satisfactory or unsatisfactory, 17.20% vs. 30.78%, *p* = 0.08), family type (nuclear or extended, 20.66% vs. 21.15%, *p* = 0.82) and gender preference (YES/NO, 17.78% vs. 13.82%, *p* = 0.40, Table 2).

**Table 2:** Pooled estimates of risk factors for PPD.

	Studies	PPD	Participants	Prevalence (95% CI)	*P* value	*I*^2^
Age (in years)					0.52	
<20	54	3412	24310	23.34 (18.58–28.89)		97.60%
>20	54	30781	1133022	20.35 (14.01–28.62)		99.90%
Alcohol use					0.02	
Yes	18	1387	10311	20.97 (15.73–27.39)		92.20%
No	17	11000	80111	14.49 (12.52–16.72)		91.60%
Breast feeding					<0.01	
Yes	49	7747	62410	16.51 (13.64–19.84)		98.30%
No	50	1225	5568	25.03 (20.99–29.54)		89.40%
Parity					0.92	
Primiparous	120	21666	834265	17.39 (13.44–22.19)		99.70%
Multiparous	120	15834	166938	17.63 (15.48–20.00)		98.40%
Education (in years)					<0.01	
<12	130	17826	106174	19.84 (17.97–21.86)		97.70%
>12	127	15676	128967	15.66 (14.44–16.95)		95.40%
Employment					0.11	
Paid employment	117	6802	48324	18.29 (15.80–21.08)		97.30%
Unemployment	118	7331	37230	21.08 (19.05–23.25)		95.10%
Ethnicity					0.27	
Indigenous	41	4114	38404	14.76 (12.19–17.76)		97.50%
Nonindigenous or immigrant	41	4783	38948	17.06 (14.22–20.35)		96.90%
Family type					0.82	
Nuclear	34	2378	11362	20.66 (17.95–23.67)		91.50%
Extend	34	2763	12870	21.15 (18.30–24.31)		92.70%
Financial problems					<0.01	
Yes	23	1434	5480	31.82 (25.57–38.79)		95.20%
No	23	1571	12286	15.92 (12.49–20.07)		96.10%
Gender preference					0.4	
Yes	17	987	10288	17.78 (11.53–26.39)		97.90%
No	17	1190	13107	13.82 (9.12–20.40)		98.10%
Gestational age					<0.01	
Term	42	13136	104944	15.48 (12.85–18.53)		98.60%
pre- or post-term	42	3169	21341	22.04 (18.14–26.52)		93.10%
Infant death					0.01	
Yes	17	479	1294	43.44 (25.89–62.80)		94.50%
No	15	1493	18042	18.50 (11.53–28.35)		98.90%
Infant illness					<0.01	
Yes	41	2597	11180	29.00 (21.78–37.46)		97.80%
No	40	5793	44536	17.95 (14.74–21.67)		98.10%
Infant weight					0.14	
Normal	29	2490	18083	17.41 (14.00–21.45)		96.90%
Abnormal	29	629	3582	23.55 (16.75–32.05)		94.30%
Infant sex					0.13	
Male	78	10252	72227	17.97 (15.62–20.58)		97.40%
Female	77	9760	67494	20.77 (18.20–23.60)		97.20%
Life stress					<0.01	
Yes	35	3735	23151	28.81 (23.49–34.78)		96.70%
No	35	2538	20435	14.53 (11.84–17.70)		96.10%
Delivery Location					0.49	
Home	11	316	5984	14.88 (6.93–29.10)		96.90%
Health facility	11	969	15285	11.08 (7.29–16.50)		97.40%
Marital status					<0.01	
Married or cohabiting	91	22284	168779	16.37 (14.55–18.37)		98.50%
Single (unmarried, widowed or divorced)	91	5247	26763	28.14 (24.96–31.55)		94.40%
Maternal health problems (perinatal period)					0.01	
Yes	68	7970	77815	23.97 (19.40–29.23)		98.30%
No	68	12014	143334	16.71 (13.81–20.09)		99.00%
Mode of delivery					0.4	
Spontaneous	125	25499	972401	18.31 (14.34–23.08)		99.70%
Instrument or CS	126	19053	555861	21.01 (16.83–25.90)		99.60%
Number of children					0.68	
1 or less	20	645	3355	17.74 (13.18–23.45)		92.80%
2 or more	20	710	4035	19.38 (14.15–25.95)		94.50%
Partner support (assistance)					<0.01	
Yes	31	10646	89016	15.40 (12.48–18.86)		97.80%
No	31	2994	6793	35.99 (28.43–44.31)		95.40%
Partnership					<0.01	
Intimate or satisfied	42	4035	23404	17.11 (14.54–20.04)		96.50%
Unsatisfied	42	1569	4921	39.96 (33.33–46.98)		94.00%
Pregnancy plan					<0.01	
Planned	81	5840	45033	17.36 (14.91–20.12)		97.40%
Unplanned	81	4237	22925	28.12 (24.18–32.43)		96.70%
Religion					0.77	
Yes	9	816	14470	15.11 (6.25–32.23)		99.20%
No	9	489	3448	17.47 (10.65–27.33)		96.00%
Residence					0.73	
Urban	15	2229	87091	12.10 (4.83–27.18)		99.70%
Rural	15	1645	21927	14.46 (8.61–23.26)		99.00%
Sleep					0.08	
Satisfied	11	948	10483	17.20 (10.10–27.75)		98.40%
Unsatisfied	12	1045	4643	30.78 (19.49–44.95)		98.20%
Smoking					<0.01	
Yes	36	1238	6948	25.17 (20.84–30.07)		88.80%
No	35	13314	97611	15.74 (13.93–17.73)		95.70%
Social support					<0.01	
Yes	55	4467	29888	15.15 (12.93–17.68)		96.60%
No	55	2741	10078	32.03 (27.42-37.02)		95.00%
Violence					<0.01	
Yes	37	2210	6987	40.40 (34.11–47.02)		95.00%
No	37	14631	111554	15.65 (12.88–18.89)		98.60%

CS = Cesarean section.

## Discussion

The global prevalence of PPD was found to be approximately 17.22% (95% CI 16.00-18.51) in the largest meta-analysis of PPD to-date. Study findings revealed significant differences between geographic regions, with Southern Africa having the highest prevalence rate (39.96%, 95% CI 27.81–53.48). Furthermore, country development and income inequalities had a major effect on PPD epidemiology. Furthermore, it was discovered that the prevalence of various variables such as marital status, educational level, violence, partnership, life stress, smoking, alcohol use, and living conditions differed significantly. However, no significant difference in PPD was noted in relation to infant gender and gender preference.

There have been recent systematic reviews of PPD worldwide [10–13] or in specific regions, including Asia [14, 15] and Africa [16], but to the researchers’ knowledge this is the most comprehensive review with the largest number of studies on PPD globally. Results showed that the estimates are significantly higher than the widely cited prevalence rate of 13% (95% CI: 12.3–13.4%), derived from a meta-analysis of studies from developed countries [10] and lower than the 19% prevalence rate for PPD derived from studies of relatively low- and middle-income countries [12]. Interestingly, the current study findings are more similar to pooled estimates of PPD when using the EPDS [13]. Furthermore, current findings revealed that the prevalence of PPD was closely linked to country development and national or regional income. This is in line with previous research, which has shown that developing countries with low or lower-middle income have a higher prevalence of PPD. Importantly, the PPD prevalence rate varied based on the scale used when comparisons were made between countries with different socioeconomic structures. Previous studies had observed inconsistent PPD prevalence with 1.9% to 82.1% in developed countries, and 5.2% to 74% in developing countries [17]. Moreover, another study evaluated PPD studies from 40 countries and reported the prevalence of PPD to be 10–15% and found this prevalence to vary between 0% and 60% in developed and developing countries, respectively [9]. One detailed study found that PPD prevalence in developing countries was greater in developing countries (31.1%) than that of developed countries (21.5%) in rural areas [18]. It is worth noting that even among nations in similar economic strata, there are differences in national estimates of the prevalence of postpartum depression. For example, the prevalence of PPD in the United Kingdom is 21.5%, while that in New Zealand, another high-income country, is 10.58%; PPD prevalence in Ghana and Egypt were 3% and 22.99% respectively, while in fact they belong to low-income countries and their per capita GDP is similar.

Almost all geographic regions were represented in this review, with the highest coverage in Eastern Asia (*n* = 96) and the lowest coverage in Central America and Caribbean areas (*n* = 1). As a result, the current study’s pooled estimates of Central America and the Caribbean are underrepresented. Furthermore, the current analysis showed substantial variations between graphic areas on the same continent. Southern Asia, for instance, led with the highest prevalence (22.32%, 95% CI 18.48–26.70), followed by Western Asia (19.83%, 95% CI 17.33–22.58), Eastern Asia (17.39%, 95% CI 16.09–18.77), and South-Eastern Asia (13.53%, 95% CI 11.00–16.52). This was mirrored in the vast spectrum of PPD prevalence in Asian countries, which ranged from 9.29 % (Korea) to 60.93% (Afghanistan). These findings matched those of a previous study, which found that Asians made up 3.5% to 63.3% of the population [19]. To be noted, current study results indicated the prevalence in Southern Asia and Western Asia were significantly higher than that of Northern America, Europe, and Oceania. This is largely in accordance with the results from previous studies [20]. Differences in cultural traditions may play a role in this disparity. In those areas, girls tended to marry at an early age. But when it comes to age as a risk factor, the literature is conflicting. Some studies discovered that mothers with younger children are more likely to develop PPD [21, 22], while other studies discovered that older age is related to PPD [23, 24]. No differences were found in a case-control study conducted by Balaha and colleagues [25]. To be noted, in the current study, the pooled estimates suggest a marginally higher PPD prevalence in adolescent mothers than adult mothers without significant difference. In addition, relationships with mothers-in-law were closely correlated with PPD in those women [23]. In these research participants, mothers-in-law tend to be essential to the survival of the marriage, as sons remain closely attached to their families of birth, frequently living with and even working within the family company their entire lives. Mothers-in-law often exercise a considerable amount of leverage over their daughters-in-law and grandchildren’s lives. As a result, having a healthy relationship with one’s mother-in-law is crucial in a woman’s life, and having a negative one can lead to depression. Moreover, other risk factors, such as postpartum rituals including the period of 40 days resting, restricted activities, and diets [26], multiple children [27] and unemployment [21, 28] were also correlated with a high prevalence rate.

Previous studies indicated that the past history of depression significantly enhanced the chance of PPD [29, 30]. That explains why studies on new mothers having perinatal depression were not included in pooled estimates. Fifteen factors were found related to an elevated risk of PPD in the current review. In terms of breastfeeding, it is generally thought to be a possible preventive factor, and it was found that starting breastfeeding reduced the likelihood of depression in this current research. Breastfeeding has also been linked to an elevated risk of PPD in several trials. Underlying variables such as the mother’s background traits may be a source of confusion. Moreover, breastfeeding duration and breastfeeding self-efficacy may also be correlated with PPD [31, 32]. Besides, according to the findings of the study, postpartum women face a variety of stressful life activities, including relationships, financial problems, social support, and maternal stressors. More than three-quarters of depressed postpartum women (78.2%) reported they had strained ties with their mothers-in-law [21]. With a strong correlation with their counterparts, almost half of postpartum mothers with depression reported having more than one stressful life events, such as low income (58.1%) and unplanned pregnancy (60.4%) [21]. Baker et al. found that low socioeconomic status, unwanted pregnancy and stressful life events during pregnancy have been associated with postpartum mental disorders [33]. Current study results are consistent with previous studies that unplanned pregnancy and a lack of family care were found to be important correlates of PPD. Support should be considered a protective factor based on the literature described previously [21, 34, 35]. Our findings are in line with previous research that support from partners, family members, and friends is shown to be adversely associated with the likelihood of PPD in the current study. The current study also found that women in financial difficulties or experiencing stressful events were at increased risk for PPD. This finding was confirmed in systematic reviews and meta-analyses conducted in low- and middle-income countries [36]. Due to lack of financial support, women in financial difficulties may be in a state of poverty, which leads to stress [36]. To be noted, our results indicated that mothers experiencing domestic violence were almost three times more likely than their counterparts to suffer from depression. Other studies found that postpartum depression was linked to mothers having a history of domestic violence and abuse from their husbands or family members [14, 37, 38]. Violence is harmful to one’s mental health. Mothers who are abused experience feelings of helplessness and despair, as well being at high risk to commit suicide. Unexpectedly, in our meta-analysis, an infant’s gender is not related to postpartum depression. Although female infant sex is favorably linked to PPD, according to research conducted in Asia (China, India), Africa (Nigeria), and Turkey, with qualitative research suggesting a role for male gender preference [39–41]. However, recent studies indicated that several biological mechanisms could underlie our observed findings, including sex-differential shifts in maternal reproductive and other hormones or immune activity [42–44].

There are strengths and limitations of this study to consider. It is the largest population-based study to-date, and allows for the investigation of factors which have not been previously studied in various regions. This study was a meta-analysis, so the pooled estimates of risk factors resulted in improved accuracy compared with findings from a single study. Limitations were also identified. First, although this was the most comprehensive PPD prevalence study, there remains a significant lack of research studies from developing countries with lower-middle or low incomes. Second, since the current research focused on cross-sectional research studies, recall bias may be present. Third, the diagnostic techniques used were based on participants’ self-reports which can result in reporting bias. Fourth, genes and polymorphisms are reported to be associated with PPD. However, in current study, we cannot further pool estimate those data due to the limited number of study on specific genes.

Postpartum depression affected one out of every five women after they gave birth which was triggered by a variety of causes. This necessitates the attention and committed intervention of primary care providers, clinicians, health authorities, and society as a whole.

## Supplementary information

Supplementary materials
