# Correction: Mapping global prevalence of depression among postpartum women

**DOI:** 10.1038/s41398-021-01692-1

**Published:** 2021-12-20

**Authors:** Ziyi Wang, Jiaye Liu, Huan Shuai, Zhongxiang Cai, Xia Fu, Yang Liu, Xiong Xiao, Wenhao Zhang, Elise Krabbendam, Shuo Liu, Zhongchun Liu, Zhihui Li, Bing Xiang Yang

**Affiliations:** 1 School of Nursing, Wuhan University, Wuhan, Hubei China; 2 Department of Thyroid and Parathyroid Surgery, West China Hospital, Sichuan University, Chengdu, Sichuan China; 3 Department of Orthopedics, Chengkou People’s Hospital, Chongqing, China; 4 Department of Nursing, Renmin Hospital of Wuhan University, Wuhan, Hubei China; 5 Department of outpatients, West China Hospital, Sichuan University, Chengdu, Sichuan China; 6 Department of Obstetrics and Gynecology, Second Affiliated Hospital, Chongqing Medical University, Chongqing, China; 7 Chengdu Women’s and Children’s Central Hospital, School of Medicine, University of Electronic Science and Technology of China, Chengdu, China; 8 Department of Obstetrics and Gynecology, Key Laboratory of Birth Defects and Related Diseases of Women and Children, Ministry of Education, West China Second University Hospital, Sichuan University, Chengdu, China; 9 Department of Medical Library, Erasmus MC-University Medical Center, Rotterdam, The Netherlands; 10 Department of Psychiatry, Renmin Hospital of Wuhan University, Wuhan, Hubei China

**Keywords:** Depression, Human behaviour

Correction to: *Translational Psychiatry* (2021) **11**:543, 10.1038/s41398-021-01663-6, published online 20 October 2021

The original version of this article unfortunately contained a mistake. Due to a typesetting error Fig. 4 was omitted. We apologize for the error. The original article has been corrected.

