# Supplementary material for: Mapping global prevalence of depression among postpartum women
Source: Transl Psychiatry. 2021 Oct 20;11:543. doi: 10.1038/s41398-021-01663-6 (PMC8528847; doi:10.1038/s41398-021-01663-6)
Supplement: Supplementary file 1 — Supplementary materials [file 41398_2021_1663_MOESM1_ESM.pdf]

Mapping global prevalence of depression among postpartum women

Supplementary method. Searching strategy for global prevalence of postnatal depression.

Database searched	via	Years of coverage	References	After de-duplication
Embase	Embase.com	1971 - Present	6610	6500
Medline ALL	Ovid	1946 - Present	6767	1901
Web of Science Core Collection	Web of Knowledge	1975 - Present	5521	1852
Cochrane Central Register of Controlled Trials	Wiley	1992 - Present	462	146
PsycINFO			2158	268
Other sources: Google Scholar			200	91
**Total**			**21718**	**10758**

Embase – 6610 refs

('postnatal depression'/de OR (((postnatal* OR postpartum* OR post-natal* OR post-partum* OR puerper* OR maternal* OR after-delivery OR post-delivery OR after-birth* OR post-birth*) NEAR/6 (depress*))):ab,ti,kw) **AND** ('epidemiological data'/de OR 'epidemiology'/de OR 'geographic distribution'/de OR 'patient volume'/de OR prevalence/exp OR 'incidence'/de OR geography/de OR 'geographic names'/exp OR 'cross-sectional study'/de OR (epidemiolog* OR ((geograph* OR global*) NEAR/3 (distribut*)) OR (patient* NEAR/3 volume*) OR prevalen* OR incidenc* OR population-based* OR cross-sectional*):ab,ti,kw) NOT ((animal/exp OR animal*:de OR nonhuman/de) NOT ('human'/exp)) NOT ([Conference Abstract]/lim)

Medline – 6767 refs

(Depression, Postpartum/ OR (((postnatal* OR postpartum* OR post-natal* OR post-partum* OR puerper* OR maternal* OR after-delivery OR post-delivery OR after-birth* OR post-birth*) ADJ6 (depress*))).ab,ti,kf.) **AND** (Epidemiological Monitoring/ OR Epidemiology/ OR

Epidemiology.fs. OR exp Incidence/ OR exp Prevalence/ OR Incidence/ OR Geography/ OR exp Geographic Locations/ OR Epidemiologic Studies OR Cross-Sectional Studies/ OR (epidemiolog* OR ((geograph* OR global*) ADJ3 (distribut*)) OR (patient* ADJ3 volume*) OR prevalen* OR incidenc* OR population-based* OR cross-sectional*).ab,ti,kf.) NOT (exp Animals/ NOT Humans/) NOT (news OR congres* OR abstract* OR book* OR chapter* OR dissertation abstract*).pt.

Cochrane – 462 refs
((((postnatal* OR postpartum* OR post-natal* OR (post NEXT/1 partum*) OR puerper* OR maternal* OR (after NEXT/1 delivery) OR (post NEXT/1 delivery) OR (after NEXT/1 birth*) OR (post NEXT/1 birth*)) NEAR/6 (depress*))):ab,ti,kw) **AND** ((epidemiolog* OR ((geograph* OR global*) NEAR/3 (distribut*)) OR (patient* NEAR/3 volume*) OR prevalen* OR incidenc* OR (population NEXT/1 based*) OR (cross NEXT/1 sectional*)):ab,ti,kw)

Web of Science – 5521 refs
TS=(((((postnatal* OR postpartum* OR post-natal* OR post-partum* OR puerper* OR maternal* OR after-delivery OR post-delivery OR after-birth* OR post-birth*) NEAR/5 (depress*)))) AND ((epidemiolog* OR ((geograph* OR global*) NEAR/2 (distribut*)) OR (patient* NEAR/2 volume*) OR prevalen* OR incidenc* OR population-based* OR cross-sectional*)) NOT ((animal* OR rat OR rats OR mouse OR mice OR murine OR dog OR dogs OR canine OR cat OR cats OR feline OR rabbit OR cow OR cows OR bovine OR rodent* OR sheep OR ovine OR pig OR swine OR porcine OR veterinar* OR chick* OR zebrafish* OR baboon* OR nonhuman* OR primate* OR cattle* OR goose OR geese OR duck OR macaque* OR avian* OR bird* OR fish*) NOT (human* OR patient* OR women OR woman OR men OR man))) AND DT=(Article OR Review)

PsycINFO – 2158 refs
(Postpartum Depression/ OR (((postnatal* OR postpartum* OR post-natal* OR post-partum* OR puerper* OR maternal* OR after-delivery OR post-delivery OR after-birth* OR post-birth*) ADJ6 (depress*))).ab,ti.) **AND** (Epidemiology/ OR Geography/ OR (epidemiolog* OR ((geograph* OR global*) ADJ3 (distribut*)) OR (patient* ADJ3 volume*) OR prevalen* OR incidenc* OR population-based* OR cross-sectional*).ab,ti.) NOT ((animal.po. OR exp animals/) NOT human.po.) NOT (news OR congres* OR abstract* OR book* OR chapter* OR dissertation abstract*).pt.

Google Scholar – top 200 refs (random)
"postnatal|postpartum|puerperal puerperium|maternal depression" epidemiology|geography|"global distribution"|"patient volume"|prevalence|incidence

Supplementary table 1. Characteristics for including studies.

Author	Country	P. year	Study time	Study design	Sample Source	Mean age	Measurement method	Cut off value	Screen time	Quality	Cases	Total
Ramchandani[1]	South Africa	2009	2007	Cohort	Community		PDQ	20	24 weeks	8	170	1035
Stewart[2]	Malawi	2010	2006	Cross-sectional	HI	24.4	DSM-IV		36 weeks	8	70	501
Ndokera[3]	Zambia	2010	2009	Cross-sectional	Community		SRQ-20	8	8-48 weeks	9	27	278
Guo[4]	Ghana	2013	2010-2011	Cohort	HI	29.1	PHQ-9	10	3-12 months	8	24	293
									3 months		26	293
									12 months		21	293
	Cote d'Ivoire								3-12 months		51	361
									3 months		43	361
									12 months		58	361
Weobong[5]	Ghana	2015	2008-2009	Cohort	Community		PHQ-9	10	4 weeks	9	455	13360
Hassanein[6]	Egypt	2014	2012	Cross-sectional	HI	23.9	EPDS	13	12 weeks	9	113	290
Mohammed[7]	Egypt	2014	2011-2012	Cross-sectional	Community	29	EPDS	10	56 weeks	9	99	200
Mahenge[8]	Tanzania	2015	2011-2012	Cross-sectional	HI	29	HSCL-25/PHQ-9		4–36 weeks		923	1180
Khalifa[9]	Sudan	2015	2013-2014	Cross-sectional	HI	28	EPDS	12	12 weeks	9	20	236
Shamu[10]	Zimbabwe	2016	2011	Cross-sectional	HI		CES-D		6 weeks	9	180	842
Stellenberg[11]	South Africa	2015	2011	Cross-sectional	Community		EPDS/BDI	15	4-14 weeks	8	80	159

									4 weeks		21	53
									10 weeks		30	52
									14 weeks		25	54
Azale[12]	Ethiopia	2016	2014	Cross-sectional	Community	28.8	PHQ-9	5	4-28weeks	9	47	385
									4-12weeks		49	385
									16-24 weeks		43	385
									28+ weeks		48	385
Wemakor[13]	Ghana	2018	2014	Cross-sectional	Community	27.9	CES-D		6-23 months	7	117	384
Rogathi[14]	Tanzania	2017	2014-2015	Cohort	HI		EPDS	13	6 weeks	8	122	1013
Anokiye[15]	Ghana	2018	2017	Cross-sectional	HI	27	PHQ-9	10	0–48 weeks	8	10	257
Glasser[16]	Israel	2000	2000	Cohort	HI	27.4	EPDS	10	6 weeks	9	65	288
Yoshida[17]	Japan	2001	2000	Cross-sectional	HI	30	EPDS	8/9	12 weeks	9	14	93
Chandran[18]	India	2002	2002	Cross-sectional	HI	22.8	CIS-R/ICD-10		6-12 weeks	8	71	359
Inandi[19]	Turkey	2002	2001	Cross-sectional	Community /HI	26.9	EPDS	13	4-48 weeks	6	684	2514
Vincent Koo[20]	Malaysia	2003	2002	Cohort	HI	29.5	EPDS	13	6 weeks	8	54	246
Wang[21]	China	2003	2001-2002	Cross-sectional	Not mentioned	28.66	BDI	10	6 weeks	8	49	197
Heh[22]	Taiwan	2004	2003	Cross-sectional	HI	28	EPDS		4 weeks	9	39	186
Fisher[23]	Vietnam	2004	2000	Cross-sectional	HI	28	EPDS	13	6 weeks	8	166	506
Aydin[24]	Turkey	2005	2003	Cross-sectional	HI		EPDS	13	4-48 weeks	9	294	728

Wang[25]	Taiwan	2006	2005	Cross-sectional	HI		BDI		6 weeks	9	33	83
Chien[26]	Taiwan	2006	2003	Cross-sectional	HI		CES-D	15	4-6 weeks	8	61	202
Rahman[27]	Pakistan	2007	2006	Prospective	Community	27.5	SCAN/ICD-10		48 weeks	8	73	129
Green[28]	United Arab Emirate	2006	2005	Prospective	HI/Community	32	EPDS	13	12-24 weeks		13	71
									12 weeks	9	19	86
									24 weeks		7	56
Ho-yen[29]	Nepal	2007	2006	Cross-sectional	HI/Community	24.5	EPDS	12	5-10 weeks	8	21	426
Xie[30]	China	2007	2004-2005	Cohort	HI	28.4	EPDS	13	6 weeks	8	52	300
Liabsuetrakul[31]	Thailand	2007	2003-2004	Cohort	HI	27.9	PDRS		6-8 weeks	9	40	400
Chee[32]	Singapore	2008	2006	Cohort	HI		EPDS	12	24-48 weeks	8	72	471
Murakami[33]	Japan	2008	2001-2003	Cohort	Community		EPDS	9	8-36 weeks	9	121	865
Horowitz[34]	USA	2011	2010	Descriptive	Community /HI		EPDS/SCID-IV	10	4-6 weeks	9	674	5169
Yusuff[35]	Malaysia	2015	2009-2010	Cohort	HI	26.7	EPDS	12	24 weeks	9	195	1362
Ogbo[36]	Australia	2018	2017	Cohort	HI		EPDS	13	6 weeks	8	425	12757
Ahmad[37]	Malaysia	2018	2016	Cross-sectional	HI		EPDS	12	6-16 weeks	8	252	5727
Osama M[38]	Egypt	2019	2016-2017	Cross-sectional	HI	25.6	DASS-42	10	8-16 weeks	8	114	500
Gausia[39]	Bangladesh	2009	2004-2005	Cohort	Community	26.9	EPDS	10	6-8 weeks	8	76	346
Gao[40]	China	2009	2006-2007	Cross-sectional	HI	29.34	EPDS	13	6-8 weeks	8	18	130
Yurdagul[41]	Turkey	2010	2009	Cross-sectional	HI	28.9	EPDS	13	8-48 weeks	9	165	785

McMahon[42]	Australia	2011	2010	Cohort	HI	29.9	SCID/ICD-10		16 weeks	8	43	541
Yong Zhang[43]	China	2012	2006-2007	Cross-sectional	HI	28	EPDS	13	30-42 days	9	67	215
Bowen[44]	Canada	2012	2011	Longitudinal	Community		EPDS	12	4-6 weeks	9	75	593
Mishina[45]	Japan	2012	2008-2010	Cross-sectional	HI		EPDS	9	0-16 weeks	9	78	630
									0-4 weeks		87	631
									4-8 weeks		102	794
									8-12 weeks		80	827
									12-16 weeks		41	312
Bener[46]	Qatar	2012	2010-2011	Cross-sectional	HI	33.5	EPDS	12	24 weeks	8	243	1379
Simone[47]	Canada	2013	2005-2006	Cross-sectional	Community /HI		EPDS	13	6 months-1 year	8	458	6126
Mathisen[48]	Argentina	2013	2011-2012	Cross-sectional	HI	28.3	EPDS	10	6 weeks	9	32	86
Gaillard[49]	France	2014	2007-2009	Cross-sectional	HI	31	EPDS	12	6-8 weeks	9	44	264
Deng[50]	China	2014	2013	Cross-sectional	Community	25.6	EPDS	13	4 weeks		499	1823
Hinai[51]	Oman	2014	2010	Cross-sectional	HI	29.2	EPDS	13	8 weeks	8	30	282
Kirkan[52]	Turkey	2015	2014	Cross-sectional	Community	21.17	EPDS	12/13	6 weeks	9	126	360
Verreault[53]	Canada	2014	2005-2009	Cross-sectional	HI	32.1	EPDS	10	12 weeks	9	37	226
Giri[54]	Nepal	2015	2012	Cross-sectional	HI	24	EPDS	10	6-8 weeks	8	105	346
Shimizu[55]	Japan	2015	2014	Cohort		30.7	GHQ	6	4 weeks	9	32	65
							EPDS	9	16 weeks		17	65
									4-16 weeks		25	65

Turkcapar[56]	Turkey	2015	2008	Cross-sectional	HI	26.12	EPDS	13	6-8 weeks	9	83	540
Boratav[57]	Turkey	2016	2010	Cross-sectional	HI		EPDS	12	12 weeks	8	42	87
Kunwar[58]	Nepal	2015	2014	Cross-sectional	HI	24.52	EPDS	13	4 weeks	9	29	100
Zainal[59]	Malaysia	2012	2008-2009	Cross-sectional	HI	27.4	M.I.N.I		6-8 weeks	8	28	411
Rebelo[60]	Brazil	2016	2009-2011	Cohort	HI		EPDS	11	30-45 days	8	30	177
H. Corrêa[61]	Brazil	2016	2010	Cross-sectional	HI		EPDS	11	4-12 weeks	8	597	3060
Bhusal[62]	Nepal	2016	2015	Cross-sectional	HI	22.75	EPDS	12/13	4-14 weeks	9	59	346
Chi[63]	China	2016	2015	Cross-sectional	Online		CES-D	16	1-3 years	9	152	506
									48 weeks		54	193
Jaeschke[64]	Poland	2017	2009-2013	Cross-sectional	HI	30.2	EPDS	13	6-12 weeks	9	66	434
Koutra[65]	Spain	2018	2007	Cohort	HI	29.51	EPDS	13	8 weeks	9	141	1037
Thang[66]	Vietnam	2017	2013	Cross-sectional	HI	28.76	EPDS	13	4-24 weeks	8	116	600
Afolabi[67]	UK	2020	2016	Cross-sectional	Online		EPDS	12	2 years	8	23	32
	Nigerian	2020	2016	Cross-sectional	Online		EPDS	12	2 years		15	79
Eckerdal[68]	Sweden	2018	2009-2014	Cross-sectional	HI	31.4	EPDS	12	6 weeks	9	505	3888
Xiong[69]	China	2018	2015-2016	Cross-sectional	HI		EPDS	10	6 weeks	8	263	468
Pampaka[70]	Kuwait	2019	2012-2015	Cohort	HI	29	EPDS	10	6 weeks	9	158	1348
Shakeel[71]	Norway	2018	2008-2010	Cohort	HI		EPDS	10	14 weeks	9	60	643
Kerie[72]	Ethiopia	2018	2017	Cross-sectional	HI	26.06	EPDS	10	48 weeks	8	138	408
Vaezi[73]	Iran	2019	2015	Cross-sectional	HI	28	EPDS	12	24 weeks	9	87	200

Ongeri[74]	Kenya	2018	2017	Cross-sectional	HI		EPDS	10/13	6-10 weeks	8	32	171
Petersen[75]	UK	2018	2000-2013	Cohort	Online		ICD-10		48 weeks	8	23623	206517
Sahin[76]	Turkey	2018	2017-2018	Cohort	HI	27.79	EPDS	13	4-6 weeks	9	24	497
Mohammad[77]	Jordan	2018	2017	Cross-sectional	HI		EPDS	12	6-8 weeks	9	181	365
Shwartz[78]	Israel	2019	2015	Cross-sectional	HI		EPDS	10	6-24 weeks	9	116	1121
Agbaje[79]	Nigeria	2019	2017	Cross-sectional	Community	28.9	EPDS	13	4-12 weeks	8	92	270
Meky[80]	Egypt	2020	2015-2016	Cross-sectional	HI		EPDS	13	8-16 weeks	9	55	370
Bitew[81]	Ethiopia	2019	2014	Cohort	Community	26.8	PHQ-9	5	4-12 weeks	8	274	1240
Nampijja[82]	Uganda	2019	2015-2016	Cross-sectional	HI	28	PHQ-9/M.I.N.I	10	4-12 weeks	9	98	300
Sharmin[83]	Bangladesh	2019	2018	Cohort	HI	26.24	EPDS		24-32 weeks	9	88	400
Topatan[84]	Turkey	2019	2014-2015	Cross-sectional	HI	17.76	EPDS	12	4-48 weeks	8	19	84
Azad[85]	Bangladesh	2019	2017	Cross-sectional	Community		EPDS	10	48 weeks	9	148	376
Nurbaeti[86]	Indonesia	2019	2016	Cross-sectional	HI	27	EPDS	12	4 weeks	9	33	166
Lin[87]	Taiwan	2019	2016-2017	Cross-sectional	HI		EPDS	10	6-8 weeks	9	29	344
Xiong[88]	China	2020	2016-2017	Cross-sectional	HI		EPDS	10	6-48 weeks	9	202	1124
Dlamini[89]	Eswatini	2019	2018	Cross-sectional	HI		EPDS	13	6 weeks	8	54	114
Shitu[90]	Ethiopia	2019	2018	Cross-sectional	Community		EPDS	8	48 weeks	8	141	596
Arante[91]	Brazil	2020	2013-2015	Cross-sectional	HI	27.5	PHQ-9	5/10	24-36 weeks	9	147	346
Roumieh[92]	Syria	2019	2017	Cross-sectional	HI	27.7	EPDS	13	4-6 weeks	9	312	1105
Simone[93]	Brazil	2020	2019	Cohort	HI		EPDS	10	48 weeks	8	613	3838

Nasr[94]	Saudi Arabia	2020	2019	Cross-sectional	HI		EPDS	13	4-24 weeks	7	51	128
Xiong[95]	China	2020	2016-2017	Cross-sectional	HI		EPDS	10	6 weeks	9	418	1230
Asaye[96]	Ethiopia	2020	2018	Cross-sectional	HI	28.7	EPDS	13	6 weeks	9	129	526
Motoko[97]	Japan	2006	2005	Cross-sectional	HI	29.7	EPDS	9	48 weeks	9	19	70
Abbott[98]	New Zealand /Pacific island nation	2006	2000	Cohort	HI	27.8	EPDS	13	6 weeks	9	224	1363
Baker[99]	USA	2005	2002-2003	Prospective	HI	23.47	PDSS		6 weeks		35	151
Teng[100]	Taiwan	2005	2001-2002	Cohort	HI	29	EPDS	12/13	6 weeks	8	24	203
Adewuya[101]	Nigeria	2005	2004	Cross-sectional	HI	28.37	EPDS	9	6 weeks	8	128	876
Chee[102]	Singapore	2005	2002-2003	Cohort	HI	31	EPDS	7	6 weeks	9	19	278
Limlomwongse[103]	Thailand	2006	2003-2004	Cohort	HI		EPDS	10	6-8 weeks	9	88	525
Husain[104]	UK/Pakistan	2006	2005	Cross-sectional	Community	27	EPDS	12	12 weeks	9	53	149
Jardri[105]	France	2006	2003-2004	Prospective	HI		EPDS	8	8 weeks	9	58	363
Azidah[106]	Malaysia	2006	2000	Cross-sectional	HI	30.3	EPDS	12	4-6 weeks	8	78	377
Miyake[107]	Japan	2006	2001-2003	Cohort	HI		EPDS	9	8-36 weeks	8	121	865
Huang[108]	UK	2006	2005	Cohort	Community		EPDS	12	12 weeks	9	9	50
	Taiwan	2006	2005	Cohort	Community		EPDS	12	12 weeks		19	101
Alami[109]	Morocco	2006	2000	Cross-sectional	HI	27	EPDS		36 weeks	9	27	100
Signe[110]	Nepal	2006	2005	Cross-sectional	Community /HI	24.5	EPDS	12	10 weeks	9	21	426
Pitanupong[111]	Thailand	2007	2003-2004	Cohort	HI	27.9	EPDS	6/7	6-8 weeks	9	38	351

Austin[112]	Australia	2007	2006	longitudinal	HI	31.1	EPDS	13	8 weeks	9	53	575
Sari[113]	Indonesia	2007	2000	Cohort	Community	24	EPDS	12/13	6 weeks	9	18	274
Secco[114]	Canada	2006	2005	longitudinal	HI	16.79	BDI	10	4 weeks	8	30	69
Dindar[115]	Turkey	2007	2003-2004	Cross-sectional	Community	26.7	EPDS	12	48 weeks	8	174	679
Montazeri[116]	Iran	2007	2006	Cohort	HI		EPDS	13	6-14 weeks	9	20	100
Huang[117]	Taiwan	2008	2003	Cross-sectional	Community		EPDS	13	24 weeks	8	27	106
Figueiredo[118]	Portugal	2007	2006	Cross-sectional	HI	22.83	EPDS	12	8-12 weeks	9	19	108
Kara[119]	Turkey	2007	2001	Cross-sectional	HI		BDI	17	4-12 weeks	9	28	163
Akman[120]	Turkey	2007	2005	Cohort	HI		SCID		6 weeks	8	19	302
Chen[121]	Taiwan	2007	2006	Cross-sectional	Community	29.31	EPDS	12	1-24 months	9	52	122
Edge[122]	UK	2007	2006	Cross-sectional	Community /HI		EPDS	12/13	6 weeks	8	39	200
Tychey[123]	France	2008	2003-2004	Cross-sectional	HI		EPDS	12	8 weeks	9	17	181
Orhon[124]	Turkey	2007	2006	Cross-sectional	HI		EPDS	12	4-48 weeks	9	35	103
Baker[125]	USA	2008	2006	Cross-sectional	HI	23.66	PDSS		6 weeks	8	112	498
Edwards[126]	Australia	2008	2003	Cross-sectional	HI	26.3	EPDS	10	6 weeks	8	33	154
Milgrom[127]	Australia	2008	2007	Cross-sectional	HI		EPDS	12	6 weeks	9	925	12361
Eugenia[128]	Mexico	2008	2007	Cross-sectional	HI	27.3	EPDS	13	2-12 weeks		40	163
Watanabe[129]	Japan	2008	2006	Cohort	HI	31.5	EPDS	9	4-12 weeks	9	30	235
Gao[130]	New Zealand	2008	2000	Cohort	HI		EPDS	12/13	6 weeks	8	165	1085
Krause[131]	USA	2009	2004-2006	Cross-sectional	HI/Commu		EPDS	13	6 weeks	9	45	491

					nity							
Bjerke[132]	Norway	2008	2007	Cohort	HI	28	EPDS	10	6-12 weeks	9	15	197
Howell[133]	USA	2009	2002	Cohort	HI	31	PHQ-2		2-24 weeks	8	132	563
Figueiredo[134]	Portugal	2009	2008	Cohort	HI	27.2	EPDS	10	12 weeks	9	24	91
Grussu[135]	Italy	2009	2004	Cohort	Community	32.5	EPDS	9	6-8 weeks	8	39	297
Glavin[136]	Norway	2009	2005-2006	Cross-sectional	HI	32.4	EPDS	10	6 weeks	8	224	2227
Heron[137]	UK	2009	2003	Longitudinal	HI	28.2	EPDS	13	8 weeks	9	27	203
Faruk[138]	Turkey	2009	2008	Cross-sectional	HI	26.8	EPDS	12	6 weeks	9	11	34
Atsuko[139]	Japan	2009	2002	Cross-sectional	HI		EPDS	9	16 weeks	9	39	169
Signe[140]	Norway	2009	2005-2006	Cross-sectional	HI	30	EPDS	10	7 weeks	9	466	2825
Shelton[141]	UK	2009	2002	Cross-sectional	Community		EPDS	10	16 weeks	8	97	399
Kirpinar[142]	Turkey	2009	2001	Prospective	HI	26.62	EPDS	13	6 weeks	8	67	479
Vicenta[143]	Spain	2011	2005	Cohort	HI		EPDS	13	12-48 weeks	9	25	360
Blom[144]	Netherlands	2010	2002-2006	Cohort	HI		EPDS	12	8 weeks	9	396	4941
Nagy[145]	Saudi Arabia	2011	2008-2009	Cross-sectional	HI	26.3	EPDS/MINI	10	8 weeks	9	14	137
Mohammad[146]	Jordan	2011	2005-2006	Cross-sectional	HI		EPDS	13	6-8 weeks	9	78	353
Lobato[147]	Brazil	2011	2007	Cross-sectional	HI	25.3	EPDS	11	12 weeks	9	128	811
Sara[148]	Sweden	2011	2006-2007	Cohort	HI	30.8	EPDS	12	6 weeks-6 months	9	166	1610
									6 weeks		187	1703
									6 months		145	1516

Ross[149]	Canada	2011	2007-2008	Cohort	HI		EPDS	12	6 weeks	9	3	87
Patricia[150]	Ireland	2012	2008	Descriptive	HI		EPDS	11	6 weeks	8	54	410
Figueiredo[151]	Portugal	2011	2010	Cross-sectional	HI		EPDS	10	12 weeks	9	29	260
Glasser[152]	Israel	2011	2008	Descriptive	HI		EPDS	10/13	12 weeks	8	36	104
Lanes[153]	Canada	2011	2005-2006	Cross-sectional	HI		EPDS	13	12 months	6	1101	6421
Matsumoto[154]	Japan	2011	2007-2010	Cohort	HI		EPDS	9	12 weeks		100	675
Banti[155]	USA	2011	2004-2007	Longitudinal	HI	32.3	EPDS	12	12-48 weeks	8	102	1666
Lee[156]	Taiwan	2011	2003-2004	Cross-sectional	HI	32.38	BDI	13	5-8 weeks	9	15	60
Ahmed[157]	Iraq	2012	2010	Cross-sectional	HI		EPDS	10	6-8 weeks	9	284	1000
Giardinelli[158]	Italy	2012	2007-2008	Cross-sectional	HI	34	EPDS/SCID	10	12 weeks	9	78	590
Chien[159]	Taiwan	2012	2007-2008	Cross-sectional	HI		EPDS	10	2 years	9	94	380
Haga[160]	Norway	2012	2008-2009	Cross-sectional	Online		EPDS	10	6 weeks	9	52	344
Nordeng[161]	Norway	2012	2008-2010	Cross-sectional	HI	31	EPDS	13	8 weeks	8	161	1984
Husain[162]	UK	2012	2011	Cohort	HI		EPDS	12	24 weeks	9	63	237
Demissie[163]	USA	2013	2001-2005	Cohort	HI	29.4	EPDS	13	12 weeks	9	43	652
Chen[164]	Taiwan	2013	2008-2009	Cohort	HI	27	EPDS	10	4 weeks	8	49	203
									24 weeks		25	203
									4-24 weeks		37	203
Dolbier[165]	USA	2013	2012	Cross-sectional	HI		EPDS	10/13	4-24 weeks	8	38	299
								10	4 weeks		48	299

									24 weeks		52	299
								13	4 weeks		24	299
									24 weeks		27	299
Mariam[166]	India	2009	2008	Prospective	HI		EPDS	12	6-8 weeks	9	39	132
Sara[167]	Sweden	2013	2006-2007	Cohort	HI	30.8	EPDS	12	6-24 weeks	9	239	2318
									6 weeks		257	2318
									24 weeks		220	2318
Lucero[168]	USA	2012	2009-2010	Cohort	HI	26.6	PDSS	60	12-42 weeks	9	40	62
Serhan[169]	Turkey	2012	2011	Cross-sectional	HI	28.61	EPDS	12/13	8-24 weeks	9	10	110
Petrozzi[170]	Italy	2013	2005-2006	Cohort	HI	32.3	EPDS	9	12 weeks	8	45	594
Alexandre[171]	Brazil	2012	2005-2006	Cohort	HI	25	SRQ-20	7/8	24-72 weeks	9	219	701
Wisner[172]	USA	2013	2012	Cross-sectional	HI	29.6	EPDS	10	4-6 weeks	9	1396	9998
Abbasi[173]	USA	2013	2009-2011	Cohort	HI		EPDS	12	4 weeks	8	151	2972
Mercier[174]	USA	2013	2001-2005	Cohort	HI	29.4	EPDS	13	12-48 weeks	9	42	619
									12 weeks		50	688
									48 weeks		33	550
Burgut[175]	Qatar	2013	2010-2011	Cross-sectional	HI	33.5	EPDS	12	24 weeks	9	243	1379
Markhus[176]	Norway	2013	2009-2011	Cohort	Community		EPDS	10	12 weeks	8	3	43
Koutra[177]	Greece	2014	2007-2013	Cohort	HI	29.77	EPDS	13	8 weeks	9	57	438
Sidebottom[178]	USA	2014	2005-2009	Cross-sectional	Community	21.9	PHQ-9	10	4-12 weeks	9	83	594

Sadat[179]	Iran	2014	2007-2008	Prospective	HI		EPDS	13	8-16 weeks	9	57	300
									8 weeks		67	300
									16 weeks		47	300
Dudek[180]	Poland	2014	2010-2012	Cross-sectional	HI		EPDS	13	6-12 weeks	9	55	344
Goshtasebi[181]	Iran	2013	2009	Longitudinal	HI	26.6	EPDS	13	4-6 weeks	9	14	254
Swapan[182]	India	2013	2012	Cross-sectional	HI	24.62	PRIME MD		6 weeks	8	32	202
Alharbi[183]	Saudi Arabia	2014	2013	Retrospective	HI	29.92	EPDS	10	8-12 weeks	7	117	352
Töreki[184]	Hungary	2014	2010-2011	Prospective	HI	30.5	EPDS	12/13	6 weeks	9	44	266
Samira[185]	Israel	2015	2008-2009	Cross-sectional	HI	28	EPDS	10	4-28 weeks	9	175	564
Abdollahi[186]	Iran	2014	2009	Cohort	HI		EPDS	12	8 weeks	9	403	2083
Tsao[187]	Taiwan	2015	2008	Cohort	HI		EPDS	13	6 weeks	8	39	162
El-Hachem[188]	Lebanon	2014	2012-2013	Longitudinal	HI	31.7	EPDS	9	4-6 weeks	8	19	149
Nishigori[189]	Japan	2014	2011	Cross-sectional	HI		EPDS	9	24-44 weeks	9	135	633
Iliadis[190]	Sweden	2015	2009-2010	Longitudinal	HI		EPDS	12	6 weeks	9	83	975
Abdollahi[191]	Malaysia	2015	2009	Cohort	HI		EPDS	12	12 weeks	9	414	2279
Park[192]	Korea	2015	2010-2011	Longitudinal	HI	31	EPDS	10	4 weeks	8	65	153
Brown[193]	UK	2016	2012-2013	Cross-sectional	Community /Online	32.09	EPDS	12	24 weeks	9	32	217
Robakis[194]	USA	2016	2011-2014	Cross-sectional	HI/Commu nity		EPDS	10/12	8 weeks	8	30	105
								10	8 weeks		35	105

								12	8 weeks		24	105
Abdollahi[195]	Iran	2016	2009	Cohort	HI	26.07	EPDS	12	12 weeks	8	362	1910
Corrigan[196]	USA	2015	2014	Cross-sectional	HI	29.89	EPDS	9	6 weeks	9	13	61
Lee[197]	Taiwan	2016	2003-2004	Cross-sectional	HI	32.38	BDI	13	5-8 weeks	9	15	60
Dennis[198]	Canada	2016	2006-2009	Cohort	HI	29.6	EPDS	9/12	16 weeks	7	85	1125
								9	16 weeks		117	1125
								12	16 weeks		53	1125
Kim[199]	USA	2017	2013	Cross-sectional	Community	28.4	EPDS	10	48 weeks	9	96	223
Astrid[200]	Norway	2017	2012-2013	Cross-sectional	HI	28.3	EPDS	10	12 months	9	3	39
Qandil[201]	UK	2016	2013-2015	Longitudinal	HI	26	EPDS	11	12 weeks	9	28	101
Nam[202]	Korea	2017	2004-2013	Cohort	HI		ICD-10		24 weeks		666	81447
Islam[203]	Bangladesh	2017	2015-2016	Cross-sectional	HI		EPDS	10	24 weeks	9	150	426
Souza[204]	Brazil	2017	2011	Cross-sectional	HI	28.8	EPDS	6	12 weeks	8	1926	10468
Dennis[205]	Canada	2018	2011-2014	Cohort	Community	31.6	EPDS	9/12	4-52 weeks	9	70	503
								9	4 weeks		138	565
									12 weeks		99	488
									52 weeks		82	457
								12	4 weeks		43	565
									12 weeks		33	488
									52 weeks		24	457

Sandraluz[206]	USA	2017	2016	Cohort	Community	29.5	EPDS	10	4-8 weeks	8	6	28
									4 weeks		5	28
									8 weeks		6	28
Parthbeen.B[207]	India	2020	2019	Prospective	HI	29	EPDS		6 week	9	8	110
Bodhare.T[208]	India	2015	2013	Cross-sectional	HI	23.2	PHQ-9		6-8 weeks	9	27	274
Hiroko.I[209]	Japan	2015	2012-2013	Prospective	HI	37.7	EPDS	9	4 weeks	9	103	479
Yuko.O[210]	Japan	2014	2002-2005	Prospective	HI	31.4	EPDS	9	1-4 months	8	21	154
Tsuruko.M[211]	Japan	2011	2007-2010	Cross-sectional	HI		EPDS	9	4 weeks	9	72	675
Zaidi[212]	India	2017	2015-2016	Longitudinal	HI		EPDS	13	6 weeks	9	19	149
Dennis[213]	Canada	2017	2016	Cohort	Community		EPDS	9	24 weeks	8	120	549
Yan[214]	China	2017	2015-2017	Cohort	HI	31.03	SDS	50	6-12 weeks	6	468	1592
Kim[215]	USA	2017	2016	Cross-sectional	Community	28.5	EPDS	10	48 weeks	9	95	223
Zhao[216]	China	2017	2014-2015	Longitudinal	HI	30.58	EPDS	9	6 weeks	9	79	167
Badr[217]	USA	2018	2014-2016	Longitudinal	HI	30.65	BDI	20	10-12 weeks	9	4	25
Adamu[218]	Ethiopia	2018	2017	Cross-sectional	HI	28	EPDS	13	6 weeks	8	144	618
Khalifa[219]	Sudan	2018	2013-2014	Cross-sectional	HI		EPDS	12	12-32 weeks	8	15	231
									12 weeks		22	238
									32 weeks		8	223
Aleksi[220]	Finland	2018	2017	Cohort	HI		EPDS	10	8 weeks	8	110	1066
Chandrasekaran[221]	Canada	2018	2017	Prospective observational	HI		EPDS	10	3-6 weeks	8	16	103

Yoshihiro.M[222]	Japan	2011	2001-2003	Prospective	Community	29.9	EPDS	10	3-4 months	8	106	771
Min.W[223]	China	2020	2019	Cross-sectional	Community	28	DASS-21	10	6 months	8	68	435
Yelland.J[224]	Australia	2010	2007	Cross-sectional	HI	31	DASS-21		6 months	8	743	4269
Deepthi.N[225]	India	2020	2018-2019	Cross-sectional	HI	26.4	EPDS	10	6 weeks	9	191	890
Nagpal.J[226]	India	2008	2008	Cross-sectional	Community	27	EPDS	10	6 months	9	102	172
Iyengar.K[227]	India	2012	2008-2009	Cross-sectional	Community		EPDS	10	6-48 weeks	8	60	353
									6-8 weeks		87	430
									12 months		32	275
Prost.A[228]	India	2012	2006-2008	Cross-sectional	Community	25.5	Kessler 10-item scale		6 weeks	9	669	5801
Khalil[229]	USA	2019	2018	Cross-sectional	HI	29	EPDS	10	48 weeks	9	29	115
Ertmann[230]	Denmark	2019	2015-2016	Cohort	HI		MDI	20	8 weeks	8	86	1312
Hassert[231]	Czech Republic	2018	2017	Cross-sectional	HI	30.3	EPDS	12	48 weeks	8	29	126
	Thailand				HI	25.82	EPDS	12	48 weeks		58	161
Hoge[232]	Belgium	2019	2016	Cohort	HI	31	Bromley postnatal depression questionnaire		1-12 months	9	17	71
Premji[233]	Canada	2019	2008-2011	Cohort	HI		EPDS	12	16-48 weeks	9	78	2339
Labrague[234]	Philippines	2019	2016	Cross-sectional	Community	23.98	EPDS	10	6 weeks	8	27	165
Gan[235]	China	2019	2013-2016	Cohort	HI		EPDS	10	6weeks	8	287	2546
Bhusal[236]	Nepal	2018	2014-2015	Cross-sectional	HI	22.75	EPDS	12	4-14 weeks	8	59	346

Hege.S[237]	India	2012	2012	Cross-sectional	Community		DSM-IV		6-14 weeks	9	22	150
Bo.H[238]	China	2020	2020	Cross-sectional	HI		PHQ9			8	359	1309
Abebe[239]	Ethiopia	2019	2018	Cross-sectional	HI	24.3	EPDS	13	4-24 weeks	8	113	511
Hamazaki[240]	Japan	2019	2011-2014	Cohort	Community /HI		EPDS	9	24 weeks	8	9761	84181
Maeda[241]	Japan	2019	2010-2013	Cohort	HI		EPDS	9	4 weeks	9	196	977
Elham[242]	Iran	2020	2019	Cohort	HI	28.87	EPDS	10	12-36 weeks	9	119	478
Valdes.V[243]	Bangladesh	2020	2020	Cross-sectional	Community	23.7	EPDS	9	6 months	8	57	235
Simhi[244]	Israel	2019	2014-2015	Cross-sectional	HI		EPDS	10	4-9 weeks	9	84	1000
Gausia.K[245]	Bangladesh	2007	2005	Cross-sectional	HI		DSM-IV		6 weeks	8	9	126
Chan[246]	Singapore	2020	2018-2019	Cohort	HI		EPDS	10	6-10 weeks	9	43	205
Wubetu[247]	Ethiopia	2020	2018	Cross-sectional	HI		EPDS	13	6 weeks	8	48	308
Nasreen.HE[248]	Bangladesh	2015	2008-2009	Prospective	HI		EPDS	12	2-8 months		92	495
Spinola.O[249]	Italy	2020	2020	Cross-sectional	Online	34	EPDS	12	1-12 months	9	107	243
Dawadi[250]	Nepal	2020	2017-2018	Cross-sectional	HI		EPDS	12	4-48 weeks	8	44	160
Pradhananga[251]	Nepal	2020	2019	Cross-sectional	HI	26	EPDS	12	6-10 weeks	9	51	348
Abdollahi.F[252]	Iran	2016	2009	Cross-sectional	HI		EPDS	12	3 months	9	248	1231
Tan[253]	Singapore	2020	2009-2015	Cohort	HI		EPDS	10	12 weeks	8	152	651
Yamada[254]	Japan	2020	2012	Cross-sectional	HI	31.4	EPDS	9	3-4 months	9	536	6159
Alikamali[255]	Iran	2020	2018	Cross-sectional	HI		EPDS	12	4-24 weeks	8	140	400
Boran[256]	Turkey	2020	2012-2018	Cross-sectional	HI		EPDS	12	1 month	9	210	1614

Bakare.M[257]	Nigeria	2014	2014	Cross-sectional	HI	28.2	EPDS	9	4 months	8	101	408
Beck.C[258]	USA	2011	2006	Cross-sectional	Online/Telephone		PHQ-2	3	12 months	9	379	903
McCoy.S[259]	USA	2006	2001-2003	Cross-sectional	HI		EPDS	13	4 weeks	8	81	209
Appolonio.K[260]	USA	2008	2008	Cross-sectional	HI		EPDS	12	6 months	8	17	87
Boury.J[261]	USA	2004	2004	Cross-sectional	HI	27	BDI		30 weeks	8	77	151
Mayberry.L[262]	USA	2007	2002	Cross-sectional	Online		EPDS	13	7-24 months	9	422	1359
									7-12 months		424	1359
									13-24 months		419	1359
Bugdayci.R[263]	Turkey	2004	2001	Cross-sectional	Home	27.5	EPDS	13	3 months-2 years	9	556	1447
									3-6 months		530	1447
									7-12 months		521	1447
									13+ months		618	1447
Chang.H[264]	Taiwan	2014	2010-2011	Cross-sectional	HI	34.3	CES-D		6-24 months	8	34	129
Miles.M[265]	USA	2007	2007	Longitudinal	HI		EPDS	16	6 months	9	64	103
Cheng.C[266]	USA	2009	2009	Cross-sectional	HI		CES-D		1 year	8	36	152
Verkerk.G[267]	Netherland	2005	2005	Longitudinal	HI	30.8	EPDS	11	1 year	8	50	277
Chien.L[268]	Taiwan	2009	2003	Cross-sectional	HI	31.6	CES-D		12-13 months	8	39	166
Lara.M[269]	Mexico	2014	2014	Cross-sectional	HI	29.5	DSM-IV		6 months	9	42	210
Leung.S[270]	Hong Kong	2005	2004	Cross-sectional	HI		EPDS	2	6 weeks	8	53	269

McMahanon.C[271]	Australia	2005	2004	Cross-sectional	HI		CES-D		6-12 months	9	51	114
									6 months		68	114
									12 months		34	114
Motzfeldt.I[272]	Denmark (Greenland)	2013	2011	Cross-sectional	Home	28	EPDS	13	3 months	9	15	174
Murray.L[273]	Vietnam	2015	2012	Cross-sectional	Community		EPDS	12/13	1-6 months	8	78	431
Lee.D[274]	Hong Kong	2004	2004	Cross-sectional	HI	29	EPDS	9	3 months	8	122	781
Necho.M[275]	Ethiopia	2020	2016-2017	Cross-sectional	HI	29.9	EPDS	13	4 weeks	9	102	378
Salem.M[276]	Egypt	2017	2015-2016	Cross-sectional	HI		EPDS	13	6 weeks	6	47	658
Akbari[277]	Iran	2020	2019	Descriptive	HI	26.23	EPDS	13	4-8 weeks	9	44	200
Zeng[278]	Singapore	2020	2015-2019	Cohort	HI		EPDS	10	5-9 weeks	9	54	518
Mokwena[279]	South Africa	2020	2019	Cross-sectional	HI	28	EPDS	13	4-48 weeks	9	232	406
Karl[280]	Germany	2020	2019	Cohort	HI	30.1	EPDS	10	8 weeks	8	83	587
Chalise[281]	Nepal	2020	2019	Cross-sectional	HI	23.8	EPDS	12	24 weeks	8	41	242
Cena[282]	Italy	2021	2017-2018	Cross-sectional	HI		EPDS	12	36 weeks	8	60	302
Costa[283]	Canada	2006	2005	Cross-sectional	Community	33.17	EPDS	12	4-38 weeks	8	49	78
Dennis[284]	Canada	2006	2001-2002	Cohort	Community	29	EPDS	9	8 weeks	8	56	396
Dennis[285]	Canada	2007	2001-2002	Cohort	Community	28.5	EPDS	12	8 weeks	9	38	475
Kim.T[286]	Canada	2014	2005-2006	Cross-sectional	HI		EPDS	13	5-9 months	9	468	6304
Dennis.C[287]	Canada	2014	2005-2006	Cross-sectional	HI		EPDS	13	5-14 months	8	513	6421
Logsdon.M[288]	USA	2005	2005	Cross-sectional	HI		CES-D		6 weeks	9	72	156

Chatzi[289]	Greece	2011	2007-2010	Cohort	Community		EPDS	13	8-10 weeks	9	74	529
Sword[290]	Canada	2011	2010	Cohort	HI		EPDS	12	6 weeks	8	195	2560
Edhborg[291]	Bangladesh	2011	2007-2008	Cohort	Community		EPDS	10	8-12 weeks	8	95	671
Dallal.F[292]	Bahrain	2012	2008	Cross-sectional	HI		EPDS	12	8 weeks	8	88	237
Eastwood[293]	Australia	2011	2000-2004	Cross-sectional	Community /HI		EPDS	9/12	12 weeks	8	2317	25455
								9	12 weeks		305	25455
								12	12 weeks		1578	25455
Buist.A[294]	Australia	2008	2002-2004	Cross-sectional	HI	30.3	EPDS	12	6-8weeks	7	920	12266
Abdelwahid.H[295]	Egypt	2012	2012	Cross-sectional	Community		EPDS	13	6-8 weeks	8	44	200
Melo Jr[296]	Brazil	2012	2011	Cross-sectional	HI		EPDS	12	4-6 weeks	9	60	555
Bener.A[297]	Qatar	2012	2010-2011	Cross-sectional	HI	32.2	DASS-21		6 months	9	309	1659
Alasoom.L[298]	Saudi Arabia	2014	2014	Cross-sectional	HI	27.8	EPDS	13	2-6 months	9	80	450
Chalise.A[299]	Nepal	2019	2017	Cross-sectional	HI	25.8	EPDS	12	6 months	9	37	195
Khadka.R[300]	Nepal	2020	2017	Cross-sectional	HI	30	PHQ-2	3	2-12 months	9	71	380
Bilszta.J[301]	Australia	2008	2008	Cross-sectional	HI		EPDS	13	4-6 weeks	8	149	1966
Boyce.P[302]	Australia	2005	2005	Cross-sectional	HI	26.9	EPDS	12	6-24 weeks	8	39	425
									6-12 weeks		38	425
									18 weeks		43	425
									24 weeks		37	425
Eckerdal.P[303]	Sweden	2020	2009-2017	Cross-sectional	HI	29	EPDS	12	6 weeks	8	193	1503

Silva[304]	Brazil	2012	2006-2008	Cohort	HI	25.2	EPDS	13	30-60 days	8	168	1019
Malta[305]	Canada	2012	2008	Cohort	Community		EPDS	10	16 weeks	8	177	1347
Brooks.J[306]	Australia	2009	2002-2004	Cross-sectional	HI		EPDS	13	10-42 weeks	9	231	3853
Leigh.B[307]	Australia	2007	2007	Cross-sectional	HI		EPDS	13	10-12 weeks	8	18	161
Wynter.K[308]	Australia	2013	2006-2007	Cross-sectional	HI		EPDS	10	4 weeks	9	21	172
Filha.M[309]	Brazil	2016	2011-2012	Cross-sectional	Telephone	25.6	EPDS	13	6-18 months	9	3293	12764
Matijasevich[310]	Brazil	2009	2004	Cross-sectional	Population		EPDS	13	2-24 months	9	582	4109
									2-3 months		468	4190
									8-12 months		62	4190
									12-24 months		65	4190
Moris.M[311]	Brazil	2013	2006-2008	Cross-sectional	HI		EPDS	12	16-52 weeks	8	17	87
Pinheiro.R[312]	Brazil	2013	2008	Cross-sectional	Population	24.5	EPDS	13	6-12 weeks	8	42	207
Dennis.C[313]	Canada	2003	2001-2002	Longitudinal	HI	28.5	EPDS	10	8 weeks	8	103	497
Dennis.C[314]	Canada	2009	2004-2006	Longitudinal	HI		EPDS	13	12 weeks	9	22	315
McDonald.S[315]	Canada	2014	2014	Cross-sectional	Population		EPDS	10	16 weeks	8	515	1280
Mao.Q[316]	China	2011	2007-2008	Cross-sectional	HI	26.6	EPDS	13	6-8 weeks	9	56	376
Paulson.J[317]	USA	2006	2001	Cross-sectional	HI		CES-D	15	9 months	9	733	5089
Drozd[318]	Norway	2018	2011-2012	Cohort	HI		EPDS	10	6-48 weeks	9	54	980
									6 weeks		92	1260
									16 weeks		63	1130

									24 weeks		45	1031
									48 weeks		16	499
Gao.L[319]	China	2010	2008	Cross-sectional	HI	28.1	EPDS	13	6-8 weeks	8	18	126
Leung.W[320]	China	2002	2000	Prospective	HI		EPDS	10	6 weeks	9	50	694
Chen.L[321]	China	2019	2017-2018	Retrospective	HI		EPDS	10	6 weeks	8	35	180
Tashakori[322]	Iran	2012	2009	Cohort	HI		EPDS	13	24 weeks	8	16	150
January[323]	Zimbabwe	2015	2014	Cross-sectional	HI	25.4	EPDS	12	48 weeks	8	101	295
Tychey.C[324]	France	2005	2005	Cross-sectional	HI		EPDS	12	4-8 weeks	9	31	277
Zejnullahu.V[325]	Serbia	2021	2019	Cross-sectional	HI		EPDS	12	6 weeks	9	52	247
Reck.C[326]	Germany	2008	2003-2005	Prospective	HI	33	DSM-IV		12 weeks	9	62	1024
Giakoumaki.O[327]	Greece	2009	2009	Cross-sectional	HI/Telephone		EPDS	14	12 weeks	8	11	235
Stone[328]	USA	2015	2007-2010	Surveys	System data		PHQ-2		8-24 weeks	8	804	5395
Lynch[329]	USA	2014	2009-2010	Cross-sectional	National data		PRAMS		8-16 weeks	8	5365	40337
Pooler[330]	USA	2013	2006-2008	Surveys	System data		PHQ-2		8-24 weeks	9	10382	75234
Schachman[331]	USA	2013	2012	Comparative descriptive	HI		EPDS	14	8 weeks	8	36	71
Sweeney[332]	USA	2013	2012	Cohort	HI		EPDS	12	8 weeks	7	5	46
Dagher[333]	USA	2012	2005-2008	Cohort	Community		EPDS	12	8 weeks	8	34	526
Gress-Smith[334]	USA	2012	2011	Longitudinal	HI	26.5	CES-D	16	20-36 weeks	8	47	132

									20 weeks		44	132
									36 weeks		50	132
Kornfeld[335]	USA	2012	2008	Retrospective	HI		US Preventive Services Task Force		8-24 weeks	9	8	36
									8 weeks		6	39
									16 weeks		11	38
									24 weeks		8	31
Gjerdingen[336]	USA	2011	2005-2006	Cohort	HI	29.1	PHQ-9	10	8-36 weeks	9	34	463
									8 weeks		33	464
									16 weeks		32	459
									24 weeks		23	455
									36 weeks		48	472
Murphy[337]	USA	2010	2008	Cohort	HI		EPDS	10	4-6 weeks	9	12	97
Le[338]	USA	2010	2009	Longitudinal	HI		PDSS	60	6-8 weeks	8	141	220
Sorenson[339]	USA	2010	2009	Exploratory	Daily newspaper		BDI		24-28 weeks	8	11	71
McGrath[340]	USA	2008	2007	Longitudinal	HI	27	EPDS	12	8-24 weeks	9	17	128
									8 weeks		22	147
									24 weeks		12	109
Pinheiro[341]	Brazil	2011	2007	Cohort	HI	26.2	EPDS	10	9-48 weeks	9	90	382
									9-12 weeks		90	397

									48 weeks		90	366
Abiodun[342]	Nigeria	2006	2005	Cross-sectional	HI	27.9	EPDS	9	6 weeks	9	67	360
Pantbangi.V[343]	USA	2009	2004-2006	Cross-sectional	HI	24.9	EPDS	12	5-8 weeks	8	94	530
Yawn.B[344]	USA	2009	2008	Cross-sectional	HI		EPDS/PHQ-9	10	5-8 weeks	8	73	481
Shafiei.T[345]	Australia	2015	2006-2007	Cross-sectional	HI	29	EPDS	13	4 months	9	12	39
Abbasi.M[346]	Iran	2014	2010-2011	Cross-sectional	HI	29	EPDS	13	12 weeks	9	142	416
Kheirabadi.G[347]	Iran	2010	2007-2008	Prospective	HI		EPDS	12	6-8 weeks	8	259	1291
Kheirabadi.G[348]	Iran	2009	2008	Cross-sectional	Population	26	EPDS	10	2-12 months	8	3784	6627
Abadiga.M[349]	Ethiopia	2019	2019	Cross-sectional	HI	29.6	EPDS	10	1-12months	9	60	287
Upadhyay.A[350]	Ethiopia	2019	2002	Cross-sectional	Population		SRQ		5-21 months	9	652	1811
	India	2019	2002	Cross-sectional	Population		SRQ		5-21 months		540	1800
	Peru	2019	2002	Cross-sectional	Population		SRQ		5-21 months		597	1992
	Vietnam	2019	2002	Cross-sectional	Population		SRQ		5-21 months		385	1835
Azale.T[351]	Ethiopia	2018	2014	Cross-sectional	Population	27.9	PHQ-9	10	1-12 months	8	385	3147
Tsai.A[352]	South America	2016	2009-2014	Longitudinal	Population		EPDS	13	6-36 months	9	489	1238
Hung.K[353]	South America	2014	2010-2011	Prospective	Community	26	EPDS	13	3 months	8	79	249
Baumgartner.J[354]	Ethiopia	2014	2014	Cross-sectional	Community		SRQ-20		1-24 moths	8	433	1319
Mauri.M[355]	Italy	2010	2008	Cross-sectional	HI		EPDS	13	1-12 months	9	78	751
Maher.A[356]	Japan	2004	2000	Cross-sectional	Community		EPDS	9	3-4 months	9	146	1048

Kim.J[357]	Korea	2008	2003-2007	Cross-sectional	HI		EPDS	12	6 weeks	9	30	239
Dow.A[358]	Malawi	2013	2008-2012	Cross-sectional	Community		EPDS	13	10-14 weeks	8	12	154
Underwood.L[359]	New Zealand	2016	2009-2010	Cross-sectional	Community		EPDS	12	9 months	8	265	5301
Adewuya.A[360]	Nigeria	2006	2005	Cross-sectional	HI		EPDS	13	12 months	9	15	86
Dorheim.S[361]	Norway	2014	2008-2010	Longitudinal	HI	31.5	EPDS	10	8 weeks	9	499	2088
Modayfer.O[362]	Saudi Arabia	2015	2008	Cross-sectional	HI		EPDS	13	5 weeks	9	78	571
Heh.S[363]	Taiwan	2008	2008	Cross-sectional	HI		EPDS	10	4 weeks	8	92	400
Akman.I[364]	Turkey	2008	2005	Cross-sectional	HI		EPDS	13	4 months	9	8	60
Leahy-Warren[365]	Ireland	2011	2008	Longitudinal	Community		EPDS	12	6-12 weeks	8	45	389
									6 weeks		54	410
									12 weeks		36	367
Goecke[366]	Germany	2012	2011	Prospective	HI	29.9	EPDS	9/12	4-72 weeks	7	16	149
Meijer[367]	Netherlands	2014	2010-2012	Cohort	HI		EPDS	10	16-28 weeks	9	137	1620
Meltzer-Brody[368]	Netherlands	2013	2004-2007	Cohort	Community /HI		EPDS	12	12 weeks	9	276	679
Dmitrovic[369]	Serbia	2014	2011-2012	Cross-sectional	HI		EPDS	12	6-8 weeks	9	23	195
Kerstis[370]	Sweden	2015	2004-2006	Cohort	HI		EPDS	10	12 weeks	8	50	305
Grote[371]	Belgium; Germany; Italy; Poland; Spain	2010	2002-2004	Cohort			EPDS	13	96 weeks	8	102	929
McMahon[372]	Australia	2015	2014	Cohort	HI	34.1	M.I.N.I		12 weeks	9	36	434

Woolhouse[373]	Australia	2014	2003-2005	Cohort	HI		EPDS	13	24-48 weeks	8	174	1305
Petrosyan[374]	Armenia	2011	2010	Case-control	HI		EPDS	12	4-12 weeks	8	63	437
Wubetu[375]	China	2014	2012-2013	Longitudinal	HI	26.5	EPDS	11	12 weeks	9	21	223
Ngai[376]	Hong Kong	2015	2011	Longitudinal	HI	32.3	GHQ	5	24 weeks	8	23	200
Lau[377]	Hong Kong	2010	2004-2006	Longitudinal	HI		EPDS	9/14	6 weeks	9	123	610
								9			193	610
								14			5	610
Taherifard[378]	Iran	2013	2011	Cross-sectional	HI	27.9	EPDS	13	6-8 weeks	9	62	179
Glasser[379]	Israel	2012	2009	Cohort	HI		EPDS	10	6 weeks	9	379	2326
Pollock[380]	Mongolia	2009	2002	Cross-sectional	HI		SRQ-20	8.5	5-9 weeks	9	95	1044
Husain[381]	Pakistan	2011	2004-2005	Cohort	HI	31.7	EPDS	12	12 weeks	9	292	763
Cankorur[382]	Turkey	2015	2007-2008	Cohort	HI		EPDS	13	8-24 weeks		151	578
Annagur[383]	Turkey	2012	2011	Prospective	HI	28.58	EPDS	12	6 weeks	8	28	197
POÇAN[384]	Turkey	2013	2008	Cross-sectional	HI	29.27	EPDS	12	4-6 weeks	9	54	187
Akyuz[385]	Turkey	2010	2008	Cohort	HI		PDSS	65	4-6 weeks	8	31	156
Beydoun[386]	Canada	2010	2006	Cross-sectional	System data		EPDS	13	20-36 weeks	9	482	6421
Ward[387]	USA	2017	2004-2011	Cohort	System data/HI		PRAMS		8-24 weeks	9	1535	10231
Daoud[388]	Canada	2019	2006	Cross-sectional	System data		EPDS	10/13	20-56 weeks	9	8617	74231
								10			11774	74231

								13			5459	74231
Miyake[389]	Japan	2020	2007-2008	Cohort	HI	31.3	EPDS	9	12-16 weeks	6	108	1316
Miller[390]	Australia	2006	2005	Cross-sectional	Community	32	EPDS	9	6-24 weeks	8	80	325
Sutter-Dallay[391]	France	2004	2003	Cohort	HI	29.6	EPDS	12	6 weeks	9	29	497
Edvinsson[392]	Sweden	2017	2010-2013	Cohort	Online	32	EPDS	13	6-14 weeks	9	33	157
Aydin.N[393]	Turkey	2004	2001	Cross-sectional	HI	26.6	EPDS	13	1-12months	8	122	341
Ege.E[394]	Turkey	2008	2004	Cross-sectional	Community	26.5	EPDS	13	6-48 weeks	9	121	364
Ekuklu.G[395]	Turkey	2004	2002	Cross-sectional	In home	21.5	EPDS	12	6 weeks	9	72	178
Mishra.K[396]	India	2020	2017-2018	Cross-sectional	HI	24	EPDS	13	4-6 weeks	9	5	60
Kaya.L[397]	Turkey	2019	2014	Cross-sectional	HI	23.3	EPDS	12	1-3 months	9	57	264
									1 month		100	264
									3 months		13	264
Liu.S[398]	China	2017	2015	Cross-sectional	HI		EPDS	10	4 weeks	8	59	882
Sylven.S[399]	Sweden	2017	2006-2007	Cross-sectional	HI		EPDS	12	6 weeks	9	42	653
Cirik.D[400]	Turkey	2016	2013-2014	Cross-sectional	HI		EPDS	13	2-6 months	8	35	149
Jenny.S[401]	USA	2013	2004-2006	Cross-sectional	Community	31	EPDS	9	8 weeks	8	60	587
Nicklas.J[402]	USA	2013	2010-2012	Cross-sectional	HI	33	EPDS	9	4-15 weeks	8	24	71
Eisenach.J[403]	USA	2008	2004-2005	Prospective	HI		EPDS	12	8 weeks	8	144	1288
Gulseren.L[404]	Turkey	2006	2002	Prospective	Community	26.5	EPDS	10	5-26 weeks	9	17	135
									5-8 weeks		21	135

									10-14 weeks		18	135
									20-26 weeks		12	135
Meijer.J[405]	Netherlands	2019	2010-2015	Cross-sectional	HI		EPDS	10	4-7 months	9	92	2003
Edge.D[406]	UK	2004	2000-2001	Cross-sectional	HI		EPDS	12	6 weeks	8	96	301
Morrell.C[407]	UK	2009	2003-2006	Cross-sectional	Community		EPDS	12	6 weeks	9	595	4084
Nakano.M[408]	Japan	2020	2013-2015	Longitudinal	Population	30	EPDS	9	3 moths	8	93	1050
Oladeji.B[409]	Nigeria	2019	2013-2015	Cross-sectional	HI		EPDS	12	1-6 months	8	590	8580
Certain.H[410]	USA	2008	2002-2005	Cross-sectional	HI		EPDS	12	6 weeks	7	153	1519
Zee-van der berg.A[411]	Netherlands	2017	2012-2014	Cross-sectional	HI		EPDS	12	9 months	9	203	2474
Mishina.H[412]	Japan	2009	2007	Cross-sectional	HI	31.5	EPDS	9	1 month	9	17	103
Dagher.R[413]	USA	2003	2001	Prospective	Community		EPDS	12.5	11 weeks	9	30	638
Shakeel.N[414]	Norway	2018	2008-2011	Cross-sectional	HI		EPDS	10	3 months	9	60	643
Gaffney.K[415]	USA	2014	2005-2007	Cross-sectional	HI		EPDS	10	2 months	8	349	1447
Glynn.L[416]	USA	2014	2002-2007	Cross-sectional	HI		EPDS	10	3 months	9	34	170
Elizabeth.A[417]	USA	2012	2009-2010	Longitudinal	HI		EPDS	13	12-24 weeks	8	34	242
Cury.A[418]	Brazil	2016	2005-2006	Prospective	HI		SRQ-20	7/8	11 months	9	196	701
Knight.J[419]	USA	2016	2013	Retrospective	HI		EPDS	14	2-6 weeks	8	8	191
Velloza.J[420]	Kenya	2020	2017-2018	Cross-sectional	HI		PHQ-9	5	1-3 months	8	12	20
Avilla.J[421]	Brazil	2020	2016	Cross-sectional	HI		EPDS	11	1 month	8	36	287
Rawahi.A[422]	Oman	2020	2014	Prospective	HI	27	EPDS	13	6-8 weeks	9	126	592

Mott.S[423]	USA	2011	2008-2009	Cross-sectional	HI		EPDS	13	52 weeks	9	11	147
Silverman.M[424]	USA	2010	2007	Cross-sectional	HI		EPDS	9	6 weeks	8	94	439
Watkin.S[425]	USA	2011	2005-2007	Cross-sectional	HI	28.8	EPDS	13	8 weeks	8	222	2586
Sun.L[426]	China	2020	2012-2016	Cross-sectional	Community		EPDS	10	11-14 months	9	95	347
Riazanova.O[427]	Russia	2018	2015-2017	Cross-sectional	HI	30	EPDS	10	6 weeks	8	10	210
Lupattelli.A[428]	Italy	2018	2011-2012	Cross-sectional	Online		EPDS	10	1 year	9	90	283
	UK	2018	2011-2012	Cross-sectional	Online		EPDS	10	1 year		209	676
	Switzerland	2018	2011-2012	Cross-sectional	Online		EPDS	10	1 year		59	269
	France	2018	2011-2012	Cross-sectional	Online		EPDS	10	1 year		51	118
	Norway	2018	2011-2012	Cross-sectional	Online		EPDS	10	1 year		114	544
	Sweden	2018	2011-2012	Cross-sectional	Online		EPDS	10	1 year		104	416
	Finland	2018	2011-2012	Cross-sectional	Online		EPDS	10	1 year		47	199
	Russia	2018	2011-2012	Cross-sectional	Online		EPDS	10	1 year		195	610
	Poland	2018	2011-2012	Cross-sectional	Online		EPDS	10	1 year		138	300
	Croatia	2018	2011-2012	Cross-sectional	Online		EPDS	10	1 year		77	171
	Slovenia	2018	2011-2012	Cross-sectional	Online		EPDS	10	1 year		14	44
	Serbia	2018	2011-2012	Cross-sectional	Online		EPDS	10	1 year		59	131
Wesselhoeft.R[429]	Denmark	2020	2010-2012	Cross-sectional	Community		EPDS	12	2.5 months	8	133	2069
	Vietnam	2020	2014-2015	Cross-sectional	Community		EPDS	12	6 weeks		24	1278
	Tanzania	2020	2014-2015	Cross-sectional	Community		EPDS	12	6 weeks		147	1169

Nhi.T[430]	Vietnam	2019	2014-2015	Cross-sectional	Community	26	EPDS	9/10	4-12 weeks	9	104	1274
Do.T[431]	Vietnam	2018	2018	Cross-sectional	Community		EPDS	12	1 year	9	32	116
Fellmeth.G[432]	Thailand	2020	2015-2016	Cross-sectional	HI		DSM-IV		1 month	8	78	396
Ing.H[433]	Thailand	2017	2014-2015	Cross-sectional	Population		SCID		4-16 weeks	8	14	670
Roomruangwong.C[434]	Thailand	2016	2010	Case-control	HI		EPDS	11	4-6 weeks	8	53	313
Panyayong.B[435]	Thailand	2013	2009	Cross-sectional	Population		EPDS	13	6-8 weeks	6	145	1731
Tomlinson.M[436]	Afghanistan	2020	2019	Cross-sectional	HI	26	PHQ-9	12	1-12 months	9	131	215
Takacs.L[437]	Czech republic	2019	2013-2014	Cross-sectional	HI		EPDS	12	9 months	9	30	282
Coo.S[438]	Chile	2020	2018-2019	Cross-sectional	HI		EPDS	10	3-6 months	9	48	229
Amemiya[439]	Japan	2016	2012	Cohort	HI		EPDS	9	16 weeks	8	614	6461
Kabir[440]	Bangladesh	2014	2008-2009	Cross-sectional	Community	25	EPDS	9/10	24-32 weeks	8	192	600
Miura[441]	Japan	2017	2012	Cross-sectional	HI	31.37	EPDS	9	12-16 weeks	9	955	6534
Tran[442]	Vietnam	2018	2014-2015	Cohort	Community		EPDS	10	4-12 weeks	8	104	1274
Silove[443]	Timor-Leste	2015	2012-2013	Cross-sectional	Community /Records		EPDS	13	12-24 weeks	8	43	170
Faisal-Cury[444]	Brazil	2013	2006-2007	Cross-sectional	HI		SRQ-20	8	12 months	9	196	701
Lobato[445]	Brazil	2012	2007	Cohort	HI		EPDS	12	20 weeks	7	197	811
Sunnqvist[446]	Sweden	2019	2012-2015	Cohort	HI		EPDS	13	72 weeks	8	175	1747
Wei[447]	USA	2008	2002-2005	Cohort	Community	23.6	PDSS	60	6 weeks	8	147	586
Silva[448]	Brazil	2017	2010	Cross-sectional			EPDS	12	2-12 weeks	9	267	2259

Sha[449]	China	2019	2015-2016	Cohort	Community		EPDS	10	4 weeks	9	56	956
Gregory[450]	USA	2014	2005-2007	Cohort	HI		EPDS	10	8 weeks	8	346	1501
Woolhouse[451]	Australia	2016	2003-2005	Cohort	HI		EPDS	13	12 weeks	8	82	1258
Woolhouse[452]	Australia	2014	2003-2005	Cohort	HI		EPDS	13	4 years	9	219	1507
Woolhouse[453]	Australia	2012	2003-2005	Cohort	HI		EPDS	13	48 weeks	8	241	1507
Nakamura[454]	Japan	2020	2004-2006	Cross-sectional		32.4	EPDS	9	4 weeks	8	138	1033
Iwata[455]	Japan	2016	2012-2013	Cohort	HI	33	EPDS	9	24 weeks	8	241	2709
Takehara[456]	Japan	2017	2012-2013	Longitudinal	HI		EPDS	8/9	12 weeks	9	83	1306
Iranpour[457]	Iran	2016	2012	Cross-sectional	HI		EPDS	13	12 weeks	8	125	359
Emerson[458]	USA	2014	2011-2012	Prospective observational	HI	27	EPDS	10	16 weeks	9	31	200
Birmingham[459]	USA	2011	2007-2008	Cross-sectional	HI		EPDS	10	24 weeks	9	45	195
Gong[460]	China	2020	2016-2017	Cohort	HI		EPDS	13	6 weeks	9	248	1121
Kothari[461]	USA	2016	2009	Cross-sectional	Community		EPDS	12	8 weeks	7	30	301
Li[462]	China	2020	2017-2018	Cross-sectional	HI	30.6	EPDS	9	8-16 weeks	9	90	522
Liu[463]	China	2020	2019	Cross-sectional	HI		EPDS	13	24 weeks	9	279	1204
Cao[464]	China	2019	2015-2017	Cohort	HI		SDS	50	6-12 weeks	8	569	1659
Chen[465]	China	2018	2016	Cross-sectional	HI	28.32	EPDS	13	6 weeks	8	184	1263
Liang[466]	China	2020	2020	Cross-sectional	HI		EPDS	10	6-12 weeks	9	253	845
Ding[467]	China	2019	2013-2016	Cohort	Population	28.7	EPDS	10	6 weeks	6	308	2615
Lu[468]	China	2020	2017-2018	Prospective	Community	29.08	EPDS	13	48 weeks	9	387	3113

					/HI							
Jiang[469]	China	2018	2011-2015	Cross-sectional	HI		SDS	53	6 weeks	8	169	924
Shi[470]	China	2021	2017-2019	Cross-sectional	HI	28.6	EPDS	10	6 weeks	8	58	397
Zheng[471]	China	2018	2013	Longitudinal	HI		EPDS	13	6 weeks	9	65	304
									12 weeks		39	214
									6-12 weeks		52	259
Wan[472]	China	2009	2006	Prospective	HI	30	EPDS	13	6-8 weeks	9	53	342
Luo[473]	China	2020	2014	Cohort	HI		EPDS	10	6 weeks	8	68	363
Quan[474]	China	2020	2015-2017	Cohort	HI		EPDS	13	6 weeks	8	26	360
Fu[475]	China	2014	2013	Cohort	HI	31	EPDS	12	12 weeks	9	26	213
Lin[476]	Taiwan	2019	2003-2006	Prospective longitudinal	HI	32	EPDS BDI		4 weeks	8	12	234
Liu[477]	China	2016	2014	Cohort	HI	30.5	EPDS	12	24 weeks	7	45	296
Deng[478]	China	2021	2014-2015	Cohort	HI		EPDS	10	6 weeks	9	100	577
Gao[479]	China	2016	2014	Cohort	HI		EPDS	12	12 weeks	9	37	340
Liu[480]	China	2019	2014-2017	Prospective longitudinal	HI	30	EPDS	10	6 weeks-2 years	9	60	598
Sun[481]	China	2020	2017-2018	Cohort	HI		EPDS	10	6 weeks	8	57	417
Ding[482]	China	2014	2009	Cohort	HI	29	EPDS	10	6 weeks	7	52	214
Pham[483]	Argentina	2018	2016	Cohort	HI		EPDS	10/13	4 weeks	8	133	539
								10			167	539

								13			99	539
Fraga[484]	Brazil	2020	2011-2012	Prospective	HI	25.7	EPDS	13	24-72 weeks	8	6291	23894
Roomruangwong[485]	Thailand	2016	2014	Prospective	HI		EPDS	11	4-6 weeks		6	113
Faisal-Cury[486]	Brazil	2020	2013-2015	Prospective	HI		PHQ-9	10	24-60 weeks	9	133	315
									24-32 weeks		145	346
									48-60 weeks		120	283
Silveira[487]	Brazil	2019	2015	Cohort	Population		EPDS	13/15	12 weeks	8	231	3065
Costa[488]	Brazil	2017	2012-2014	Cohort	HI	20.1	BDI	12	4-8 weeks	9	204	527
Faisal-Cury[489]	USA	2019	2011	Cohort	Community		PHQ-9	10	24-60 weeks	7	133	315
Brito[490]	Brazil	2015	2005-2006	Cohort	Community		EPDS	11/12	32 weeks	8	274	1056
Ivan[491]	Brazil	2018	2017	Cross-sectional	HI		EPDS	10	4-8 weeks	9	30	151
Callo-Quinte[492]	Brazil	2019	2004	Cohort	Population		EPDS	10	48 weeks	9	1059	3838
Zaconeta[493]	Brazil	2013	2011	Cohort	HI		EPDS	13	4-8 weeks	8	12	107
Tannous[494]	Brazil	2008	2001	Cross-sectional	Community		EPDS	13	6-8 weeks	8	56	271
Faisal-Cury[495]	Brazil	2020	2011	Prospective	HI		PHQ-9	10	48-60 weeks	9	56	294
Pinheiro[496]	Brazil	2006	2004	Cross-sectional	Population		BDI	9	6-12 weeks	9	91	386
Valdes[497]	Chile	2017	2013	Cohort	HI		EPDS	12	12-48 weeks	9	46	223
Bauman[498]	USA	2020	2012-2018	Prospective	System data		The Patient Health Questionnaire-2		8-24 weeks	9	4311	32659
Wissart[499]	Jamaica	2005	2000-2001	Prospective	HI	27	SDS	50	6 weeks	8	25	73

Youn[500]	Korea	2017	2010-2012	Cross-sectional	Database		ICD-10		48 weeks	9	17483	1269130
Choi[501]	Korea	2014	2009-2010	Cohort	HI		EPDS	10	8 weeks	9	56	192
Kim[502]	Korea	2016	2006-2007	Prospective	Community		DSM-IV		4 weeks	7	95	679
Suhitharan[503]	Singapore	2016	2010-2013	Case-control	HI	31.2	EPDS	7	4-8 weeks	9	62	479
Alves[504]	Portugal	2018	2015-2016	Cross-sectional	Community	32.75	EPDS	9/12	48 weeks	9	43	204
								9			56	204
								12			29	204
Alfonso[505]	Spain	2019	2008	Prospective longitudinal	HI		EPDS	9	8-32 weeks	7	42	525
Ikeda[506]	Japan	2013	2009-2010	Cohort	HI	33.4	PDPI-R-J	7.5	4 weeks	9	16	76
Fellmeth[507]	Burma/Thaila nd	2020	2015-2016	Cohort	HI		SCID		4 weeks	8	78	396
Hossain[508]	Bangladesh	2020	2017	Cross-sectional	Community	24.98	SRQ-20	7	24-64 weeks	8	307	591
Nasreen[509]	Bangladesh	2013	2008	Cohort	Community	24.2	EPDS	9/10	24-32 weeks	9	207	652
Hanieh[510]	Iran	2017	2012-2016	Cohort	HI	26.1	EPDS	12	6-48 weeks	6	29	307
Dayan[511]	Iran	2018	2015	Cohort	HI	25.7	EPDS	12	6-8 weeks	8	56	174
Ezzeddin[512]	Iran	2018	2014	Cross-sectional	Community	28.62	EPDS	13	12-32 weeks	9	115	325
Ahmed[513]	Syrian/Canad a	2017	2015-2016	Qualitatively-dri ven mixed	HI	27.17	EPDS	10	48 weeks	9	7	23
Safadi[514]	Jordan	2015	2013-2014	Cross-sectional	HI	27.83	PHQ-9	10	12 weeks	9	79	315
Yehia[515]	Jordan	2013	2006	Cross-sectional	HI		EPDS	11	6 weeks	8	201	300
Shwartz[516]	Israel	2020	2014-2015	Prospective	HI		EPDS	10	24 weeks	9	109	1055

Lichter[517]	Israel	2020	2018-2019	Longitudinal	HI	31.64	EPDS	10	8 weeks	9	14	312
Freedman[518]	Israel	2020	2014	Cross-sectional	HI		EPDS	10	8-28 weeks	8	22	143
Goren[519]	Israel	2020	2014-2015	Longitudinal	HI	29.50	EPDS	13	36 weeks	7	7	114
Orbach-Zinger[520]	Israel	2017	2015-2016	Cohort	HI		EPDS	10	6 weeks	8	87	1326
Bina[521]	Israel	2014	2008-2009	Longitudinal	Community		EPDS	9	6 weeks	8	94	805
Alzahrani[522]	Saudi Arabia	2019	2016	Cross-sectional	HI	33.20	EPDS	13	8-10 weeks	8	37	217
Haight[523]	USA	2020	2012-2015	Prospective	System data		PHQ-2		4-6 weeks	9	2825	23990
Farr[524]	USA	2014	2009-2010	Cross-sectional	System data		EPDS	10	8-32 weeks	9	181	2012
Farr[525]	USA	2014	2009-2010	Prospective	System data		Responses to three statements	10	12-36 weeks	8	1558	4451
Mark[526]	Malawi	2020	2016-2017	Cross-sectional	HI	25.40	SRQ	8	16-20 weeks	8	34	175
Sari[527]	Finland	2013	2002-2010	Prospective	Database		ICD-10		6 weeks		1438	511522
Demirchyan[528]	Armenia	2014	2012	Prospective	Community		EPDS	13	24-28 weeks	9	19	146
Eckerdal[529]	Sweden	2016	2006-2007 2009-2012	Cohort	HI	31.10	EPDS	12	6 weeks	8	53	446
Falah-Hassani[530]	Canada	2016	2001-2002	Longitudinal	HI	28.70	EPDS	10	4-8 weeks	8	106	490
									4 weeks		110	478
									8 weeks		102	501
Kiviruusu[531]	Finland	2019	2011-2012	Cohort	HI		CES-D	10	12-96 weeks	8	163	1248
									12 weeks		149	1415

									32 weeks		190	1291
									96 weeks		151	1038
Sarberg[532]	Sweden	2016	2007	Prospective	HI		EPDS	10	9 weeks	7	29	293
Muchanga[533]	Japan	2020	2011-2014	Cohort	HI		EPDS	9	4 weeks	9	11255	80396
Asif[534]	Sweden	2020	2009-2019	Cohort	HI		EPDS	12	6 weeks	8	138	2990
Rosander[535]	Sweden	2020	2017	Cross-sectional	Web questionnaire		EPDS	12	84 weeks	9	247	888
Holm-Larsen[536]	Tanzania	2018	2014-2017	Cohort	HI		EPDS	13	6 weeks	9	138	1128
Maliszewska[537]	Poland	2017	2013-2014	Cross-sectional	HI	29.49	EPDS	12	4-8 weeks	8	48	387
Maliszewska[538]	Poland	2017	2013-2014	Cross-sectional	HI		EPDS	12	4-12 weeks	9	58	548
Gray[539]	Australia	2013	2007-2009	Case control	HI		EPDS	12	48 weeks	8	17	199
Shah[540]	Pakistan	2017	2015	Cross-sectional	HI		EPDS	10	6-48 weeks	8	75	434
Fellmeth[541]	UK	2019	2014	Cross-sectional	Data information		EPDS	10/13	12 weeks	8	945	6752
								13			612	6752
								10			1277	6752
Cruise[542]	Ireland	2017	2013	Cohort	Data information		CESD	7	36 weeks	9	1176	10895
Wesseloo[543]	Netherlands	2017	2013-2014	Cohort	Community		EPDS	13	16 weeks	9	68	1075
Fritel[544]	France	2016	2003-2006	Cohort	HI	29.5	EPDS	10	16-48 weeks	9	232	1226
									16 weeks		206	1226
									48 weeks		258	1226

Binda[545]	Chile	2019	2013-2015	Prospective	HI	25.5	EPDS	10	8-48 weeks	8	83	177
Leite[546]	Brazil	2020	2011-2012	Cross-sectional	HI		EPDS	13	24-48 weeks	9	2782	11725
Ferrari[547]	Italy	2020	2012-2017	Observational	HI		EPDS<br>DSM-5	9	6-8 weeks	8	454	3102
Epifanio[548]	Italy	2015		Cross-sectional	HI		EPDS	9	4 weeks	8	11	53
Vismara[549]	Italy	2016	2015	Prospective	HI	35.03	EPDS	13	12-24 weeks	8	32	181
Vismara[550]	Italy	2017	2012	Prospective	HI	33.50	EPDS	12	8-12 weeks	9	126	2706
Palumbo[551]	Italy	2016	2012-2014	Prospective	HI	32.50	EPDS	12	6-12 weeks	9	110	1558
Gremigni[552]	Italy	2011	2007-2008	Prospective	HI	31.17	EPDS	9	12 weeks	8	39	70
Turner[553]	Italy	2006	2004-2005	Prospective	HI		EPDS	9/10	5-8 weeks	8	14	70
Albacar[554]	Spain	2011	2003-2004	Cohort	HI	31.70	EPDS	9	32 weeks	8	65	729
Albacar[555]	Spain	2009	2003-2004	Cohort	HI	31.00	EPDS	9/10	32 weeks	9	87	1053
Jin[556]	New Zealand	2020	2016-2017	Cohort	HI	31.50	EPDS	10	12-48 weeks	8	12	79
									12 weeks		16	86
									24 weeks		9	79
									48 weeks		10	71
Chan[557]	New Zealand	2018	2013-2015	Cohort	HI		EPDS	13	24 weeks	8	15	229
Gould[558]	Australia	2015	2005-2008	Prospective	HI		EPDS	12	6-24 weeks	9	99	1037
									6 weeks		97	1037
									24 weeks		100	1037
Pingo[559]	South Africa	2017	2005-2010	Cohort	HI	24.00	EPDS	13	6 weeks	9	26	57

Madeghe[560]	Kenya	2016	2014	Cross-sectional	HI		EPDS		6-14 weeks	9	26	200
Xie[561]	China	2011	2007	Cohort	HI		EPDS	13	4 weeks	8	103	534
Tychey[562]	France	2005	2004	Cohort	HI		EPDS	12	4-8 weeks	8	31	277
Alasoom[563]	Saudi Arabia	2014	2013	Cross-sectional	HI		EPDS	10	8-24 weeks	8	80	450
Shivalli[564]	India	2015	2012	Cross-sectional	HI	23.1	EPDS	13	4-10 weeks	8	32	102
Lambrinoudaki[565]	Greece	2010	2009	Cross-sectional	HI	32.69	EPDS	11	6 weeks	8	13	57

Supplementary table 2. Characteristics for including studies.

Study	Marital status		Employment		Educational level		Sex of Infant		Parity		Social support (parents/ in-low/ family /friend)		Patner support (assistance)		Violence (IPV)		Maternal age range		Residence	
	married/cohabiting	single/divorced/widowed	Yes	No	>12 years	《12 years	Male	Female	Primiparous	Multiparous	Yes	No	Yes	No	Yes	No	Adolescent	Adult	Urban or rural	Suburb
Ramchandani							81/500	89/535	61/404	109/631										
Ndokera					3/34		14/152	13/126									2/25	25/253		
Weobong	401/12174	54/1186	313/9629	142/3731	433/13533	22/827	229/6843	226/6517	118/3038	366/10332							52/1511	403/11849	128/4089	358/9840
Mohammed					98/195	1/5	58/113	41/87	14/37	85/163	85/178	14/22	79/176	20/24	24/38	75/162				
Mahenge	753/971	80/97	699/880	223/299	793/997	53/183											193/236	730/944		
Khalifa					11/144	9/92			9/162	11/154										
Stellenberg	34/55	45/102	31/64	49/95							52/123	28/36					19/38	61/121		
Wemakor	124/312	44/72					87/195	81/189									20/35	148/349		
Anokiye	7/142	11/115	12/235	6/22	3/13															
Glasser					56/265				13/79	52/209	22/186	43/102					5/16	60/272		
Chandran									11/152	22/149										
Inandi			63/287	621/2227	632/2283	52/232					394/1574	290/937					243/927	440/1583	586/2174	95/336
Fisher			102/371	64/135																
Aydin			34/106	211/602	219/615	26/93											13/40	232/423		
Wang									41/83	42/83										
Ho-yen											12/345	9/81			7/30	14/395				
Xie							21/174	31/126	27/152	25/148										
Chee					26/134	46/337	36/235	36/236	26/203	46/268	29/250	43/221								
Yusuff			44/391	146/939	168/1174	20/146	107/723	88/632			135/1022	47/277	170/1293	39/74						

Ogbo													299/10131	36/103	11/137	329/10339	8/228	392/11775		
Gausia											45/262	31/84	65/329	11/17	8/16	68/330				
Yurdagul																				
Yong Zhang															56/132	11/83				
Bener			149/955	94/424	188/1020	55/359	119/692	124/687	211/1231	32/148	182/1161	61/213								
Mathisen	28/77	4/9	16/42	11/31			13/45	19/41	13/47	19/39										
Gaillard	22/143	22/121	38/254	6/10					30/122	14/142					6/17	38/247				
Giri																	14/45	91/301		
Turkcapar			4/60	79/480											7/14	76/522				
Boratav			27/55	15/32	18/36	21/43	21/45	21/42												
Kunwar			3/27	26/73	26/83	3/17			17/61	12/39										
Zainal									18/274	10/137										
H. Corrêa					413/1917	184/1143											139/740	458/2320	300/1463	297/1597
Koutra	108/878	22/115	53/498	78/493	101/706	30/286	59/530	72/507	53/409	80/580										
Thang	110/589	6/10			85/404	31/196							85/531	31/69	14/36	102/564	2/12	114/588		
Xiong			241/446	14/22	87/155	176/313	137/224	126/224	148/252	115/216	96/204	167/264							148/269	115/199
Pampaka			66/613	91/734	57/426	100/921	97/712	60/636	41/424	115/922	33/332	124/1015								
Shakeel					38/345	22/294	25/309	32/307			43/565	17/78								
Kerie	68/323	70/85			128/365	10/37														
Vaezi			16/34	71/166	60/130	27/70														
Ongeri	29/150	3/21			27/125	5/46							17/116	15/55						
Shwartz	109/1056	7/64	54/675	61/439	64/379	52/742			29/326	87/794	43/668	73/448			43/402	73/719				
Nampijja	84/277	8/21	57/200	36/100			52/169	41/131	30/115	63/185	66/229	27/71			27/50	66/249				

Sharmin			14/37	89/363			28/144	75/256									14/35	89/365		
Azad			18/29	130/347							26/64	11/17	84/230	54/108	129/260	19/116	28/85	112/252		
Xiong			197/1087	5/37	98/553	104/571	94/706	108/418	123/362	79/762									108/597	94/527
Dlamini			14/40	40/74			23/57	31/57	20/43	34/71			19/68	35/46						
Shitu									32/75	109/521										
Nasr			15/38	52/136									59/163	8/11						
Xiong			293/846	125/384			321/659	187/571	145/436	273/794										
Asaye																				
Motoko					13/40	6/30			17/58	2/12			7/31	12/39						
Abbott	169/1097	55/266																		
Teng			6/111	18/92																
Adewuya	96/778	32/98					52/540	76/336												
Chee			7/111	12/167	4/79	15/199	9/128	10/150			6/188	13/90								
Husain			11/23	42/126			28/79	25/70							3/3	50/146				
Azidah			13/102	65/275	73/350	5/27	37/186	41/191	17/55	61/322	74/352	4/25	75/369	3/8						
Alami			2/5	25/95					12/45	15/55										
Dindar					151/522	23/157	76/323	98/356			99/471	75/208								
Montazeri																				
Figueiredo																	14/54	5/54		
Tychey							13/94	4/87												
Orhon			20/67	15/36	16/44	19/59														
Watanabe			16/119	14/116	10/63	20/172			15/111	15/124										
Krause	30/388	15/103				27/203	18/288													
Bjerke	12/192	3/5	13/136	2/61	10/124															
Grussu	38/294	1/2	35/265	2/21	31/238	7/49			36/268	3/28										

Faruk									4/14	7/20										
Signe																				
Kirpinar			3/31	64/512			38/224	29/255	23/134	44/345										
Blom					340/3503	56/1438	212/2457	184/2484												
Nagy			6/45	8/92	7/40	7/97														
Lobato	109/702	19/109			98/583	30/228											27/184	101/627		
Ross																			2/41	1/46
Ahmed							150/508	134/492							67/118	217/882	14/75	270/925		
Chien																				
Husain	60/230	3/7									45/192	18/45								
Demissie	29/543	14/109	21/342	22/310	17/95	26/557			20/320	23/331										
Mariam			10/29	29/103	15/61	24/71	23/81	16/51	28/101	11/31									34/114	5/18
Lucero	27/60	25/36			38/69	13/26														
Alexandre																	47/147	149/554		
Wisner	756/6706	619/2947			411/1910	982/8059														
Abbasi	120/2637	31/334			29/495	122/2477														
Burgut																				
Sadat			7/34	60/266	63/266	4/34														
Swapan				32/196							12/157	20/45								
Alharbi			30/82	87/270	53/156	64/196	61/185	56/167	36/115	81/237										
Abdollahi											319/1528	84/553								
El-Hachem			58/168	18/60	13/37	62/186	42/107	34/121	25/81	51/147	70/212	6/16								

Abdollahi			18/113	344/1887	321/1647	41/263	170/961	190/447	225/1144	137/766									199/985	163/925
Qandil					6/13	22/88	15/46	13/53	2/18	26/83									10/42	15/51
Nam									481/53948	185/27499									628/76362	38/5085
Islam									53/175	97/251	63/298	87/128			100/150	50276	28/106	122/320		
Hiroko.I	101/468	2/9	62/216	41/263	14/74	89/405														
Zaidi			3/37	16/112			4/63	15/86	10/111	9/38										
Kim	73/179	22/44	37/70	58/153			42/106	53/117												
Zhao																				
Badr					10/55	9/95	15/74	16/76	11/58	18/93	2/43	27/107								
Adamu	122/526	22/92	67/337	77/281	68/285	47/217	80/307	64/311	33/228	111/390	64/514	80/104			52/87	92/531				
Yoshihiro.M			21/227	85/544	29/221	77/550														
Min.W																				
Deepthi.N			23/129	168/761	121/549	70/341	96/463	95/427												
Ertmann	80/1262	47/779	51/991	35/321					44/634	42/678										
Hoge			6/40	11/31					5/26	7/14	16/65	0/5								
Labrague	9/86	18/79	10/33	17/132			18/125	9/40	5/59	22/106							9/39	18/126		
Gan					26/258	259/2283			252/2138	35/408	231/2267	56/279								
Bhusal			29/176	30/170					18/153	41/193					23/41	36/305				
Hege.S			2/18	20/132	22/135	0/15	2/73	20/77	8/80	14/70	14/132	8/18					0/15	22/135		
Abebe															47/84	66/427				
Maeda					21/75	175/902			156/591	40/386										
Simhi	80/978	4/22																		

Chan																				
Wubetu	30/261	18/47	33/267	15/41							14/194	34/114	40/286	8/22						
Dawadi									16/72	28/88			31/117	13/43	10/30	34/130	6/20	38/140		
Tan	140/611	7/26	100/451	46/185			75/343	77/308												
Alikamali			63/192	77/208	56/178	84/222			63/204	77/196										
McCoy.S	21/64	60/145															26/72	55/137		
Necho.M	60/283	42/95									92/315	10/63			61/90	41/288				
Salem.M	29/639	18/19	22/309	25/349	45/421	2/237	13/313	34/345	17/262	30/396	24/311	23/374								
Chalise			11/53	30/189	26/149	15/93			25/151	16/91	37/230	4/12								
Dallal.F	87/235	1/2	30/74	58/163	53/149	35/88	41/108	47/129					37/130	51/106			4/8	84/229		
Melo Jr	47/469	13/86			52/459	8/96	35/298	25/257	24/281	36/274	51/514	9/41	28/319	32/236	5/11	55/544	2/37	58/518		
Bener.A			149/890	159/769			168/843	140/816												
Alasoom.L	78/447	2/3	4/27	76/423	56/305	24/145					39/204	41/246	59/401	21/49						
Chalise.A							18/103	19/92	16/105	21/90	34/191	3/4					3/15	34/180		
Khadka.R			59/296	12/84			54/191	17/189											2/122	69/258
Eckerdal.P	188/1477	5/26			35/273	158/1230							120/1061	73/442	25/151	168/1352				
Silva	106/729	61/286															42/215	126/804		
Malta	159/1265	17/71			24/135	152/1203					102/1157	75/190								
Filha.M																	1229/4570	2064/8194		
Dennis.C	97/464	6/33			42/177	61/320			40/226	63/271										
Paulson.J			315/2649	417/2432	355/1934	378/3155											50/201	683/4888		

Chen.L																				
January	97/282	4/13							36/115	65/180										
Zejnullahu.V			11/68	41/179	39/151	13/96	30/134	22/113	35/172	17/75	29/157	23/90								
Giakoumaki.O	32/226	2/9	26/185	8/50	22/148	12/87			15/102	19/133									27/191	7/44
Schachman					11/18	23/51														
Abadiga.M	51/247	9/40	42/220	18/67	51/233	9/54			23/64	37/223	26/216	34/71			33/68	27/219				
Azale.T									40/504	345/2643	281/2578	104/569					11/107	374/3040	22/359	363/2788
Modayfer.O			12/86	66/485	50/361	28/190	42/297	34/265							9/33	69/538				
Meijer					62/716	75/904			52/677	85/943										
Meltzer-Brody	162/390	114/289													61/130	215/549				
Dmitrovic							8/93	14/97	16/141	7/53										
Kerstis					34/200	16/105			23/130	27/175										
Woolhouse	165/1251	9/54	133/1126	38/155	49/339	124/959														
Petrosyan																				
Wubetu			12/143	9/80	11/102	10/121					16/196	5/27								
Shivalli			13/27	19/75			13/61	19/41	18/69	14/33							13/36	19/66		
Alasoom	78/447	2/3	4/27	76/423	56/305	24/145					39/204	41/246	59/401	21/49						
Edvinsson	33/155	0/2			12/37	21/120			15/76	18/81										
Liu.S					7/139	52/743	32/462	27/420	51/635	8/247										
Cirik.D			3/24	32/125					21/88	14/61							5/14	30/135		
Jenny.S	52/520	8/67	5/36	8/89	4/48	56/532	27/300	33/287	51/470	9/117										
Nicklas.J	22/59	2/12			4/12	20/59			8/33	16/38										
Nakano.M			51/554	42/495			43/543	48/489	53/424	38/588	82/990	5/17								
Shakeel.N	55/604	4/34			38/345	22/294			27/298	33/345										

Gaffney.K					65/271	284/1176	185/721	164/726												
Glynn.L					6/20	28/150														
Cury.A	142/514	54/187							56/246	140/455	79/458	117/243					47/147	149/554		
Rawahi.A			41/196	85/396	73/318	53/274			50/237	76/355										
Nhi.T			95/1105	9/169	68/717	36/557					59/1028	45/245			13/45	91/1229				
Do.T					8/47	24/69			22/61	10/55									16/50	16/66
Roomruangwong.C			41/217	12/96	25/171	28/142	30/156	23/157	32/196	21/117										
Panyayong.B	137/1682	7/40									263/1465	116/263	89/1297	52/395	33/205	112/1527	19/278	126/1453		
Tomlinson.M											80/151	51/64			24/30	107/185				
Tran			95/1104	9/169	72/716	32/557					59/1028	45/245			74/639	30/630				
Faisal-Cury	157/574	39/127															47/147	149/554		
Lobato	150/702	47/109	77/393	120/418	174/583	23/228											59/184	138/627		
Sunnqvist	26/93	148/1602	148/1648	27/98																
Gregory	266/1210	80/291			198/781	128/650			126/488	220/1013										
Woolhouse	76/1206	6/52	66/1096	15/138	21/321	60/930														
Woolhouse	191/1233	19/70	163/1126	43/155	54/339	154/959									74/216	134/1082				
Iranpour			10/29	115/330	97/276	28/83	59/186	66/173	86/200	57/159										
Emerson					12/81	19/118														
Birmingham	20/118	25/77	11/48	34/147	23/80	22/105							34/156	11/35						
Gong			136/647	66/280	89/385	157/731			64/293	183/803										
Li							43/297	47/225					72/469	18/53						
Liu					186/710	93/494					143/8	61/11								

											67	9								
Cao	167/465	402/1194			501/1445	68/214			410/1151	159/508										
Liang			182/618	71/227	138/462	115/383			119/397	134/448										
Wan					15/100	38/242			29/159	23/174			39/292	14/49						
Quan			19/277	7/83			13/180	12/169	15/236	11/124										
Gao	24/284	13/56			22/162	15/178	19/174	18/166					25/280	12/60						
Xie							31/295	72/239												
Brito			62/319	212/737							37/317	237/739					33/146	241/910		
Tannous	39/224	17/47	16/105	40/166													10/43	33/170		
Choi									9348/706375	315/19948							208/10201	17275/1038198		
Kim					17/280	78/399													67/357	25/310
Suhitharan	61/478		23/188	23/146	19/121	43/358														
Alves	48/182	8/12																		
Hossain							161/305	146/286							99/128	208/463				
Dayan			8/16	48/158	49/147	7/27	24/84	32/90	25/73	31/101										
Ezzeddin			12/44	103/281	34/71	81/254			51/157	64/168										
Haight	1404/14453	1421/9537			1346/9369	1479/14621			1187/10001	1638/13989							268/1418	2557/22572		
Mark																	5/39	27/129		
Demirchyan																				
Eckerdal					14/129	67/317			25/212	28/234			30/286	23/160						
Sarberg	28/289	1/4							14/141	15/152										
Muchanga	10208/76854	690/2939			4706/27963	6303/52309	5682/41062	5346/39332	4165/23741	6715/55877			8736/67412	2292/4247	353/961	10675/79434				
Asif					45/612	85/2190			65/1415	74/1568										
Holm-Larsen					134/107	4/56	73/579	65/549							71/33	67/795	12/137	126/11		

					2										3			17		
Fellmeth	473/5915	139/837							287/3320	325/3432							20/128	592/6624		
Ferrari	441/3041	13/61	340/2417	114/685	29/210	435/2892			275/1684	179/1418										
Palumbo	103/1496	7/62	75/1217	35/341	76/947	34/611			76/1072	34/486	78/1399	32/158	88/1438	22/118						
Albacar	63/713	2/16	41/559	24/170	52/532	13/197			22/282	43/447										
Albacar	84/1024	3/29	52/755	35/298	71/774	16/279			46/486	41/567										
Madeghe	17/174	9/26	4/67	16/118	26/197	0/3											4/21	22/179		

**Study**	**Religion**		**Mode of delivery**		**Ethnicity**		**Location of delivery**		**Gestational age**		**Breast Feeding**		**Infant death**		**Sleeping**		**Pregnancy plan**	
	**Yes**	**No**	**Spontaneous**	**Instrumental/caesarean**	**Indigenous**	**Nonindigenous/immigrant**	**Home**	**Health facility**	**Term**	**Pre-/post-term**	**Yes**	**No**	**Yes**	**No**	**Satisfactory**	**Unsatisfactory**	**Yes**	**No**
Ramchandani																		
Ndokera			27/268	0/10			13/107	14/171	10/124	17/154	27/273	0/5						
Weobong	409/12332	46/1028	419/12520	36/840	165/5791	319/7569	171/4625	284/8735	372/11026	107/2334			39/581	408/12779			235/7078	251/6282
Mohammed															45/126	54/74		
Mahenge																		
Khalifa							0/18	20/198										
Stellenberg			71/133	9/25													25/46	42/96
Wemakor	158/362	10/22			148/341	20/43												
Anokiye																		
Glasser					46/235	19/53											32/89	33/196
Chandran																		
Inandi																		
Fisher			141/453	25/53								54/137					86/358	80/148
Aydin																		

Wang																		
Ho-yen																		
Xie			21/117	31/183														
Chee					34/233	38/238												
Yusuff			172/1227	22/120	152/1046	43/316											99/763	94/576
Ogbo			219/7865	206/4856														
Gausia			61/263	15/83			36/150	40/196					6/10	70/336			45/246	31/100
Yurdagul																	100/587	65/198
Yong Zhang																	9/102	58/113
Bener									202/1203	41/176	188/1202	55/177					117/734	126/643
Mathisen			7/29	25/57													13/43	19/43
Gaillard			29/170	15/94	29/212	15/52											26/175	18/89
Giri																		
Turkcapar											78/520	3/17	1/7	82/533			60/430	23/107
Boratav																	14/54	28/33
Kunwar			24/68	5/32													19/74	10/26
Zainal			17/333	11/78							1/188	27/223					22/361	6/50
H. Corrêa			238/1598	236/1260														
Koutra			66/515	75/519	129/952	11/72			121/926	20/111		22/148				18/55	79/547	62/395
Thang																	27/100	89/500
Xiong			121/208	141/259							242/438	21/30			31/58	232/410	144/286	119/182
Pampaka					48/340	109/1008												
Shakeel					47/371	13/272												
Kerie																		
Vaezi			33/71	54/129													69/168	18/32
Ongeri			27/139	5/32									5/11	27/160				
Shwartz	79/708	37/412			92/886	22/230			96/923	19/195							87/993	28/117

Nampijja	77/249	16/51	59/184	34/116					80/261	13/38								
Sharmin			60/238	43/162														
Azad									118/309	28/58							76/254	72/122
Xiong			61/373	141/751							185/1005	17/119					160/864	42/260
Dlamini			51/104	3/10					45/94	9/20							11/35	43/79
Shitu			92/373	49/223														
Nasr			33/99	34/75							49/134	18/40					51/134	16/40
Xiong			307/931	111/299													183/572	235/658
Asaye									88/440	41/86							72/382	57/144
Motoko			14/50	5/20														
Abbott															116/774	24/72	65/506	158/855
Teng											18/171	6/32						
Adewuya			97/783	31/93					69/687	59/189								
Chee					12/147	7/131					15/199	4/79					11/191	8/87
Husain																		
Azidah			71/354	7/23									3/9	75/368			32/163	46/214
Alami			23/87	4/13							26/98	1/2					20/72	7/28
Dindar																		
Montazeri			9/50	13/50														
Figueiredo																		
Tychey																		
Orhon																		
Watanabe																		
Krause			27/301	18/182							24/302	21/189						
Bjerke			9/155	6/42							13/189	2/8						
Grussu			22/220	14/68														
Faruk																		

Signe																		
Kirpinar			49/387	18/92			8/44	59/435			55/454	19/43	7/11	60/468			39/322	28/157
Blom			252/3232	144/1709	160/3105	236/1836	25/833	369/4081	372/4710	24/231							275/3949	121/992
Nagy					7/74	7/63					11/125	3/12						
Lobato									114/742	14/69								
Ross																		
Ahmed			124/521	160/479							221/811	63/189					234/468	50/100
Chien					16/190	78/190												
Husain					14/46	49/191												
Demissie																		
Mariam			26/92	13/40														
Lucero			39/72	9/20							42/79	10/17						
Alexandre																		
Wisner																		
Abbasi																	87/2020	64/952
Burgut					146/837	97/542												
Sadat			36/150	31/150													54/263	13/37
Swapan			26/161	6/41													29/185	3/17
Alharbi			88/269	29/83					111/338	6/14								
Abdollahi																	294/1647	108/428
El-Hachem			12/60	64/186	73/224	3/4			59/189	16/38	67/215	7/11		75/227				
Abdollahi																		
Qandil			22/80	6/21			2/3	26/98									10/52	18/49
Nam			378/52330	288/29117														
Islam			117/295	33/131													103/319	47/107
Hiroko.I			70/370	33/109							99/472	4/7						
Zaidi			7/81	12/68													11/115	8/34

Kim			46/126	49/97														
Zhao																		
Badr			13/91	16/59														
Adamu			99/416	45/202									19/40	125/578			87/439	57/179
Yoshihiro.M																		
Min.W			37/242	31/193														
Deepthi.N			93/448	98/424									42/124	149/766				
Ertmann															45/273	41/1039		
Hoge					13/47	4/24											13/59	3/11
Labrague			26/160	1/5							26/157	1/8					6/47	21/118
Gan															88/1250	199/1292		
Bhusal			43/234	16/112			2/9	57/337										
Hege.S			15/100	7/50													14/108	8/42
Abebe			80/370	33/141													83/422	30/89
Maeda			100/557	96/420														
Simhi	44/533	40/467			55/766	29/234												
Chan			21/70	22/135														
Wubetu																	35/260	13/48
Dawadi			32/131	12/29									4/5	40/155			21/77	23/83
Tan			106/471	46/179														
Alikamali			67/225	73/175													117/260	83/140
McCoy.S			58/163	23/46							16/70	65/139						
Necho.M			57/273	45/105													43/248	53/124
Salem.M			12/332	35/326					32/437	15/221							10/525	37/133
Chalise																	26/188	15/54
Dallal.F			63/128	25/55					79/214	9/23	85/230	3/6					60/171	28/66
Melo Jr			32/295	28/260							1/34	59/521						

Bener.A																	
Alasoom.L		64/360	16/90							50/327	30/123					38/303	42/147
Chalise.A		22/131	15/64													17/157	20/38
Khadka.R		67/336	4/44			43/131	28/249			48/285	23/95			27/242	44/138		
Eckerdal.P		136/1138	114/730														
Silva																	
Malta				128/1026	48/313												
Filha.M																	
Dennis.C																	
Paulson.J																	
Chen.L		4/36	31/144														
January				83/236	18/59												
Zejnullahu.V		44/177	8/70							35/184	17/63					49/240	3/7
Giakoumaki.O		11/97	23/138	25/166	9/69												
Schachman																	
Abadiga.M		42/205	18/82			15/45	45/424									22/206	38/81
Azale.T																	
Modayfer.O		52/398	25/168					66/439	12/132							43/393	7/35
Meijer																	
Meltzer-Brody																	
Dmitrovic				10/99	13/96												
Kerstis																	
Woolhouse				128/1000	45/298												
Petrosyan																	
Wubetu		13/151	8/72														
Shivalli		26/84	6/18					30/99	2/3							20/55	12/47
Alasoom		64/360	16/90							50/327	30/123					38/303	42/147
Edvinsson										25/140	8/17						

Liu.S			34/530	25/352					48/839	11/43	54/866	5/16						
Cirik.D																		
Jenny.S											50/520	10/67						
Nicklas.J			8/40	16/31							17/56	7/15						
Nakano.M			66/752	26/291					88/1009	5/40	74/902	19/148						
Shakeel.N																		
Gaffney.K																		
Glynn.L																		
Cury.A									155/571	41/130							50/225	146/476
Rawahi.A																	63/348	63/244
Nhi.T									92/1200	10/57								
Do.T	2/13	30/103									31/111	1/5					25/101	7/15
Roomruangwong.C			30/190	23/123							29/186	19/102					34/175	19/138
Panyayong.B									108/1434	37/314	128/1643	17/86					52/895	87/801
Tomlinson.M															50/117	81/98		
Tran									92/1200	10/57								
Faisal-Cury																		
Lobato			127/478	70/333					174/760	23/51								
Sunnqvist																		
Gregory					20/71	326/1430												
Woolhouse					62/975	20/283												
Woolhouse					154/1000	55/298												
Iranpour			44/132	81/227											88/187	35/165		
Emerson																		
Birmingham																		
Gong																		
Li			47/205	43/217							50/457	40/65						

Liu	14/72	265/1132									245/1054	34/150					221/1025	58/179
Cao			293/833	276/826					541/1564	28/95								
Liang			158/522	95/323	107/405	146/440												
Wan									46/321	4/17								
Quan									24/332	0/26							17/241	8/112
Gao			17/202	20/138							19/249	18/91					25/275	12/65
Xie																		
Brito																	83/420	191/636
Tannous	25/132	31/139																
Choi			9784/805946	7699/455485														
Kim																		
Suhitharan			49/382	12/92	60/472	2/7											36/302	26/177
Alves																		
Hossain																		
Dayan			40/119	16/55														
Ezzeddin			19/72	96/253													77/258	38/67
Haight					516/5357	2309/18633												
Mark																		
Demirchyan																		
Eckerdal									50/422	3/24					22/266	31/180		
Sarberg			21/242	8/51					26/287	3/6								
Muchanga			6059/46514	4920/33542					8683/64938	2345/15457	3608/34479							
Asif									133/2819	6/171								
Holm-Larsen									126/1063	12/65								
Fellmeth			349/3811	252/2834	486/5458	121/1235									393/6040	219/712	404/5242	206/1461
Ferrari			286/2022	168/1080	402/2847	52/255											378/2694	76/408

Palumbo					96/1474	14/84					73/1081	37/477			43/1150	67/408		
Albacar			57/640	8/89							61/646	4/83						
Albacar																		
Madeghe																		

Study	Gender preference		Financial problems		Partnership		Life Stress		Smoking		Alcohol		Family type		Living condition		Number of children	
	**Yes**	**No**	**Yes**	**No**	**Intimate**	**Unsatisfied**	**Yes**	**No**	**Yes**	**No**	**Yes**	**No**	**Nuclear**	**Extended**	**Satisfactory**	**Unsatisfactory**	**1**	**2 or more**
Ramchandani																		
Ndokera																		
Weobong	198/5794	288/7566																
Mohammed			53/79	46/121														
Mahenge																		
Khalifa																		
Stellenberg					36/94	44/65												
Wemakor																		
Anokiye																		
Glasser			37/112	28/176	37/243	28/43												
Chandran	10/42	23/259					6/15	27/286										
Inandi																		
Fisher					137/349	29/157												
Aydin													134/434	102/184				
Wang																		
Ho-yen							15/157	6/268	4/18	17/407								
Xie															38/263	14/37		
Chee																		

Yusuff					179/1321	14/28												
Ogbo																		
Gausia	37/169	39/177			69/338	7/8							49/200	27/146				
Yurdagul																		
Yong Zhang	23/66	44/149							7/15	60/200	36/87	31/128						
Bener	88/448	155/931	186/975	57/404	171/818	68/473												
Mathisen			6/12	26/74					3/7	29/79								
Gaillard																		
Giri							20/34	85/312										
Turkcapar													53/360	30/180			64/440	19/95
Boratav																		
Kunwar																		
Zainal																		
H. Corrêa																		
Koutra									46/297		28/192							
Thang			23/83	93/517			68/199	48/401										
Xiong					154/281	109/187												
Pampaka							75/351	81/989										
Shakeel							30/136	24/470										
Kerie																		
Vaezi																		
Ongeri			17/59	15/112	14/132	18/39											10/64	22/107
Shwartz							89/536	22/549									44/396	72/725
Nampijja																		
Sharmin													77/297	26/103			91/186	12/214
Azad					90/271	48/67	94/143	54/233									62/183	86/193
Xiong													69/387	133/737				

Dlamini																		
Shitu	48/277	93/319															37/91	104/505
Nasr							35/67	32/107										
Xiong			251/510	167/720	208/842	210/388												
Asaye																		
Motoko							13/51	6/19										
Abbott			124/472	100/891	150/1101	44/73									145/1073	79/288		
Teng																		
Adewuya																		
Chee	1/10	18/268			11/243	8/35											6/115	13/163
Husain			14/35	39/114	48/136	5/13									46/133	7/19		
Azidah			44/158	34/219	54/296	24/81												
Alami			5/28	22/72	23/80	4/20												
Dindar					117/576	57/103			60/141	114/538								
Montazeri																		
Figueiredo																		
Tychey																		
Orhon																	22/64	13/39
Watanabe											2/25	28/210						
Krause																	16/201	29/190
Bjerke					10/182	4/12	2/171	13/26					9/130	6/67			2/59	13/138
Grussu																		
Faruk																		
Signe					359/2571	103/241	117/1324	331/1367										
Kirpinar													52/324	15/155				
Blom																		
Nagy							7/42	7/95										

Lobato																		
Ross																		
Ahmed									13/33	271/967			172/603	112/397				
Chien																		
Husain																		
Demissie									10/53	33/599								
Mariam													12/47	27/85				
Lucero																		
Alexandre																		
Wisner																		
Abbasi																		
Burgut																		
Sadat																	30/147	37/153
Swapan	13/48	19/154			21/184	11/18	25/39	7/163					16/78	16/124	17/135	15/67		
Alharbi																		
Abdollahi					274/1210	130/872	103/633	301/1347					132/583	272/1500				
El-Hachem																		
Abdollahi													250/1379	112/531				
Qandil																		
Nam																		
Islam	98/184	52/242					129/243	21/183									110/332	40/94
Hiroko.I																		
Zaidi													8/55	11/94				
Kim																	13/51	82/172
Zhao					43/109	36/58	48/127	31/40										
Badr					10/89	19/61					17/26	12/124						
Adamu	66/343	78/275	71/210	73/408	77/505	67/113	6/21	138/597										
Yoshihiro.M																		

Min.W																		
Deepthi.N																		
Ertmann									8/86	78/1226	1/10	85/1302						
Hoge							12/35	4/35										
Labrague					10/78	17/87			2/7	25/158			16/95	11/70				
Gan			58/306	219/2191					2/8	283/2530	42/231	245/2311						
Bhusal					32/300	27/46	14/42	45/304										
Hege.S													7/33	16/117				
Abebe							62/127	52/385										
Maeda																		
Simhi			28/188	56/812													46/383	37/611
Chan									1/7	42/198								
Wubetu			14/37	34/271														
Dawadi													24/69	20/91				
Tan									124/549	24/94	4/18	137/605						
Alikamali																		
McCoy.S									31/59	50/150								
Necho.M																		
Salem.M					25/312	22/346												
Chalise									5/11	36/231			13/55	28/187				
Dallal.F																		
Melo Jr									5/20	55/535	13/44	47/511						
Bener.A									20/96	288/1563								
Alasoom.L	47/225	33/225					41/139	39/311					65/384	15/66				
Chalise.A																		
Khadka.R													34/126	37/254			16/103	55/277
Eckerdal.P							19/75	174/1428	68/450	125/1053								
Silva							115/568	51/442	55/206	113/923								
Malta																		

Filha.M																		
Dennis.C									16/64	87/433								
Paulson.J																		
Chen.L																		
January																		
Zejnullahu.V					42/224	10/23			6/18	46/229			13/75	39/172				
Giakoumaki.O									4/17	30/218	1/13	33/222						
Schachman																		
Abadiga.M					37/203	23/84	11/38	49/249										
Azale.T					305/28<br>56	80/291												
Modayfer.O					75/565	3/6	36/153	42/418										
Meijer																		
Meltzer-Brody																		
Dmitrovic																	21/188	2/7
Kerstis																		
Woolhouse									58/354	111/916								
Petrosyan																		
Wubetu					15/186	6/37												
Shivalli													7/37	25/65				
Alasoom	47/225	33/225					41/139	39/311					65/384	15/66				
Edvinsson									1/4	32/153	5/40	28/117						
Liu.S																		
Cirik.D									6/12	29/137								
Jenny.S																		
Nicklas.J																		
Nakano.M																		
Shakeel.N							37/288	19/302										
Gaffney.K									71/191	278/1256								
Glynn.L																		

Cury.A																		
Rawahi.A																		
Nhi.T	81/845	23/423																
Do.T							23/90	9/23					11/48	21/68				
Roomruangwong.C									1/4	52/309	1/8	52/305						
Panyayong.B			75/472	69/1258														
Tomlinson.M																		
Tran	81/850	23/423																
Faisal-Cury																		
Lobato							152/477	45/334			84/272	113/539						
Sunnqvist			113/845	62/900					48/332	126/1365	89/905	85/783						
Gregory																		
Woolhouse									8/128	75/1100								
Woolhouse																		
Iranpour																		
Emerson																		
Birmingham			19/33	26/162											22/153	23/42	25/130	20/65
Gong	76/285	167/824			239/1106	2/4							87/426	156/649				
Li													31/179	59/343				
Liu									6/10	273/1194								
Cao																		
Liang			100/332	153/513	217/720	36/125												
Wan					44/308	9/33												
Quan																		
Gao							15/60	22/280										
Xie																		
Brito																		
Tannous																		

Choi																		
Kim																		
Suhitharan																		
Alves																		
Hossain															18/37	289/554		
Dayan																		
Ezzeddin			42/74	73/249														
Haight							2248/16588	577/7402										
Mark																		
Demirchyan																		
Eckerdal																		
Sarberg							2/10	27/283	1/12	28/281								
Muchanga									476/3527	10129/76027	1005/8182	9918/71559						
Asif																		
Holm-Larsen									2/4	136/1124	21/128	117/1000						
Fellmeth																		
Ferrari																		
Palumbo			25/189	85/1369	94/1509	16/49												
Albacar																		
Albacar																		
Madeghe																		

Supplementary Table 3. Sensitivity analysis and leave-one-out analysis for including studies.

	resid	se	z
542	-4.3093	0.7894	-5.4589
201	-3.2344	0.7997	-4.0444
9	2.8534	0.8048	3.5456
515	-2.7062	0.8025	-3.3722
444	-2.3904	0.8311	-2.8762
530	2.2819	0.8146	2.8014
67	2.5116	0.8969	2.8003
449	-2.2811	0.8501	-2.6833
336	2.1528	0.8181	2.6313
167	2.1711	0.8491	2.5569
281	2.0977	0.84	2.4973
263	2.0685	0.8318	2.4867
452	2.0176	0.8186	2.4647
225	1.9496	0.8217	2.3725
346	1.859	0.8075	2.3022
277	1.8607	0.8134	2.2876
70	1.8221	0.8127	2.2421
37	-1.8014	0.8088	-2.2272
28	1.838	0.8268	2.223
233	-1.8004	0.8156	-2.2074
6	-1.7786	0.8088	-2.199
567	1.8023	0.8429	2.1381
423	1.9779	0.9283	2.1307
523	1.6505	0.8122	2.0322
259	1.6122	0.8245	1.9554
12	1.585	0.8238	1.9241
528	1.9084	0.9987	1.9109
78	1.556	0.8151	1.9089
329	1.6005	0.8427	1.8994
8	1.5524	0.8207	1.8915

16	-1.6397	0.8706	-1.8833
38	-1.5116	0.811	-1.8637
408	-1.4665	0.8157	-1.7979
549	-1.4615	0.8134	-1.7968
57	1.5032	0.8367	1.7965
565	-1.4522	0.8138	-1.7844
215	1.4644	0.8235	1.7783
90	1.4669	0.8303	1.7667
149	-1.7646	1.0001	-1.7644
422	-1.5627	0.886	-1.7638
560	1.4478	0.8227	1.7597
532	-1.4908	0.8539	-1.7458
416	-1.4418	0.8302	-1.7366
439	1.4119	0.8171	1.7278
286	1.418	0.8247	1.7194
360	-1.3774	0.8114	-1.6977
77	-1.4138	0.8356	-1.6918
172	-1.3603	0.8133	-1.6726
325	-1.4464	0.8661	-1.67
440	1.3726	0.8235	1.6668
442	1.3729	0.8279	1.6583
30	-1.392	0.8395	-1.6582
111	-1.392	0.8395	-1.6582
574	1.3961	0.8518	1.639
430	-1.4283	0.8717	-1.6385
269	1.3607	0.8308	1.6379
248	1.3322	0.8194	1.6259
74	1.3105	0.8217	1.5949
198	1.2921	0.8205	1.5748
491	-1.3502	0.8619	-1.5665
434	1.2991	0.8304	1.5644
92	1.2691	0.8166	1.5542

214	1.2738	0.8206	1.5523
316	-1.2744	0.8213	-1.5517
115	1.3095	0.845	1.5497
504	1.2583	0.8173	1.5395
191	1.269	0.8257	1.5368
256	1.248	0.8121	1.5367
122	1.2746	0.8298	1.536
180	-1.274	0.8549	-1.4901
465	-1.2095	0.8211	-1.473
370	1.1933	0.8133	1.4673
394	-1.2136	0.8318	-1.4589
304	-1.1848	0.8123	-1.4585
25	1.1824	0.813	1.4542
313	1.1761	0.8115	1.4492
398	1.185	0.8239	1.4384
324	-1.1743	0.8201	-1.4318
154	-1.1624	0.816	-1.4245
353	1.1454	0.8117	1.4111
95	1.1597	0.8296	1.398
86	1.1396	0.8165	1.3957
558	-1.1275	0.8193	-1.3762
26	1.1561	0.8402	1.376
472	-1.1225	0.8176	-1.373
7	1.1229	0.8186	1.3717
503	1.1122	0.8146	1.3652
443	-1.1103	0.8147	-1.3629
261	1.1001	0.8115	1.3556
257	1.1141	0.8221	1.3552
384	1.0936	0.8132	1.3448
402	-1.1097	0.8253	-1.3445
121	-1.1332	0.8438	-1.343
467	-1.0954	0.8178	-1.3395

229	-1.0894	0.8174	-1.3327
331	-1.1043	0.829	-1.3322
535	-1.0884	0.8173	-1.3316
162	-1.0828	0.8251	-1.3124
401	-1.0676	0.821	-1.3004
534	-1.1589	0.899	-1.2891
218	-1.0993	0.8528	-1.2891
114	-1.0869	0.8458	-1.2851
412	-1.038	0.811	-1.2799
572	-1.09	0.8528	-1.2781
173	-1.0523	0.8255	-1.2747
290	1.0449	0.821	1.2728
59	-1.0479	0.8333	-1.2576
49	1.0482	0.8401	1.2477
102	-1.0444	0.8441	-1.2373
566	-1.0096	0.816	-1.2373
143	-1.0273	0.8361	-1.2287
348	0.9962	0.8115	1.2277
312	-1.0212	0.8396	-1.2162
274	-0.997	0.824	-1.2099
231	0.9971	0.8265	1.2065
396	0.9864	0.8179	1.2061
318	-0.9877	0.8232	-1.1998
527	0.9692	0.8183	1.1844
104	0.9773	0.828	1.1804
489	-0.985	0.8353	-1.1793
332	0.9789	0.8302	1.1791
202	0.9616	0.8164	1.1779
284	-0.9554	0.8115	-1.1773
540	0.9525	0.8107	1.175
48	-0.9477	0.8115	-1.1678
128	-0.9467	0.8108	-1.1677

334	-0.9671	0.8295	-1.1659
253	0.9524	0.8169	1.1659
52	0.9524	0.8176	1.1648
292	-0.9443	0.8108	-1.1646
388	-0.9434	0.8115	-1.1625
502	-0.939	0.813	-1.155
473	0.9444	0.8177	1.155
299	-0.933	0.8146	-1.1453
197	-0.9363	0.8179	-1.1447
288	-0.9275	0.8136	-1.1401
480	0.9213	0.8118	1.1349
381	0.9363	0.8252	1.1346
169	-0.9334	0.8249	-1.1316
245	-0.9968	0.8809	-1.1315
321	0.9186	0.8194	1.121
344	0.914	0.8167	1.1191
152	0.9353	0.836	1.1188
96	0.9073	0.8124	1.1168
80	0.9113	0.8203	1.1109
73	0.9001	0.8169	1.1018
206	-0.9773	0.8895	-1.0987
426	-0.9466	0.8688	-1.0895
132	-0.9278	0.8536	-1.0869
514	0.9189	0.847	1.0849
125	0.9071	0.8365	1.0843
285	-0.8757	0.8115	-1.079
144	-0.8722	0.8119	-1.0743
397	0.874	0.8179	1.0686
182	0.8738	0.8181	1.0681
43	-0.8812	0.8257	-1.0673
405	0.8991	0.8482	1.06
520	-0.8742	0.8261	-1.0582

283	-0.8742	0.8277	-1.0561
160	-0.8587	0.8144	-1.0543
355	0.8553	0.8124	1.0528
24	0.8543	0.8158	1.0471
359	-0.9027	0.8643	-1.0444
446	-0.8522	0.8167	-1.0434
458	-0.8522	0.8167	-1.0434
414	-0.8466	0.8136	-1.0406
391	-0.8464	0.8165	-1.0366
83	0.8479	0.8196	1.0345
570	-0.8391	0.818	-1.0257
175	-1.0218	1.0075	-1.0142
4	-0.8484	0.8379	-1.0126
451	-0.824	0.815	-1.0111
374	-0.8347	0.8288	-1.0071
244	-0.821	0.8183	-1.0032
456	0.8174	0.8151	1.0028
438	0.8159	0.815	1.0011
526	0.8258	0.8265	0.9992
369	-0.8136	0.8153	-0.998
409	0.8125	0.8198	0.9911
524	0.8058	0.8147	0.989
431	0.8082	0.8204	0.9852
189	-0.8064	0.8185	-0.9852
88	-0.817	0.8333	-0.9804
428	-0.7972	0.8134	-0.98
354	0.8048	0.8218	0.9793
252	-0.7886	0.8117	-0.9716
10	-0.8112	0.8434	-0.9618
170	0.7822	0.8145	0.9604
260	0.7734	0.8126	0.9518
184	0.7723	0.8155	0.947

44	0.7785	0.8237	0.9452
554	-0.8024	0.8492	-0.9449
432	0.7671	0.8147	0.9415
380	0.7883	0.8381	0.9406
138	0.8334	0.8895	0.9369
411	-0.7629	0.8177	-0.9329
471	-0.758	0.8133	-0.9321
270	-0.7925	0.8543	-0.9276
441	0.8088	0.8727	0.9268
569	-0.7555	0.8208	-0.9205
539	-0.7458	0.8142	-0.9159
14	0.746	0.818	0.9119
199	-0.9163	1.0089	-0.9082
54	0.7402	0.8189	0.9039
291	-0.7328	0.8108	-0.9038
383	-0.7331	0.8176	-0.8967
349	0.7237	0.8122	0.8911
350	0.7223	0.812	0.8895
27	0.7331	0.8249	0.8887
399	-0.8293	0.9355	-0.8866
482	0.7209	0.814	0.8856
131	-0.7252	0.8255	-0.8785
300	-0.7238	0.8278	-0.8744
113	-0.7189	0.8233	-0.8733
343	0.7599	0.8817	0.8619
72	-0.7054	0.8218	-0.8583
417	-0.7054	0.8218	-0.8583
213	0.6948	0.8124	0.8552
455	-0.6852	0.8117	-0.8442
165	0.7019	0.8327	0.8429
168	-0.734	0.8758	-0.8381
573	-0.6801	0.8175	-0.832

525	-0.6918	0.8337	-0.8298
516	0.6836	0.826	0.8276
377	-0.6952	0.8424	-0.8253
124	-0.6981	0.8497	-0.8216
58	0.6755	0.84	0.8041
156	0.6462	0.8137	0.7941
3	-0.661	0.8356	-0.7912
93	0.638	0.8134	0.7844
193	0.6545	0.8389	0.7802
207	-0.645	0.8356	-0.7718
462	-0.6267	0.8146	-0.7694
547	-0.6401	0.8339	-0.7675
421	0.6244	0.815	0.7661
460	0.6244	0.815	0.7661
136	-0.6222	0.8137	-0.7646
413	-0.6206	0.8152	-0.7614
32	-0.6286	0.8276	-0.7595
477	-0.6323	0.8332	-0.7589
550	0.6172	0.8141	0.7581
63	0.6253	0.8264	0.7567
507	0.606	0.8115	0.7468
238	0.5976	0.8131	0.735
404	-0.6042	0.8221	-0.735
166	-0.5946	0.8136	-0.7308
148	-0.5946	0.8149	-0.7296
200	0.6125	0.8406	0.7286
219	-0.5936	0.8169	-0.7267
429	0.5952	0.8196	0.7262
531	-0.5923	0.817	-0.7249
249	0.6013	0.8298	0.7247
447	0.6056	0.8369	0.7237
79	-0.5905	0.8166	-0.7231

20	0.5866	0.812	0.7225
356	-0.5864	0.8195	-0.7155
276	-0.5822	0.8234	-0.7071
145	-0.6044	0.8584	-0.7041
273	0.5753	0.819	0.7024
553	-0.5653	0.8226	-0.6872
110	0.576	0.8415	0.6846
98	0.5833	0.8541	0.6829
210	-0.5565	0.8203	-0.6785
161	0.5548	0.824	0.6732
453	-0.5595	0.8335	-0.6713
557	-0.5433	0.8114	-0.6695
500	0.5417	0.8109	0.668
294	-0.5415	0.8222	-0.6585
320	-0.5565	0.8528	-0.6525
112	-0.5399	0.8288	-0.6514
262	0.5431	0.8351	0.6504
494	-0.534	0.8293	-0.644
134	0.544	0.845	0.6438
368	-0.549	0.8529	-0.6437
505	0.5219	0.8139	0.6413
373	-0.5241	0.8176	-0.641
307	0.5142	0.8111	0.634
55	0.5326	0.8585	0.6203
116	0.5051	0.8156	0.6193
406	-0.5037	0.8156	-0.6175
235	-0.4944	0.8133	-0.6079
151	-0.5063	0.8344	-0.6068
322	-0.5025	0.8329	-0.6033
393	-0.5025	0.8329	-0.6033
305	-0.5036	0.8485	-0.5935
118	0.4969	0.8409	0.5909

76	-0.4779	0.8109	-0.5893
459	0.4876	0.8298	0.5875
463	0.4765	0.8164	0.5836
529	0.4762	0.8212	0.5798
227	-0.4686	0.8119	-0.5772
508	-0.5001	0.8668	-0.5769
436	0.4719	0.8187	0.5764
228	0.4835	0.8388	0.5764
240	-0.4625	0.811	-0.5703
242	0.4663	0.8177	0.5703
330	-0.5352	0.939	-0.5699
390	-0.4612	0.811	-0.5687
366	-0.4651	0.8262	-0.5629
22	0.4651	0.8275	0.5621
378	-0.4718	0.8406	-0.5612
255	0.4588	0.819	0.5602
158	0.4578	0.8196	0.5586
499	0.4545	0.817	0.5563
536	-0.4545	0.8183	-0.5554
71	-0.4502	0.8153	-0.5522
392	0.4513	0.8211	0.5496
538	-0.4449	0.8111	-0.5485
483	-0.4447	0.8132	-0.5469
97	0.4464	0.8172	0.5462
196	0.4718	0.8639	0.5462
155	0.4718	0.8639	0.5462
464	-0.4407	0.8135	-0.5418
495	-0.4415	0.8225	-0.5368
461	0.4337	0.815	0.5321
141	0.4348	0.8193	0.5307
544	-0.4346	0.824	-0.5274
371	-0.4431	0.8407	-0.527

497	0.4341	0.8264	0.5253
100	-0.4404	0.8395	-0.5246
243	0.4317	0.8251	0.5233
418	0.4243	0.8132	0.5217
15	-0.4194	0.8166	-0.5135
186	0.4218	0.8315	0.5073
362	0.4122	0.8126	0.5073
51	-0.4181	0.8313	-0.503
561	0.4027	0.8112	0.4964
352	-0.4015	0.8128	-0.494
551	-0.4015	0.816	-0.492
13	-0.4039	0.8257	-0.4891
91	0.3989	0.8167	0.4884
232	0.4146	0.8573	0.4836
490	-0.404	0.8375	-0.4824
511	0.3943	0.8198	0.481
264	0.4003	0.8331	0.4805
339	0.3935	0.8199	0.4799
437	0.3967	0.8279	0.4791
306	-0.4037	0.8437	-0.4785
133	0.3871	0.817	0.4738
46	-0.3878	0.8199	-0.473
484	-0.3832	0.8128	-0.4714
266	0.3897	0.8313	0.4688
251	0.3817	0.8162	0.4676
552	-0.3858	0.8255	-0.4673
403	0.3895	0.8336	0.4672
217	0.379	0.8165	0.4641
479	0.3718	0.8138	0.4568
445	-0.37	0.8158	-0.4536
424	-0.3729	0.8303	-0.4491
466	0.3649	0.8133	0.4487

335	-0.3887	0.8676	-0.448
358	-0.3721	0.8341	-0.4461
45	-0.3634	0.8203	-0.443
475	0.3664	0.8286	0.4422
364	0.362	0.8196	0.4417
139	0.3664	0.8313	0.4407
230	0.3629	0.8381	0.433
407	-0.3684	0.8514	-0.4327
164	-0.3579	0.8293	-0.4315
39	0.3507	0.818	0.4287
301	-0.346	0.8146	-0.4247
129	-0.3527	0.8342	-0.4228
187	-0.354	0.8473	-0.4178
211	-0.354	0.8473	-0.4178
17	0.3375	0.8231	0.41
69	-0.3329	0.8124	-0.4097
518	-0.3368	0.8223	-0.4096
126	0.333	0.8181	0.407
254	-0.3309	0.8144	-0.4063
35	-0.3284	0.8121	-0.4044
367	0.351	0.8702	0.4034
176	-0.3306	0.8233	-0.4016
546	-0.3265	0.8153	-0.4004
85	0.3403	0.8519	0.3995
575	-0.3318	0.8378	-0.3961
303	-0.3195	0.815	-0.392
543	-0.3306	0.8474	-0.3901
513	-0.3143	0.8112	-0.3874
135	-0.3203	0.829	-0.3863
150	-0.3168	0.824	-0.3845
476	0.3118	0.8142	0.3829
82	0.3102	0.8139	0.3812

157	-0.3125	0.8201	-0.381
239	0.3112	0.818	0.3804
146	0.3102	0.8211	0.3778
327	-0.3055	0.8112	-0.3766
84	0.3046	0.8199	0.3715
375	-0.3026	0.8151	-0.3713
40	0.3025	0.8213	0.3684
470	-0.3004	0.8162	-0.3681
275	0.3046	0.8288	0.3675
293	0.3046	0.8288	0.3675
21	0.3017	0.8255	0.3655
137	-0.3055	0.8369	-0.365
433	0.3007	0.8243	0.3647
212	0.2963	0.8176	0.3624
338	-0.3071	0.8518	-0.3606
333	0.3174	0.9046	0.3509
545	0.283	0.8184	0.3458
65	-0.28	0.8161	-0.3431
400	0.2805	0.8247	0.3402
365	-0.3026	0.8954	-0.3379
208	0.2753	0.8186	0.3363
363	-0.2746	0.8201	-0.3348
224	0.2728	0.8151	0.3347
496	-0.2738	0.8235	-0.3325
427	0.2699	0.8193	0.3294
11	0.2679	0.8154	0.3285
209	-0.2766	0.8443	-0.3276
221	-0.2671	0.8178	-0.3267
127	0.2709	0.8345	0.3246
188	0.2648	0.8168	0.3242
328	-0.2628	0.8111	-0.324
425	0.2623	0.8172	0.3209

357	-0.2518	0.8159	-0.3086
204	-0.253	0.8212	-0.3081
171	-0.2491	0.8116	-0.307
41	-0.2589	0.8498	-0.3046
195	0.2639	0.8692	0.3036
556	-0.2464	0.8118	-0.3035
548	-0.2461	0.8111	-0.3034
177	-0.2483	0.8196	-0.3029
2	-0.2484	0.8212	-0.3024
107	-0.247	0.817	-0.3023
34	-0.247	0.817	-0.3023
42	0.2464	0.8158	0.302
519	0.2499	0.829	0.3015
517	-0.2468	0.8186	-0.3015
287	-0.247	0.8207	-0.3009
323	0.2484	0.8259	0.3007
142	-0.2471	0.8217	-0.3007
351	0.2441	0.8131	0.3002
435	0.2425	0.8179	0.2966
395	0.2463	0.8343	0.2953
246	0.2437	0.829	0.294
454	0.2428	0.8271	0.2936
23	0.2432	0.8308	0.2928
420	-0.2419	0.8319	-0.2908
347	0.2395	0.8239	0.2907
205	0.2708	0.9326	0.2904
521	0.2483	0.8584	0.2893
308	-0.2325	0.8123	-0.2862
278	-0.2345	0.8197	-0.286
5	-0.2355	0.825	-0.2854
289	-0.2329	0.8186	-0.2846
282	-0.2343	0.8238	-0.2844

311	0.2285	0.8186	0.2791
106	0.2264	0.821	0.2757
386	-0.2284	0.8363	-0.2731
509	0.2248	0.8248	0.2726
36	-0.2199	0.8148	-0.2699
512	0.2226	0.8278	0.2689
315	-0.2129	0.8121	-0.2621
563	0.2303	0.8789	0.262
317	-0.2224	0.85	-0.2617
376	-0.2118	0.8224	-0.2575
468	-0.2024	0.8144	-0.2486
410	-0.1995	0.8123	-0.2456
481	-0.1995	0.815	-0.2448
310	0.2018	0.8293	0.2433
457	-0.1957	0.8118	-0.2411
101	-0.196	0.8167	-0.24
562	-0.1941	0.8127	-0.2389
486	-0.1962	0.8234	-0.2383
379	0.194	0.8173	0.2373
250	-0.1925	0.8251	-0.2334
345	0.1876	0.8141	0.2305
241	0.1876	0.815	0.2302
237	-0.1916	0.8432	-0.2272
192	-0.1852	0.8333	-0.2223
267	0.1837	0.8292	0.2216
419	0.1837	0.8334	0.2204
117	0.1837	0.8487	0.2165
81	-0.1758	0.8241	-0.2134
280	0.1754	0.8238	0.2129
326	-0.1729	0.812	-0.2129
568	0.1837	0.8643	0.2125
87	0.1762	0.8341	0.2112

314	-0.1736	0.8239	-0.2107
387	0.1757	0.8355	0.2103
506	0.1754	0.8363	0.2097
19	0.1697	0.8218	0.2065
389	-0.1649	0.8116	-0.2032
448	0.1646	0.8209	0.2006
522	0.1646	0.8209	0.2006
268	0.165	0.8254	0.1999
159	-0.1561	0.8249	-0.1892
501	0.1555	0.822	0.1892
61	0.1528	0.8124	0.188
18	-0.161	0.8613	-0.1869
342	-0.1514	0.821	-0.1844
123	0.1521	0.8305	0.1832
130	-0.149	0.8155	-0.1827
64	-0.149	0.822	-0.1812
258	0.1547	0.8549	0.1809
309	0.1547	0.8549	0.1809
492	-0.1494	0.8271	-0.1806
319	0.1486	0.8327	0.1784
541	0.1476	0.8333	0.1771
185	0.1423	0.813	0.1751
33	-0.1428	0.8211	-0.174
66	0.1415	0.8177	0.173
571	-0.1503	0.8695	-0.1729
498	0.1386	0.8185	0.1693
56	-0.1364	0.8198	-0.1664
533	-0.1353	0.8435	-0.1604
487	-0.1267	0.8247	-0.1536
474	-0.1264	0.8343	-0.1515
510	0.123	0.8246	0.1492
178	0.1199	0.8243	0.1454

247	-0.12	0.8262	-0.1452
337	-0.1269	0.8748	-0.1451
220	-0.1238	0.8554	-0.1447
194	0.1168	0.8132	0.1437
272	-0.1172	0.8171	-0.1435
297	0.1182	0.8314	0.1422
222	-0.1164	0.8218	-0.1416
559	0.1149	0.8144	0.1411
68	0.1191	0.8603	0.1384
147	-0.105	0.8168	-0.1285
109	0.1076	0.8501	0.1266
488	0.1024	0.8222	0.1246
75	0.1011	0.8344	0.1212
298	0.0992	0.8217	0.1207
181	-0.1005	0.8337	-0.1206
295	0.0954	0.8136	0.1172
340	0.0944	0.8223	0.1148
94	-0.0908	0.8123	-0.1118
105	-0.0903	0.8237	-0.1097
469	-0.0893	0.8142	-0.1097
179	-0.0896	0.8243	-0.1086
203	0.0803	0.8115	0.0989
216	-0.0886	0.9773	-0.0907
485	0.073	0.8156	0.0895
29	0.0743	0.8671	0.0857
163	0.0687	0.8312	0.0827
382	-0.0669	0.8131	-0.0823
190	0.0647	0.8129	0.0795
234	-0.0618	0.8379	-0.0738
53	-0.0612	0.8308	-0.0737
271	0.06	0.8207	0.0732
372	-0.0596	0.8257	-0.0722

1	-0.0573	0.8155	-0.0703
99	-0.0567	0.8144	-0.0696
265	0.0569	0.826	0.0689
302	-0.0528	0.8155	-0.0648
140	-0.0522	0.8127	-0.0642
89	0.0515	0.8148	0.0632
415	-0.0515	0.8534	-0.0604
108	0.0534	0.8906	0.06
183	-0.0489	0.8277	-0.059
50	-0.0398	0.8277	-0.0481
296	0.0383	0.8204	0.0467
385	0.0383	0.8204	0.0467
341	0.0354	0.8191	0.0432
103	-0.033	0.8195	-0.0402
564	0.0315	0.8342	0.0378
174	0.0275	0.8142	0.0338
47	0.0275	0.8142	0.0338
119	0.0255	0.8495	0.0301
450	-0.0207	0.825	-0.0251
279	-0.0201	0.829	-0.0242
60	-0.0196	0.8355	-0.0234
226	-0.0162	0.8234	-0.0196
361	0.0151	0.8594	0.0176
223	0.0125	0.8121	0.0154
236	-0.0123	0.8236	-0.0149
62	-0.0123	0.8236	-0.0149
537	-0.0124	0.8309	-0.0149
31	0.0075	0.8253	0.0091
493	0.0074	0.8185	0.009
153	-0.0056	0.8118	-0.0069
555	0.0039	0.821	0.0047
120	-0.0034	0.8372	-0.004

478	0.0011	0.8194	0.0013

	estimate	zval	pval	ci.lb	ci.ub	Q	Qp	tau2	I2	H2
1	0.172274	-45.4178	0	0.162829	0.182148	174619.3	0	0.656729	99.4358	177.2419
2	0.172322	-45.4128	0	0.162876	0.182197	174641.5	0	0.656604	99.43601	177.3064
3	0.172421	-45.4164	0	0.162975	0.182295	174643.9	0	0.655935	99.43557	177.1695
4	0.172465	-45.4232	0	0.16302	0.182338	174641.7	0	0.655466	99.43518	177.0463
5	0.172318	-45.4138	0	0.162872	0.182193	174642.1	0	0.656607	99.43607	177.3266
6	0.172721	-45.5262	0	0.163296	0.182571	173904.9	0	0.650757	99.42951	175.2878
7	0.171976	-45.5554	0	0.162559	0.18182	174467.9	0	0.654433	99.43411	176.7128
8	0.171871	-45.645	0	0.162473	0.181695	174438.7	0	0.652405	99.43243	176.1909
9	0.171532	-46.0851	0	0.162223	0.18126	172415.5	0	0.641487	99.42224	173.0829
10	0.172454	-45.4222	0	0.163009	0.182327	174642.7	0	0.655582	99.43529	177.0819
11	0.172191	-45.4389	0	0.16275	0.18206	174565.7	0	0.656603	99.43569	177.2088
12	0.171866	-45.6505	0	0.162469	0.181689	174475.7	0	0.652281	99.43237	176.1697
13	0.17236	-45.4118	0	0.162913	0.182236	174644.5	0	0.656413	99.43591	177.2777
14	0.17207	-45.4934	0	0.162641	0.181928	174522.7	0	0.65571	99.43516	177.041
15	0.172366	-45.41	0	0.162919	0.182242	174644.3	0	0.656403	99.43565	177.1961
16	0.172627	-45.4835	0	0.163194	0.182486	174631.8	0	0.652765	99.4329	176.3357
17	0.172175	-45.4461	0	0.162735	0.182042	174611.1	0	0.656509	99.43596	177.2927
18	0.172296	-45.4229	0	0.162852	0.182169	174643.5	0	0.656567	99.43616	177.3541
19	0.172217	-45.4322	0	0.162774	0.182087	174620.7	0	0.656665	99.43607	177.3265
20	0.172108	-45.4722	0	0.162674	0.18197	174077.9	0	0.656097	99.4339	176.6486
21	0.172184	-45.4432	0	0.162744	0.182052	174619	0	0.656545	99.43602	177.3121
22	0.172144	-45.459	0	0.162707	0.182009	174611.9	0	0.656322	99.43585	177.2585
23	0.172199	-45.4391	0	0.162759	0.182069	174628.6	0	0.656589	99.4361	177.3373
24	0.172042	-45.5098	0	0.162616	0.181895	174446.5	0	0.655386	99.43477	176.918
25	0.171957	-45.5687	0	0.162543	0.181798	174161.2	0	0.654132	99.43345	176.5054
26	0.171982	-45.5547	0	0.162565	0.181826	174591.6	0	0.654474	99.43433	176.7824
27	0.172076	-45.4912	0	0.162647	0.181934	174582.2	0	0.655757	99.43535	177.0999
28	0.171808	-45.7107	0	0.162423	0.181617	174474.9	0	0.650855	99.43116	175.7965
29	0.172242	-45.4338	0	0.1628	0.182113	174641.5	0	0.656584	99.43618	177.3602
30	0.172595	-45.4635	0	0.163158	0.182459	174628.2	0	0.653473	99.43347	176.5135

31	0.172257	-45.4229	0		0.162813	0.18213	174634.5	0		0.656705	99.43616	177.3541
32	0.172416	-45.4144	0		0.162969	0.18229	174643.9	0		0.656003	99.43558	177.1737
33	0.172295	-45.4157	0		0.16285	0.18217	174637.8	0		0.65668	99.43607	177.3258
34	0.172322	-45.4119	0		0.162876	0.182198	174639.1	0		0.656616	99.43585	177.2571
35	0.172344	-45.4095	0		0.162897	0.18222	174631.1	0		0.656539	99.43395	176.6619
36	0.172316	-45.412	0		0.162869	0.182191	174633.4	0		0.656645	99.43563	177.1897
37	0.172727	-45.5301	0		0.163303	0.182575	173928.7	0		0.650605	99.42949	175.2809
38	0.17265	-45.4843	0		0.163217	0.182509	174389.2	0		0.652441	99.43173	175.9739
39	0.17217	-45.4467	0		0.162731	0.182038	174584.1	0		0.656503	99.43581	177.246
40	0.172183	-45.4427	0		0.162743	0.182052	174608.5	0		0.656553	99.43596	177.2937
41	0.17232	-45.4183	0		0.162875	0.182194	174644	0		0.65653	99.43611	177.3398
42	0.172196	-45.4372	0		0.162755	0.182066	174576	0		0.656622	99.43575	177.2274
43	0.17248	-45.4238	0		0.163035	0.182352	174638.4	0		0.655328	99.43499	176.9883
44	0.172065	-45.4977	0		0.162636	0.181921	174571.8	0		0.655633	99.43523	177.0623
45	0.172351	-45.411	0		0.162904	0.182227	174643.8	0		0.656478	99.43588	177.2661
46	0.172358	-45.4107	0		0.162911	0.182233	174644.1	0		0.656445	99.43584	177.2539
47	0.172252	-45.4218	0		0.162808	0.182125	174593.2	0		0.656738	99.43559	177.1749
48	0.172504	-45.4268	0		0.16306	0.182376	174552.3	0		0.655043	99.43328	176.4542
49	0.172008	-45.5366	0		0.162587	0.181856	174597.9	0		0.654861	99.43467	176.8862
50	0.172269	-45.4211	0		0.162825	0.182143	174637.6	0		0.656695	99.43617	177.359
51	0.172362	-45.4129	0		0.162916	0.182238	174644.6	0		0.656385	99.43593	177.2836
52	0.172018	-45.5254	0		0.162595	0.181868	174477.1	0		0.655069	99.43461	176.8682
53	0.172274	-45.4208	0		0.16283	0.182148	174639.3	0		0.656683	99.43618	177.3625
54	0.172072	-45.4925	0		0.162642	0.18193	174536.1	0		0.655727	99.43521	177.0555
55	0.172137	-45.4678	0		0.162702	0.182	174631.9	0		0.656187	99.43583	177.2513
56	0.172294	-45.4156	0		0.162848	0.182168	174636.4	0		0.656687	99.43604	177.3166
57	0.171897	-45.6237	0		0.162495	0.181726	174559.5	0		0.652924	99.43299	176.364
58	0.172097	-45.4833	0		0.162665	0.181956	174617.3	0		0.655914	99.43557	177.1683
59	0.172516	-45.4343	0		0.163074	0.182387	174636.3	0		0.65481	99.4346	176.8653
60	0.172264	-45.4235	0		0.16282	0.182137	174639.4	0		0.656676	99.4362	177.3677
61	0.17222	-45.4294	0		0.162777	0.182091	174452.1	0		0.656701	99.43449	176.8309
62	0.172262	-45.4216	0		0.162818	0.182136	174634	0		0.65671	99.43614	177.3483
63	0.172104	-45.4772	0		0.162671	0.181965	174597.3	0		0.656016	99.43558	177.174
64	0.172297	-45.4156	0		0.162851	0.182171	174638.6	0		0.656674	99.43608	177.3302

65	0.172331	-45.4111	0	0.162884	0.182206	174639.8	0		0.656586	99.43575	177.2277
66	0.172223	-45.4295	0	0.16278	0.182095	174608.6	0		0.656692	99.43595	177.2901
67	0.171737	-45.8043	0	0.162371	0.181526	174587	0		0.648694	99.42937	175.2459
68	0.172232	-45.4352	0	0.16279	0.182102	174640.4	0		0.656589	99.43617	177.3596
69	0.172345	-45.4096	0	0.162898	0.182221	174635.1	0		0.656533	99.4345	176.8347
70	0.171796	-45.7206	0	0.162413	0.181604	174035.6	0		0.650591	99.43061	175.6267
71	0.172375	-45.4098	0	0.162927	0.182251	174644.6	0		0.656355	99.43549	177.1447
72	0.172437	-45.4157	0	0.162991	0.182312	174642	0		0.655815	99.43535	177.1014
73	0.172031	-45.5169	0	0.162606	0.181883	174471.1	0		0.655243	99.43472	176.9036
74	0.171932	-45.5911	0	0.162522	0.181767	174487.5	0		0.653646	99.43351	176.5252
75	0.172235	-45.4299	0	0.162792	0.182106	174635.9	0		0.656662	99.43618	177.3629
76	0.172383	-45.4091	0	0.162936	0.182259	174644.5	0		0.656311	99.35772	155.6953
77	0.172604	-45.4665	0	0.163167	0.182467	174624.9	0		0.653324	99.43333	176.47
78	0.171865	-45.6498	0	0.162468	0.181688	174267.4	0		0.652282	99.43217	176.109
79	0.17241	-45.4118	0	0.162963	0.182286	174643.5	0		0.656083	99.43539	177.1141
80	0.17203	-45.5182	0	0.162605	0.181882	174527.6	0		0.65522	99.43482	176.9342
81	0.172303	-45.4152	0	0.162857	0.182178	174640.4	0		0.656653	99.4361	177.3354
82	0.17218	-45.4424	0	0.16274	0.182048	174511.5	0		0.656561	99.43538	177.1116
83	0.172045	-45.5083	0	0.162619	0.181899	174528.6	0		0.655419	99.43497	176.9812
84	0.172182	-45.4427	0	0.162742	0.182051	174602.5	0		0.656554	99.43593	177.2828
85	0.17218	-45.4496	0	0.162741	0.182047	174634.8	0		0.656452	99.43605	177.3199
86	0.17197	-45.5592	0	0.162554	0.181813	174409.8	0		0.654346	99.43396	176.6649
87	0.172216	-45.4347	0	0.162774	0.182087	174633.4	0		0.656628	99.43615	177.3532
88	0.17246	-45.4214	0	0.163015	0.182333	174641.7	0		0.655541	99.43522	177.0611
89	0.172246	-45.4233	0	0.162802	0.182119	174597.9	0		0.656732	99.43571	177.2146
90	0.1719	-45.6196	0	0.162497	0.18173	174537.3	0		0.653008	99.43304	176.3786
91	0.172158	-45.4512	0	0.162719	0.182024	174561.7	0		0.656439	99.43568	177.2053
92	0.171938	-45.5848	0	0.162527	0.181775	174386.7	0		0.653778	99.43349	176.5186
93	0.172096	-45.4788	0	0.162663	0.181956	174365.2	0		0.65598	99.43485	176.9433
94	0.172283	-45.4157	0	0.162837	0.182157	174566.5	0		0.656728	99.43438	176.797
95	0.171974	-45.5587	0	0.162558	0.181817	174562.4	0		0.654375	99.43421	176.743
96	0.172026	-45.5188	0	0.162602	0.181878	174114.2	0		0.655199	99.43398	176.6718
97	0.172146	-45.4562	0	0.162708	0.182011	174561.3	0		0.656364	99.43566	177.1975
98	0.172124	-45.4728	0	0.16269	0.181985	174629.1	0		0.656103	99.43575	177.2271

99	0.172274	-45.4176	0	0.162828	0.182148	174611.2	0	0.656732	99.43563	177.1888
100	0.172366	-45.4144	0	0.162919	0.182241	174644.7	0	0.656341	99.43593	177.2824
101	0.172309	-45.4131	0	0.162863	0.182184	174635.9	0	0.656659	99.43586	177.2623
102	0.172509	-45.4343	0	0.163066	0.182379	174639.1	0	0.654886	99.4347	176.8957
103	0.172268	-45.4198	0	0.162823	0.182141	174630.1	0	0.656721	99.43605	177.322
104	0.172018	-45.5275	0	0.162595	0.181868	174572.4	0	0.655036	99.43476	176.9164
105	0.172282	-45.4181	0	0.162837	0.182156	174637.3	0	0.656695	99.43613	177.3445
106	0.172202	-45.4363	0	0.162761	0.182072	174613.9	0	0.656627	99.43602	177.3104
107	0.172322	-45.4119	0	0.162876	0.182198	174639.1	0	0.656616	99.43585	177.2571
108	0.172248	-45.4363	0	0.162806	0.182118	174642.6	0	0.656531	99.43614	177.3499
109	0.172234	-45.4329	0	0.162792	0.182105	174639.4	0	0.656618	99.43619	177.3637
110	0.172121	-45.4717	0	0.162687	0.181983	174622.8	0	0.656118	99.43574	177.2243
111	0.172595	-45.4635	0	0.163158	0.182459	174628.2	0	0.653473	99.43347	176.5135
112	0.172393	-45.4131	0	0.162947	0.182268	174644.6	0	0.656184	99.43575	177.225
113	0.17244	-45.4164	0	0.162994	0.182314	174642	0	0.655783	99.43535	177.1002
114	0.172518	-45.4371	0	0.163076	0.182388	174638.6	0	0.654751	99.43458	176.8604
115	0.171949	-45.5808	0	0.162538	0.181788	174590.6	0	0.653909	99.43386	176.634
116	0.17213	-45.4625	0	0.162694	0.181994	174519.3	0	0.656263	99.43545	177.132
117	0.172216	-45.4375	0	0.162775	0.182086	174637.7	0	0.656587	99.43616	177.3547
118	0.17214	-45.4632	0	0.162704	0.182004	174625.6	0	0.656257	99.43586	177.2611
119	0.172253	-45.4283	0	0.16281	0.182125	174640.7	0	0.656637	99.4362	177.3683
120	0.17226	-45.4246	0	0.162816	0.182133	174639.5	0	0.656671	99.4362	177.3686
121	0.17253	-45.4403	0	0.163089	0.182399	174637.2	0	0.654573	99.43443	176.8114
122	0.171947	-45.5803	0	0.162535	0.181785	174553.1	0	0.653899	99.4338	176.6161
123	0.172222	-45.4325	0	0.16278	0.182093	174632.2	0	0.656651	99.43615	177.3534
124	0.172424	-45.4193	0	0.162979	0.182298	174644	0	0.655865	99.43554	177.1614
125	0.17204	-45.5151	0	0.162615	0.181892	174600.4	0	0.655299	99.43503	177.0003
126	0.172175	-45.445	0	0.162735	0.182043	174587.4	0	0.656525	99.43584	177.2533
127	0.172193	-45.4418	0	0.162753	0.182062	174630.1	0	0.656556	99.43609	177.3344
128	0.172505	-45.4267	0	0.16306	0.182377	174458.4	0	0.655042	99.43163	175.9422
129	0.172346	-45.4138	0	0.162899	0.182221	174644.3	0	0.656466	99.43602	177.31
130	0.172297	-45.4143	0	0.162851	0.182172	174629.4	0	0.656693	99.43578	177.2348
131	0.172441	-45.4169	0	0.162995	0.182315	174642.3	0	0.65577	99.43537	177.1054
132	0.172476	-45.4282	0	0.163032	0.182348	174642	0	0.655292	99.43506	177.0092

133	0.172161	-45.4501	0	0.162722	0.182028	174568.9	0	0.656455	99.43572	177.2176
134	0.17213	-45.4683	0	0.162695	0.181993	174626.2	0	0.656175	99.4358	177.242
135	0.172339	-45.4131	0	0.162892	0.182214	174643.8	0	0.656514	99.43603	177.3137
136	0.172419	-45.412	0	0.162972	0.182295	174640.7	0	0.65601	99.43496	176.9782
137	0.172334	-45.4149	0	0.162887	0.182208	174644	0	0.656514	99.43607	177.3261
138	0.172081	-45.5002	0	0.162653	0.181937	174631.9	0	0.655631	99.43537	177.108
139	0.172169	-45.4496	0	0.162731	0.182036	174623.3	0	0.656459	99.436	177.3034
140	0.172273	-45.4175	0	0.162827	0.182147	174573.4	0	0.656738	99.43487	176.9496
141	0.172149	-45.4551	0	0.162712	0.182015	174583.4	0	0.65638	99.43576	177.2305
142	0.172322	-45.4129	0	0.162875	0.182197	174641.6	0	0.656605	99.43601	177.3095
143	0.17251	-45.433	0	0.163067	0.18238	174637.8	0	0.654899	99.43469	176.8925
144	0.172485	-45.4222	0	0.16304	0.182358	174588.9	0	0.655303	99.43373	176.5948
145	0.172399	-45.4188	0	0.162954	0.182273	174644.5	0	0.656058	99.43572	177.2165
146	0.172181	-45.4433	0	0.162741	0.182049	174606.8	0	0.656545	99.43595	177.2899
147	0.172286	-45.4161	0	0.16284	0.18216	174629.6	0	0.65671	99.43591	177.2778
148	0.172412	-45.4116	0	0.162965	0.182287	174642.8	0	0.656074	99.43521	177.058
149	0.172559	-45.472	0	0.163124	0.182421	174639.9	0	0.653776	99.4338	176.6151
150	0.172339	-45.4122	0	0.162892	0.182214	174643.5	0	0.656528	99.43599	177.3019
151	0.172383	-45.4137	0	0.162937	0.182258	174644.7	0	0.656241	99.43583	177.2501
152	0.172033	-45.5195	0	0.162608	0.181884	174597.7	0	0.655211	99.43495	176.9759
153	0.172261	-45.4196	0	0.162816	0.182134	174438.5	0	0.656746	99.43288	176.3303
154	0.172557	-45.4438	0	0.163116	0.182425	174600.3	0	0.654233	99.43384	176.6272
155	0.172152	-45.4622	0	0.162716	0.182016	174634.5	0	0.656273	99.43591	177.2761
156	0.172094	-45.4799	0	0.162661	0.181954	174387.2	0	0.655961	99.43491	176.9627
157	0.172338	-45.4114	0	0.162892	0.182214	174642.8	0	0.656541	99.43592	177.2805
158	0.172143	-45.4576	0	0.162707	0.182009	174582.6	0	0.656343	99.43574	177.2235
159	0.172298	-45.416	0	0.162853	0.182173	174640.1	0	0.656662	99.43612	177.3412
160	0.17248	-45.4216	0	0.163035	0.182353	174623.6	0	0.655357	99.4346	176.8668
161	0.17212	-45.4687	0	0.162686	0.181983	174595.3	0	0.656163	99.43568	177.2046
162	0.17253	-45.4368	0	0.163088	0.1824	174630.2	0	0.654627	99.43439	176.7994
163	0.172242	-45.4274	0	0.162799	0.182114	174635.6	0	0.656681	99.43618	177.3627
164	0.172348	-45.4128	0	0.162901	0.182223	174644.2	0	0.656469	99.43599	177.3023
165	0.172088	-45.4868	0	0.162657	0.181946	174606.4	0	0.655846	99.43548	177.1418
166	0.172412	-45.4114	0	0.162965	0.182288	174641.9	0	0.656073	99.43496	176.9798

167	0.171755	-45.7748	0	0.162383	0.181551	174545.2	0	0.64934	99.42991	175.4102
168	0.172422	-45.4236	0	0.162978	0.182295	174644.1	0	0.655814	99.43552	177.1553
169	0.172493	-45.4266	0	0.163049	0.182365	174636.2	0	0.655158	99.43484	176.9401
170	0.172059	-45.4989	0	0.162631	0.181915	174405.2	0	0.6556	99.4348	176.9289
171	0.172324	-45.4107	0	0.162877	0.182199	174580.1	0	0.656627	99.43168	175.9572
172	0.172609	-45.4646	0	0.163172	0.182473	174534.1	0	0.653282	99.43284	176.3154
173	0.172522	-45.4345	0	0.16308	0.182393	174632	0	0.654745	99.43449	176.8323
174	0.172252	-45.4218	0	0.162808	0.182125	174593.2	0	0.656738	99.43559	177.1749
175	0.172431	-45.4419	0	0.16299	0.182299	174643.9	0	0.655474	99.43526	177.0714
176	0.172342	-45.4119	0	0.162896	0.182218	174643.6	0	0.656514	99.43597	177.2947
177	0.172322	-45.4124	0	0.162876	0.182197	174640.9	0	0.656608	99.43597	177.2941
178	0.172229	-45.4293	0	0.162786	0.182101	174628.1	0	0.656683	99.43613	177.3444
179	0.172282	-45.4183	0	0.162836	0.182156	174637.7	0	0.656694	99.43613	177.3471
180	0.172556	-45.4508	0	0.163117	0.182423	174636.4	0	0.654133	99.43407	176.6987
181	0.172284	-45.4197	0	0.162839	0.182158	174640.8	0	0.656664	99.43618	177.3621
182	0.172038	-45.5126	0	0.162613	0.181891	174501.9	0	0.655333	99.43485	176.9435
183	0.172271	-45.4207	0	0.162827	0.182145	174637.9	0	0.656694	99.43617	177.3586
184	0.172062	-45.4974	0	0.162634	0.181919	174455.8	0	0.655631	99.43494	176.9729
185	0.172223	-45.4288	0	0.16278	0.182094	174518.8	0	0.656705	99.43508	177.0172
186	0.172156	-45.4549	0	0.162718	0.182021	174620.7	0	0.656384	99.43593	177.2837
187	0.172343	-45.4163	0	0.162897	0.182218	174644.5	0	0.656441	99.43603	177.3148
188	0.172192	-45.4388	0	0.162751	0.182061	174585.9	0	0.656602	99.43583	177.2509
189	0.172464	-45.4194	0	0.163019	0.182338	174636.7	0	0.655531	99.43503	176.9994
190	0.172243	-45.4237	0	0.162799	0.182115	174544.1	0	0.656735	99.43506	177.0088
191	0.171945	-45.5809	0	0.162533	0.181783	174530.7	0	0.65388	99.43376	176.6021
192	0.172305	-45.4168	0	0.162859	0.182179	174642.3	0	0.656623	99.43615	177.3507
193	0.172101	-45.4808	0	0.162669	0.181962	174617.2	0	0.655959	99.4356	177.1794
194	0.172229	-45.4271	0	0.162786	0.182101	174540.2	0	0.656717	99.43521	177.058
195	0.1722	-45.4458	0	0.16276	0.182068	174639.1	0	0.656488	99.4361	177.3348
196	0.172152	-45.4622	0	0.162716	0.182016	174634.5	0	0.656273	99.43591	177.2761
197	0.172498	-45.4264	0	0.163053	0.18237	174628.4	0	0.655115	99.43466	176.8846
198	0.171935	-45.5878	0	0.162525	0.181772	174473.5	0	0.653717	99.43355	176.5377
199	0.172413	-45.4402	0	0.162971	0.182282	174644.2	0	0.655638	99.4354	177.1155
200	0.172112	-45.4758	0	0.162679	0.181973	174620.6	0	0.656047	99.43568	177.2046

201	0.173104	-45.9207	0	0.163757	0.182867	169633.1	0	0.636886	99.41629	171.3177
202	0.172015	-45.5272	0	0.162592	0.181865	174443.2	0	0.65503	99.43452	176.8396
203	0.172239	-45.4244	0	0.162795	0.182111	174147.2	0	0.656735	99.43036	175.5495
204	0.172323	-45.4127	0	0.162877	0.182198	174641.6	0	0.6566	99.436	177.3052
205	0.172206	-45.4533	0	0.162768	0.182073	174642.1	0	0.656365	99.43601	177.3084
206	0.17247	-45.4328	0	0.163027	0.18234	174642.9	0	0.655282	99.43507	177.0142
207	0.172417	-45.416	0	0.162971	0.182291	174644.1	0	0.655971	99.4356	177.1791
208	0.172189	-45.44	0	0.162749	0.182059	174598.7	0	0.656588	99.43591	177.2773
209	0.172325	-45.4169	0	0.16288	0.1822	174644	0	0.656526	99.4361	177.3357
210	0.1724	-45.4118	0	0.162953	0.182276	174644.3	0	0.656154	99.4356	177.1806
211	0.172343	-45.4163	0	0.162897	0.182218	174644.5	0	0.656441	99.43603	177.3148
212	0.172184	-45.4417	0	0.162744	0.182053	174588.3	0	0.656568	99.43585	177.2566
213	0.172081	-45.4865	0	0.16265	0.18194	174189	0	0.655837	99.43432	176.7781
214	0.17194	-45.584	0	0.162529	0.181777	174477.4	0	0.653802	99.43362	176.5607
215	0.171895	-45.6231	0	0.162492	0.181724	174487.8	0	0.652918	99.43291	176.3387
216	0.172275	-45.4426	0	0.162835	0.182144	174644.2	0	0.656351	99.43601	177.3064
217	0.172163	-45.4492	0	0.162724	0.18203	174563.3	0	0.656468	99.4357	177.2096
218	0.172517	-45.4379	0	0.163075	0.182386	174639.4	0	0.654751	99.43459	176.8639
219	0.172411	-45.4119	0	0.162964	0.182286	174643.5	0	0.656076	99.43541	177.1186
220	0.172288	-45.423	0	0.162844	0.182161	174643	0	0.656598	99.43618	177.3604
221	0.172327	-45.4117	0	0.162881	0.182203	174640.6	0	0.656595	99.43588	177.2665
222	0.172289	-45.4167	0	0.162843	0.182163	174637.1	0	0.656691	99.43609	177.333
223	0.172256	-45.4206	0	0.162811	0.182129	174495.5	0	0.656745	99.43402	176.6842
224	0.172189	-45.4393	0	0.162749	0.182059	174559.8	0	0.656599	99.43566	177.1975
225	0.171775	-45.7461	0	0.162398	0.181577	174400.4	0	0.649991	99.43037	175.5528
226	0.172263	-45.4214	0	0.162819	0.182137	174634	0	0.656711	99.43614	177.3474
227	0.17238	-45.4092	0	0.162933	0.182256	174644.6	0	0.656327	99.43374	176.5983
228	0.172143	-45.4617	0	0.162706	0.182007	174624.6	0	0.656281	99.43588	177.2656
229	0.172537	-45.4374	0	0.163095	0.182407	174615	0	0.654545	99.43416	176.7295
230	0.172171	-45.4501	0	0.162733	0.182038	174628.9	0	0.656451	99.43602	177.3105
231	0.172012	-45.5311	0	0.16259	0.181861	174564	0	0.65496	99.43469	176.8925
232	0.172163	-45.4565	0	0.162726	0.182029	174634.4	0	0.656357	99.43597	177.2966
233	0.172719	-45.5262	0	0.163294	0.182568	174513.8	0	0.650791	99.43094	175.7274
234	0.172274	-45.4221	0	0.16283	0.182147	174640.8	0	0.656663	99.4362	177.367

235	0.172387	-45.4097	0	0.162939	0.182262	174644.7	0	0.656279	99.43499	176.9868
236	0.172262	-45.4216	0	0.162818	0.182136	174634	0	0.65671	99.43614	177.3483
237	0.172305	-45.4186	0	0.16286	0.182179	174643.2	0	0.656594	99.43615	177.3531
238	0.172106	-45.4736	0	0.162672	0.181967	174342.4	0	0.656073	99.43479	176.9247
239	0.17218	-45.443	0	0.16274	0.182049	174589.7	0	0.656551	99.43585	177.2588
240	0.172379	-45.409	0	0.162932	0.182255	174641.7	0	0.656339	99.40306	167.52
241	0.172211	-45.4324	0	0.162769	0.182082	174575	0	0.656672	99.4357	177.2091
242	0.172141	-45.4584	0	0.162704	0.182006	174564.8	0	0.65633	99.43566	177.1979
243	0.172151	-45.4553	0	0.162714	0.182017	174608.9	0	0.656378	99.43588	177.2658
244	0.172468	-45.42	0	0.163023	0.182342	174635.8	0	0.655487	99.43499	176.9864
245	0.172478	-45.4332	0	0.163035	0.182349	174642.5	0	0.6552	99.435	176.9912
246	0.172199	-45.4389	0	0.162758	0.182068	174626.9	0	0.656593	99.43609	177.3346
247	0.172289	-45.4175	0	0.162844	0.182163	174639.5	0	0.656677	99.43614	177.3494
248	0.171924	-45.5967	0	0.162516	0.181759	174448.5	0	0.653516	99.43336	176.4774
249	0.172111	-45.4743	0	0.162678	0.181973	174607.5	0	0.656068	99.43565	177.1963
250	0.172307	-45.415	0	0.162861	0.182182	174641.1	0	0.656639	99.4361	177.3357
251	0.172162	-45.4494	0	0.162723	0.182029	174558.3	0	0.656464	99.43567	177.2015
252	0.172463	-45.4178	0	0.163017	0.182337	174598.3	0	0.655565	99.43348	176.5158
253	0.172017	-45.5256	0	0.162595	0.181868	174458.4	0	0.655065	99.43457	176.8563
254	0.172344	-45.41	0	0.162897	0.18222	174640.6	0	0.656531	99.43548	177.1404
255	0.172143	-45.4576	0	0.162706	0.182008	174577.9	0	0.656342	99.43572	177.217
256	0.17194	-45.5824	0	0.162529	0.181777	173989	0	0.653826	99.43301	176.3703
257	0.17198	-45.5527	0	0.162563	0.181825	174519	0	0.654496	99.43424	176.7529
258	0.172223	-45.4365	0	0.162782	0.182093	174639.2	0	0.656587	99.43617	177.3574
259	0.17186	-45.6564	0	0.162464	0.181681	174480	0	0.652143	99.43225	176.1352
260	0.172061	-45.4978	0	0.162632	0.181917	174188	0	0.655621	99.43428	176.7671
261	0.171977	-45.5533	0	0.16256	0.181821	173790.6	0	0.654468	99.4331	176.397
262	0.172127	-45.4678	0	0.162692	0.18199	174618.6	0	0.656181	99.43578	177.2348
263	0.171759	-45.7675	0	0.162385	0.181556	174487.8	0	0.649488	99.43	175.4381
264	0.172161	-45.453	0	0.162723	0.182028	174623.5	0	0.656411	99.43596	177.2937
265	0.172245	-45.4257	0	0.162801	0.182117	174632.9	0	0.656698	99.43616	177.3546
266	0.172163	-45.4518	0	0.162725	0.18203	174622.2	0	0.656428	99.43597	177.2954
267	0.172214	-45.4344	0	0.162772	0.182084	174629.9	0	0.656636	99.43613	177.3468
268	0.172218	-45.4325	0	0.162776	0.182089	174627	0	0.656658	99.43612	177.3418

269	0.171926	-45.5971	0	0.162518	0.181761	174549.5	0		0.653524	99.43348	176.5173
270	0.172444	-45.4227	0	0.162999	0.182317	174643.4	0		0.655652	99.43537	177.1063
271	0.172244	-45.4249	0	0.1628	0.182117	174626.1	0		0.656713	99.43608	177.3311
272	0.172289	-45.4157	0	0.162843	0.182164	174631.2	0		0.656704	99.43593	177.282
273	0.172114	-45.4709	0	0.162679	0.181976	174561.9	0		0.656122	99.43554	177.1599
274	0.172509	-45.4306	0	0.163066	0.18238	174633.1	0		0.654936	99.43464	176.8782
275	0.172184	-45.4439	0	0.162744	0.182052	174623.6	0		0.656535	99.43604	177.3188
276	0.172406	-45.4128	0	0.162959	0.182281	174644.2	0		0.656101	99.43562	177.1854
277	0.171787	-45.7306	0	0.162407	0.181593	174101.3	0		0.650352	99.43046	175.5812
278	0.172319	-45.4127	0	0.162872	0.182194	174640.4	0		0.656621	99.43598	177.2975
279	0.172264	-45.4223	0	0.16282	0.182137	174637.5	0		0.656694	99.43618	177.3617
280	0.172215	-45.433	0	0.162773	0.182086	174624.1	0		0.656656	99.4361	177.3347
281	0.171761	-45.7658	0	0.162388	0.181559	174524	0		0.649545	99.43007	175.4604
282	0.172318	-45.4136	0	0.162872	0.182193	174641.8	0		0.656611	99.43606	177.3225
283	0.172477	-45.4236	0	0.163032	0.182349	174639.3	0		0.655357	99.43503	177.0017
284	0.172506	-45.4273	0	0.163062	0.182378	174547.1	0		0.655015	99.43322	176.4355
285	0.172486	-45.4223	0	0.163041	0.182359	174571.1	0		0.655291	99.43331	176.4633
286	0.171908	-45.6122	0	0.162503	0.181739	174505.4	0		0.65317	99.43314	176.4097
287	0.172322	-45.4127	0	0.162875	0.182197	174641.3	0		0.656607	99.43599	177.303
288	0.172498	-45.4256	0	0.163054	0.18237	174608.8	0		0.655124	99.43428	176.7659
289	0.172319	-45.4126	0	0.162872	0.182194	174639.7	0		0.656625	99.43594	177.2863
290	0.171997	-45.5405	0	0.162577	0.181844	174516.4	0		0.654755	99.43444	176.8155
291	0.172449	-45.4152	0	0.163003	0.182324	174509.4	0		0.655723	99.42744	174.6554
292	0.172504	-45.4266	0	0.16306	0.182376	174461.4	0		0.655051	99.43166	175.9502
293	0.172184	-45.4439	0	0.162744	0.182052	174623.6	0		0.656535	99.43604	177.3188
294	0.172396	-45.4119	0	0.162949	0.182271	174644.5	0		0.656184	99.43567	177.2014
295	0.172235	-45.4257	0	0.162791	0.182107	174561.5	0		0.656724	99.43538	177.1101
296	0.17225	-45.4236	0	0.162805	0.182122	174627.1	0		0.656718	99.43608	177.3301
297	0.17223	-45.4304	0	0.162787	0.182101	174634	0		0.656664	99.43617	177.3587
298	0.172234	-45.4275	0	0.162791	0.182106	174625.4	0		0.656699	99.4361	177.3347
299	0.172499	-45.426	0	0.163055	0.182371	174616.6	0		0.65511	99.43443	176.8128
300	0.172439	-45.4172	0	0.162993	0.182313	174642.6	0		0.655777	99.43539	177.1139
301	0.172348	-45.4099	0	0.162901	0.182224	174641.7	0		0.656511	99.43551	177.1524
302	0.172273	-45.418	0	0.162827	0.182147	174619.1	0		0.656729	99.43581	177.2442

303	0.172341	-45.4103	0		0.162894	0.182217	174640.7	0		0.656544	99.4356	177.1784
304	0.172565	-45.4461	0		0.163125	0.182433	174537.3	0		0.6541	99.43326	176.4472
305	0.172378	-45.4162	0		0.162933	0.182253	174644.7	0		0.656235	99.43586	177.2601
306	0.172356	-45.4153	0		0.16291	0.182231	174644.6	0		0.656386	99.43598	177.2978
307	0.172126	-45.4633	0		0.162691	0.18199	172198.4	0		0.656248	99.42661	174.4017
308	0.172319	-45.4112	0		0.162872	0.182195	174614	0		0.656641	99.43436	176.7921
309	0.172223	-45.4365	0		0.162782	0.182093	174639.2	0		0.656587	99.43617	177.3574
310	0.172209	-45.4358	0		0.162768	0.182079	174629.2	0		0.656624	99.43612	177.3437
311	0.172201	-45.4361	0		0.16276	0.182071	174603.8	0		0.656631	99.43595	177.2879
312	0.172506	-45.4327	0		0.163063	0.182377	174638.8	0		0.654939	99.43473	176.9067
313	0.171957	-45.5681	0		0.162543	0.181798	173801.4	0		0.654144	99.43295	176.3513
314	0.172303	-45.4153	0		0.162857	0.182177	174640.2	0		0.656655	99.4361	177.3349
315	0.172314	-45.4116	0		0.162868	0.18219	174599.7	0		0.656658	99.43389	176.6447
316	0.172581	-45.454	0		0.163142	0.182447	174612.7	0		0.653793	99.43363	176.5625
317	0.172312	-45.4191	0		0.162867	0.182185	174643.7	0		0.656557	99.43613	177.3472
318	0.172508	-45.43	0		0.163064	0.182379	174632.8	0		0.654964	99.43465	176.8825
319	0.172223	-45.4326	0		0.162781	0.182094	174633.6	0		0.656647	99.43616	177.3562
320	0.17239	-45.4173	0		0.162944	0.182264	174644.6	0		0.656144	99.43579	177.2378
321	0.172027	-45.5195	0		0.162603	0.181879	174515.1	0		0.655192	99.43477	176.9198
322	0.172383	-45.4134	0		0.162936	0.182258	174644.7	0		0.656249	99.43583	177.25
323	0.172197	-45.4388	0		0.162756	0.182067	174623	0		0.656596	99.43607	177.327
324	0.172557	-45.4446	0		0.163116	0.182425	174616.8	0		0.65422	99.43397	176.6685
325	0.172587	-45.4648	0		0.163151	0.182451	174635	0		0.653553	99.43358	176.5462
326	0.172304	-45.4127	0		0.162857	0.182179	174579.9	0		0.656688	99.4337	176.5852
327	0.172338	-45.4096	0		0.162891	0.182214	174498.4	0		0.656569	99.41845	171.9545
328	0.172327	-45.4103	0		0.16288	0.182203	174197	0		0.656615	99.40209	167.2487
329	0.17188	-45.6411	0		0.16248	0.181704	174568.1	0		0.65253	99.43267	176.2627
330	0.172363	-45.4299	0		0.16292	0.182234	174644.7	0		0.656126	99.43581	177.2445
331	0.172533	-45.4384	0		0.163091	0.182402	174632.4	0		0.654574	99.43437	176.795
332	0.172019	-45.5274	0		0.162596	0.181869	174580.5	0		0.655043	99.43478	176.9228
333	0.172193	-45.4534	0		0.162756	0.18206	174640.8	0		0.656381	99.43602	177.3109
334	0.172499	-45.4289	0		0.163055	0.18237	174637.3	0		0.655068	99.4348	176.928
335	0.172347	-45.4197	0		0.162902	0.182221	174644.6	0		0.656369	99.43599	177.3027
336	0.171722	-45.8101	0		0.162357	0.18151	174294.7	0		0.648419	99.42896	175.1197

337	0.172288	-45.4264	0	0.162844	0.18216	174643.6	0	0.656549	99.43615	177.3525
338	0.172331	-45.4178	0	0.162886	0.182205	174644.3	0	0.656482	99.43607	177.328
339	0.17216	-45.451	0	0.162721	0.182026	174592.3	0	0.656442	99.43583	177.2524
340	0.172236	-45.4273	0	0.162792	0.182107	174626.7	0	0.656699	99.43611	177.3385
341	0.17225	-45.4232	0	0.162806	0.182123	174624.2	0	0.656722	99.43604	177.3175
342	0.172298	-45.4153	0	0.162852	0.182172	174638	0	0.656676	99.43606	177.3235
343	0.172093	-45.4922	0	0.162664	0.181951	174632	0	0.655775	99.43549	177.1456
344	0.172027	-45.5192	0	0.162603	0.181879	174463.4	0	0.655196	99.43467	176.8883
345	0.172211	-45.4322	0	0.162769	0.182082	174552.5	0	0.656674	99.43551	177.1497
346	0.171781	-45.7366	0	0.162401	0.181585	165701.2	0	0.650196	99.42446	173.7512
347	0.172199	-45.4378	0	0.162758	0.182069	174620.2	0	0.656608	99.43606	177.3227
348	0.172003	-45.5343	0	0.162582	0.181851	173735.6	0	0.654876	99.43316	176.4173
349	0.172073	-45.4906	0	0.162643	0.181931	174097.2	0	0.65576	99.43407	176.7007
350	0.172073	-45.4904	0	0.162643	0.181931	174040.4	0	0.655763	99.43392	176.654
351	0.172196	-45.4366	0	0.162755	0.182066	174485.2	0	0.656631	99.4351	177.0222
352	0.172363	-45.4092	0	0.162916	0.182239	174642.6	0	0.656437	99.4348	176.9299
353	0.171965	-45.562	0	0.16255	0.181808	173863.9	0	0.654279	99.43311	176.4017
354	0.172057	-45.5016	0	0.162629	0.181913	174555.9	0	0.655554	99.43513	177.0318
355	0.17204	-45.5104	0	0.162614	0.181893	174126.6	0	0.65537	99.43407	176.7001
356	0.172408	-45.4122	0	0.162961	0.182283	174643.9	0	0.656092	99.43553	177.1573
357	0.172324	-45.4116	0	0.162877	0.182199	174638.2	0	0.656614	99.43576	177.2308
358	0.17235	-45.4136	0	0.162904	0.182225	174644.4	0	0.656443	99.436	177.3036
359	0.172465	-45.4279	0	0.163021	0.182337	174642.7	0	0.655396	99.43516	177.0406
360	0.172615	-45.467	0	0.163179	0.182478	174442.6	0	0.653168	99.43232	176.156
361	0.172256	-45.4295	0	0.162813	0.182127	174641.7	0	0.656611	99.43619	177.3654
362	0.172153	-45.4522	0	0.162715	0.18202	174342.4	0	0.656425	99.43464	176.877
363	0.172329	-45.412	0	0.162882	0.182204	174641.9	0	0.656582	99.43596	177.2918
364	0.172168	-45.4479	0	0.162729	0.182035	174594.6	0	0.656485	99.43586	177.2617
365	0.172324	-45.4255	0	0.16288	0.182196	174644.5	0	0.656405	99.43604	177.3165
366	0.172375	-45.412	0	0.162929	0.182251	174644.7	0	0.656318	99.43584	177.2539
367	0.172181	-45.4524	0	0.162742	0.182047	174637.8	0	0.656409	99.43603	177.3139
368	0.172388	-45.4172	0	0.162942	0.182262	174644.6	0	0.656157	99.4358	177.2412
369	0.172468	-45.4194	0	0.163022	0.182341	174630.8	0	0.6555	99.43481	176.932
370	0.171954	-45.5708	0	0.162541	0.181795	174186.9	0	0.654086	99.43345	176.5067

371	0.172366	-45.4146	0	0.16292	0.182241	174644.7	0	0.656336	99.43593	177.282
372	0.172274	-45.4198	0	0.162829	0.182148	174637.3	0	0.656698	99.43615	177.3535
373	0.172393	-45.4108	0	0.162946	0.182268	174644.5	0	0.65622	99.43556	177.1665
374	0.172466	-45.4217	0	0.163021	0.182339	174640.6	0	0.655478	99.43515	177.0365
375	0.172337	-45.4105	0	0.16289	0.182213	174640	0	0.656563	99.43563	177.1871
376	0.172313	-45.4139	0	0.162866	0.182188	174640.8	0	0.656632	99.43605	177.3213
377	0.172426	-45.4183	0	0.162981	0.1823	174643.8	0	0.655864	99.43553	177.1569
378	0.172373	-45.4146	0	0.162927	0.182248	174644.7	0	0.656293	99.43589	177.2703
379	0.17221	-45.4333	0	0.162768	0.182081	174600.2	0	0.656661	99.43591	177.2761
380	0.172069	-45.498	0	0.16264	0.181925	174609.5	0	0.655639	99.43532	177.0927
381	0.172026	-45.5214	0	0.162603	0.181878	174563.6	0	0.65516	99.43484	176.9421
382	0.172277	-45.4169	0	0.162831	0.182151	174590.5	0	0.656733	99.43514	177.0341
383	0.172446	-45.4162	0	0.163	0.182321	174639.2	0	0.655736	99.43516	177.0419
384	0.171979	-45.5516	0	0.162562	0.181824	174199.2	0	0.654508	99.43375	176.6005
385	0.17225	-45.4236	0	0.162805	0.182122	174627.1	0	0.656718	99.43608	177.3301
386	0.172315	-45.4163	0	0.162869	0.182189	174643.2	0	0.656585	99.43613	177.3444
387	0.172216	-45.4349	0	0.162775	0.182087	174634.1	0	0.656625	99.43616	177.3542
388	0.172503	-45.4265	0	0.163059	0.182375	174549.3	0	0.655058	99.43321	176.4321
389	0.172302	-45.4129	0	0.162855	0.182177	174514.1	0	0.656694	99.43134	175.8527
390	0.172379	-45.409	0	0.162931	0.182255	174641.7	0	0.656341	99.40692	168.6108
391	0.172476	-45.4211	0	0.16303	0.182349	174631.5	0	0.655403	99.43483	176.9374
392	0.172145	-45.457	0	0.162708	0.182011	174592.5	0	0.656352	99.43579	177.2384
393	0.172383	-45.4134	0	0.162936	0.182258	174644.7	0	0.656249	99.43583	177.25
394	0.172558	-45.4473	0	0.163118	0.182425	174630.1	0	0.654164	99.43404	176.6903
395	0.172199	-45.4399	0	0.162758	0.182068	174631	0	0.656578	99.43611	177.3401
396	0.17201	-45.5311	0	0.162588	0.181859	174476.6	0	0.65495	99.43452	176.8405
397	0.172038	-45.5126	0	0.162612	0.181891	174497	0	0.655331	99.43484	176.9399
398	0.171964	-45.5653	0	0.16255	0.181806	174526.2	0	0.654222	99.43403	176.6875
399	0.172421	-45.4322	0	0.162978	0.182292	174644.2	0	0.655697	99.43544	177.1292
400	0.172189	-45.4413	0	0.162749	0.182058	174618.9	0	0.656569	99.43603	177.3154
401	0.172529	-45.4357	0	0.163087	0.182399	174625.8	0	0.654657	99.43436	176.7896
402	0.172537	-45.4389	0	0.163095	0.182406	174629.2	0	0.654526	99.4343	176.7732
403	0.172164	-45.452	0	0.162726	0.182031	174624.5	0	0.656425	99.43598	177.2982
404	0.172412	-45.413	0	0.162965	0.182287	174643.9	0	0.656054	99.43556	177.1662

405	0.172047	-45.5124	0	0.162622	0.181901	174614.6	0		0.655364	99.43511	177.0263
406	0.172388	-45.4102	0	0.162941	0.182264	174644.6	0		0.65626	99.43545	177.1329
407	0.172346	-45.4169	0	0.1629	0.18222	174644.5	0		0.656418	99.43602	177.3106
408	0.172634	-45.477	0	0.1632	0.182495	174559.6	0		0.652767	99.4326	176.2433
409	0.172054	-45.503	0	0.162627	0.181909	174535.7	0		0.655526	99.43506	177.0106
410	0.172311	-45.412	0	0.162864	0.182186	174604.6	0		0.656668	99.43435	176.789
411	0.172454	-45.4174	0	0.163008	0.182328	174638	0		0.655654	99.4351	177.0219
412	0.172528	-45.4334	0	0.163085	0.182399	174474	0		0.654701	99.43251	176.2137
413	0.172418	-45.4122	0	0.162972	0.182294	174642	0		0.656015	99.43521	177.0556
414	0.172477	-45.4209	0	0.163032	0.18235	174619.8	0		0.655393	99.43449	176.8305
415	0.172271	-45.4254	0	0.162828	0.182144	174642.1	0		0.656623	99.4362	177.3664
416	0.172615	-45.4706	0	0.163179	0.182477	174618.4	0		0.653115	99.43313	176.4072
417	0.172437	-45.4157	0	0.162991	0.182312	174642	0		0.655815	99.43535	177.1014
418	0.17215	-45.4535	0	0.162712	0.182016	174428.3	0		0.656405	99.43506	177.0086
419	0.172214	-45.4351	0	0.162773	0.182085	174632.8	0		0.656626	99.43615	177.3515
420	0.172319	-45.4151	0	0.162873	0.182193	174643.1	0		0.656585	99.43611	177.3381
421	0.1721	-45.477	0	0.162667	0.181961	174472.8	0		0.656013	99.43519	177.0509
422	0.172598	-45.4725	0	0.163163	0.18246	174635.8	0		0.653307	99.43338	176.4835
423	0.171873	-45.6609	0	0.162478	0.181693	174615.8	0		0.652182	99.43241	176.1849
424	0.172351	-45.4129	0	0.162905	0.182227	174644.4	0		0.656448	99.43598	177.2988
425	0.172192	-45.4387	0	0.162752	0.182062	174590.1	0		0.656604	99.43586	177.2597
426	0.172473	-45.4301	0	0.16303	0.182344	174642.5	0		0.655293	99.43507	177.0139
427	0.172191	-45.4396	0	0.16275	0.18206	174603.2	0		0.656592	99.43594	177.2866
428	0.172465	-45.4184	0	0.163019	0.182338	174624.4	0		0.655544	99.43455	176.8514
429	0.172109	-45.4734	0	0.162675	0.181971	174565.1	0		0.65608	99.43552	177.1551
430	0.172579	-45.4624	0	0.163142	0.182443	174636.2	0		0.653689	99.4337	176.5839
431	0.172056	-45.5023	0	0.162628	0.181911	174543.1	0		0.65554	99.43509	177.0192
432	0.172063	-45.4967	0	0.162634	0.18192	174420.6	0		0.655644	99.43486	176.9481
433	0.172184	-45.4429	0	0.162744	0.182053	174616.7	0		0.656549	99.43601	177.3084
434	0.171941	-45.5849	0	0.16253	0.181778	174553.3	0		0.653798	99.43372	176.59
435	0.172198	-45.4372	0	0.162756	0.182067	174597.7	0		0.656621	99.43591	177.275
436	0.17214	-45.459	0	0.162703	0.182005	174574.2	0		0.65632	99.43569	177.2087
437	0.172161	-45.4521	0	0.162723	0.182027	174617.2	0		0.656424	99.43594	177.2871
438	0.172051	-45.504	0	0.162624	0.181906	174422.3	0		0.655502	99.43479	176.9238

439	0.171903	-45.6151	0	0.162498	0.181733	174378.7	0	0.653091	99.43293	176.3461
440	0.171918	-45.6031	0	0.162511	0.181751	174499.8	0	0.653375	99.4333	176.4606
441	0.172079	-45.4983	0	0.162651	0.181935	174628.9	0	0.655656	99.43539	177.1122
442	0.171921	-45.601	0	0.162514	0.181755	174533.7	0	0.653431	99.43339	176.4873
443	0.172544	-45.4392	0	0.163102	0.182413	174595.5	0	0.654439	99.4339	176.6484
444	0.172844	-45.6274	0	0.16344	0.182672	174559.1	0	0.647082	99.42789	174.7927
445	0.172354	-45.41	0	0.162907	0.18223	174643.1	0	0.656477	99.43564	177.1906
446	0.172477	-45.4215	0	0.163032	0.18235	174631.6	0	0.655386	99.43483	176.9371
447	0.172112	-45.4749	0	0.162679	0.181974	174617.5	0	0.656061	99.43568	177.2046
448	0.172218	-45.4317	0	0.162775	0.182089	174618.7	0	0.65667	99.43605	177.3212
449	0.172794	-45.5877	0	0.163381	0.18263	174600.4	0	0.648611	99.42927	175.2149
450	0.172264	-45.4215	0	0.16282	0.182138	174635.4	0	0.656706	99.43615	177.3529
451	0.172471	-45.4199	0	0.163025	0.182344	174629.1	0	0.655467	99.43476	176.9144
452	0.171755	-45.7691	0	0.162382	0.181552	174326.3	0	0.649424	99.42984	175.389
453	0.172397	-45.4141	0	0.16295	0.182271	174644.5	0	0.656145	99.43574	177.223
454	0.172199	-45.4386	0	0.162758	0.182068	174624.9	0	0.656598	99.43608	177.3313
455	0.172437	-45.4136	0	0.16299	0.182311	174621.4	0	0.655853	99.43347	176.5145
456	0.172051	-45.5042	0	0.162623	0.181906	174425.4	0	0.655497	99.43479	176.9252
457	0.17231	-45.412	0	0.162863	0.182185	174578.4	0	0.656672	99.43319	176.4253
458	0.172477	-45.4215	0	0.163032	0.18235	174631.6	0	0.655386	99.43483	176.9371
459	0.172139	-45.4615	0	0.162703	0.182003	174614.7	0	0.656282	99.43584	177.2535
460	0.1721	-45.477	0	0.162667	0.181961	174472.8	0	0.656013	99.43519	177.0509
461	0.172148	-45.4546	0	0.162711	0.182014	174520.4	0	0.656388	99.43548	177.1419
462	0.17242	-45.4123	0	0.162973	0.182295	174641.4	0	0.656	99.43512	177.028
463	0.172138	-45.4594	0	0.162701	0.182003	174544.1	0	0.656314	99.43557	177.1688
464	0.172373	-45.4094	0	0.162925	0.182249	174644.3	0	0.656373	99.43514	177.035
465	0.172565	-45.4477	0	0.163125	0.182432	174616.8	0	0.654079	99.43387	176.6372
466	0.172165	-45.4475	0	0.162726	0.182033	174454.8	0	0.656493	99.43513	177.0304
467	0.172538	-45.4379	0	0.163096	0.182408	174615.8	0	0.654524	99.43416	176.7282
468	0.172311	-45.4124	0	0.162865	0.182187	174630.3	0	0.65666	99.43557	177.1697
469	0.172282	-45.4162	0	0.162837	0.182157	174613.8	0	0.656723	99.43557	177.1684
470	0.172336	-45.4108	0	0.162889	0.182212	174640.8	0	0.656564	99.43574	177.2245
471	0.172455	-45.4166	0	0.163008	0.182329	174627.9	0	0.655658	99.43459	176.8621
472	0.172545	-45.4402	0	0.163104	0.182414	174612.8	0	0.654413	99.43406	176.6972

473	0.17202	-45.5241	0	0.162597	0.181871	174479.8	0	0.655097	99.43463	176.8762
474	0.17229	-45.4189	0	0.162845	0.182164	174641.4	0	0.656652	99.43617	177.3598
475	0.172169	-45.4493	0	0.16273	0.182036	174620	0	0.656464	99.43598	177.2992
476	0.172179	-45.4426	0	0.162739	0.182048	174523.8	0	0.656558	99.43546	177.1352
477	0.172415	-45.4154	0	0.162968	0.182289	174644.1	0	0.655997	99.43561	177.183
478	0.172259	-45.4214	0	0.162814	0.182132	174627.5	0	0.656723	99.43605	177.3212
479	0.172164	-45.4482	0	0.162725	0.182031	174489.3	0	0.656482	99.43531	177.0895
480	0.172022	-45.5213	0	0.162599	0.181874	173910.9	0	0.655148	99.43356	176.5417
481	0.17231	-45.4126	0	0.162864	0.182186	174632.3	0	0.656661	99.43568	177.2056
482	0.172075	-45.4901	0	0.162645	0.181933	174389.5	0	0.65577	99.43485	176.9428
483	0.172374	-45.4094	0	0.162927	0.18225	174644.4	0	0.656367	99.435	176.9901
484	0.172358	-45.4093	0	0.162911	0.182234	174641.5	0	0.656464	99.43482	176.936
485	0.172241	-45.4247	0	0.162797	0.182113	174602.5	0	0.656725	99.43581	177.2457
486	0.172309	-45.4145	0	0.162862	0.182183	174640.7	0	0.656641	99.43608	177.329
487	0.172291	-45.417	0	0.162845	0.182165	174639.1	0	0.656678	99.43613	177.3446
488	0.172233	-45.4278	0	0.16279	0.182105	174626	0	0.656696	99.4361	177.337
489	0.1725	-45.4303	0	0.163056	0.182371	174638.6	0	0.655037	99.4348	176.9285
490	0.172357	-45.4141	0	0.162911	0.182232	174644.6	0	0.656395	99.43597	177.2949
491	0.172569	-45.4567	0	0.16313	0.182434	174636.1	0	0.653899	99.43387	176.6383
492	0.172296	-45.4167	0	0.162851	0.182171	174640.5	0	0.65666	99.43614	177.3478
493	0.172257	-45.4216	0	0.162813	0.182131	174625.1	0	0.656726	99.43602	177.3121
494	0.172392	-45.4131	0	0.162945	0.182267	174644.6	0	0.656195	99.43576	177.229
495	0.172371	-45.4112	0	0.162924	0.182246	174644.6	0	0.656361	99.43582	177.2497
496	0.172328	-45.4127	0	0.162882	0.182203	174642.6	0	0.656575	99.43602	177.3117
497	0.172151	-45.4557	0	0.162714	0.182017	174611.9	0	0.656372	99.43589	177.2689
498	0.172224	-45.4295	0	0.162781	0.182096	174612.9	0	0.656691	99.43599	177.3023
499	0.172144	-45.457	0	0.162707	0.182009	174557.4	0	0.656351	99.43564	177.1906
500	0.172119	-45.4666	0	0.162684	0.181983	169675.5	0	0.656193	99.41818	171.8758
501	0.17222	-45.4313	0	0.162778	0.182091	174622	0	0.656673	99.43608	177.3294
502	0.172501	-45.4263	0	0.163057	0.182373	174599.9	0	0.655081	99.43412	176.7145
503	0.171976	-45.5546	0	0.162559	0.18182	174328.4	0	0.654444	99.43391	176.6491
504	0.171941	-45.5823	0	0.16253	0.181779	174413.1	0	0.653837	99.43357	176.5434
505	0.172125	-45.4644	0	0.16269	0.181989	174441.3	0	0.656232	99.43514	177.0359
506	0.172217	-45.435	0	0.162775	0.182087	174634.4	0	0.656623	99.43616	177.3547

507	0.172103	-45.4746	0	0.16267	0.181965	173738.3	0		0.656054	99.43284	176.3177
508	0.172373	-45.4192	0	0.162927	0.182246	174644.7	0		0.656226	99.43587	177.2639
509	0.172203	-45.4368	0	0.162762	0.182073	174622.7	0		0.656618	99.43608	177.329
510	0.172228	-45.4295	0	0.162786	0.1821	174628.3	0		0.656681	99.43613	177.3449
511	0.17216	-45.4511	0	0.162721	0.182026	174591.7	0		0.656441	99.43583	177.2513
512	0.172204	-45.4371	0	0.162763	0.182074	174626.7	0		0.656613	99.4361	177.337
513	0.172341	-45.4095	0	0.162893	0.182217	174539.1	0		0.656558	99.42192	172.9861
514	0.172042	-45.5154	0	0.162617	0.181895	174612.6	0		0.655303	99.43506	177.0092
515	0.172966	-45.7459	0	0.163585	0.182767	79344.82	0		0.642789	99.35768	155.6851
516	0.172089	-45.4846	0	0.162658	0.181949	174591.1	0		0.655882	99.43547	177.137
517	0.172322	-45.4122	0	0.162875	0.182197	174640.3	0		0.656612	99.43593	177.2828
518	0.172344	-45.4116	0	0.162897	0.18222	174643.6	0		0.656508	99.43595	177.288
519	0.172197	-45.4394	0	0.162757	0.182067	174626.7	0		0.656587	99.43609	177.3332
520	0.172478	-45.4234	0	0.163033	0.18235	174638.7	0		0.655351	99.43501	176.9957
521	0.172202	-45.4433	0	0.162762	0.18207	174638	0		0.656522	99.43612	177.3411
522	0.172218	-45.4317	0	0.162775	0.182089	174618.7	0		0.65667	99.43605	177.3212
523	0.171838	-45.6756	0	0.162446	0.181656	173976.5	0		0.651669	99.43142	175.8776
524	0.172053	-45.5025	0	0.162626	0.181909	174411.6	0		0.655531	99.43478	176.9215
525	0.172429	-45.417	0	0.162983	0.182303	174643.5	0		0.655863	99.4355	177.1479
526	0.172054	-45.5043	0	0.162627	0.181909	174580.1	0		0.655506	99.43515	177.0381
527	0.172014	-45.5281	0	0.162592	0.181864	174489	0		0.655014	99.43459	176.8624
528	0.171937	-45.613	0	0.162532	0.181768	174628.1	0		0.653348	99.43343	176.5003
529	0.172139	-45.4597	0	0.162703	0.182004	174590.7	0		0.65631	99.43576	177.2285
530	0.171686	-45.8562	0	0.16233	0.181463	174140.1	0		0.647269	99.42789	174.7925
531	0.17241	-45.4119	0	0.162964	0.182286	174643.5	0		0.656079	99.43541	177.1205
532	0.172607	-45.4712	0	0.163172	0.182469	174631.1	0		0.65321	99.43327	176.4507
533	0.172292	-45.4204	0	0.162847	0.182165	174642.5	0		0.656623	99.43618	177.3612
534	0.172503	-45.4419	0	0.163062	0.182372	174641.7	0		0.654826	99.43469	176.893
535	0.172537	-45.4373	0	0.163095	0.182406	174614.8	0		0.654549	99.43416	176.7293
536	0.172375	-45.4104	0	0.162928	0.182251	174644.7	0		0.656344	99.4357	177.2093
537	0.172262	-45.423	0	0.162818	0.182135	174638	0		0.656689	99.43619	177.3644
538	0.172375	-45.4089	0	0.162927	0.182251	174641.5	0		0.656369	99.42653	174.3767
539	0.172451	-45.4162	0	0.163005	0.182325	174633.2	0		0.655694	99.43483	176.9373
540	0.172014	-45.5268	0	0.162591	0.181864	172559.3	0		0.655032	99.43102	175.7541

541	0.172223	-45.4326	0	0.162781	0.182094	174633.9	0	0.656646	99.43617	177.3569
542	0.173391	-46.4082	0	0.16414	0.183049	153512.9	0	0.621344	99.39866	166.2963
543	0.172338	-45.4166	0	0.162892	0.182212	174644.3	0	0.656467	99.43605	177.3217
544	0.172368	-45.4115	0	0.162921	0.182244	174644.6	0	0.65637	99.43586	177.2595
545	0.172187	-45.4406	0	0.162747	0.182056	174596.4	0	0.656581	99.4359	177.2727
546	0.172343	-45.4103	0	0.162896	0.182219	174641.4	0	0.656535	99.43564	177.1903
547	0.172416	-45.4157	0	0.16297	0.182291	174644.1	0	0.655981	99.4356	177.1796
548	0.172323	-45.4107	0	0.162876	0.182199	174080.5	0	0.656632	99.39933	166.4804
549	0.172635	-45.477	0	0.163201	0.182496	174518.4	0	0.652756	99.43243	176.1882
550	0.172101	-45.4761	0	0.162668	0.181962	174430.4	0	0.656029	99.43506	177.011
551	0.172362	-45.4099	0	0.162915	0.182238	174644	0	0.656432	99.43563	177.1872
552	0.172356	-45.4119	0	0.162909	0.182231	174644.3	0	0.656439	99.43593	177.2837
553	0.172402	-45.4123	0	0.162955	0.182277	174644.3	0	0.656136	99.43564	177.1906
554	0.172449	-45.4225	0	0.163004	0.182322	174643.1	0	0.655617	99.43533	177.0947
555	0.172258	-45.4219	0	0.162814	0.182131	174630.2	0	0.656718	99.4361	177.3348
556	0.172323	-45.4108	0	0.162876	0.182199	174600.1	0	0.656629	99.43316	176.4171
557	0.1724	-45.41	0	0.162953	0.182276	174640.6	0	0.656184	99.43188	176.0184
558	0.172545	-45.4405	0	0.163104	0.182414	174618.1	0	0.654407	99.43411	176.7122
559	0.17223	-45.4272	0	0.162786	0.182102	174578.2	0	0.656714	99.43561	177.183
560	0.171899	-45.6199	0	0.162495	0.181728	174481.3	0	0.652991	99.43296	176.3554
561	0.172155	-45.451	0	0.162717	0.182022	172973.1	0	0.656442	99.42801	174.8272
562	0.172309	-45.4123	0	0.162863	0.182185	174612.9	0	0.656671	99.43482	176.9336
563	0.172209	-45.4447	0	0.162769	0.182077	174640.3	0	0.656491	99.4361	177.3374
564	0.172252	-45.4258	0	0.162808	0.182124	174637.8	0	0.656678	99.4362	177.3668
565	0.172632	-45.4757	0	0.163198	0.182493	174531.6	0	0.652814	99.43252	176.2173
566	0.172518	-45.4313	0	0.163074	0.182389	174616.2	0	0.654843	99.43434	176.7832
567	0.171833	-45.6872	0	0.162443	0.181648	174555	0	0.651445	99.43173	175.9721
568	0.172217	-45.4397	0	0.162777	0.182087	174639.8	0	0.65655	99.43615	177.3504
569	0.17245	-45.4174	0	0.163004	0.182324	174640.3	0	0.655681	99.43522	177.0597
570	0.172473	-45.4209	0	0.163028	0.182346	174634.5	0	0.655431	99.43493	176.968
571	0.172293	-45.4247	0	0.162849	0.182166	174643.6	0	0.656552	99.43615	177.3523
572	0.172515	-45.4373	0	0.163072	0.182384	174639.5	0	0.654783	99.43462	176.8724
573	0.172433	-45.4143	0	0.162986	0.182308	174641.2	0	0.655873	99.43527	177.075
574	0.171934	-45.5943	0	0.162526	0.18177	174595.1	0	0.653616	99.43362	176.5588

575	0.17234	-45.4147	0	0.162894	0.182215	174644.2	0	0.656484	99.43605	177.319

Supplementary Table 4. Leave one out analysis by using a build-in function for including studies.

	rstudent	dffits	cook.d	cov.r	tau2.del	QE.del	hat	weight	Dfbs	inf
1	-0.0703	-0.0029	0	1.0036	0.6567	174619.3	0.0018	0.1796	-0.0029	
2	-0.3024	-0.0127	0.0002	1.0034	0.6566	174641.5	0.0018	0.1771	-0.0127	
3	-0.7912	-0.0327	0.0011	1.0024	0.6559	174643.9	0.0017	0.1709	-0.0327	
4	-1.0126	-0.0418	0.0017	1.0017	0.6555	174641.7	0.0017	0.1698	-0.0418	
5	-0.2854	-0.0119	0.0001	1.0034	0.6566	174642.1	0.0018	0.1754	-0.0119	
6	-2.199	-0.094	0.0088	0.9949	0.6508	173904.9	0.0018	0.1809	-0.094	
7	1.3717	0.0578	0.0033	1.0002	0.6544	174467.9	0.0018	0.1776	0.0578	
8	1.8915	0.0793	0.0063	0.9972	0.6524	174438.7	0.0018	0.1762	0.0793	
9	3.5456	0.1498	0.022	0.9812	0.6415	172415.5	0.0018	0.1802	0.1497	*
10	-0.9618	-0.0394	0.0016	1.0018	0.6556	174642.7	0.0017	0.1676	-0.0394	
11	0.3285	0.014	0.0002	1.0034	0.6566	174565.7	0.0018	0.1796	0.014	
12	1.9241	0.0803	0.0064	0.997	0.6523	174475.7	0.0017	0.1748	0.0803	
13	-0.4891	-0.0204	0.0004	1.0031	0.6564	174644.5	0.0018	0.1751	-0.0204	
14	0.9119	0.0385	0.0015	1.0021	0.6557	174522.7	0.0018	0.1782	0.0385	
15	-0.5135	-0.0217	0.0005	1.0031	0.6564	174644.3	0.0018	0.179	-0.0217	
16	-1.8833	-0.0748	0.0056	0.9976	0.6528	174631.8	0.0016	0.1567	-0.0748	
17	0.41	0.0173	0.0003	1.0032	0.6565	174611.1	0.0018	0.1762	0.0173	
18	-0.1869	-0.0074	0.0001	1.0032	0.6566	174643.5	0.0016	0.1609	-0.0074	
19	0.2065	0.0088	0.0001	1.0035	0.6567	174620.7	0.0018	0.1768	0.0088	
20	0.7225	0.0308	0.0009	1.0027	0.6561	174077.9	0.0018	0.181	0.0308	
21	0.3655	0.0154	0.0002	1.0033	0.6565	174619	0.0018	0.1752	0.0154	
22	0.5621	0.0235	0.0006	1.003	0.6563	174611.9	0.0017	0.1743	0.0235	
23	0.2928	0.0123	0.0002	1.0033	0.6566	174628.6	0.0017	0.173	0.0123	
24	1.0471	0.0443	0.002	1.0016	0.6554	174446.5	0.0018	0.1791	0.0443	
25	1.4542	0.0617	0.0038	0.9998	0.6541	174161.2	0.0018	0.18	0.0617	
26	1.376	0.0565	0.0032	1.0002	0.6545	174591.6	0.0017	0.1686	0.0565	
27	0.8887	0.0373	0.0014	1.0021	0.6558	174582.2	0.0018	0.1753	0.0373	
28	2.223	0.0923	0.0085	0.9949	0.6509	174474.9	0.0017	0.1732	0.0923	
29	0.0857	0.0035	0	1.0032	0.6566	174641.5	0.0016	0.1587	0.0035	

30	-1.6582	-0.0683	0.0046	0.9987	0.6535	174628.2	0.0017	0.1687	-0.0683
31	0.0091	0.0005	0	1.0035	0.6567	174634.5	0.0018	0.1753	0.0005
32	-0.7595	-0.0317	0.001	1.0025	0.656	174643.9	0.0017	0.1742	-0.0317
33	-0.174	-0.0072	0.0001	1.0035	0.6567	174637.8	0.0018	0.1771	-0.0072
34	-0.3023	-0.0127	0.0002	1.0034	0.6566	174639.1	0.0018	0.1789	-0.0127
35	-0.4044	-0.0172	0.0003	1.0033	0.6565	174631.1	0.0018	0.1811	-0.0172
36	-0.2699	-0.0114	0.0001	1.0035	0.6566	174633.4	0.0018	0.1799	-0.0114
37	-2.2272	-0.0952	0.009	0.9946	0.6506	173928.7	0.0018	0.1809	-0.0952
38	-1.8637	-0.0795	0.0063	0.9973	0.6524	174389.2	0.0018	0.1804	-0.0794
39	0.4287	0.0182	0.0003	1.0033	0.6565	174584.1	0.0018	0.1785	0.0182
40	0.3684	0.0156	0.0002	1.0033	0.6566	174608.5	0.0018	0.177	0.0156
41	-0.3046	-0.0123	0.0002	1.0032	0.6565	174644	0.0017	0.1653	-0.0123
42	0.302	0.0129	0.0002	1.0034	0.6566	174576	0.0018	0.1794	0.0129
43	-1.0673	-0.0447	0.002	1.0015	0.6553	174638.4	0.0017	0.1748	-0.0447
44	0.9452	0.0397	0.0016	1.002	0.6556	174571.8	0.0018	0.1757	0.0397
45	-0.443	-0.0186	0.0003	1.0032	0.6565	174643.8	0.0018	0.1774	-0.0186
46	-0.473	-0.0199	0.0004	1.0032	0.6564	174644.1	0.0018	0.1776	-0.0199
47	0.0338	0.0015	0	1.0036	0.6567	174593.2	0.0018	0.1802	0.0015
48	-1.1678	-0.0497	0.0025	1.0011	0.655	174552.3	0.0018	0.1809	-0.0497
49	1.2477	0.0513	0.0026	1.0008	0.6549	174597.9	0.0017	0.1687	0.0513
50	-0.0481	-0.0019	0	1.0035	0.6567	174637.6	0.0017	0.1743	-0.0019
51	-0.503	-0.0209	0.0004	1.003	0.6564	174644.6	0.0017	0.1727	-0.0209
52	1.1648	0.0492	0.0024	1.0012	0.6551	174477.1	0.0018	0.1782	0.0492
53	-0.0737	-0.003	0	1.0035	0.6567	174639.3	0.0017	0.173	-0.003
54	0.9039	0.0382	0.0015	1.0021	0.6557	174536.1	0.0018	0.1778	0.0382
55	0.6203	0.025	0.0006	1.0026	0.6562	174631.9	0.0016	0.1619	0.025
56	-0.1664	-0.0069	0	1.0035	0.6567	174636.4	0.0018	0.1777	-0.0069
57	1.7965	0.0739	0.0054	0.9979	0.6529	174559.5	0.0017	0.1696	0.0739
58	0.8041	0.0331	0.0011	1.0023	0.6559	174617.3	0.0017	0.169	0.0331
59	-1.2576	-0.0522	0.0027	1.0007	0.6548	174636.3	0.0017	0.1715	-0.0522
60	-0.0234	-0.0009	0	1.0034	0.6567	174639.4	0.0017	0.1711	-0.0009
61	0.188	0.0081	0.0001	1.0036	0.6567	174452.1	0.0018	0.181	0.0081
62	-0.0149	-0.0005	0	1.0035	0.6567	174634	0.0018	0.1761	-0.0005
63	0.7567	0.0317	0.001	1.0025	0.656	174597.3	0.0017	0.1747	0.0317

64	-0.1812	-0.0075	0.0001	1.0035	0.6567	174638.6	0.0018	0.1767	-0.0075
65	-0.3431	-0.0145	0.0002	1.0034	0.6566	174639.8	0.0018	0.1793	-0.0145
66	0.173	0.0074	0.0001	1.0035	0.6567	174608.6	0.0018	0.1786	0.0074
67	2.8003	0.1068	0.0113	0.9915	0.6487	174587	0.0015	0.1469	0.1069
68	0.1384	0.0056	0	1.0032	0.6566	174640.4	0.0016	0.1613	0.0056
69	-0.4097	-0.0174	0.0003	1.0033	0.6565	174635.1	0.0018	0.1809	-0.0174
70	2.2421	0.0947	0.0089	0.9946	0.6506	174035.6	0.0018	0.1792	0.0947
71	-0.5522	-0.0234	0.0005	1.0031	0.6564	174644.6	0.0018	0.1796	-0.0234
72	-0.8583	-0.0361	0.0013	1.0022	0.6558	174642	0.0018	0.1766	-0.0361
73	1.1018	0.0466	0.0022	1.0014	0.6552	174471.1	0.0018	0.1786	0.0466
74	1.5949	0.0669	0.0045	0.999	0.6536	174487.5	0.0018	0.1761	0.0669
75	0.1212	0.0051	0	1.0034	0.6567	174635.9	0.0017	0.1715	0.0051
76	-0.5893	-0.0251	0.0006	1.003	0.6563	174644.5	0.0018	0.1815	-0.0251
77	-1.6918	-0.07	0.0049	0.9985	0.6533	174624.9	0.0017	0.1702	-0.07
78	1.9089	0.0805	0.0065	0.9971	0.6523	174267.4	0.0018	0.1786	0.0805
79	-0.7231	-0.0306	0.0009	1.0026	0.6561	174643.5	0.0018	0.1789	-0.0306
80	1.1109	0.0468	0.0022	1.0014	0.6552	174527.6	0.0018	0.1771	0.0468
81	-0.2134	-0.0089	0.0001	1.0035	0.6567	174640.4	0.0018	0.1758	-0.0089
82	0.3812	0.0163	0.0003	1.0034	0.6566	174511.5	0.0018	0.1803	0.0163
83	1.0345	0.0436	0.0019	1.0017	0.6554	174528.6	0.0018	0.1774	0.0436
84	0.3715	0.0157	0.0002	1.0033	0.6566	174602.5	0.0018	0.1776	0.0157
85	0.3995	0.0163	0.0003	1.003	0.6565	174634.8	0.0016	0.1645	0.0163
86	1.3957	0.0589	0.0035	1.0001	0.6543	174409.8	0.0018	0.1785	0.0589
87	0.2112	0.0088	0.0001	1.0034	0.6566	174633.4	0.0017	0.1716	0.0088
88	-0.9804	-0.0407	0.0017	1.0018	0.6555	174641.7	0.0017	0.1717	-0.0407
89	0.0632	0.0028	0	1.0036	0.6567	174597.9	0.0018	0.1799	0.0028
90	1.7667	0.0732	0.0053	0.9981	0.653	174537.3	0.0017	0.1723	0.0732
91	0.4884	0.0207	0.0004	1.0032	0.6564	174561.7	0.0018	0.179	0.0207
92	1.5542	0.0656	0.0043	0.9993	0.6538	174386.7	0.0018	0.1783	0.0656
93	0.7844	0.0334	0.0011	1.0025	0.656	174365.2	0.0018	0.1803	0.0334
94	-0.1118	-0.0047	0	1.0036	0.6567	174566.5	0.0018	0.181	-0.0047
95	1.398	0.0581	0.0034	1.0001	0.6544	174562.4	0.0017	0.1729	0.0581
96	1.1168	0.0475	0.0023	1.0014	0.6552	174114.2	0.0018	0.1805	0.0475
97	0.5462	0.0232	0.0005	1.0031	0.6564	174561.3	0.0018	0.1787	0.0232

98	0.6829	0.0277	0.0008	1.0025	0.6561	174629.1	0.0016	0.1635	0.0277
99	-0.0696	-0.0029	0	1.0036	0.6567	174611.2	0.0018	0.1801	-0.0029
100	-0.5246	-0.0215	0.0005	1.0029	0.6563	174644.7	0.0017	0.1693	-0.0215
101	-0.24	-0.0101	0.0001	1.0035	0.6567	174635.9	0.0018	0.179	-0.0101
102	-1.2373	-0.0507	0.0026	1.0008	0.6549	174639.1	0.0017	0.1671	-0.0507
103	-0.0402	-0.0016	0	1.0036	0.6567	174630.1	0.0018	0.1778	-0.0016
104	1.1804	0.0492	0.0024	1.0011	0.655	174572.4	0.0017	0.1738	0.0492
105	-0.1097	-0.0045	0	1.0035	0.6567	174637.3	0.0018	0.176	-0.0045
106	0.2757	0.0117	0.0001	1.0034	0.6566	174613.9	0.0018	0.1772	0.0117
107	-0.3023	-0.0127	0.0002	1.0034	0.6566	174639.1	0.0018	0.1789	-0.0127
108	0.06	0.0024	0	1.003	0.6565	174642.6	0.0015	0.1504	0.0024
109	0.1266	0.0052	0	1.0033	0.6566	174639.4	0.0017	0.1652	0.0052
110	0.6846	0.0282	0.0008	1.0026	0.6561	174622.8	0.0017	0.1685	0.0282
111	-1.6582	-0.0683	0.0046	0.9987	0.6535	174628.2	0.0017	0.1687	-0.0683
112	-0.6514	-0.0271	0.0007	1.0027	0.6562	174644.6	0.0017	0.1737	-0.0271
113	-0.8733	-0.0366	0.0013	1.0022	0.6558	174642	0.0018	0.176	-0.0366
114	-1.2851	-0.0525	0.0028	1.0006	0.6548	174638.6	0.0017	0.1664	-0.0525
115	1.5497	0.0632	0.004	0.9993	0.6539	174590.6	0.0017	0.1666	0.0632
116	0.6193	0.0263	0.0007	1.0029	0.6563	174519.3	0.0018	0.1794	0.0263
117	0.2165	0.0089	0.0001	1.0033	0.6566	174637.7	0.0017	0.1657	0.0089
118	0.5909	0.0243	0.0006	1.0028	0.6563	174625.6	0.0017	0.1688	0.0243
119	0.0301	0.0013	0	1.0033	0.6566	174640.7	0.0017	0.1654	0.0013
120	-0.004	-0.0001	0	1.0034	0.6567	174639.5	0.0017	0.1703	-0.0001
121	-1.343	-0.055	0.003	1.0003	0.6546	174637.2	0.0017	0.1672	-0.055
122	1.536	0.0638	0.0041	0.9994	0.6539	174553.1	0.0017	0.1727	0.0638
123	0.1832	0.0077	0.0001	1.0034	0.6567	174632.2	0.0017	0.1731	0.0077
124	-0.8216	-0.0334	0.0011	1.0022	0.6559	174644	0.0017	0.1652	-0.0334
125	1.0843	0.0448	0.002	1.0014	0.6553	174600.4	0.0017	0.1703	0.0448
126	0.407	0.0173	0.0003	1.0033	0.6565	174587.4	0.0018	0.1784	0.0173
127	0.3246	0.0135	0.0002	1.0033	0.6566	174630.1	0.0017	0.1715	0.0135
128	-1.1677	-0.0498	0.0025	1.0011	0.655	174458.4	0.0018	0.1812	-0.0498
129	-0.4228	-0.0175	0.0003	1.0031	0.6565	174644.3	0.0017	0.1715	-0.0175
130	-0.1827	-0.0077	0.0001	1.0035	0.6567	174629.4	0.0018	0.1796	-0.0077
131	-0.8785	-0.0368	0.0014	1.0021	0.6558	174642.3	0.0018	0.175	-0.0368

132	-1.0869	-0.044	0.0019	1.0013	0.6553	174642	0.0016	0.1635	-0.044
133	0.4738	0.0201	0.0004	1.0032	0.6565	174568.9	0.0018	0.1788	0.0201
134	0.6438	0.0264	0.0007	1.0027	0.6562	174626.2	0.0017	0.1671	0.0264
135	-0.3863	-0.016	0.0003	1.0032	0.6565	174643.8	0.0017	0.1737	-0.016
136	-0.7646	-0.0324	0.0011	1.0026	0.656	174640.7	0.0018	0.1802	-0.0324
137	-0.365	-0.015	0.0002	1.0032	0.6565	174644	0.0017	0.1704	-0.015
138	0.9369	0.0364	0.0013	1.0017	0.6556	174631.9	0.0015	0.1507	0.0364
139	0.4407	0.0184	0.0003	1.0031	0.6565	174623.3	0.0017	0.1728	0.0184
140	-0.0642	-0.0027	0	1.0036	0.6567	174573.4	0.0018	0.1808	-0.0027
141	0.5307	0.0225	0.0005	1.0031	0.6564	174583.4	0.0018	0.1779	0.0225
142	-0.3007	-0.0126	0.0002	1.0034	0.6566	174641.6	0.0018	0.1769	-0.0126
143	-1.2287	-0.0508	0.0026	1.0008	0.6549	174637.8	0.0017	0.1704	-0.0508
144	-1.0743	-0.0457	0.0021	1.0015	0.6553	174588.9	0.0018	0.1808	-0.0457
145	-0.7041	-0.0283	0.0008	1.0024	0.6561	174644.5	0.0016	0.1619	-0.0283
146	0.3778	0.016	0.0003	1.0033	0.6565	174606.8	0.0018	0.1771	0.016
147	-0.1285	-0.0054	0	1.0036	0.6567	174629.6	0.0018	0.179	-0.0054
148	-0.7296	-0.0309	0.001	1.0026	0.6561	174642.8	0.0018	0.1797	-0.0309
149	-1.7644	-0.0609	0.0037	0.9987	0.6538	174639.9	0.0012	0.1189	-0.061
150	-0.3845	-0.0161	0.0003	1.0033	0.6565	174643.5	0.0018	0.1758	-0.0161
151	-0.6068	-0.0251	0.0006	1.0028	0.6562	174644.7	0.0017	0.1714	-0.0251
152	1.1188	0.0462	0.0021	1.0013	0.6552	174597.7	0.0017	0.1705	0.0462
153	-0.0069	-0.0002	0	1.0036	0.6567	174438.5	0.0018	0.1812	-0.0002
154	-1.4245	-0.0604	0.0036	0.9999	0.6542	174600.3	0.0018	0.1787	-0.0604
155	0.5462	0.0219	0.0005	1.0027	0.6563	174634.5	0.0016	0.1599	0.0219
156	0.7941	0.0338	0.0011	1.0025	0.656	174387.2	0.0018	0.1802	0.0338
157	-0.381	-0.016	0.0003	1.0033	0.6565	174642.8	0.0018	0.1775	-0.016
158	0.5586	0.0236	0.0006	1.003	0.6563	174582.6	0.0018	0.1777	0.0236
159	-0.1892	-0.0079	0.0001	1.0035	0.6567	174640.1	0.0018	0.1755	-0.0079
160	-1.0543	-0.0447	0.002	1.0016	0.6554	174623.6	0.0018	0.1797	-0.0447
161	0.6732	0.0283	0.0008	1.0027	0.6562	174595.3	0.0018	0.1758	0.0283
162	-1.3124	-0.055	0.003	1.0005	0.6546	174630.2	0.0017	0.1749	-0.055
163	0.0827	0.0035	0	1.0035	0.6567	174635.6	0.0017	0.1728	0.0035
164	-0.4315	-0.0179	0.0003	1.0032	0.6565	174644.2	0.0017	0.1736	-0.0179
165	0.8429	0.035	0.0012	1.0022	0.6558	174606.4	0.0017	0.172	0.035

166	-0.7308	-0.031	0.001	1.0026	0.6561	174641.9	0.0018	0.1803	-0.031
167	2.5569	0.1031	0.0105	0.9926	0.6493	174545.2	0.0016	0.1639	0.1032
168	-0.8381	-0.0331	0.0011	1.002	0.6558	174644.1	0.0016	0.1555	-0.0331
169	-1.1316	-0.0474	0.0022	1.0013	0.6552	174636.2	0.0018	0.1751	-0.0474
170	0.9604	0.0408	0.0017	1.0019	0.6556	174405.2	0.0018	0.1797	0.0408
171	-0.307	-0.013	0.0002	1.0035	0.6566	174580.1	0.0018	0.1813	-0.013
172	-1.6726	-0.0711	0.005	0.9985	0.6533	174534.1	0.0018	0.1796	-0.0711
173	-1.2747	-0.0534	0.0028	1.0006	0.6547	174632	0.0017	0.1747	-0.0534
174	0.0338	0.0015	0	1.0036	0.6567	174593.2	0.0018	0.1802	0.0015
175	-1.0142	-0.0348	0.0012	1.0011	0.6555	174643.9	0.0012	0.1174	-0.0348
176	-0.4016	-0.0168	0.0003	1.0033	0.6565	174643.6	0.0018	0.1761	-0.0168
177	-0.3029	-0.0127	0.0002	1.0034	0.6566	174640.9	0.0018	0.1777	-0.0127
178	0.1454	0.0062	0	1.0035	0.6567	174628.1	0.0018	0.1757	0.0062
179	-0.1086	-0.0045	0	1.0035	0.6567	174637.7	0.0018	0.1757	-0.0045
180	-1.4901	-0.0603	0.0036	0.9996	0.6541	174636.4	0.0016	0.1628	-0.0603
181	-0.1206	-0.0049	0	1.0034	0.6567	174640.8	0.0017	0.1718	-0.0049
182	1.0681	0.0451	0.002	1.0015	0.6553	174501.9	0.0018	0.1781	0.0451
183	-0.059	-0.0024	0	1.0035	0.6567	174637.9	0.0017	0.1743	-0.0024
184	0.947	0.0401	0.0016	1.002	0.6556	174455.8	0.0018	0.1793	0.0401
185	0.1751	0.0075	0.0001	1.0036	0.6567	174518.8	0.0018	0.1807	0.0075
186	0.5073	0.0212	0.0004	1.003	0.6564	174620.7	0.0017	0.1727	0.0212
187	-0.4178	-0.017	0.0003	1.003	0.6564	174644.5	0.0017	0.1662	-0.017
188	0.3242	0.0138	0.0002	1.0034	0.6566	174585.9	0.0018	0.179	0.0138
189	-0.9852	-0.0416	0.0017	1.0018	0.6555	174636.7	0.0018	0.178	-0.0416
190	0.0795	0.0035	0	1.0036	0.6567	174544.1	0.0018	0.1807	0.0035
191	1.5368	0.0641	0.0041	0.9994	0.6539	174530.7	0.0017	0.1744	0.0641
192	-0.2223	-0.0091	0.0001	1.0034	0.6566	174642.3	0.0017	0.1719	-0.0091
193	0.7802	0.0322	0.001	1.0024	0.656	174617.2	0.0017	0.1695	0.0322
194	0.1437	0.0062	0	1.0036	0.6567	174540.2	0.0018	0.1806	0.0062
195	0.3036	0.0121	0.0001	1.003	0.6565	174639.1	0.0016	0.158	0.0121
196	0.5462	0.0219	0.0005	1.0027	0.6563	174634.5	0.0016	0.1599	0.0219
197	-1.1447	-0.0484	0.0023	1.0012	0.6551	174628.4	0.0018	0.1781	-0.0484
198	1.5748	0.0661	0.0044	0.9992	0.6537	174473.5	0.0018	0.1766	0.0661
199	-0.9082	-0.0311	0.001	1.0014	0.6556	174644.2	0.0012	0.1171	-0.0311

200	0.7286	0.03	0.0009	1.0025	0.656	174620.6	0.0017	0.1688	0.03	
201	-4.0444	-0.1738	0.0293	0.9745	0.6369	169633.1	0.0018	0.1811	-0.1737	*
202	1.1779	0.0498	0.0025	1.0011	0.655	174443.2	0.0018	0.1787	0.0498	
203	0.0989	0.0043	0	1.0036	0.6567	174147.2	0.0018	0.1814	0.0043	
204	-0.3081	-0.0129	0.0002	1.0034	0.6566	174641.6	0.0018	0.1771	-0.0129	
205	0.2904	0.0108	0.0001	1.0026	0.6564	174642.1	0.0014	0.1372	0.0108	
206	-1.0987	-0.0427	0.0018	1.0012	0.6553	174642.9	0.0015	0.1506	-0.0427	
207	-0.7718	-0.0319	0.001	1.0024	0.656	174644.1	0.0017	0.1708	-0.0319	
208	0.3363	0.0143	0.0002	1.0034	0.6566	174598.7	0.0018	0.1782	0.0143	
209	-0.3276	-0.0133	0.0002	1.0032	0.6565	174644	0.0017	0.1675	-0.0133	
210	-0.6785	-0.0286	0.0008	1.0027	0.6562	174644.3	0.0018	0.1773	-0.0286	
211	-0.4178	-0.017	0.0003	1.003	0.6564	174644.5	0.0017	0.1662	-0.017	
212	0.3624	0.0154	0.0002	1.0034	0.6566	174588.3	0.0018	0.1786	0.0154	
213	0.8552	0.0364	0.0013	1.0023	0.6558	174189	0.0018	0.1807	0.0364	
214	1.5523	0.0652	0.0042	0.9993	0.6538	174477.4	0.0018	0.1766	0.0652	
215	1.7783	0.0743	0.0055	0.998	0.6529	174487.8	0.0018	0.1751	0.0743	
216	-0.0907	-0.0032	0	1.0025	0.6564	174644.2	0.0012	0.1249	-0.0032	
217	0.4641	0.0197	0.0004	1.0032	0.6565	174563.3	0.0018	0.1791	0.0197	
218	-1.2891	-0.0523	0.0027	1.0005	0.6548	174639.4	0.0016	0.1637	-0.0523	
219	-0.7267	-0.0307	0.0009	1.0026	0.6561	174643.5	0.0018	0.1788	-0.0307	
220	-0.1447	-0.0058	0	1.0032	0.6566	174643	0.0016	0.1631	-0.0058	
221	-0.3267	-0.0137	0.0002	1.0034	0.6566	174640.6	0.0018	0.1786	-0.0137	
222	-0.1416	-0.0059	0	1.0035	0.6567	174637.1	0.0018	0.1768	-0.0059	
223	0.0154	0.0007	0	1.0036	0.6567	174495.5	0.0018	0.1811	0.0007	
224	0.3347	0.0143	0.0002	1.0034	0.6566	174559.8	0.0018	0.1797	0.0143	
225	2.3725	0.099	0.0097	0.9937	0.65	174400.4	0.0018	0.1751	0.099	
226	-0.0196	-0.0007	0	1.0035	0.6567	174634	0.0018	0.1762	-0.0007	
227	-0.5772	-0.0245	0.0006	1.003	0.6563	174644.6	0.0018	0.1811	-0.0245	
228	0.5764	0.0238	0.0006	1.0028	0.6563	174624.6	0.0017	0.1696	0.0238	
229	-1.3327	-0.0564	0.0032	1.0004	0.6545	174615	0.0018	0.1782	-0.0564	
230	0.433	0.0179	0.0003	1.0031	0.6565	174628.9	0.0017	0.1699	0.0179	
231	1.2065	0.0504	0.0025	1.001	0.655	174564	0.0017	0.1744	0.0504	
232	0.4836	0.0196	0.0004	1.0029	0.6564	174634.4	0.0016	0.1624	0.0196	
233	-2.2074	-0.0935	0.0087	0.9949	0.6508	174513.8	0.0018	0.1779	-0.0935	

234	-0.0738	-0.003	0	1.0034	0.6567	174640.8	0.0017	0.1701	-0.003
235	-0.6079	-0.0258	0.0007	1.003	0.6563	174644.7	0.0018	0.1805	-0.0258
236	-0.0149	-0.0005	0	1.0035	0.6567	174634	0.0018	0.1761	-0.0005
237	-0.2272	-0.0092	0.0001	1.0033	0.6566	174643.2	0.0017	0.1679	-0.0092
238	0.735	0.0313	0.001	1.0026	0.6561	174342.4	0.0018	0.1805	0.0313
239	0.3804	0.0162	0.0003	1.0033	0.6566	174589.7	0.0018	0.1785	0.0162
240	-0.5703	-0.0243	0.0006	1.003	0.6563	174641.7	0.0018	0.1815	-0.0243
241	0.2302	0.0098	0.0001	1.0035	0.6567	174575	0.0018	0.1798	0.0098
242	0.5703	0.0242	0.0006	1.003	0.6563	174564.8	0.0018	0.1785	0.0242
243	0.5233	0.022	0.0005	1.003	0.6564	174608.9	0.0018	0.1754	0.022
244	-1.0032	-0.0424	0.0018	1.0018	0.6555	174635.8	0.0018	0.178	-0.0424
245	-1.1315	-0.0444	0.002	1.0011	0.6552	174642.5	0.0015	0.1535	-0.0444
246	0.294	0.0123	0.0002	1.0033	0.6566	174626.9	0.0017	0.1737	0.0123
247	-0.1452	-0.006	0	1.0035	0.6567	174639.5	0.0017	0.175	-0.006
248	1.6259	0.0683	0.0047	0.9989	0.6535	174448.5	0.0018	0.177	0.0683
249	0.7247	0.0302	0.0009	1.0026	0.6561	174607.5	0.0017	0.1733	0.0302
250	-0.2334	-0.0097	0.0001	1.0034	0.6566	174641.1	0.0018	0.1754	-0.0097
251	0.4676	0.0199	0.0004	1.0032	0.6565	174558.3	0.0018	0.1792	0.0199
252	-0.9716	-0.0414	0.0017	1.0019	0.6556	174598.3	0.0018	0.181	-0.0414
253	1.1659	0.0493	0.0024	1.0012	0.6551	174458.4	0.0018	0.1786	0.0493
254	-0.4063	-0.0172	0.0003	1.0033	0.6565	174640.6	0.0018	0.18	-0.0172
255	0.5602	0.0237	0.0006	1.003	0.6563	174577.9	0.0018	0.178	0.0237
256	1.5367	0.0652	0.0042	0.9993	0.6538	173989	0.0018	0.1803	0.0652
257	1.3552	0.0569	0.0032	1.0003	0.6545	174519	0.0018	0.1761	0.0569
258	0.1809	0.0074	0.0001	1.0032	0.6566	174639.2	0.0016	0.1633	0.0074
259	1.9554	0.0815	0.0066	0.9968	0.6521	174480	0.0017	0.1745	0.0815
260	0.9518	0.0405	0.0016	1.002	0.6556	174188	0.0018	0.1806	0.0405
261	1.3556	0.0576	0.0033	1.0003	0.6545	173790.6	0.0018	0.1807	0.0576
262	0.6504	0.027	0.0007	1.0027	0.6562	174618.6	0.0017	0.1711	0.027
263	2.4867	0.1024	0.0104	0.9929	0.6495	174487.8	0.0017	0.1708	0.1024
264	0.4805	0.02	0.0004	1.0031	0.6564	174623.5	0.0017	0.172	0.02
265	0.0689	0.003	0	1.0035	0.6567	174632.9	0.0018	0.175	0.003
266	0.4688	0.0196	0.0004	1.0031	0.6564	174622.2	0.0017	0.1727	0.0196
267	0.2216	0.0093	0.0001	1.0034	0.6566	174629.9	0.0017	0.1737	0.0093

268	0.1999	0.0085	0.0001	1.0035	0.6567	174627	0.0018	0.1753	0.0085
269	1.6379	0.0679	0.0046	0.9988	0.6535	174549.5	0.0017	0.1722	0.0679
270	-0.9276	-0.0375	0.0014	1.0019	0.6557	174643.4	0.0016	0.1634	-0.0375
271	0.0732	0.0032	0	1.0036	0.6567	174626.1	0.0018	0.1773	0.0032
272	-0.1435	-0.006	0	1.0036	0.6567	174631.2	0.0018	0.1789	-0.006
273	0.7024	0.0297	0.0009	1.0027	0.6561	174561.9	0.0018	0.1779	0.0297
274	-1.2099	-0.0508	0.0026	1.0009	0.6549	174633.1	0.0018	0.1754	-0.0508
275	0.3675	0.0154	0.0002	1.0033	0.6565	174623.6	0.0017	0.1738	0.0154
276	-0.7071	-0.0297	0.0009	1.0026	0.6561	174644.2	0.0018	0.176	-0.0297
277	2.2876	0.0965	0.0092	0.9942	0.6504	174101.3	0.0018	0.1788	0.0965
278	-0.286	-0.012	0.0001	1.0034	0.6566	174640.4	0.0018	0.1777	-0.012
279	-0.0242	-0.0009	0	1.0035	0.6567	174637.5	0.0017	0.1738	-0.0009
280	0.2129	0.009	0.0001	1.0035	0.6567	174624.1	0.0018	0.176	0.009
281	2.4973	0.1019	0.0103	0.9929	0.6495	174524	0.0017	0.1675	0.1019
282	-0.2844	-0.0119	0.0001	1.0034	0.6566	174641.8	0.0018	0.176	-0.0119
283	-1.0561	-0.0441	0.0019	1.0015	0.6554	174639.3	0.0017	0.174	-0.0441
284	-1.1773	-0.0502	0.0025	1.0011	0.655	174547.1	0.0018	0.1809	-0.0502
285	-1.079	-0.046	0.0021	1.0015	0.6553	174571.1	0.0018	0.181	-0.046
286	1.7194	0.0718	0.0051	0.9983	0.6532	174505.4	0.0017	0.1747	0.0718
287	-0.3009	-0.0126	0.0002	1.0034	0.6566	174641.3	0.0018	0.1773	-0.0126
288	-1.1401	-0.0484	0.0023	1.0013	0.6551	174608.8	0.0018	0.18	-0.0484
289	-0.2846	-0.0119	0.0001	1.0034	0.6566	174639.7	0.0018	0.1782	-0.0119
290	1.2728	0.0535	0.0029	1.0007	0.6548	174516.4	0.0018	0.1767	0.0535
291	-0.9038	-0.0385	0.0015	1.0021	0.6557	174509.4	0.0018	0.1814	-0.0385
292	-1.1646	-0.0497	0.0025	1.0012	0.6551	174461.4	0.0018	0.1812	-0.0497
293	0.3675	0.0154	0.0002	1.0033	0.6565	174623.6	0.0017	0.1738	0.0154
294	-0.6585	-0.0276	0.0008	1.0028	0.6562	174644.5	0.0018	0.1765	-0.0276
295	0.1172	0.0051	0	1.0036	0.6567	174561.5	0.0018	0.1805	0.0051
296	0.0467	0.002	0	1.0036	0.6567	174627.1	0.0018	0.1774	0.002
297	0.1422	0.006	0	1.0034	0.6567	174634	0.0017	0.1728	0.006
298	0.1207	0.0052	0	1.0035	0.6567	174625.4	0.0018	0.1769	0.0052
299	-1.1453	-0.0486	0.0024	1.0012	0.6551	174616.6	0.0018	0.1796	-0.0486
300	-0.8744	-0.0365	0.0013	1.0022	0.6558	174642.6	0.0017	0.1741	-0.0365
301	-0.4247	-0.018	0.0003	1.0033	0.6565	174641.7	0.0018	0.1799	-0.018

302	-0.0648	-0.0027	0	1.0036	0.6567	174619.1	0.0018	0.1796	-0.0027
303	-0.392	-0.0166	0.0003	1.0033	0.6565	174640.7	0.0018	0.1798	-0.0166
304	-1.4585	-0.0621	0.0038	0.9998	0.6541	174537.3	0.0018	0.1803	-0.0621
305	-0.5935	-0.0241	0.0006	1.0027	0.6562	174644.7	0.0017	0.1657	-0.0241
306	-0.4785	-0.0196	0.0004	1.003	0.6564	174644.6	0.0017	0.1677	-0.0196
307	0.634	0.0271	0.0007	1.0029	0.6562	172198.4	0.0018	0.1814	0.0271
308	-0.2862	-0.0121	0.0001	1.0035	0.6566	174614	0.0018	0.181	-0.0121
309	0.1809	0.0074	0.0001	1.0032	0.6566	174639.2	0.0016	0.1633	0.0074
310	0.2433	0.0102	0.0001	1.0034	0.6566	174629.2	0.0017	0.1736	0.0102
311	0.2791	0.0119	0.0001	1.0034	0.6566	174603.8	0.0018	0.1782	0.0119
312	-1.2162	-0.0501	0.0025	1.0009	0.6549	174638.8	0.0017	0.169	-0.0501
313	1.4492	0.0616	0.0038	0.9998	0.6541	173801.4	0.0018	0.1807	0.0616
314	-0.2107	-0.0088	0.0001	1.0035	0.6567	174640.2	0.0018	0.1759	-0.0088
315	-0.2621	-0.0111	0.0001	1.0035	0.6567	174599.7	0.0018	0.1811	-0.0111
316	-1.5517	-0.0653	0.0043	0.9993	0.6538	174612.7	0.0018	0.1763	-0.0653
317	-0.2617	-0.0106	0.0001	1.0032	0.6566	174643.7	0.0017	0.1652	-0.0106
318	-1.1998	-0.0504	0.0025	1.001	0.655	174632.8	0.0018	0.1758	-0.0504
319	0.1784	0.0075	0.0001	1.0034	0.6566	174633.6	0.0017	0.1722	0.0075
320	-0.6525	-0.0264	0.0007	1.0026	0.6561	174644.6	0.0016	0.164	-0.0264
321	1.121	0.0472	0.0022	1.0013	0.6552	174515.1	0.0018	0.1775	0.0472
322	-0.6033	-0.025	0.0006	1.0028	0.6562	174644.7	0.0017	0.172	-0.025
323	0.3007	0.0127	0.0002	1.0034	0.6566	174623	0.0018	0.175	0.0127
324	-1.4318	-0.0604	0.0036	0.9999	0.6542	174616.8	0.0018	0.1769	-0.0604
325	-1.67	-0.0666	0.0044	0.9987	0.6536	174635	0.0016	0.1585	-0.0667
326	-0.2129	-0.009	0.0001	1.0036	0.6567	174579.9	0.0018	0.1811	-0.009
327	-0.3766	-0.016	0.0003	1.0034	0.6566	174498.4	0.0018	0.1815	-0.016
328	-0.324	-0.0137	0.0002	1.0035	0.6566	174197	0.0018	0.1815	-0.0137
329	1.8994	0.0775	0.006	0.9973	0.6525	174568.1	0.0017	0.1672	0.0775
330	-0.5699	-0.0209	0.0004	1.0023	0.6561	174644.7	0.0014	0.1352	-0.0209
331	-1.3322	-0.0556	0.0031	1.0004	0.6546	174632.4	0.0017	0.1732	-0.0556
332	1.1791	0.049	0.0024	1.0011	0.655	174580.5	0.0017	0.1728	0.049
333	0.3509	0.0135	0.0002	1.0028	0.6564	174640.8	0.0015	0.1458	0.0135
334	-1.1659	-0.0486	0.0024	1.0011	0.6551	174637.3	0.0017	0.1732	-0.0486
335	-0.448	-0.0178	0.0003	1.0029	0.6564	174644.6	0.0016	0.1585	-0.0178

336	2.6313	0.1101	0.012	0.9914	0.6484	174294.7	0.0018	0.1762	0.1101
337	-0.1451	-0.0057	0	1.0031	0.6565	174643.6	0.0016	0.156	-0.0057
338	-0.3606	-0.0146	0.0002	1.0031	0.6565	174644.3	0.0016	0.1645	-0.0146
339	0.4799	0.0203	0.0004	1.0032	0.6564	174592.3	0.0018	0.1776	0.0203
340	0.1148	0.0049	0	1.0035	0.6567	174626.7	0.0018	0.1766	0.0049
341	0.0432	0.0019	0	1.0036	0.6567	174624.2	0.0018	0.178	0.0019
342	-0.1844	-0.0077	0.0001	1.0035	0.6567	174638	0.0018	0.1772	-0.0077
343	0.8619	0.0338	0.0011	1.0019	0.6558	174632	0.0015	0.1534	0.0338
344	1.1191	0.0473	0.0022	1.0013	0.6552	174463.4	0.0018	0.1786	0.0473
345	0.2305	0.0099	0.0001	1.0035	0.6567	174552.5	0.0018	0.1802	0.0099
346	2.3022	0.0978	0.0095	0.994	0.6502	165701.2	0.0018	0.1814	0.0978
347	0.2907	0.0123	0.0002	1.0034	0.6566	174620.2	0.0018	0.1759	0.0123
348	1.2277	0.0522	0.0027	1.0009	0.6549	173735.6	0.0018	0.1809	0.0522
349	0.8911	0.0379	0.0014	1.0022	0.6558	174097.2	0.0018	0.1808	0.0379
350	0.8895	0.0379	0.0014	1.0022	0.6558	174040.4	0.0018	0.1809	0.0379
351	0.3002	0.0128	0.0002	1.0035	0.6566	174485.2	0.0018	0.1806	0.0128
352	-0.494	-0.021	0.0004	1.0032	0.6564	174642.6	0.0018	0.1807	-0.021
353	1.4111	0.0599	0.0036	1	0.6543	173863.9	0.0018	0.1806	0.0599
354	0.9793	0.0412	0.0017	1.0018	0.6556	174555.9	0.0018	0.1766	0.0412
355	1.0528	0.0448	0.002	1.0016	0.6554	174126.6	0.0018	0.1806	0.0448
356	-0.7155	-0.0301	0.0009	1.0026	0.6561	174643.9	0.0018	0.1777	-0.0301
357	-0.3086	-0.013	0.0002	1.0034	0.6566	174638.2	0.0018	0.1794	-0.013
358	-0.4461	-0.0184	0.0003	1.0031	0.6564	174644.4	0.0017	0.1716	-0.0184
359	-1.0444	-0.0418	0.0017	1.0014	0.6554	174642.7	0.0016	0.1595	-0.0418
360	-1.6977	-0.0723	0.0052	0.9984	0.6532	174442.6	0.0018	0.1805	-0.0723
361	0.0176	0.0008	0	1.0033	0.6566	174641.7	0.0016	0.1616	0.0008
362	0.5073	0.0217	0.0005	1.0032	0.6564	174342.4	0.0018	0.1808	0.0217
363	-0.3348	-0.014	0.0002	1.0034	0.6566	174641.9	0.0018	0.1775	-0.014
364	0.4417	0.0187	0.0004	1.0032	0.6565	174594.6	0.0018	0.1777	0.0187
365	-0.3379	-0.013	0.0002	1.0028	0.6564	174644.5	0.0015	0.1488	-0.013
366	-0.5629	-0.0235	0.0006	1.003	0.6563	174644.7	0.0017	0.1748	-0.0235
367	0.4034	0.0161	0.0003	1.0029	0.6564	174637.8	0.0016	0.1576	0.0161
368	-0.6437	-0.026	0.0007	1.0026	0.6562	174644.6	0.0016	0.164	-0.026
369	-0.998	-0.0423	0.0018	1.0018	0.6555	174630.8	0.0018	0.1794	-0.0423

370	1.4673	0.0622	0.0039	0.9997	0.6541	174186.9	0.0018	0.1799	0.0622
371	-0.527	-0.0216	0.0005	1.0029	0.6563	174644.7	0.0017	0.1688	-0.0216
372	-0.0722	-0.0029	0	1.0035	0.6567	174637.3	0.0018	0.1752	-0.0029
373	-0.641	-0.0271	0.0007	1.0028	0.6562	174644.5	0.0018	0.1785	-0.0271
374	-1.0071	-0.042	0.0018	1.0017	0.6555	174640.6	0.0017	0.1735	-0.042
375	-0.3713	-0.0157	0.0002	1.0034	0.6566	174640	0.0018	0.1797	-0.0157
376	-0.2575	-0.0108	0.0001	1.0034	0.6566	174640.8	0.0018	0.1766	-0.0108
377	-0.8253	-0.0338	0.0011	1.0022	0.6559	174643.8	0.0017	0.1681	-0.0338
378	-0.5612	-0.023	0.0005	1.0029	0.6563	174644.7	0.0017	0.1689	-0.023
379	0.2373	0.0101	0.0001	1.0035	0.6567	174600.2	0.0018	0.1788	0.0101
380	0.9406	0.0388	0.0015	1.0019	0.6556	174609.5	0.0017	0.1698	0.0388
381	1.1346	0.0475	0.0023	1.0013	0.6552	174563.6	0.0017	0.175	0.0475
382	-0.0823	-0.0034	0	1.0036	0.6567	174590.5	0.0018	0.1807	-0.0034
383	-0.8967	-0.0379	0.0014	1.0021	0.6557	174639.2	0.0018	0.1784	-0.0379
384	1.3448	0.057	0.0032	1.0003	0.6545	174199.2	0.0018	0.18	0.057
385	0.0467	0.002	0	1.0036	0.6567	174627.1	0.0018	0.1774	0.002
386	-0.2731	-0.0112	0.0001	1.0033	0.6566	174643.2	0.0017	0.1707	-0.0112
387	0.2103	0.0088	0.0001	1.0034	0.6566	174634.1	0.0017	0.171	0.0088
388	-1.1625	-0.0495	0.0025	1.0012	0.6551	174549.3	0.0018	0.1809	-0.0495
389	-0.2032	-0.0086	0.0001	1.0036	0.6567	174514.1	0.0018	0.1813	-0.0086
390	-0.5687	-0.0242	0.0006	1.0031	0.6563	174641.7	0.0018	0.1815	-0.0242
391	-1.0366	-0.0439	0.0019	1.0016	0.6554	174631.5	0.0018	0.1788	-0.0439
392	0.5496	0.0232	0.0005	1.003	0.6564	174592.5	0.0018	0.1771	0.0232
393	-0.6033	-0.025	0.0006	1.0028	0.6562	174644.7	0.0017	0.172	-0.025
394	-1.4589	-0.0606	0.0037	0.9998	0.6542	174630.1	0.0017	0.1719	-0.0606
395	0.2953	0.0123	0.0002	1.0033	0.6566	174631	0.0017	0.1715	0.0123
396	1.2061	0.0509	0.0026	1.001	0.6549	174476.6	0.0018	0.1781	0.0509
397	1.0686	0.0451	0.002	1.0015	0.6553	174497	0.0018	0.1782	0.0451
398	1.4384	0.0602	0.0036	0.9999	0.6542	174526.2	0.0018	0.1753	0.0602
399	-0.8866	-0.0327	0.0011	1.0017	0.6557	174644.2	0.0014	0.1362	-0.0327
400	0.3402	0.0143	0.0002	1.0033	0.6566	174618.9	0.0018	0.1756	0.0143
401	-1.3004	-0.0548	0.003	1.0005	0.6547	174625.8	0.0018	0.1767	-0.0548
402	-1.3445	-0.0563	0.0032	1.0003	0.6545	174629.2	0.0017	0.1748	-0.0563
403	0.4672	0.0194	0.0004	1.0031	0.6564	174624.5	0.0017	0.1718	0.0194

404	-0.735	-0.0309	0.001	1.0026	0.6561	174643.9	0.0018	0.1765	-0.0309
405	1.06	0.0432	0.0019	1.0015	0.6554	174614.6	0.0017	0.1656	0.0432
406	-0.6175	-0.0261	0.0007	1.0029	0.6563	174644.6	0.0018	0.1794	-0.0261
407	-0.4327	-0.0175	0.0003	1.003	0.6564	174644.5	0.0016	0.1646	-0.0175
408	-1.7979	-0.0762	0.0058	0.9978	0.6528	174559.6	0.0018	0.1784	-0.0762
409	0.9911	0.0418	0.0017	1.0018	0.6555	174535.7	0.0018	0.1774	0.0418
410	-0.2456	-0.0104	0.0001	1.0035	0.6567	174604.6	0.0018	0.181	-0.0104
411	-0.9329	-0.0394	0.0016	1.002	0.6557	174638	0.0018	0.1783	-0.0394
412	-1.2799	-0.0546	0.003	1.0006	0.6547	174474	0.0018	0.181	-0.0546
413	-0.7614	-0.0323	0.001	1.0026	0.656	174642	0.0018	0.1796	-0.0323
414	-1.0406	-0.0442	0.002	1.0016	0.6554	174619.8	0.0018	0.1801	-0.0442
415	-0.0604	-0.0024	0	1.0033	0.6566	174642.1	0.0016	0.1639	-0.0024
416	-1.7366	-0.0723	0.0052	0.9982	0.6531	174618.4	0.0017	0.1724	-0.0723
417	-0.8583	-0.0361	0.0013	1.0022	0.6558	174642	0.0018	0.1766	-0.0361
418	0.5217	0.0222	0.0005	1.0031	0.6564	174428.3	0.0018	0.1805	0.0222
419	0.2204	0.0092	0.0001	1.0034	0.6566	174632.8	0.0017	0.1719	0.0092
420	-0.2908	-0.012	0.0001	1.0033	0.6566	174643.1	0.0017	0.1725	-0.012
421	0.7661	0.0325	0.0011	1.0026	0.656	174472.8	0.0018	0.1796	0.0325
422	-1.7638	-0.0688	0.0047	0.9983	0.6533	174635.8	0.0015	0.1514	-0.0688
423	2.1307	0.0788	0.0062	0.9965	0.6522	174615.8	0.0014	0.1378	0.0789
424	-0.4491	-0.0186	0.0003	1.0031	0.6564	174644.4	0.0017	0.1732	-0.0186
425	0.3209	0.0137	0.0002	1.0034	0.6566	174590.1	0.0018	0.1788	0.0137
426	-1.0895	-0.0433	0.0019	1.0013	0.6553	174642.5	0.0016	0.1579	-0.0433
427	0.3294	0.014	0.0002	1.0034	0.6566	174603.2	0.0018	0.1779	0.014
428	-0.98	-0.0416	0.0017	1.0019	0.6555	174624.4	0.0018	0.1802	-0.0416
429	0.7262	0.0307	0.0009	1.0026	0.6561	174565.1	0.0018	0.1776	0.0307
430	-1.6385	-0.065	0.0042	0.9989	0.6537	174636.2	0.0016	0.1565	-0.065
431	0.9852	0.0415	0.0017	1.0018	0.6555	174543.1	0.0018	0.1771	0.0415
432	0.9415	0.04	0.0016	1.002	0.6556	174420.6	0.0018	0.1796	0.04
433	0.3647	0.0154	0.0002	1.0033	0.6565	174616.7	0.0018	0.1757	0.0154
434	1.5644	0.0649	0.0042	0.9992	0.6538	174553.3	0.0017	0.1725	0.0649
435	0.2966	0.0126	0.0002	1.0034	0.6566	174597.7	0.0018	0.1785	0.0126
436	0.5764	0.0244	0.0006	1.003	0.6563	174574.2	0.0018	0.1781	0.0244
437	0.4791	0.0201	0.0004	1.0031	0.6564	174617.2	0.0017	0.1741	0.0201

438	1.0011	0.0424	0.0018	1.0018	0.6555	174422.3	0.0018	0.1795	0.0424
439	1.7278	0.0728	0.0053	0.9982	0.6531	174378.7	0.0018	0.1779	0.0728
440	1.6668	0.0697	0.0048	0.9986	0.6534	174499.8	0.0018	0.1752	0.0697
441	0.9268	0.0367	0.0013	1.0018	0.6557	174628.9	0.0016	0.1565	0.0367
442	1.6583	0.069	0.0047	0.9987	0.6534	174533.7	0.0017	0.1734	0.069
443	-1.3629	-0.0578	0.0033	1.0002	0.6544	174595.5	0.0018	0.1793	-0.0578
444	-2.8762	-0.1194	0.0141	0.9894	0.6471	174559.1	0.0017	0.1705	-0.1194
445	-0.4536	-0.0192	0.0004	1.0032	0.6565	174643.1	0.0018	0.1794	-0.0192
446	-1.0434	-0.0442	0.0019	1.0016	0.6554	174631.6	0.0018	0.1787	-0.0442
447	0.7237	0.0299	0.0009	1.0025	0.6561	174617.5	0.0017	0.1703	0.0299
448	0.2006	0.0085	0.0001	1.0035	0.6567	174618.7	0.0018	0.1772	0.0085
449	-2.6833	-0.1089	0.0118	0.9915	0.6486	174600.4	0.0016	0.1634	-0.109
450	-0.0251	-0.001	0	1.0035	0.6567	174635.4	0.0018	0.1755	-0.001
451	-1.0111	-0.0429	0.0018	1.0018	0.6555	174629.1	0.0018	0.1795	-0.0429
452	2.4647	0.1032	0.0105	0.9928	0.6494	174326.3	0.0018	0.1763	0.1032
453	-0.6713	-0.0278	0.0008	1.0027	0.6561	174644.5	0.0017	0.1718	-0.0278
454	0.2936	0.0123	0.0002	1.0034	0.6566	174624.9	0.0017	0.1745	0.0123
455	-0.8442	-0.0359	0.0013	1.0023	0.6559	174621.4	0.0018	0.181	-0.0359
456	1.0028	0.0425	0.0018	1.0018	0.6555	174425.4	0.0018	0.1795	0.0425
457	-0.2411	-0.0102	0.0001	1.0035	0.6567	174578.4	0.0018	0.1812	-0.0102
458	-1.0434	-0.0442	0.0019	1.0016	0.6554	174631.6	0.0018	0.1787	-0.0442
459	0.5875	0.0245	0.0006	1.0029	0.6563	174614.7	0.0017	0.1733	0.0245
460	0.7661	0.0325	0.0011	1.0026	0.656	174472.8	0.0018	0.1796	0.0325
461	0.5321	0.0226	0.0005	1.0031	0.6564	174520.4	0.0018	0.1797	0.0226
462	-0.7694	-0.0326	0.0011	1.0025	0.656	174641.4	0.0018	0.1798	-0.0326
463	0.5836	0.0248	0.0006	1.003	0.6563	174544.1	0.0018	0.1791	0.0248
464	-0.5418	-0.023	0.0005	1.0031	0.6564	174644.3	0.0018	0.1804	-0.023
465	-1.473	-0.062	0.0038	0.9997	0.6541	174616.8	0.0018	0.1764	-0.062
466	0.4487	0.0191	0.0004	1.0033	0.6565	174454.8	0.0018	0.1805	0.0191
467	-1.3395	-0.0566	0.0032	1.0004	0.6545	174615.8	0.0018	0.178	-0.0566
468	-0.2486	-0.0105	0.0001	1.0035	0.6567	174630.3	0.0018	0.1801	-0.0105
469	-0.1097	-0.0046	0	1.0036	0.6567	174613.8	0.0018	0.1802	-0.0046
470	-0.3681	-0.0155	0.0002	1.0034	0.6566	174640.8	0.0018	0.1793	-0.0155
471	-0.9321	-0.0396	0.0016	1.002	0.6557	174627.9	0.0018	0.1803	-0.0396

472	-1.373	-0.0581	0.0034	1.0002	0.6544	174612.8	0.0018	0.1781	-0.0581
473	1.155	0.0488	0.0024	1.0012	0.6551	174479.8	0.0018	0.1782	0.0488
474	-0.1515	-0.0062	0	1.0034	0.6567	174641.4	0.0017	0.1716	-0.0062
475	0.4422	0.0185	0.0003	1.0032	0.6565	174620	0.0017	0.1739	0.0185
476	0.3829	0.0163	0.0003	1.0034	0.6566	174523.8	0.0018	0.1801	0.0163
477	-0.7589	-0.0315	0.001	1.0025	0.656	174644.1	0.0017	0.1718	-0.0315
478	0.0013	0.0001	0	1.0036	0.6567	174627.5	0.0018	0.1779	0.0001
479	0.4568	0.0195	0.0004	1.0032	0.6565	174489.3	0.0018	0.1803	0.0195
480	1.1349	0.0483	0.0023	1.0013	0.6551	173910.9	0.0018	0.1808	0.0483
481	-0.2448	-0.0103	0.0001	1.0035	0.6567	174632.3	0.0018	0.1798	-0.0103
482	0.8856	0.0376	0.0014	1.0022	0.6558	174389.5	0.0018	0.18	0.0376
483	-0.5469	-0.0232	0.0005	1.0031	0.6564	174644.4	0.0018	0.1805	-0.0232
484	-0.4714	-0.02	0.0004	1.0032	0.6565	174641.5	0.0018	0.1807	-0.02
485	0.0895	0.0039	0	1.0036	0.6567	174602.5	0.0018	0.1796	0.0039
486	-0.2383	-0.0099	0.0001	1.0034	0.6566	174640.7	0.0018	0.1761	-0.0099
487	-0.1536	-0.0064	0	1.0035	0.6567	174639.1	0.0018	0.1756	-0.0064
488	0.1246	0.0053	0	1.0035	0.6567	174626	0.0018	0.1767	0.0053
489	-1.1793	-0.0488	0.0024	1.001	0.655	174638.6	0.0017	0.1707	-0.0488
490	-0.4824	-0.0199	0.0004	1.003	0.6564	174644.6	0.0017	0.1702	-0.0199
491	-1.5665	-0.0628	0.0039	0.9993	0.6539	174636.1	0.0016	0.1601	-0.0628
492	-0.1806	-0.0075	0.0001	1.0035	0.6567	174640.5	0.0017	0.1746	-0.0075
493	0.009	0.0005	0	1.0036	0.6567	174625.1	0.0018	0.1783	0.0005
494	-0.644	-0.0268	0.0007	1.0028	0.6562	174644.6	0.0017	0.1735	-0.0268
495	-0.5368	-0.0225	0.0005	1.003	0.6564	174644.6	0.0018	0.1765	-0.0225
496	-0.3325	-0.0139	0.0002	1.0033	0.6566	174642.6	0.0018	0.1761	-0.0139
497	0.5253	0.022	0.0005	1.003	0.6564	174611.9	0.0017	0.1748	0.022
498	0.1693	0.0072	0.0001	1.0035	0.6567	174612.9	0.0018	0.1783	0.0072
499	0.5563	0.0236	0.0006	1.003	0.6564	174557.4	0.0018	0.1788	0.0236
500	0.668	0.0285	0.0008	1.0028	0.6562	169675.5	0.0018	0.1815	0.0285
501	0.1892	0.008	0.0001	1.0035	0.6567	174622	0.0018	0.1768	0.008
502	-1.155	-0.0491	0.0024	1.0012	0.6551	174599.9	0.0018	0.1803	-0.0491
503	1.3652	0.0578	0.0033	1.0002	0.6544	174328.4	0.0018	0.1794	0.0578
504	1.5395	0.0649	0.0042	0.9993	0.6538	174413.1	0.0018	0.178	0.0649
505	0.6413	0.0273	0.0007	1.0029	0.6562	174441.3	0.0018	0.1802	0.0273

506	0.2097	0.0087	0.0001	1.0034	0.6566	174634.4	0.0017	0.1707	0.0087	
507	0.7468	0.0319	0.001	1.0026	0.6561	173738.3	0.0018	0.1812	0.0319	
508	-0.5769	-0.023	0.0005	1.0027	0.6562	174644.7	0.0016	0.1588	-0.023	
509	0.2726	0.0115	0.0001	1.0034	0.6566	174622.7	0.0018	0.1755	0.0115	
510	0.1492	0.0063	0	1.0035	0.6567	174628.3	0.0018	0.1756	0.0063	
511	0.481	0.0204	0.0004	1.0032	0.6564	174591.7	0.0018	0.1777	0.0204	
512	0.2689	0.0113	0.0001	1.0034	0.6566	174626.7	0.0017	0.1743	0.0113	
513	-0.3874	-0.0164	0.0003	1.0034	0.6566	174539.1	0.0018	0.1815	-0.0164	
514	1.0849	0.0442	0.002	1.0014	0.6553	174612.6	0.0017	0.1661	0.0442	
515	-3.3722	-0.1448	0.0206	0.9832	0.6428	79344.82	0.0018	0.1815	-0.1447	*
516	0.8276	0.0347	0.0012	1.0023	0.6559	174591.1	0.0017	0.1748	0.0347	
517	-0.3015	-0.0127	0.0002	1.0034	0.6566	174640.3	0.0018	0.1782	-0.0127	
518	-0.4096	-0.0172	0.0003	1.0032	0.6565	174643.6	0.0018	0.1766	-0.0172	
519	0.3015	0.0127	0.0002	1.0033	0.6566	174626.7	0.0017	0.1737	0.0127	
520	-1.0582	-0.0443	0.002	1.0015	0.6554	174638.7	0.0017	0.1747	-0.0443	
521	0.2893	0.0117	0.0001	1.0031	0.6565	174638	0.0016	0.162	0.0117	
522	0.2006	0.0085	0.0001	1.0035	0.6567	174618.7	0.0018	0.1772	0.0085	
523	2.0322	0.086	0.0073	0.9962	0.6517	173976.5	0.0018	0.1797	0.086	
524	0.989	0.042	0.0018	1.0018	0.6555	174411.6	0.0018	0.1796	0.042	
525	-0.8298	-0.0344	0.0012	1.0023	0.6559	174643.5	0.0017	0.1716	-0.0344	
526	0.9992	0.0418	0.0017	1.0018	0.6555	174580.1	0.0017	0.1745	0.0418	
527	1.1844	0.05	0.0025	1.0011	0.655	174489	0.0018	0.1779	0.05	
528	1.9109	0.0658	0.0043	0.998	0.6533	174628.1	0.0012	0.1192	0.0658	
529	0.5798	0.0245	0.0006	1.003	0.6563	174590.7	0.0018	0.177	0.0245	
530	2.8014	0.1176	0.0137	0.9897	0.6473	174140.1	0.0018	0.1775	0.1176	
531	-0.7249	-0.0306	0.0009	1.0026	0.6561	174643.5	0.0018	0.1788	-0.0306	
532	-1.7458	-0.0707	0.005	0.9983	0.6532	174631.1	0.0016	0.163	-0.0707	
533	-0.1604	-0.0065	0	1.0033	0.6566	174642.5	0.0017	0.1678	-0.0065	
534	-1.2891	-0.0496	0.0025	1.0005	0.6548	174641.7	0.0015	0.1473	-0.0496	
535	-1.3316	-0.0563	0.0032	1.0004	0.6545	174614.8	0.0018	0.1782	-0.0563	
536	-0.5554	-0.0234	0.0005	1.003	0.6563	174644.7	0.0018	0.1783	-0.0234	
537	-0.0149	-0.0005	0	1.0035	0.6567	174638	0.0017	0.173	-0.0005	
538	-0.5485	-0.0233	0.0005	1.0031	0.6564	174641.5	0.0018	0.1814	-0.0233	
539	-0.9159	-0.0389	0.0015	1.0021	0.6557	174633.2	0.0018	0.1799	-0.0389	

540	1.175	0.05	0.0025	1.0011	0.655	172559.3	0.0018	0.1813	0.05	
541	0.1771	0.0074	0.0001	1.0034	0.6566	174633.9	0.0017	0.172	0.0074	
542	-5.4589	-0.2356	0.0526	0.9517	0.6213	153512.9	0.0018	0.1814	-0.2354	*
543	-0.3901	-0.0159	0.0003	1.0031	0.6565	174644.3	0.0017	0.1662	-0.0159	
544	-0.5274	-0.0221	0.0005	1.003	0.6564	174644.6	0.0018	0.1758	-0.0221	
545	0.3458	0.0147	0.0002	1.0034	0.6566	174596.4	0.0018	0.1783	0.0147	
546	-0.4004	-0.0169	0.0003	1.0033	0.6565	174641.4	0.0018	0.1796	-0.0169	
547	-0.7675	-0.0318	0.001	1.0024	0.656	174644.1	0.0017	0.1715	-0.0318	
548	-0.3034	-0.0129	0.0002	1.0035	0.6566	174080.5	0.0018	0.1815	-0.0129	
549	-1.7968	-0.0764	0.0058	0.9978	0.6528	174518.4	0.0018	0.1795	-0.0764	
550	0.7581	0.0322	0.001	1.0026	0.656	174430.4	0.0018	0.18	0.0322	
551	-0.492	-0.0208	0.0004	1.0032	0.6564	174644	0.0018	0.1793	-0.0208	
552	-0.4673	-0.0195	0.0004	1.0031	0.6564	174644.3	0.0018	0.1752	-0.0195	
553	-0.6872	-0.0288	0.0008	1.0027	0.6561	174644.3	0.0018	0.1764	-0.0288	
554	-0.9449	-0.0384	0.0015	1.0018	0.6556	174643.1	0.0017	0.1653	-0.0384	
555	0.0047	0.0003	0	1.0036	0.6567	174630.2	0.0018	0.1772	0.0003	
556	-0.3035	-0.0129	0.0002	1.0035	0.6566	174600.1	0.0018	0.1812	-0.0129	
557	-0.6695	-0.0285	0.0008	1.0028	0.6562	174640.6	0.0018	0.1813	-0.0285	
558	-1.3762	-0.0581	0.0034	1.0002	0.6544	174618.1	0.0018	0.1773	-0.0581	
559	0.1411	0.0061	0	1.0036	0.6567	174578.2	0.0018	0.1801	0.0061	
560	1.7597	0.0736	0.0054	0.9981	0.653	174481.3	0.0018	0.1755	0.0736	
561	0.4964	0.0212	0.0005	1.0032	0.6564	172973.1	0.0018	0.1814	0.0212	
562	-0.2389	-0.0101	0.0001	1.0035	0.6567	174612.9	0.0018	0.1808	-0.0101	
563	0.262	0.0104	0.0001	1.003	0.6565	174640.3	0.0015	0.1545	0.0104	
564	0.0378	0.0016	0	1.0034	0.6567	174637.8	0.0017	0.1716	0.0016	
565	-1.7844	-0.0758	0.0057	0.9979	0.6528	174531.6	0.0018	0.1793	-0.0758	
566	-1.2373	-0.0524	0.0027	1.0008	0.6548	174616.2	0.0018	0.1789	-0.0524	
567	2.1381	0.0871	0.0075	0.9957	0.6514	174555	0.0017	0.1668	0.0871	
568	0.2125	0.0086	0.0001	1.0031	0.6566	174639.8	0.0016	0.1598	0.0086	
569	-0.9205	-0.0387	0.0015	1.002	0.6557	174640.3	0.0018	0.177	-0.0387	

Supplementary Table 5. Univariate and multi-variable meta-regression.

Variable	Results
Country development	Mixed-Effects Model (k = 571; tau^2 estimator: DL)<br><br>tau^2 (estimated amount of residual heterogeneity): 0.0037 (SE = 0.0011)<br>tau (square root of estimated tau^2 value): 0.0611<br>I^2 (residual heterogeneity / unaccounted variability): 98.71%<br>H^2 (unaccounted variability / sampling variability): 77.46<br>R^2 (amount of heterogeneity accounted for): 2.25%<br><br>Test for Residual Heterogeneity:<br>QE(df = 569) = 44076.7958, p-val < .0001<br><br>Test of Moderators (coefficient 2):<br>F(df1 = 1, df2 = 569) = 29.8930, p-val < .0001<br><br>Model Results:<br><br>estimate se tval pval ci.lb ci.ub<br>intrcpt 0.1619 0.0069 23.6235 <.0001 0.1485 0.1754 ***

	develop 0.0522 0.0096 5.4674 <.0001 0.0335 0.0710 ***<br><br>---<br>Signif. codes: 0 ‘***’ 0.001 ‘**’ 0.01 ‘*’ 0.05 ‘.’ 0.1 ‘ ’ 1
Continents	Mixed-Effects Model (k = 571; tau^2 estimator: DL)<br><br>tau^2 (estimated amount of residual heterogeneity): 0.0038 (SE = 0.0011)<br>tau (square root of estimated tau^2 value): 0.0620<br>I^2 (residual heterogeneity / unaccounted variability): 98.74%<br>H^2 (unaccounted variability / sampling variability): 79.40<br>R^2 (amount of heterogeneity accounted for): 0.00%<br><br>Test for Residual Heterogeneity:<br>QE(df = 569) = 45177.7704, p-val < .0001<br><br>Test of Moderators (coefficient 2):<br>F(df1 = 1, df2 = 569) = 1.0210, p-val = 0.3127<br><br>Model Results:

	estimate se tval pval ci.lb ci.ub<br>intrcpt 0.1782 0.0118 15.1483 <.0001 0.1551 0.2013 ***<br>continent 0.0011 0.0011 1.0104 0.3127 -0.0010 0.0031<br><br>---<br>Signif. codes: 0 ‘***’ 0.001 ‘**’ 0.01 ‘*’ 0.05 ‘.’ 0.1 ‘ ’ 1
**Diagnostic technique**	**Number of studies combined: k = 571**<br><br>**proportion 95%-CI**<br>**Random effects model 0.1744 [0.1673; 0.1817]**<br><br>**Quantifying heterogeneity:**<br>**tau^2 = 0.3435 [<0.0000; <0.0000]; tau = 0.5861 [<0.0000; <0.0000];**<br>**I^2 = 98.8% [98.8%; 98.8%]; H = 9.09 [8.95; 9.24]**<br><br>**Quantifying residual heterogeneity:**<br>**I^2 = 98.6% [98.6%; 98.7%]; H = 8.51 [8.37; 8.65]**<br><br>**Test of heterogeneity:**<br>**Q d.f. p-value**

	47120.25 570 0

Results for subgroups (random effects model):

	k	proportion	95%-CI	tau^2	tau	Q	I^2
meas = Others	22	0.1494	[0.1306; 0.1703]	0.1095	0.3308	543.63	96.1%
meas = DSM-IV	7	0.1294	[0.0914; 0.1800]	0.2434	0.4933	70.32	91.5%
meas = SRQ	12	0.2590	[0.2116; 0.3127]	0.2073	0.4553	472.38	97.7%
meas = PHQ-9	19	0.1859	[0.1211; 0.2744]	1.2283	1.1083	2108.75	99.1%
meas = EPDS	464	0.1686	[0.1604; 0.1772]	0.4049	0.6364	35406.40	98.7%
meas = CES-D	13	0.2506	[0.1955; 0.3150]	0.3226	0.5680	537.47	97.8%
meas = BDI	10	0.2970	[0.2307; 0.3731]	0.2579	0.5079	84.37	89.3%
meas = DASS	4	0.1847	[0.1643; 0.2070]	0.0139	0.1180	10.86	72.4%
meas = SCID	5	0.1011	[0.0375; 0.2448]	1.4250	1.1937	355.45	98.9%
meas = ICD-10	1	0.1144	[0.1130; 0.1158]	--	--	0.00	--
meas = PDSS	5	0.3723	[0.2147; 0.5627]	0.7512	0.8667	158.89	97.5%
meas = SDS	4	0.2817	[0.2130; 0.3624]	0.1283	0.3582	73.12	95.9%
meas = PHQ-2	5	0.1847	[0.1442; 0.2334]	0.1095	0.3308	595.50	99.3%

Test for subgroup differences (random effects model):

Q d.f. p-value

	**Between groups 465.06 12 < 0.0001**<br><br>**Details on meta-analytical method:**<br>**- Inverse variance method**<br>**- DerSimonian-Laird estimator for tau^2**<br>**- Jackson method for confidence interval of tau^2 and tau**<br>**- Logit transformation**
**Country or regional income**	**Mixed-Effects Model (k = 571; tau^2 estimator: DL)**<br><br>**tau^2 (estimated amount of residual heterogeneity): 0.0039 (SE = 0.0011)**<br>**tau (square root of estimated tau^2 value): 0.0623**<br>**I^2 (residual heterogeneity / unaccounted variability): 98.76%**<br>**H^2 (unaccounted variability / sampling variability): 80.36**<br>**R^2 (amount of heterogeneity accounted for): 0.00%**<br><br>**Test for Residual Heterogeneity:**<br>**QE(df = 569) = 45724.6180, p-val < .0001**<br><br>**Test of Moderators (coefficient 2):**<br>**F(df1 = 1, df2 = 569) = 11.2399, p-val = 0.0009**

	**Model Results:**<br><br>estimate se tval pval ci.lb ci.ub<br>intrcpt 0.1562 0.0110 14.2483 <.0001 0.1346 0.1777 ***<br>income 0.0225 0.0067 3.3526 0.0009 0.0093 0.0357 ***<br><br>---<br>Signif. codes: 0 ‘***’ 0.001 ‘**’ 0.01 ‘*’ 0.05 ‘.’ 0.1 ‘ ’ 1
**Publication time**	**Mixed-Effects Model (k = 571; tau^2 estimator: DL)**<br><br>tau^2 (estimated amount of residual heterogeneity): 0.0039 (SE = 0.0011)<br>tau (square root of estimated tau^2 value): 0.0622<br>I^2 (residual heterogeneity / unaccounted variability): 98.76%<br>H^2 (unaccounted variability / sampling variability): 80.68<br>R^2 (amount of heterogeneity accounted for): 0.00%<br><br>Test for Residual Heterogeneity:<br>QE(df = 569) = 45908.3548, p-val < .0001

	**Test of Moderators (coefficient 2):**<br>**F(df1 = 1, df2 = 569) = 0.2000, p-val = 0.6549**<br><br>**Model Results:**<br><br>**estimate se tval pval ci.lb ci.ub**<br>**intrcpt 0.1980 0.0205 9.6569 <.0001 0.1577 0.2382 *****<br>**public -0.0051 0.0114 -0.4472 0.6549 -0.0274 0.0172**<br><br>**---**<br>**Signif. codes: 0 '***' 0.001 '**' 0.01 '*' 0.05 '.' 0.1 ' ' 1**
**Study quality**	**Mixed-Effects Model (k = 571; tau^2 estimator: DL)**<br><br>**tau^2 (estimated amount of residual heterogeneity): 0.0038 (SE = 0.0011)**<br>**tau (square root of estimated tau^2 value): 0.0620**<br>**I^2 (residual heterogeneity / unaccounted variability): 98.76%**<br>**H^2 (unaccounted variability / sampling variability): 80.41**<br>**R^2 (amount of heterogeneity accounted for): 0.00%**<br><br>**Test for Residual Heterogeneity:**

	**QE(df = 569) = 45752.3485, p-val < .0001**<br><br>**Test of Moderators (coefficient 2):**<br>**F(df1 = 1, df2 = 569) = 0.3618, p-val = 0.5477**<br><br>**Model Results:**<br><br>**estimate se tval pval ci.lb ci.ub**<br>**intrcpt 0.1634 0.0429 3.8110 0.0002 0.0792 0.2476 ******<br>**quality 0.0132 0.0219 0.6015 0.5477 -0.0298 0.0561**<br><br>**---**<br>**Signif. codes: 0 ‘***’ 0.001 ‘**’ 0.01 ‘*’ 0.05 ‘.’ 0.1 ‘ ’ 1**
**Study size**	**Mixed-Effects Model (k = 571; tau^2 estimator: DL)**<br><br>**tau^2 (estimated amount of residual heterogeneity): 0.0037 (SE = 0.0011)**<br>**tau (square root of estimated tau^2 value): 0.0609**<br>**I^2 (residual heterogeneity / unaccounted variability): 98.72%**<br>**H^2 (unaccounted variability / sampling variability): 77.91**<br>**R^2 (amount of heterogeneity accounted for): 2.97%**

	Test for Residual Heterogeneity:<br>QE(df = 569) = 44329.2063, p-val < .0001<br><br>Test of Moderators (coefficient 2):<br>F(df1 = 1, df2 = 569) = 32.1439, p-val < .0001<br><br>Model Results:<br><br>estimate se tval pval ci.lb ci.ub<br>intrcpt 0.2655 0.0143 18.5135 <.0001 0.2373 0.2936 ***<br>size -0.0592 0.0104 -5.6696 <.0001 -0.0797 -0.0387 ***<br><br>---<br>Signif. codes: 0 ‘***’ 0.001 ‘**’ 0.01 ‘*’ 0.05 ‘.’ 0.1 ‘ ’ 1
Study period	proportion 95%-CI<br>Random effects model 0.1606 [0.1496; 0.1723]<br><br>Quantifying heterogeneity:<br>tau^2 = 1.0560 [<0.0000; <0.0000]; tau = 1.0276 [<0.0000; <0.0000];

I^2 = 99.6% [99.6%; 99.6%]; H = 15.66 [15.49; 15.83]

Quantifying residual heterogeneity:

I^2 = 99.5% [99.5%; 99.5%]; H = 14.47 [14.31; 14.64]

Test of heterogeneity:

Q	d.f.	p-value
144702.08	590	0

Results for subgroups (random effects model):

k proportion 95%-CI tau^2 tau Q I^2

screen = 2 412 0.1531 [0.1431; 0.1636] 0.6450 0.8031 45795.44 99.1% 86745 1239690

screen = 3 67 0.1819 [0.1356; 0.2396] 2.0826 1.4431 71257.34 99.9% 55147 1560862

screen = 1 97 0.1770 [0.1595; 0.1960] 0.3642 0.6035 5472.61 98.2% 23639 179923

screen = 4 15 0.1795 [0.1380; 0.2301] 0.3437 0.5862 410.88 96.6% 1781 11374

Test for subgroup differences (random effects model):

	Q	d.f.	p-value
Between groups	6.74	3	0.0807

	**Details on meta-analytical method:**<br><br>**- Inverse variance method**<br><br>**- DerSimonian-Laird estimator for tau^2**<br><br>**- Jackson method for confidence interval of tau^2 and tau**<br><br>**- Logit transformation**
**Multi-variable meta-regression**	**Multimodel Inference: Final Results**<br><br>**--------------------------**<br><br>**- Number of fitted models: 128**<br><br>**- Full formula: ~ develop + meanss + income + public + quality + continent + size**<br><br>**- Coefficient significance test: knha**<br><br>**- Interactions modeled: no**<br><br>**- Evaluation criterion: AICc**<br><br>**Best 5 Models**<br><br>**--------------------------**<br><br>**Global model call: metafor::rma(yi = TE, sei = seTE, mods = form, data = glm.data,**

```
    method = method, test = test)
---
Model selection table
   (Intrc)      cntnn      devlp      menss       publc      qulty        size df   logLik
67         +               0.04075                                       -0.05833   4 417.494
68         + 0.0005807 0.04042                                          -0.05820   5 416.398
75         +               0.04002 0.001407                              -0.05890   5 416.365
99         +               0.04073                         0.008371 -0.05809   5 416.305
83         +               0.04100               -0.002788               -0.05797   5 416.272
     AICc delta weight
67 -826.9   0.00   0.688
68 -822.7   4.23   0.083
75 -822.6   4.29   0.080
99 -822.5   4.42   0.076
83 -822.4   4.48   0.073
Models ranked by AICc(x)



Multimodel Inference Coefficients
-------------------------
```

Estimate Std. Error z value Pr(>|z|)

intrcpt 2.459378e-01 0.0236240879 10.41046746 0.0000000

develop 4.062991e-02 0.0103875810 3.91139273 0.0000918

size -5.835051e-02 0.0110220664 5.29397154 0.0000001

continent 6.359389e-05 0.0003909129 0.16268046 0.8707700

meanss 1.501456e-04 0.0010309626 0.14563631 0.8842085

quality 8.274048e-04 0.0073435042 0.11267166 0.9102909

public -2.695695e-04 0.0036118702 0.07463432 0.9405057

income -1.033980e-04 0.0027730652 0.03728653 0.9702565

Predictor Importance

----

model importance

1 size 0.99999408

2 develop 0.99626514

	**3 continent 0.10798448**<br>**4 meanss 0.10493701**<br>**5 quality 0.09895461**<br>**6 income 0.09650220**<br>**7 public 0.09619530**<br>**>**

Supplementary Figure 1. Sensitivity analysis by using a build-in function.

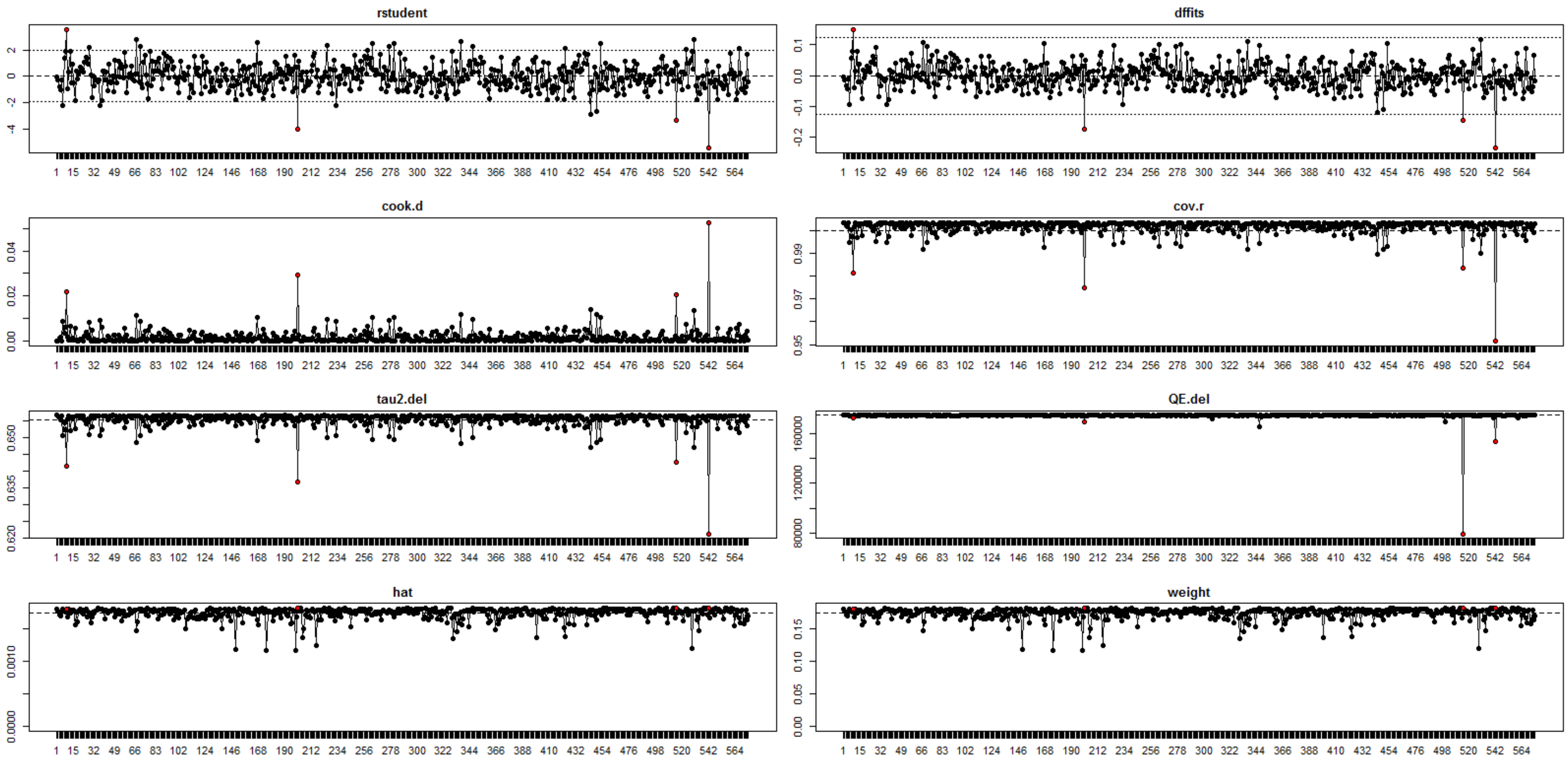

Reference

1. Ramchandani PG, Richter LM, Stein A, Norris SA. Predictors of postnatal depression in an urban South African cohort. Journal of affective disorders 2009; 113(3): 279-84.

2. Stewart RC, Bunn J, Vokhiwa M, et al. Common mental disorder and associated factors amongst women with young infants in rural Malawi. Social psychiatry and psychiatric epidemiology 2010; 45(5): 551-9.

3. Ndokera R, MacArthur C. The relationship between maternal depression and adverse infant health outcomes in Zambia: a cross-sectional feasibility study. Child: care, health and development 2011; 37(1): 74-81.

4. Guo N, Bindt C, Te Bonle M, et al. Association of antepartum and postpartum depression in Ghanaian and Ivorian women with febrile illness in their offspring: a prospective birth cohort study. American journal of epidemiology 2013; 178(9): 1394-402.

5. Weobong B, Ten Asbroek AH, Soremekun S, et al. Determinants of postnatal depression in rural ghana: findings from the don population based cohort study. Depression and anxiety 2015; 32(2): 108-19.

6. Hassanein IM, Fathalla MM, Abdel Rahim T. The role of newborn gender in postpartum depressive symptoms among women in Upper Egypt. International journal of gynaecology and obstetrics: the official organ of the International Federation of Gynaecology and Obstetrics 2014; 125(2): 138-40.

7. Mohammed ES, Mosalem FA, Mahfouz EM, Abd ElHameed MA. Predictors of postpartum depression among rural women in Minia, Egypt: an epidemiological study. Public health 2014; 128(9): 817-24.

8. Mahenge B, Stockl H, Likindikoki S, Kaaya S, Mbwambo J. The prevalence of mental health morbidity and its associated factors among women attending a prenatal clinic in Tanzania. International journal of gynaecology and obstetrics: the official organ of the International Federation of Gynaecology and Obstetrics 2015; 130(3): 261-5.

9. Khalifa DS, Glavin K, Bjertness E, Lien L. Postnatal depression among Sudanese women: prevalence and validation of the Edinburgh Postnatal Depression Scale at 3 months postpartum. International journal of women's health 2015; 7: 677-84.

10. Shamu S, Zarowsky C, Roelens K, Temmerman M, Abrahams N. High-frequency intimate partner violence during pregnancy, postnatal depression and suicidal tendencies in Harare, Zimbabwe. General hospital psychiatry 2016; 38: 109-14.

11. Stellenberg EL, Abrahams JM. Prevalence of and factors influencing postnatal depression in a rural community in South Africa. African journal of primary health care & family medicine 2015; 7(1): 874.

12. Azale T, Fekadu A, Hanlon C. Treatment gap and help-seeking for postpartum depression in a rural African setting. BMC psychiatry 2016; 16: 196.

13. Wemakor A, Mensah KA. Association between maternal depression and child stunting in Northern Ghana: a cross-sectional study. BMC public health 2016; 16(1): 869.

14. Rogathi JJ, Manongi R, Mushi D, et al. Postpartum depression among women who have experienced intimate partner violence: A prospective cohort study at Moshi, Tanzania. Journal of affective disorders 2017; 218: 238-45.

15. Anokye R, Acheampong E, Budu-Ainooson A, Obeng EI, Akwasi AG. Prevalence of postpartum depression and interventions utilized for its management. Annals of general psychiatry 2018; 17: 18.

16. Glasser S, Barell V, Boyko V, et al. Postpartum depression in an Israeli cohort: demographic, psychosocial and medical risk factors. Journal of psychosomatic obstetrics and gynaecology 2000; 21(2): 99-108.

17. Yoshida K, Yamashita H, Ueda M, Tashiro N. Postnatal depression in Japanese mothers and the reconsideration of 'Satogaeri bunben'. Pediatrics international : official journal of the Japan Pediatric Society 2001; 43(2): 189-93.

18. Chandran M, Tharyan P, Muliyil J, Abraham S. Post-partum depression in a cohort of women from a rural area of Tamil Nadu, India. Incidence and risk factors. The British journal of psychiatry : the journal of mental science 2002; 181: 499-504.

19. Inandi T, Elci OC, Ozturk A, Egri M, Polat A, Sahin TK. Risk factors for depression in postnatal first year, in eastern Turkey. International journal of epidemiology 2002; 31(6): 1201-7.

20. Koo V, Lynch J, Cooper S. Risk of postnatal depression after emergency delivery. The journal of obstetrics and gynaecology research 2003; 29(4): 246-50.

21. Wang SY, Jiang XY, Jan WC, Chen CH. A comparative study of postnatal depression and its predictors in Taiwan and mainland China. American journal of obstetrics and gynecology 2003; 189(5): 1407-12.

22. Heh SS, Coombes L, Bartlett H. The association between depressive symptoms and social support in Taiwanese women during the month. International journal of nursing studies 2004; 41(5): 573-9.

23. Fisher JR, Morrow MM, Ngoc NT, Anh LT. Prevalence, nature, severity and correlates of postpartum depressive symptoms in Vietnam. BJOG : an international journal of obstetrics and gynaecology 2004; 111(12): 1353-60.

24. Aydin N, Inandi T, Karabulut N. Depression and associated factors among women within their first postnatal year in Erzurum province in eastern Turkey. Women & health 2005; 41(2): 1-12.

25. Wang SY, Chen CH. Psychosocial health of Taiwanese postnatal husbands and wives. Journal of psychosomatic research 2006; 60(3): 303-7.

26. Chien LY, Tai CJ, Ko YL, Huang CH, Sheu SJ. Adherence to "Doing-the-month" practices is associated with fewer physical and depressive symptoms among postpartum women in Taiwan. Research in nursing & health 2006; 29(5): 374-83.

27. Rahman A, Creed F. Outcome of prenatal depression and risk factors associated with persistence in the first postnatal year: prospective study from Rawalpindi, Pakistan. Journal of affective disorders 2007; 100(1-3): 115-21.

28. Green K, Broome H, Mirabella J. Postnatal depression among mothers in the United Arab Emirates: socio-cultural and physical factors. Psychology, health & medicine 2006; 11(4): 425-31.

29. Ho-Yen SD, Bondevik GT, Eberhard-Gran M, Bjorvatn B. Factors associated with depressive symptoms among postnatal women in Nepal. Acta obstetricia et gynecologica Scandinavica 2007; 86(3): 291-7.

30. Xie RH, He G, Liu A, Bradwejn J, Walker M, Wen SW. Fetal gender and postpartum depression in a cohort of Chinese women. Social science & medicine 2007; 65(4): 680-4.

31. Liabsuetrakul T, Vittayanont A, Pitanupong J. Clinical applications of anxiety, social support, stressors, and self-esteem measured during pregnancy and postpartum for screening postpartum depression in Thai women. The journal of obstetrics and gynaecology research 2007; 33(3): 333-40.

32. Chee CY, Chong YS, Ng TP, Lee DT, Tan LK, Fones CS. The association between maternal depression and frequent non-routine visits to the infant's doctor--a cohort study. Journal of affective disorders 2008; 107(1-3): 247-53.

33. Murakami K, Miyake Y, Sasaki S, et al. Dietary glycemic index and load and the risk of postpartum depression in Japan: the Osaka Maternal and Child Health Study. Journal of affective disorders 2008; 110(1-2): 174-9.

34. Horowitz JA, Murphy CA, Gregory KE, Wojcik J. A community-based screening initiative to identify mothers at risk for postpartum depression. Journal of obstetric, gynecologic, and neonatal nursing : JOGNN 2011; 40(1): 52-61.

35. Mohamad Yusuff AS, Tang L, Binns CW, Lee AH. Prevalence and risk factors for postnatal depression in Sabah, Malaysia: a cohort study. Women and birth : journal of the Australian College of Midwives 2015; 28(1): 25-9.

36. Ogbo FA, Eastwood J, Hendry A, et al. Determinants of antenatal depression and postnatal depression in Australia. BMC psychiatry 2018; 18(1): 49.

37. Ahmad NA, Silim UA, Rosman A, et al. Postnatal depression and intimate partner violence: a nationwide clinic-based cross-sectional study in Malaysia. BMJ open 2018; 8(5): e020649.

38. Wassif OM, Abdo AS, Elawady MA, Abd Elmaksoud AE, Eldesouky RS. Assessment of Postpartum Depression and Anxiety among Females Attending Primary Health Care Facilities in Qaliubeya Governorate, Egypt. Journal of environmental and public health 2019; 2019: 3691752.

39. Gausia K, Fisher C, Ali M, Oosthuizen J. Magnitude and contributory factors of postnatal depression: a community-based cohort study from a rural subdistrict of Bangladesh. Psychological medicine 2009; 39(6): 999-1007.

40. Gao LL, Chan SW, Mao Q. Depression, perceived stress, and social support among first-time Chinese mothers and fathers in the postpartum period. Research in nursing & health 2009; 32(1): 50-8.

41. Yagmur Y, Ulukoca N. Social support and postpartum depression in low-socioeconomic level postpartum women in Eastern Turkey. International journal of public health 2010; 55(6): 543-9.

42. McMahon CA, Boivin J, Gibson FL, et al. Older first-time mothers and early postpartum depression: a prospective cohort study of women conceiving spontaneously or with assisted reproductive technologies. Fertility and sterility 2011; 96(5): 1218-24.

43. Zhang Y, Zou S, Cao Y, Zhang Y. Relationship between domestic violence and postnatal depression among pregnant Chinese women. International journal of gynaecology and obstetrics: the official organ of the International Federation of Gynaecology and Obstetrics 2012; 116(1): 26-30.

44. Bowen A, Bowen R, Butt P, Rahman K, Muhajarine N. Patterns of depression and treatment in pregnant and postpartum women. Canadian journal of psychiatry Revue canadienne de psychiatrie 2012; 57(3): 161-7.

45. Mishina H, Yamamoto Y, Ito M. Regional variations in prevalence of postpartum depressive symptoms: population-based survey. Pediatrics international : official journal of the Japan Pediatric Society 2012; 54(4): 563-5.

46. Bener A, Burgut FT, Ghuloum S, Sheikh J. A study of postpartum depression in a fast developing country: prevalence and related factors. International journal of psychiatry in medicine 2012; 43(4): 325-37.

47. Vigod SN, Tarasoff LA, Bryja B, Dennis CL, Yudin MH, Ross LE. Relation between place of residence and postpartum depression. CMAJ : Canadian Medical Association journal = journal de l'Association medicale canadienne 2013; 185(13): 1129-35.

48. Mathisen SE, Glavin K, Lien L, Lagerlov P. Prevalence and risk factors for postpartum depressive symptoms in Argentina: a cross-sectional study. International journal of women's health 2013; 5: 787-93.

49. Gaillard A, Le Strat Y, Mandelbrot L, Keita H, Dubertret C. Predictors of postpartum depression: prospective study of 264 women followed during pregnancy and postpartum. Psychiatry research 2014; 215(2): 341-6.

50. Deng AW, Xiong RB, Jiang TT, Luo YP, Chen WZ. Prevalence and risk factors of postpartum depression in a population-based sample of women in Tangxia Community, Guangzhou. Asian Pacific journal of tropical medicine 2014; 7(3): 244-9.

51. Al Hinai FI, Al Hinai SS. Prospective study on prevalence and risk factors of postpartum depression in Al-dakhliya governorate in oman. Oman medical journal 2014; 29(3): 198-202.

52. Kirkan TS, Aydin N, Yazici E, Aslan PA, Acemoglu H, Daloglu AG. The depression in women in pregnancy and postpartum period: A follow-up study. The International journal of social psychiatry 2015; 61(4): 343-9.

53. Verreault N, Da Costa D, Marchand A, Ireland K, Dritsa M, Khalife S. Rates and risk factors associated with depressive symptoms during pregnancy and with postpartum onset. Journal of psychosomatic obstetrics and gynaecology 2014; 35(3): 84-91.

54. Giri RK, Khatri RB, Mishra SR, Khanal V, Sharma VD, Gartoula RP. Prevalence and factors associated with depressive symptoms among post-partum mothers in Nepal. BMC research notes 2015; 8: 111.

55. Shimizu A, Nishiumi H, Okumura Y, Watanabe K. Depressive symptoms and changes in physiological and social factors 1 week to 4 months postpartum in Japan. Journal of affective disorders 2015; 179: 175-82.

56. Turkcapar AF, Kadioglu N, Aslan E, Tunc S, Zayifoglu M, Mollamahmutoglu L. Sociodemographic and clinical features of postpartum depression among Turkish women: a prospective study. BMC pregnancy and childbirth 2015; 15: 108.

57. Bolak Boratav H, Toker O, Kuey L. Postpartum depression and its psychosocial correlates: A longitudinal study among a group of women in Turkey. Women & health 2016; 56(5): 502-21.

58. Kumwar D, Corey EK, Sharma P, Risal A. Screening for Postpartum Depression and Associated Factors among Women who Deliver at a University Hospital, Nepal. Kathmandu University medical journal 2015; 13(49): 44-8.

59. Zainal NZ, Kaka AS, Ng CG, Jawan R, Singh Gill J. Prevalence of postpartum depression in a hospital setting among Malaysian mothers. Asia-Pacific psychiatry : official journal of the Pacific Rim College of Psychiatrists 2012; 4(2): 144-9.

60. Rebelo F, Farias DR, Struchiner CJ, Kac G. Plasma adiponectin and depressive symptoms during pregnancy and the postpartum period: A prospective cohort study. Journal of affective disorders 2016; 194: 171-9.

61. Correa H, Castro ECT, Santos W, Romano-Silva MA, Santos LM. Postpartum depression symptoms among Amazonian and Northeast Brazilian women. Journal of affective disorders 2016; 204: 214-8.

62. Bhusal BR, Bhandari N, Chapagai M, Gavidia T. Validating the Edinburgh Postnatal Depression Scale as a screening tool for postpartum depression in Kathmandu, Nepal. International journal of mental health systems 2016; 10: 71.

63. Chi X, Zhang P, Wu H, Wang J. Screening for Postpartum Depression and Associated Factors Among Women in China: A Cross-Sectional Study. Frontiers in psychology 2016; 7: 1668.

64. Jaeschke RR, Dudek D, Topor-Madry R, et al. Postpartum depression: bipolar or unipolar? Analysis of 434 Polish postpartum women. Revista brasileira de psiquiatria 2017; 39(2): 154-9.

65. Koutra K, Vassilaki M, Georgiou V, et al. Pregnancy, perinatal and postpartum complications as determinants of postpartum depression: the Rhea mother-child cohort in Crete, Greece. Epidemiology and psychiatric sciences 2018; 27(3): 244-55.

66. Van Vo T, Hoa TKD, Hoang TD. Postpartum Depressive Symptoms and Associated Factors in Married Women: A Cross-sectional Study in Danang City, Vietnam. Frontiers in public health 2017; 5: 93.

67. Afolabi O, Bunce L, Lusher J, Banbury S. Postnatal depression, maternal-infant bonding and social support: a cross-cultural comparison of Nigerian and British mothers. Journal of mental health 2020; 29(4): 424-30.

68. Eckerdal P, Georgakis MK, Kollia N, Wikstrom AK, Hogberg U, Skalkidou A. Delineating the association between mode of delivery and postpartum depression symptoms: a longitudinal study. Acta obstetricia et gynecologica Scandinavica 2018; 97(3): 301-11.

69. Xiong R, Deng A, Wan B, Liu Y. Prevalence and factors associated with postpartum depression in women from single-child families. International journal of gynaecology and obstetrics: the official organ of the International Federation of Gynaecology and Obstetrics 2018; 141(2): 194-9.

70. Pampaka D, Papatheodorou SI, AlSeaidan M, et al. Postnatal depressive symptoms in women with and without antenatal depressive symptoms: results from a prospective cohort study. Archives of women's mental health 2019; 22(1): 93-103.

71. Shakeel N, Sletner L, Falk RS, et al. Prevalence of postpartum depressive symptoms in a multiethnic population and the role of ethnicity and integration. Journal of affective disorders 2018; 241: 49-58.

72. Kerie S, Menberu M, Niguse W. Prevalence and associated factors of postpartum depression in Southwest, Ethiopia, 2017: a cross-sectional study. BMC research notes 2018; 11(1): 623.

73. Vaezi A, Soojoodi F, Banihashemi AT, Nojomi M. The association between social support and postpartum depression in women: A cross sectional study. Women and birth : journal of the Australian College of Midwives 2019; 32(2): e238-e42.

74. Ongeri L, Wanga V, Otieno P, et al. Demographic, psychosocial and clinical factors associated with postpartum depression in Kenyan women. BMC psychiatry 2018; 18(1): 318.

75. Petersen I, Peltola T, Kaski S, Walters KR, Hardoon S. Depression, depressive symptoms and treatments in women who have recently given birth: UK cohort study. BMJ open 2018; 8(10): e022152.

76. Sahin E, Seven M. Depressive symptoms during pregnancy and postpartum: a prospective cohort study. Perspectives in psychiatric care 2019; 55(3): 430-7.

77. Mohammad KI. Postpartum depression symptoms among Syrian refugee women living in Jordan. Research in nursing & health 2018.

78. Shwartz N, Shoahm-Vardi I, Daoud N. Postpartum depression among Arab and Jewish women in Israel: Ethnic inequalities and risk factors. Midwifery 2019; 70: 54-63.

79. Agbaje OS, Anyanwu JI, Umoke PIC, et al. Depressive and anxiety symptoms and associated factors among postnatal women in Enugu-North Senatorial District, South-East Nigeria: a cross-sectional study. Archives of public health = Archives belges de sante publique 2019; 77: 1.

80. Meky HK, Shaaban MM, Ahmed MR, Mohammed TY. Prevalence of postpartum depression regarding mode of delivery: a cross-sectional study. The journal of maternal-fetal & neonatal medicine : the official journal of the European Association of Perinatal Medicine, the Federation of Asia and Oceania Perinatal Societies, the International Society of Perinatal Obstet 2020; 33(19): 3300-7.

81. Bitew T, Hanlon C, Medhin G, Fekadu A. Antenatal predictors of incident and persistent postnatal depressive symptoms in rural Ethiopia: a population-based prospective study. Reproductive health 2019; 16(1): 28.

82. Nampijja M, Natamba B, Mpango R, Kinyanda E. The burden and risk factors for postnatal depression and depressive symptomatology among women in Kampala. Tropical doctor 2019; 49(3): 170-7.

83. Sharmin KN, Sarwar N, Mumu SJ, Taleb DA, Flora MS. Postnatal depression and infant growth in an urban area of Bangladesh. Midwifery 2019; 74: 57-67.

84. Topatan S, Demirci N. Frequency of Depression and Risk Factors among Adolescent Mothers in Turkey within the First Year of the Postnatal Period. Journal of pediatric and adolescent gynecology 2019; 32(5): 514-9.

85. Azad R, Fahmi R, Shrestha S, et al. Prevalence and risk factors of postpartum depression within one year after birth in urban slums of Dhaka, Bangladesh. PloS one 2019; 14(5): e0215735.

86. Nurbaeti I, Deoisres W, Hengudomsub P. Association between psychosocial factors and postpartum depression in South Jakarta, Indonesia. Sexual & reproductive healthcare : official journal of the Swedish Association of Midwives 2019; 20: 72-6.

87. Lin YH, Chen CM, Su HM, et al. Association between Postpartum Nutritional Status and Postpartum Depression Symptoms. Nutrients 2019; 11(6).

88. Xiong R, Deng A. Incidence and risk factors associated with postpartum depression among women of advanced maternal age from Guangzhou, China. Perspectives in psychiatric care 2020; 56(2): 316-20.

89. Dlamini LP, Mahanya S, Dlamini SD, Shongwe MC. Prevalence and factors associated with postpartum depression at a primary healthcare facility in Eswatini. The South African journal of psychiatry : SAJP : the journal of the Society of Psychiatrists of South Africa 2019; 25(0): 1404.

90. Shitu S, Geda B, Dheresa M. Postpartum depression and associated factors among mothers who gave birth in the last twelve months in Ankesha district, Awi zone, North West Ethiopia. BMC pregnancy and childbirth 2019; 19(1): 435.

91. Arante FO, Tabb KM, Wang Y, Faisal-Cury A. The Relationship Between Postpartum Depression and Lower Maternal Confidence in Mothers with a History of Depression During Pregnancy. The Psychiatric quarterly 2020; 91(1): 21-30.

92. *Roumieh M, Bashour H, Kharouf M, Chaikha S. Prevalence and risk factors for postpartum depression among women seen at Primary Health Care Centres in Damascus. BMC pregnancy and childbirth 2019; 19(1): 519.*

93. *Farias-Antunez S, Santos IS, Matijasevich A, de Barros AJD. Maternal mood symptoms in pregnancy and postpartum depression: association with exclusive breastfeeding in a population-based birth cohort. Social psychiatry and psychiatric epidemiology 2020; 55(5): 635-43.*

94. *Al Nasr RS, Altharwi K, Derbah MS, et al. Prevalence and predictors of postpartum depression in Riyadh, Saudi Arabia: A cross sectional study. PloS one 2020; 15(2): e0228666.*

95. *Xiong R, Deng A. Prevalence and associated factors of postpartum depression among immigrant women in Guangzhou, China. BMC pregnancy and childbirth 2020; 20(1): 247.*

96. *Asaye MM, Muche HA, Zelalem ED. Prevalence and Predictors of Postpartum Depression: Northwest Ethiopia. Psychiatry journal 2020; 2020: 9565678.*

97. *Ueda M, Yamashita H, Yoshida K. Impact of infant health problems on postnatal depression: pilot study to evaluate a health visiting system. Psychiatry and clinical neurosciences 2006; 60(2): 182-9.*

98. *Abbott MW, Williams MM. Postnatal depressive symptoms among Pacific mothers in Auckland: prevalence and risk factors. The Australian and New Zealand journal of psychiatry 2006; 40(3): 230-8.*

99. *Baker L, Cross S, Greaver L, Wei G, Lewis R, Healthy Start C. Prevalence of postpartum depression in a native American population. Maternal and child health journal 2005; 9(1): 21-5.*

100. *Teng HW, Hsu CS, Shih SM, Lu ML, Pan JJ, Shen WW. Screening postpartum depression with the Taiwanese version of the Edinburgh Postnatal Depression scale. Comprehensive psychiatry 2005; 46(4): 261-5.*

101. *Adewuya AO, Fatoye FO, Ola BA, Ijaodola OR, Ibigbami SM. Sociodemographic and obstetric risk factors for postpartum depressive symptoms in Nigerian women. Journal of psychiatric practice 2005; 11(5): 353-8.*

102. *Chee CY, Lee DT, Chong YS, Tan LK, Ng TP, Fones CS. Confinement and other psychosocial factors in perinatal depression: a transcultural study in Singapore. Journal of affective disorders 2005; 89(1-3): 157-66.*

103. *Limlomwongse N, Liabsuetrakul T. Cohort study of depressive moods in Thai women during late pregnancy and 6-8 weeks of postpartum using the Edinburgh Postnatal Depression Scale (EPDS). Archives of women's mental health 2006; 9(3): 131-8.*

104. *Husain N, Bevc I, Husain M, Chaudhry IB, Atif N, Rahman A. Prevalence and social correlates of postnatal depression in a low income country. Archives of women's mental health 2006; 9(4): 197-202.*

105. Jardri R, Pelta J, Maron M, et al. Predictive validation study of the Edinburgh Postnatal Depression Scale in the first week after delivery and risk analysis for postnatal depression. Journal of affective disorders 2006; 93(1-3): 169-76.

106. Azidah AK, Shaiful BI, Rusli N, Jamil MY. Postnatal depression and socio-cultural practices among postnatal mothers in Kota Bahru, Kelantan, Malaysia. The Medical journal of Malaysia 2006; 61(1): 76-83.

107. Miyake Y, Sasaki S, Tanaka K, et al. Dietary folate and vitamins B12, B6, and B2 intake and the risk of postpartum depression in Japan: the Osaka Maternal and Child Health Study. Journal of affective disorders 2006; 96(1-2): 133-8.

108. Huang YC, Mathers NJ. A comparison of sexual satisfaction and post-natal depression in the UK and Taiwan. International nursing review 2006; 53(3): 197-204.

109. Alami KM, Kadri N, Berrada S. Prevalence and psychosocial correlates of depressed mood during pregnancy and after childbirth in a Moroccan sample. Archives of women's mental health 2006; 9(6): 343-6.

110. HO-YEN SD. The prevalence of depressive symptoms in the postnatal period in Lalitpur district, Nepal. Acta Obstetricia et Gynecologica 2006; 85: 1186-92.

111. Pitanupong J, Liabsuetrakul T, Vittayanont A. Validation of the Thai Edinburgh Postnatal Depression Scale for screening postpartum depression. Psychiatry research 2007; 149(1-3): 253-9.

112. Austin MP, Tully L, Parker G. Examining the relationship between antenatal anxiety and postnatal depression. Journal of affective disorders 2007; 101(1-3): 169-74.

113. Andajani-Sutjahjo S, Manderson L, Astbury J. Complex emotions, complex problems: understanding the experiences of perinatal depression among new mothers in urban Indonesia. Culture, medicine and psychiatry 2007; 31(1): 101-22.

114. Loretta Secco M, Profit S, Kennedy E, Walsh A, Letourneau N, Stewart M. Factors affecting postpartum depressive symptoms of adolescent mothers. Journal of obstetric, gynecologic, and neonatal nursing : JOGNN 2007; 36(1): 47-54.

115. Dindar I, Erdogan S. Screening of Turkish women for postpartum depression within the first postpartum year: the risk profile of a community sample. Public health nursing 2007; 24(2): 176-83.

116. Montazeri A, Torkan B, Omidvari S. The Edinburgh Postnatal Depression Scale (EPDS): translation and validation study of the Iranian version. BMC psychiatry 2007; 7: 11.

117. Huang YC, Mathers NJ. Postnatal depression and the experience of South Asian marriage migrant women in Taiwan: survey and semi-structured interview study. International journal of nursing studies 2008; 45(6): 924-31.

118. Figueiredo B, Pacheco A, Costa R. Depression during pregnancy and the postpartum period in adolescent and adult Portuguese mothers. Archives of women's mental health 2007; 10(3): 103-9.

119. Kara B, Unalan P, Cifcili S, Cebeci DS, Sarper N. Is there a role for the family and close community to help reduce the risk of postpartum depression in new mothers? A cross-sectional study of Turkish women. Maternal and child health journal 2008; 12(2): 155-61.

120. Akman C, Uguz F, Kaya N. Postpartum-onset major depression is associated with personality disorders. Comprehensive psychiatry 2007; 48(4): 343-7.

121. Chen CM, Kuo SF, Chou YH, Chen HC. Postpartum Taiwanese women: their postpartum depression, social support and health-promoting lifestyle profiles. Journal of clinical nursing 2007; 16(8): 1550-60.

122. Edge D. Ethnicity, psychosocial risk, and perinatal depression--a comparative study among inner-city women in the United Kingdom. Journal of psychosomatic research 2007; 63(3): 291-5.

123. de Tychey C, Briancon S, Lighezzolo J, et al. Quality of life, postnatal depression and baby gender. Journal of clinical nursing 2008; 17(3): 312-22.

124. Orhon FS, Ulukol B, Soykan A. Postpartum mood disorders and maternal perceptions of infant patterns in well-child follow-up visits. Acta paediatrica 2007; 96(12): 1777-83.

125. Baker L, Oswalt K. Screening for postpartum depression in a rural community. Community mental health journal 2008; 44(3): 171-80.

126. Edwards B, Galletly C, Semmler-Booth T, Dekker G. Does antenatal screening for psychosocial risk factors predict postnatal depression? A follow-up study of 154 women in Adelaide, South Australia. The Australian and New Zealand journal of psychiatry 2008; 42(1): 51-5.

127. Milgrom J, Gemmill AW, Bilszta JL, et al. Antenatal risk factors for postnatal depression: a large prospective study. Journal of affective disorders 2008; 108(1-2): 147-57.

128. Flores-Quijano ME, Cordova A, Contreras-Ramirez V, Farias-Hernandez L, Cruz Tolentino M, Casanueva E. Risk for postpartum depression, breastfeeding practices, and mammary gland permeability. Journal of human lactation : official journal of International Lactation Consultant Association 2008; 24(1): 50-7.

129. Watanabe M, Wada K, Sakata Y, et al. Maternity blues as predictor of postpartum depression: a prospective cohort study among Japanese women. Journal of psychosomatic obstetrics and gynaecology 2008; 29(3): 206-12.

130. Gao W, Paterson J, Abbott M, Carter S, Iusitini L. Pacific Islands families study: intimate partner violence and postnatal depression. Journal of immigrant and minority health 2010; 12(2): 242-8.

131. Krause KM, Ostbye T, Swamy GK. Occurrence and correlates of postpartum depression in overweight and obese women: results from the active mothers postpartum (AMP) study. Maternal and child health journal 2009; 13(6): 832-8.

132. Bjerke SE, Vangen S, Nordhagen R, Ytterdahl T, Magnus P, Stray-Pedersen B. Postpartum depression among Pakistani women in Norway: prevalence and risk factors. The journal of maternal-fetal & neonatal medicine : the official journal of the European Association of Perinatal Medicine, the Federation of Asia and Oceania Perinatal Societies, the International Society of Perinatal Obstet 2008; 21(12): 889-94.

133. Howell EA, Mora PA, DiBonaventura MD, Leventhal H. Modifiable factors associated with changes in postpartum depressive symptoms. Archives of women's mental health 2009; 12(2): 113-20.

134. Figueiredo B, Costa R. Mother's stress, mood and emotional involvement with the infant: 3 months before and 3 months after childbirth. Archives of women's mental health 2009; 12(3): 143-53.

135. Grussu P, Quatraro RM. Prevalence and risk factors for a high level of postnatal depression symptomatology in Italian women: a sample drawn from ante-natal classes. European psychiatry : the journal of the Association of European Psychiatrists 2009; 24(5): 327-33.

136. Glavin K, Smith L, Sorum R. Prevalence of postpartum depression in two municipalities in Norway. Scandinavian journal of caring sciences 2009; 23(4): 705-10.

137. Heron J, Haque S, Oyebode F, Craddock N, Jones I. A longitudinal study of hypomania and depression symptoms in pregnancy and the postpartum period. Bipolar disorders 2009; 11(4): 410-7.

138. Uguz F, Akman C, Sahingoz M, Kaya N, Kucur R. One year follow-up of post-partum-onset depression: the role of depressive symptom severity and personality disorders. Journal of psychosomatic obstetrics and gynaecology 2009; 30(2): 141-5.

139. Satoh A, Kitamiya C, Kudoh H, Watanabe M, Menzawa K, Sasaki H. Factors associated with late post-partum depression in Japan. Japan journal of nursing science : JJNS 2009; 6(1): 27-36.

140. Dorheim SK, Bondevik GT, Eberhard-Gran M, Bjorvatn B. Sleep and depression in postpartum women: a population-based study. Sleep 2009; 32(7): 847-55.

141. Shelton NJ, Herrick KG. Comparison of scoring methods and thresholds of the General Health Questionnaire-12 with the Edinburgh Postnatal Depression Scale in English women. Public health 2009; 123(12): 789-93.

142. Kirpinar I, Gozum S, Pasinlioglu T. Prospective study of postpartum depression in eastern Turkey prevalence, socio-demographic and obstetric correlates, prenatal anxiety and early awareness. Journal of clinical nursing 2010; 19(3-4): 422-31.

143. Escriba-Aguir V, Artazcoz L. Gender differences in postpartum depression: a longitudinal cohort study. Journal of epidemiology and community health 2011; 65(4): 320-6.

144. Blom EA, Jansen PW, Verhulst FC, et al. Perinatal complications increase the risk of postpartum depression. The Generation R Study. BJOG : an international journal of obstetrics and gynaecology 2010; 117(11): 1390-8.

145. Hamdan A, Tamim H. Psychosocial risk and protective factors for postpartum depression in the United Arab Emirates. Archives of women's mental health 2011; 14(2): 125-33.

146. Mohammad KI, Gamble J, Creedy DK. Prevalence and factors associated with the development of antenatal and postnatal depression among Jordanian women. Midwifery 2011; 27(6): e238-45.

147. Lobato G, Moraes CL, Dias AS, Reichenheim ME. Postpartum depression according to time frames and sub-groups: a survey in primary health care settings in Rio de Janeiro, Brazil. Archives of women's mental health 2011; 14(3): 187-93.

148. Sylven SM, Papadopoulos FC, Mpazakidis V, Ekselius L, Sundstrom-Poromaa I, Skalkidou A. Newborn gender as a predictor of postpartum mood disturbances in a sample of Swedish women. Archives of women's mental health 2011; 14(3): 195-201.

149. Ross LE, Villegas L, Dennis CL, et al. Rural residence and risk for perinatal depression: a Canadian pilot study. Archives of women's mental health 2011; 14(3): 175-85.

150. Leahy-Warren P, McCarthy G, Corcoran P. First-time mothers: social support, maternal parental self-efficacy and postnatal depression. Journal of clinical nursing 2012; 21(3-4): 388-97.

151. Figueiredo B, Conde A. Anxiety and depression in women and men from early pregnancy to 3-months postpartum. Archives of women's mental health 2011; 14(3): 247-55.

152. Glasser S, Stoski E, Kneler V, Magnezi R. Postpartum depression among Israeli Bedouin women. Archives of women's mental health 2011; 14(3): 203-8.

153. Lanes A, Kuk JL, Tamim H. Prevalence and characteristics of postpartum depression symptomatology among Canadian women: a cross-sectional study. BMC public health 2011; 11: 302.

154. Matsumoto K, Tsuchiya KJ, Itoh H, et al. Age-specific 3-month cumulative incidence of postpartum depression: the Hamamatsu Birth Cohort (HBC) Study. Journal of affective disorders 2011; 133(3): 607-10.

155. Banti S, Mauri M, Oppo A, et al. From the third month of pregnancy to 1 year postpartum. Prevalence, incidence, recurrence, and new onset of depression. Results from the perinatal depression-research & screening unit study. Comprehensive psychiatry 2011; 52(4): 343-51.

156. Lee SH, Liu LC, Kuo PC, Lee MS. Postpartum depression and correlated factors in women who received in vitro fertilization treatment. Journal of midwifery & women's health 2011; 56(4): 347-52.

157. Ahmed HM, Alalaf SK, Al-Tawil NG. Screening for postpartum depression using Kurdish version of Edinburgh postnatal depression scale. Archives of gynecology and obstetrics 2012; 285(5): 1249-55.

158. Giardinelli L, Innocenti A, Benni L, et al. Depression and anxiety in perinatal period: prevalence and risk factors in an Italian sample. Archives of women's mental health 2012; 15(1): 21-30.

159. Chien LY, Tai CJ, Yeh MC. Domestic decision-making power, social support, and postpartum depression symptoms among immigrant and native women in Taiwan. Nursing research 2012; 61(2): 103-10.

160. Haga SM, Ulleberg P, Slinning K, Kraft P, Steen TB, Staff A. A longitudinal study of postpartum depressive symptoms: multilevel growth curve analyses of emotion regulation strategies, breastfeeding self-efficacy, and social support. Archives of women's mental health 2012; 15(3): 175-84.

161. Nordeng H, Hansen C, Garthus-Niegel S, Eberhard-Gran M. Fear of childbirth, mental health, and medication use during pregnancy. Archives of women's mental health 2012; 15(3): 203-9.

162. Husain N, Cruickshank K, Husain M, Khan S, Tomenson B, Rahman A. Social stress and depression during pregnancy and in the postnatal period in British Pakistani mothers: a cohort study. Journal of affective disorders 2012; 140(3): 268-76.

163. Demissie Z, Siega-Riz AM, Evenson KR, Herring AH, Dole N, Gaynes BN. Physical activity during pregnancy and postpartum depressive symptoms. Midwifery 2013; 29(2): 139-47.

164. Chen HH, Hwang FM, Tai CJ, Chien LY. The interrelationships among acculturation, social support, and postpartum depression symptoms among marriage-based immigrant women in Taiwan: a cohort study. Journal of immigrant and minority health 2013; 15(1): 17-23.

165. Dolbier CL, Rush TE, Sahadeo LS, Shaffer ML, Thorp J, Community Child Health Network I. Relationships of race and socioeconomic status to postpartum depressive symptoms in rural African American and non-Hispanic white women. Maternal and child health journal 2013; 17(7): 1277-87.

166. Mariam KA, Srinivasan K. Antenatal psychological distress and postnatal depression: A prospective study from an urban clinic. Asian journal of psychiatry 2009; 2(2): 71-3.

167. Sylven SM, Ekselius L, Sundstrom-Poromaa I, Skalkidou A. Premenstrual syndrome and dysphoric disorder as risk factors for postpartum depression. Acta obstetricia et gynecologica Scandinavica 2013; 92(2): 178-84.

168. Lucero NB, Beckstrand RL, Callister LC, Sanchez Birkhead AC. Prevalence of postpartum depression among Hispanic immigrant women. Journal of the American Academy of Nurse Practitioners 2012; 24(12): 726-34.

169. Serhan N, Ege E, Ayranci U, Kosgeroglu N. Prevalence of postpartum depression in mothers and fathers and its correlates. Journal of clinical nursing 2013; 22(1-2): 279-84.

170. Petrozzi A, Gagliardi L. Anxious and depressive components of Edinburgh Postnatal Depression Scale in maternal postpartum psychological problems. Journal of perinatal medicine 2013; 41(4): 343-8.

171. Faisal-Cury A, Menezes PR. Antenatal depression strongly predicts postnatal depression in primary health care. Revista brasileira de psiquiatria 2012; 34(4): 446-50.

172. Wisner KL, Sit DK, McShea MC, et al. Onset timing, thoughts of self-harm, and diagnoses in postpartum women with screen-positive depression findings. JAMA psychiatry 2013; 70(5): 490-8.

173. Abbasi S, Chuang CH, Dagher R, Zhu J, Kjerulff K. Unintended pregnancy and postpartum depression among first-time mothers. Journal of women's health 2013; 22(5): 412-6.

174. Mercier RJ, Garrett J, Thorp J, Siega-Riz AM. Pregnancy intention and postpartum depression: secondary data analysis from a prospective cohort. BJOG : an international journal of obstetrics and gynaecology 2013; 120(9): 1116-22.

175. Burgut FT, Bener A, Ghuloum S, Sheikh J. A study of postpartum depression and maternal risk factors in Qatar. Journal of psychosomatic obstetrics and gynaecology 2013; 34(2): 90-7.

176. Markhus MW, Skotheim S, Graff IE, et al. Low omega-3 index in pregnancy is a possible biological risk factor for postpartum depression. PloS one 2013; 8(7): e67617.

177. Koutra K, Vassilaki M, Georgiou V, et al. Antenatal maternal mental health as determinant of postpartum depression in a population based mother-child cohort (Rhea Study) in Crete, Greece. Social psychiatry and psychiatric epidemiology 2014; 49(5): 711-21.

178. Sidebottom AC, Hellerstedt WL, Harrison PA, Hennrikus D. An examination of prenatal and postpartum depressive symptoms among women served by urban community health centers. Archives of women's mental health 2014; 17(1): 27-40.

179. Sadat Z, Kafaei Atrian M, Masoudi Alavi N, Abbaszadeh F, Karimian Z, Taherian A. Effect of mode of delivery on postpartum depression in Iranian women. The journal of obstetrics and gynaecology research 2014; 40(1): 172-7.

180. Dudek D, Jaeschke R, Siwek M, Maczka G, Topor-Madry R, Rybakowski J. Postpartum depression: identifying associations with bipolarity and personality traits. Preliminary results from a cross-sectional study in Poland. Psychiatry research 2014; 215(1): 69-74.

181. Goshtasebi A, Alizadeh M, Gandevani SB. Association between maternal anaemia and postpartum depression in an urban sample of pregnant women in Iran. Journal of health, population, and nutrition 2013; 31(3): 398-402.

182. Gupta S, Kishore J, Mala YM, Ramji S, Aggarwal R. Postpartum depression in north Indian women: prevalence and risk factors. Journal of obstetrics and gynaecology of India 2013; 63(4): 223-9.

183. Alharbi AA, Abdulghani HM. Risk factors associated with postpartum depression in the Saudi population. Neuropsychiatric disease and treatment 2014; 10: 311-6.

184. Toreki A, Ando B, Dudas RB, et al. Validation of the Edinburgh Postnatal Depression Scale as a screening tool for postpartum depression in a clinical sample in Hungary. Midwifery 2014; 30(8): 911-8.

185. Alfayumi-Zeadna S, Kaufman-Shriqui V, Zeadna A, Lauden A, Shoham-Vardi I. The association between sociodemographic characteristics and postpartum depression symptoms among Arab-Bedouin women in Southern Israel. Depression and anxiety 2015; 32(2): 120-8.

186. Abdollahi F, Rohani S, Sazlina GS, et al. Bio-psycho-socio-demographic and Obstetric Predictors of Postpartum Depression in Pregnancy: A prospective Cohort Study. Iranian journal of psychiatry and behavioral sciences 2014; 8(2): 11-21.

187. Tsao Y, Creedy DK, Gamble J. Prevalence and psychological correlates of postnatal depression in rural Taiwanese women. Health care for women international 2015; 36(4): 457-74.

188. El-Hachem C, Rohayem J, Bou Khalil R, et al. Early identification of women at risk of postpartum depression using the Edinburgh Postnatal Depression Scale (EPDS) in a sample of Lebanese women. BMC psychiatry 2014; 14: 242.

189. Nishigori H, Sugawara J, Obara T, et al. Surveys of postpartum depression in Miyagi, Japan, after the Great East Japan Earthquake. Archives of women's mental health 2014; 17(6): 579-81.

190. Iliadis SI, Koulouris P, Gingnell M, et al. Personality and risk for postpartum depressive symptoms. Archives of women's mental health 2015; 18(3): 539-46.

191. Abdollahi F, Zarghami M, Sazlina SG, Lye MS. Stability of depressive symptoms over 3 months post-partum. Early intervention in psychiatry 2017; 11(1): 57-62.

192. Park JH, Karmaus W, Zhang H. Prevalence of and Risk Factors for Depressive Symptoms in Korean Women throughout Pregnancy and in Postpartum Period. Asian nursing research 2015; 9(3): 219-25.

193. Brown A, Rance J, Bennett P. Understanding the relationship between breastfeeding and postnatal depression: the role of pain and physical difficulties. Journal of advanced nursing 2016; 72(2): 273-82.

194. Robakis TK, Williams KE, Crowe S, Lin KW, Gannon J, Rasgon NL. Maternal attachment insecurity is a potent predictor of depressive symptoms in the early postnatal period. Journal of affective disorders 2016; 190: 623-31.

195. Abdollahi F, Etemadinezhad S, Lye MS. Postpartum mental health in relation to sociocultural practices. Taiwanese journal of obstetrics & gynecology 2016; 55(1): 76-80.

196. Corrigan CP, Kwasky AN, Groh CJ. Social Support, Postpartum Depression, and Professional Assistance: A Survey of Mothers in the Midwestern United States. The Journal of perinatal education 2015; 24(1): 48-60.

197. Khalifa DS, Glavin K, Bjertness E, Lien L. Determinants of postnatal depression in Sudanese women at 3 months postpartum: a cross-sectional study. BMJ open 2016; 6(3): e009443.

198. Dennis CL, Merry L, Stewart D, Gagnon AJ. Prevalence, continuation, and identification of postpartum depressive symptomatology among refugee, asylum-seeking, non-refugee immigrant, and Canadian-born women: results from a prospective cohort study. Archives of women's mental health 2016; 19(6): 959-67.

199.Kim Y, Dee V. Self-Care for Health in Rural Hispanic Women at Risk for Postpartum Depression. Maternal and child health journal 2017; 21(1): 77-84.

200. Lovlie AL, Madar AA. Postpartum Depression Among Somali Women in Norway. Journal of immigrant and minority health 2017; 19(3): 638-44.

201. Qandil S, Jabr S, Wagler S, Collin SM. Postpartum depression in the Occupied Palestinian Territory: a longitudinal study in Bethlehem. BMC pregnancy and childbirth 2016; 16(1): 375.

202. Nam JY, Choi Y, Kim J, Cho KH, Park EC. The synergistic effect of breastfeeding discontinuation and cesarean section delivery on postpartum depression: A nationwide population-based cohort study in Korea. Journal of affective disorders 2017; 218: 53-8.

203. Islam MJ, Broidy L, Baird K, Mazerolle P. Intimate partner violence around the time of pregnancy and postpartum depression: The experience of women of Bangladesh. PloS one 2017; 12(5): e0176211.

204. Souza KJ, Rattner D, Gubert MB. Institutional violence and quality of service in obstetrics are associated with postpartum depression. Revista de saude publica 2017; 51: 69.

205. Dennis CL, Brown HK, Wanigaratne S, et al. Prevalence, Incidence, and Persistence of Postpartum Depression, Anxiety, and Comorbidity among Chinese Immigrant and Nonimmigrant Women: A Longitudinal Cohort Study. Canadian journal of psychiatry Revue canadienne de psychiatrie 2018; 63(1): 44-53.

206. Lara-Cinisomo S, McKenney K, Di Florio A, Meltzer-Brody S. Associations Between Postpartum Depression, Breastfeeding, and Oxytocin Levels in Latina Mothers. Breastfeeding medicine : the official journal of the Academy of Breastfeeding Medicine 2017; 12(7): 436-42.

207. Muruganandam P, Shanmugam D, Ramachandran N. Does the Mode of Conception Influence Early Postpartum Depression? A Prospective Comparative Study from South India. Indian journal of psychological medicine 2020; 42(6): 525-9.

208. Bodhare TN, Sethi P, Bele SD, Gayatri D, Vivekanand A. Postnatal quality of life, depressive symptoms, and social support among women in southern India. Women & health 2015; 55(3): 353-65.

209. Iwata H, Mori E, Tsuchiya M, et al. Predictors of depressive symptoms in older Japanese primiparas at 1 month post-partum: A risk-stratified analysis. Japan journal of nursing science : JJNS 2016; 13(1): 147-55.

210. Otake Y, Nakajima S, Uno A, et al. Association between maternal antenatal depression and infant development: a hospital-based prospective cohort study. Environmental health and preventive medicine 2014; 19(1): 30-45.

211. Mori T, Tsuchiya KJ, Matsumoto K, et al. Psychosocial risk factors for postpartum depression and their relation to timing of onset: the Hamamatsu Birth Cohort (HBC) Study. Journal of affective disorders 2011; 135(1-3): 341-6.

212. Zaidi F, Nigam A, Anjum R, Agarwalla R. Postpartum Depression in Women: A Risk Factor Analysis. Journal of clinical and diagnostic research : JCDR 2017; 11(8): QC13-QC6.

213. Dennis CL, Brown HK, Wanigaratne S, et al. Determinants of comorbid depression and anxiety postnatally: A longitudinal cohort study of Chinese-Canadian women. Journal of affective disorders 2018; 227: 24-30.

214. Yan J, Liu Y, Cao L, Zheng Y, Li W, Huang G. Association between Duration of Folic Acid Supplementation during Pregnancy and Risk of Postpartum Depression. Nutrients 2017; 9(11).

215. Kim Y, Dee V. Sociodemographic and Obstetric Factors Related to Symptoms of Postpartum Depression in Hispanic Women in Rural California. Journal of obstetric, gynecologic, and neonatal nursing : JOGNN 2018; 47(1): 23-31.

216. Zhao Y, Munro-Kramer ML, Shi S, Wang J, Zhu X. A longitudinal study of perinatal depression among Chinese high-risk pregnant women. Women and birth : journal of the Australian College of Midwives 2018; 31(6): e395-e402.

217. Badr LK, Ayvazian N, Lameh S, Charafeddine L. Is the Effect of Postpartum Depression on Mother-Infant Bonding Universal? Infant behavior & development 2018; 51: 15-23.

218. Adamu AF, Adinew YM. Domestic Violence as a Risk Factor for Postpartum Depression Among Ethiopian Women: Facility Based Study. Clinical practice and epidemiology in mental health : CP & EMH 2018; 14: 109-19.

219. Khalifa DS, Glavin K, Bjertness E, Lien L. Course of depression symptoms between 3 and 8 months after delivery using two screening tools (EPDS and HSCL-10) on a sample of Sudanese women in Khartoum state. BMC pregnancy and childbirth 2018; 18(1): 324.

220. Ruohomaki A, Toffol E, Upadhyaya S, et al. The association between gestational diabetes mellitus and postpartum depressive symptomatology: A prospective cohort study. Journal of affective disorders 2018; 241: 263-8.

221. Chandrasekaran N, De Souza LR, Urquia ML, et al. Is anemia an independent risk factor for postpartum depression in women who have a cesarean section? - A prospective observational study. BMC pregnancy and childbirth 2018; 18(1): 400.

222. Miyake Y, Tanaka K, Sasaki S, Hirota Y. Employment, income, and education and risk of postpartum depression: the Osaka Maternal and Child Health Study. Journal of affective disorders 2011; 130(1-2): 133-7.

223. Min W, Nie W, Song S, et al. Associations between Maternal and Infant Illness and the Risk of Postpartum Depression in Rural China: A Cross-Sectional Observational Study. International journal of environmental research and public health 2020; 17(24).

224. Yelland J, Sutherland G, Brown SJ. Postpartum anxiety, depression and social health: findings from a population-based survey of Australian women. BMC public health 2010; 10: 771.

225. Nayak D, Karuppusamy D, Maurya DK, Kar SS, Bharadwaj B, Keepanasseril A. Postpartum depression and its risk factors in women with a potentially life-threatening complication. International journal of gynaecology and obstetrics: the official organ of the International Federation of Gynaecology and Obstetrics 2020.

226. Nagpal J, Dhar RS, Sinha S, Bhargava V, Sachdeva A, Bhartia A. An exploratory study to evaluate the utility of an adapted Mother Generated Index (MGI) in assessment of postpartum quality of life in India. Health and quality of life outcomes 2008; 6: 107.

227. Iyengar K, Yadav R, Sen S. Consequences of maternal complications in women's lives in the first postpartum year: a prospective cohort study. Journal of health, population, and nutrition 2012; 30(2): 226-40.

228. Prost A, Lakshminarayana R, Nair N, et al. Predictors of maternal psychological distress in rural India: a cross-sectional community-based study. Journal of affective disorders 2012; 138(3): 277-86.

229. Alhasanat-Khalil D, Giurgescu C, Benkert R, Fry-McComish J, Misra DP, Yarandi H. Acculturation and Postpartum Depression Among Immigrant Women of Arabic Descent. Journal of immigrant and minority health 2019; 21(6): 1208-16.

230. Ertmann RK, Nicolaisdottir DR, Kragstrup J, Siersma V, Lutterodt MC, Bech P. Physical discomfort in early pregnancy and postpartum depressive symptoms. Nordic journal of psychiatry 2019; 73(3): 200-6.

231. Hassert S, Sharon SR, Payakkakom A, Kodysova E. Postpartum Depressive Symptoms: Risks for Czech and Thai Mothers. The Journal of perinatal education 2018; 27(1): 38-49.

232. Hoge A, Tabar V, Donneau AF, et al. Imbalance between Omega-6 and Omega-3 Polyunsaturated Fatty Acids in Early Pregnancy Is Predictive of Postpartum Depression in a Belgian Cohort. Nutrients 2019; 11(4).

233. Premji S, McDonald SW, Metcalfe A, et al. Examining postpartum depression screening effectiveness in well child clinics in Alberta, Canada: A study using the All Our Families cohort and administrative data. Preventive medicine reports 2019; 14: 100888.

234. Labrague LJ, McEnroe-Petitte D, Tsaras K, et al. Predictors of postpartum depression and the utilization of postpartum depression services in rural areas in the Philippines. Perspectives in psychiatric care 2020; 56(2): 308-15.

235. Gan Y, Xiong R, Song J, et al. The effect of perceived social support during early pregnancy on depressive symptoms at 6 weeks postpartum: a prospective study. BMC psychiatry 2019; 19(1): 232.

236. Bhusal BR, Bhandari N. Identifying the factors associated with depressive symptoms among postpartum mothers in Kathmandu, Nepal. International journal of nursing sciences 2018; 5(3): 268-74.

237. Hegde S. Postpartum Depression: Prevalence and Associated Factors among Women in India. Journal of Women's Health, Issue & Care 2012.

238. Bo HX, Yang Y, Chen J, et al. Prevalence of depressive symptoms among Chinese pregnant and postpartum women during the COVID-19 pandemic. Psychosomatic medicine 2020; Publish Ahead of Print.

239. Abebe A, Tesfaw G, Mulat H, Hibdye G, Yohannes K. Postpartum depression and associated factors among mothers in Bahir Dar Town, Northwest Ethiopia. Annals of general psychiatry 2019; 18: 19.

240. Hamazaki K, Matsumura K, Tsuchida A, et al. Dietary intake of fish and n-3 polyunsaturated fatty acids and risk of postpartum depression: a nationwide longitudinal study - the Japan Environment and Children's Study (JECS). Psychological medicine 2020; 50(14): 2416-24.

241. Maeda Y, Ogawa K, Morisaki N, Tachibana Y, Horikawa R, Sago H. Association between perinatal anemia and postpartum depression: A prospective cohort study of Japanese women. International journal of gynaecology and obstetrics: the official organ of the International Federation of Gynaecology and Obstetrics 2020; 148(1): 48-52.

242. Amirchaghmaghi E, Malekzadeh F, Chehrazi M, Ezabadi Z, Sabeti SH. A Comparison of Postpartum Depression in Mothers Conceived by Assisted Reproductive Technology and Those Naturally Conceived. International journal of fertility & sterility 2020; 13(4): 277-81.

243. Valdes V, Berens AE, Nelson CA, 3rd. Socioeconomic and psychological correlates of postpartum depression at 6 months in Dhaka, Bangladesh. International journal of psychology : Journal international de psychologie 2020.

244. Simhi M, Sarid O, Cwikel J. Preferences for mental health treatment for post-partum depression among new mothers. Israel journal of health policy research 2019; 8(1): 84.

245. Gausia K. Validation of the Bangla version of the Edinburgh Postnatal Depression Scale for a Bangladeshi sample. Journal of reproductive and infant psychology 2007; VOL. 25, NO. 4, NOVEMBER 2007: 308-15.

246. Chan CL, Tan CW, Chan JJI, et al. Factors Associated with the Development of Postnatal Depression After Cesarean Delivery: A Prospective Study. Neuropsychiatric disease and treatment 2020; 16: 715-27.

247. Wubetu AD, Engidaw NA, Gizachew KD. Prevalence of postpartum depression and associated factors among postnatal care attendees in Debre Berhan, Ethiopia, 2018. BMC pregnancy and childbirth 2020; 20(1): 189.

248. Hashima-E-Nasreen. Incidence and Risk Factor of Postpartum Depressive Symptoms in Women: A Population Based Prospective Cohort Study in a Rural District in Bangladesh. Journal of Depression and Anxiety 2015.

249. Spinola O, Liotti M, Speranza AM, Tambelli R. Effects of COVID-19 Epidemic Lockdown on Postpartum Depressive Symptoms in a Sample of Italian Mothers. Frontiers in psychiatry 2020; 11: 589916.

250. Dawadi P, Bhatta AS, Shakya J. Factors Associated with Postpartum Depressive Symptoms in Community of Central Nepal. Psychiatry journal 2020; 2020: 8305304.

251. Pradhananga P, Mali P, Poudel L, Gurung M. Prevalence of Postpartum Depression in a Tertiary Health Care. JNMA; journal of the Nepal Medical Association 2020; 58(223): 137-40.

252. Abdollahi F, Agajani-Delavar M, Zarghami M, Lye MS. Postpartum Mental Health in First-Time Mothers: A Cohort Study. Iranian journal of psychiatry and behavioral sciences 2016; 10(1): e426.

253. Tan CW, Sultana R, Kee MZL, Meaney MJ, Sng BL. Investigating the association between labour epidural analgesia and postpartum depression: A prospective cohort study. European journal of anaesthesiology 2020; 37(9): 796-802.

254. Yamada A, Isumi A, Fujiwara T. Association between Lack of Social Support from Partner or Others and Postpartum Depression among Japanese Mothers: A Population-Based Cross-Sectional Study. International journal of environmental research and public health 2020; 17(12).

255. Alikamali M, Khodabandeh S, Motesaddi M, Bagheri Z, Esmaeili MA. The Association Between Demographic Characteristics and Attempting of Pregnancy with Postpartum Depression and Anxiety Among Women Referring to Community Health Centres: A Cross Sectional Study. The Malaysian journal of medical sciences : MJMS 2020; 27(3): 93-104.

256. Boran P, Waqas A, Askan OO, Topcu I, Dogan T, Rahman A. Screening of postpartum depression among new mothers in Istanbul: a psychometric evaluation of the Turkish Edinburgh Postnatal Depression Scale. BMC research notes 2020; 13(1): 355.

257. Bakare MO, Okoye JO, Obindo JT. Introducing depression and developmental screenings into the national programme on immunization (NPI) in southeast Nigeria: an experimental cross-sectional assessment. General hospital psychiatry 2014; 36(1): 105-12.

258. Tatano Beck C, Gable RK, Sakala C, Declercq ER. Postpartum Depressive symptomatology: results from a two-stage US national survey. Journal of midwifery & women's health 2011; 56(5): 427-35.

259. McCoy SJ, Beal JM, Shipman SB, Payton ME, Watson GH. Risk factors for postpartum depression: a retrospective investigation at 4-weeks postnatal and a review of the literature. The Journal of the American Osteopathic Association 2006; 106(4): 193-8.

260. *Appolonio KK, Fingerhut R. Postpartum depression in a military sample. Military medicine 2008; 173(11): 1085-91.*

261. *Boury JM, Larkin KT, Krummel DA. Factors related to postpartum depressive symptoms in low-income women. Women & health 2004; 39(3): 19-34.*

262. *Mayberry LJ, Horowitz JA, Declercq E. Depression symptom prevalence and demographic risk factors among U.S. women during the first 2 years postpartum. Journal of obstetric, gynecologic, and neonatal nursing : JOGNN 2007; 36(6): 542-9.*

263. *Bugdayci R, Sasmaz CT, Tezcan H, Kurt AO, Oner S. A cross-sectional prevalence study of depression at various times after delivery in Mersin province in Turkey. Journal of women's health 2004; 13(1): 63-8.*

264. *Luan B, Goodarzi MO, Phillips NG, et al. Leptin-mediated increases in catecholamine signaling reduce adipose tissue inflammation via activation of macrophage HDAC4. Cell metabolism 2014; 19(6): 1058-65.*

265. *Miles MS, Holditch-Davis D, Schwartz TA, Scher M. Depressive symptoms in mothers of prematurely born infants. Journal of developmental and behavioral pediatrics : JDBP 2007; 28(1): 36-44.*

266. *Cheng CY, Pickler RH. Effects of stress and social support on postpartum health of Chinese mothers in the United States. Research in nursing & health 2009; 32(6): 582-91.*

267. *Verkerk GJ, Denollet J, Van Heck GL, Van Son MJ, Pop VJ. Personality factors as determinants of depression in postpartum women: a prospective 1-year follow-up study. Psychosomatic medicine 2005; 67(4): 632-7.*

268. *Chien LY, Tai CJ, Hwang FM, Huang CM. Postpartum physical symptoms and depressive symptomatology at 1 month and 1 year after delivery: a longitudinal questionnaire survey. International journal of nursing studies 2009; 46(9): 1201-8.*

269. *Lara MA, Navarrete L, Nieto L, Martin JP, Navarro JL, Lara-Tapia H. Prevalence and incidence of perinatal depression and depressive symptoms among Mexican women. Journal of affective disorders 2015; 175: 18-24.*

270. *Leung SS, Martinson IM, Arthur D. Postpartum depression and related psychosocial variables in Hong Kong Chinese women: findings from a prospective study. Research in nursing & health 2005; 28(1): 27-38.*

271. *McMahon C, Barnett B, Kowalenko N, Tennant C. Psychological factors associated with persistent postnatal depression: past and current relationships, defence styles and the mediating role of insecure attachment style. Journal of affective disorders 2005; 84(1): 15-24.*

272. *Motzfeldt I, Andreasen S, Pedersen AL, Pedersen ML. Prevalence of postpartum depression in Nuuk, Greenland--a cross-sectional study using Edinburgh Postnatal Depression Scale. International journal of circumpolar health 2013; 72.*

273. Murray L, Dunne MP, Van Vo T, Anh PN, Khawaja NG, Cao TN. Postnatal depressive symptoms amongst women in Central Vietnam: a cross-sectional study investigating prevalence and associations with social, cultural and infant factors. BMC pregnancy and childbirth 2015; 15: 234.

274. Lee DT, Yip AS, Leung TY, Chung TK. Ethnoepidemiology of postnatal depression. Prospective multivariate study of sociocultural risk factors in a Chinese population in Hong Kong. The British journal of psychiatry : the journal of mental science 2004; 184: 34-40.

275. Necho M, Belete A, Zenebe Y. The association of intimate partner violence with postpartum depression in women during their first month period of giving delivery in health centers at Dessie town, 2019. Annals of general psychiatry 2020; 19: 59.

276. Salem MN. Factors affecting the occurrence of postpartum depression among puerperal women in Sohag city, Egypt. Proceedings in Obstetrics and Gynecology 2017; 2017;7(1):4.

277. Akbari V, Rahmatinejad P, Shater MM, Vahedian M, Khalajinia Z. Investigation of the relationship of perceived social support and spiritual well-being with postpartum depression. Journal of education and health promotion 2020; 9: 174.

278. Zeng Y, Tan CW, Sultana R, et al. Association of Pain Catastrophizing with Postnatal Depressive States in Nulliparous Parturients: A Prospective Study. Neuropsychiatric disease and treatment 2020; 16: 1853-62.

279.Mokwena K, Masike I. The Need for Universal Screening for Postnatal Depression in South Africa: Confirmation from a Sub-District in Pretoria, South Africa. International journal of environmental research and public health 2020; 17(19).

280. Karl M, Schaber R, Kress V, et al. Precarious working conditions and psychosocial work stress act as a risk factor for symptoms of postpartum depression during maternity leave: results from a longitudinal cohort study. BMC public health 2020; 20(1): 1505.

281. Chalise M, Karmacharya I, Kaphle M, Wagle A, Chand N, Adhikari L. Factors Associated with Postnatal Depression among Mothers Attending at Bharatpur Hospital, Chitwan. Depression research and treatment 2020; 2020: 9127672.

282. Cena L, Mirabella F, Palumbo G, Gigantesco A, Trainini A, Stefana A. Prevalence of maternal antenatal and postnatal depression and their association with sociodemographic and socioeconomic factors: A multicentre study in Italy. Journal of affective disorders 2021; 279: 217-21.

283. Da Costa D, Dritsa M, Rippen N, Lowensteyn I, Khalife S. Health-related quality of life in postpartum depressed women. Archives of women's mental health 2006; 9(2): 95-102.

284. Dennis CL, Ross L. Women's perceptions of partner support and conflict in the development of postpartum depressive symptoms. Journal of advanced nursing 2006; 56(6): 588-99.

285. Dennis CL, Letourneau N. Global and relationship-specific perceptions of support and the development of postpartum depressive symptomatology. Social psychiatry and psychiatric epidemiology 2007; 42(5): 389-95.

286. Kim TH, Connolly JA, Tamim H. The effect of social support around pregnancy on postpartum depression among Canadian teen mothers and adult mothers in the maternity experiences survey. BMC pregnancy and childbirth 2014; 14: 162.

287. Dennis CL, Heaman M, Vigod S. Epidemiology of postpartum depressive symptoms among Canadian women: regional and national results from a cross-sectional survey. Canadian journal of psychiatry Revue canadienne de psychiatrie 2012; 57(9): 537-46.

288. Logsdon MC, Birkimer JC, Simpson T, Looney S. Postpartum depression and social support in adolescents. Journal of obstetric, gynecologic, and neonatal nursing : JOGNN 2005; 34(1): 46-54.

289. Chatzi L, Melaki V, Sarri K, et al. Dietary patterns during pregnancy and the risk of postpartum depression: the mother-child 'Rhea' cohort in Crete, Greece. Public health nutrition 2011; 14(9): 1663-70.

290. Sword W, Landy CK, Thabane L, et al. Is mode of delivery associated with postpartum depression at 6 weeks: a prospective cohort study. BJOG : an international journal of obstetrics and gynaecology 2011; 118(8): 966-77.

291. Edhborg M, Nasreen HE, Kabir ZN. Impact of postpartum depressive and anxiety symptoms on mothers' emotional tie to their infants 2-3 months postpartum: a population-based study from rural Bangladesh. Archives of women's mental health 2011; 14(4): 307-16.

292. Al Dallal FH, Grant IN. Postnatal depression among Bahraini women: prevalence of symptoms and psychosocial risk factors. Eastern Mediterranean health journal = La revue de sante de la Mediterranee orientale = al-Majallah al-sihhiyah li-sharq al-mutawassit 2012; 18(5): 439-45.

293. Eastwood JG, Phung H, Barnett B. Postnatal depression and socio-demographic risk: factors associated with Edinburgh Depression Scale scores in a metropolitan area of New South Wales, Australia. The Australian and New Zealand journal of psychiatry 2011; 45(12): 1040-6.

294. Buist AE, Austin MP, Hayes BA, et al. Postnatal mental health of women giving birth in Australia 2002-2004: findings from the beyondblue National Postnatal Depression Program. The Australian and New Zealand journal of psychiatry 2008; 42(1): 66-73.

295. Abdelwahid HAE. Postpartum depression among women in a rural community, Ismailia, Egypt. 2012.

296. Melo EF, Jr., Cecatti JG, Pacagnella RC, Leite DF, Vulcani DE, Makuch MY. The prevalence of perinatal depression and its associated factors in two different settings in Brazil. Journal of affective disorders 2012; 136(3): 1204-8.

297. Bener A, Gerber LM, Sheikh J. Prevalence of psychiatric disorders and associated risk factors in women during their postpartum period: a major public health problem and global comparison. International journal of women's health 2012; 4: 191-200.

298. Alasoom LI, Koura MR. Predictors of postpartum depression in the eastern province capital of saudi arabia. Journal of family medicine and primary care 2014; 3(2): 146-50.

299. Chalise A, Bhandari TR. Postpartum Depression and its Associated Factors: A Community-based Study in Nepal. Journal of Nepal Health Research Council 2019; 17(2): 200-5.

300. Khadka R, Hong SA, Chang YS. Prevalence and determinants of poor sleep quality and depression among postpartum women: a community-based study in Ramechhap district, Nepal. International health 2020; 12(2): 125-31.

301. Bilszta JL, Gu YZ, Meyer D, Buist AE. A geographic comparison of the prevalence and risk factors for postnatal depression in an Australian population. Australian and New Zealand journal of public health 2008; 32(5): 424-30.

302. Boyce P, Hickey A. Psychosocial risk factors to major depression after childbirth. Social psychiatry and psychiatric epidemiology 2005; 40(8): 605-12.

303. Eckerdal P, Kollia N, Karlsson L, et al. Epidural Analgesia During Childbirth and Postpartum Depressive Symptoms: A Population-Based Longitudinal Cohort Study. Anesthesia and analgesia 2020; 130(3): 615-24.

304. Silva R, Jansen K, Souza L, et al. Sociodemographic risk factors of perinatal depression: a cohort study in the public health care system. Revista brasileira de psiquiatria 2012; 34(2): 143-8.

305. Malta LA, McDonald SW, Hegadoren KM, Weller CA, Tough SC. Influence of interpersonal violence on maternal anxiety, depression, stress and parenting morale in the early postpartum: a community based pregnancy cohort study. BMC pregnancy and childbirth 2012; 12: 153.

306. Brooks J, Nathan E, Speelman C, Swalm D, Jacques A, Doherty D. Tailoring screening protocols for perinatal depression: prevalence of high risk across obstetric services in Western Australia. Archives of women's mental health 2009; 12(2): 105-12.

307. Leigh B, Milgrom J. Risk factors for antenatal depression, postnatal depression and parenting stress. BMC psychiatry 2008; 8: 24.

308. Wynter K, Rowe H, Fisher J. Common mental disorders in women and men in the first six months after the birth of their first infant: a community study in Victoria, Australia. Journal of affective disorders 2013; 151(3): 980-5.

309. Theme Filha MM, Ayers S, da Gama SG, Leal Mdo C. Factors associated with postpartum depressive symptomatology in Brazil: The Birth in Brazil National Research Study, 2011/2012. Journal of affective disorders 2016; 194: 159-67.

310. Matijasevich A, Golding J, Smith GD, Santos IS, Barros AJ, Victora CG. Differentials and income-related inequalities in maternal depression during the first two years after childbirth: birth cohort studies from Brazil and the UK. Clinical practice and epidemiology in mental health : CP & EMH 2009; 5: 12.

311. MORAIS MdLSe. Postpartum depression and child development in first year of life. 2013.

312. Pinheiro RT, Coelho FM, Silva RA, et al. Association of a serotonin transporter gene polymorphism (5-HTTLPR) and stressful life events with postpartum depressive symptoms: a population-based study. Journal of psychosomatic obstetrics and gynaecology 2013; 34(1): 29-33.

313. Dennis CL, Vigod S. The relationship between postpartum depression, domestic violence, childhood violence, and substance use: epidemiologic study of a large community sample. Violence against women 2013; 19(4): 503-17.

314. Dennis CL, Hodnett E, Kenton L, et al. Effect of peer support on prevention of postnatal depression among high risk women: multisite randomised controlled trial. Bmj 2009; 338: a3064.

315. McDonald S, Wall J, Forbes K, et al. Development of a prenatal psychosocial screening tool for post-partum depression and anxiety. Paediatric and perinatal epidemiology 2012; 26(4): 316-27.

316. Mao Q, Zhu LX, Su XY. A comparison of postnatal depression and related factors between Chinese new mothers and fathers. Journal of clinical nursing 2011; 20(5-6): 645-52.

317. Paulson JF, Dauber S, Leiferman JA. Individual and combined effects of postpartum depression in mothers and fathers on parenting behavior. Pediatrics 2006; 118(2): 659-68.

318. Drozd F, Haga SM, Valla L, Slinning K. Latent trajectory classes of postpartum depressive symptoms: A regional population-based longitudinal study. Journal of affective disorders 2018; 241: 29-36.

319. Gao LL, Chan SW, You L, Li X. Experiences of postpartum depression among first-time mothers in mainland China. Journal of advanced nursing 2010; 66(2): 303-12.

320. Leung WC, Kung F, Lam J, Leung TW, Ho PC. Domestic violence and postnatal depression in a Chinese community. International journal of gynaecology and obstetrics: the official organ of the International Federation of Gynaecology and Obstetrics 2002; 79(2): 159-66.

321.Chen L, Wang X, Ding Q, Shan N, Qi H. Development of Postpartum Depression in Pregnant Women with Preeclampsia: A Retrospective Study. BioMed research international 2019; 2019: 9601476.

322. Tashakori A, Behbahani AZ, Irani RD. Comparison Of Prevalence Of Postpartum Depression Symptoms Between Breastfeeding Mothers And Non-breastfeeding Mothers. Iranian journal of psychiatry 2012; 7(2): 61-5.

323. January J, Chivanhu H, Chiwara J, et al. Prevalence and the correlates of postnatal depression in an urban high density suburb of Harare. The Central African journal of medicine 2015; 61(1-4): 1-4.

324.de Tychey C, Spitz E, Briancon S, et al. Pre- and postnatal depression and coping: a comparative approach. Journal of affective disorders 2005; 85(3): 323-6.

325. Zejnullahu VA, Ukella-Lleshi D, Zejnullahu VA, Miftari E, Govori V. Prevalence of postpartum depression at the clinic for obstetrics and gynecology in Kosovo teaching hospital: Demographic, obstetric and psychosocial risk factors. European journal of obstetrics, gynecology, and reproductive biology 2021; 256: 215-20.

326. Reck C, Struben K, Backenstrass M, et al. Prevalence, onset and comorbidity of postpartum anxiety and depressive disorders. Acta psychiatrica Scandinavica 2008; 118(6): 459-68.

327. Giakoumaki O, Vasilaki K, Lili L, Skouroliakou M, Liosis G. The role of maternal anxiety in the early postpartum period: screening for anxiety and depressive symptomatology in Greece. Journal of psychosomatic obstetrics and gynaecology 2009; 30(1): 21-8.

328. Stone SL, Diop H, Declercq E, Cabral HJ, Fox MP, Wise LA. Stressful events during pregnancy and postpartum depressive symptoms. Journal of women's health 2015; 24(5): 384-93.

329. Lynch CD, Prasad MR. Association between infertility treatment and symptoms of postpartum depression. Fertility and sterility 2014; 102(5): 1416-21.

330. Pooler J, Perry DF, Ghandour RM. Prevalence and risk factors for postpartum depressive symptoms among women enrolled in WIC. Maternal and child health journal 2013; 17(10): 1969-80.

331. Schachman K, Lindsey L. A resilience perspective of postpartum depressive symptomatology in military wives. Journal of obstetric, gynecologic, and neonatal nursing : JOGNN 2013; 42(2): 157-67.

332. Sweeney AC, Fingerhut R. Examining relationships between body dissatisfaction, maladaptive perfectionism, and postpartum depression symptoms. Journal of obstetric, gynecologic, and neonatal nursing : JOGNN 2013; 42(5): 551-61.

333. Dagher RK, Shenassa ED. Prenatal health behaviors and postpartum depression: is there an association? Archives of women's mental health 2012; 15(1): 31-7.

334. Gress-Smith JL, Luecken LJ, Lemery-Chalfant K, Howe R. Postpartum depression prevalence and impact on infant health, weight, and sleep in low-income and ethnic minority women and infants. Maternal and child health journal 2012; 16(4): 887-93.

335. Kornfeld BD, Bair-Merritt MH, Frosch E, Solomon BS. Postpartum depression and intimate partner violence in urban mothers: co-occurrence and child healthcare utilization. The Journal of pediatrics 2012; 161(2): 348-53 e2.

336. Gjerdingen D, Crow S, McGovern P, Miner M, Center B. Changes in depressive symptoms over 0-9 months postpartum. Journal of women's health 2011; 20(3): 381-6.

337. Murphy PK, Mueller M, Hulsey TC, Ebeling MD, Wagner CL. An exploratory study of postpartum depression and vitamin d. Journal of the American Psychiatric Nurses Association 2010; 16(3): 170-7.

338. Le HN, Perry DF, Ortiz G. The Postpartum Depression Screening Scale-Spanish version: examining the psychometric properties and prevalence of risk for postpartum depression. Journal of immigrant and minority health 2010; 12(2): 249-58.

339. Sorenson DS, Tschetter L. Prevalence of negative birth perception, disaffirmation, perinatal trauma symptoms, and depression among postpartum women. Perspectives in psychiatric care 2010; 46(1): 14-25.

340. McGrath JM, Records K, Rice M. Maternal depression and infant temperament characteristics. Infant behavior & development 2008; 31(1): 71-80.

341. Pinheiro KA, Pinheiro RT, Silva RA, et al. Chronicity and severity of maternal postpartum depression and infant sleep disorders: a population-based cohort study in southern Brazil. Infant behavior & development 2011; 34(2): 371-3.

342. Abiodun OA. Postnatal depression in primary care populations in Nigeria. General hospital psychiatry 2006; 28(2): 133-6.

343. Panthangi V, West P, Savoy-Moore RT, Geeta M, Reickert E. Is seasonal variation another risk factor for postpartum depression? Journal of the American Board of Family Medicine : JABFM 2009; 22(5): 492-7.

344. Yawn BP, Pace W, Wollan PC, et al. Concordance of Edinburgh Postnatal Depression Scale (EPDS) and Patient Health Questionnaire (PHQ-9) to assess increased risk of depression among postpartum women. Journal of the American Board of Family Medicine : JABFM 2009; 22(5): 483-91.

345. Shafiei T, Small R, McLachlan H. Immigrant Afghan women's emotional well-being after birth and use of health services in Melbourne, Australia. Midwifery 2015; 31(7): 671-7.

346. Abbasi M, van den Akker O, Bewley C. Persian couples' experiences of depressive symptoms and health-related quality of life in the pre- and perinatal period. Journal of psychosomatic obstetrics and gynaecology 2014; 35(1): 16-21.

347. Kheirabadi GR, Maracy MR. Perinatal depression in a cohort study on Iranian women. Journal of research in medical sciences : the official journal of Isfahan University of Medical Sciences 2010; 15(1): 41-9.

348. Kheirabadi GR, Maracy MR, Barekatain M, et al. Risk factors of postpartum depression in rural areas of Isfahan Province, Iran. Archives of Iranian medicine 2009; 12(5): 461-7.

349. Abadiga M. Magnitude and associated factors of postpartum depression among women in Nekemte town, East Wollega zone, west Ethiopia, 2019: A community-based study. PloS one 2019; 14(11): e0224792.

350. Upadhyay AK, Singh A, Singh A. Association between unintended births and risk of postpartum depression: Evidence from Ethiopia, India, Peru and Vietnam. SSM - population health 2019; 9: 100495.

351. Azale T, Fekadu A, Hanlon C. Postpartum depressive symptoms in the context of high social adversity and reproductive health threats: a population-based study. International journal of mental health systems 2018; 12: 42.

352. Tsai AC, Tomlinson M, Comulada WS, Rotheram-Borus MJ. Intimate Partner Violence and Depression Symptom Severity among South African Women during Pregnancy and Postpartum: Population-Based Prospective Cohort Study. PLoS medicine 2016; 13(1): e1001943.

353. Hung KJ, Tomlinson M, le Roux IM, Dewing S, Chopra M, Tsai AC. Community-based prenatal screening for postpartum depression in a South African township. International journal of gynaecology and obstetrics: the official organ of the International Federation of Gynaecology and Obstetrics 2014; 126(1): 74-7.

354. Baumgartner JN, Parcesepe A, Mekuria YG, et al. Maternal mental health in Amhara region, Ethiopia: a cross-sectional survey. Global health, science and practice 2014; 2(4): 482-6.

355. Mauri M, Oppo A, Montagnani MS, et al. Beyond "postpartum depressions": specific anxiety diagnoses during pregnancy predict different outcomes: results from PND-ReScU. Journal of affective disorders 2010; 127(1-3): 177-84.

356. Nishizono-Maher A, Kishimoto J, Yoshida H, et al. The role of self-report questionnaire in the screening of postnatal depression- a community sample survey in central Tokyo. Social psychiatry and psychiatric epidemiology 2004; 39(3): 185-90.

357. Kim JJ, Gordon TE, La Porte LM, Adams M, Kuendig JM, Silver RK. The utility of maternal depression screening in the third trimester. American journal of obstetrics and gynecology 2008; 199(5): 509 e1-5.

358. Dow A, Dube Q, Pence BW, Van Rie A. Postpartum depression and HIV infection among women in Malawi. Journal of acquired immune deficiency syndromes 2014; 65(3): 359-65.

359. Underwood L, Waldie KE, D'Souza S, Peterson ER, Morton SM. A Longitudinal Study of Pre-pregnancy and Pregnancy Risk Factors Associated with Antenatal and Postnatal Symptoms of Depression: Evidence from Growing Up in New Zealand. Maternal and child health journal 2017; 21(4): 915-31.

360. Adewuya AO, Ola BA, Dada AO, Fasoto OO. Validation of the Edinburgh Postnatal Depression Scale as a screening tool for depression in late pregnancy among Nigerian women. Journal of psychosomatic obstetrics and gynaecology 2006; 27(4): 267-72.

361. Dorheim SK, Bjorvatn B, Eberhard-Gran M. Can insomnia in pregnancy predict postpartum depression? A longitudinal, population-based study. PloS one 2014; 9(4): e94674.

362. Al-Modayfer O. Postpartum depression and related risk factors among Saudi females. International Journal of Culture and Mental Health 2015; 8:3: 316-24.

363. Heh SS, Huang LH, Ho SM, Fu YY, Wang LL. Effectiveness of an exercise support program in reducing the severity of postnatal depression in Taiwanese women. Birth 2008; 35(1): 60-5.

364. Akman I, Kuscu MK, Yurdakul Z, et al. Breastfeeding duration and postpartum psychological adjustment: role of maternal attachment styles. Journal of paediatrics and child health 2008; 44(6): 369-73.

365. *Leahy-Warren P, McCarthy G, Corcoran P. Postnatal depression in first-time mothers: prevalence and relationships between functional and structural social support at 6 and 12 weeks postpartum. Archives of psychiatric nursing 2011; 25(3): 174-84.*

366. *Goecke TW, Voigt F, Faschingbauer F, Spangler G, Beckmann MW, Beetz A. The association of prenatal attachment and perinatal factors with pre- and postpartum depression in first-time mothers. Archives of gynecology and obstetrics 2012; 286(2): 309-16.*

367. *Meijer JL, Beijers C, van Pampus MG, et al. Predictive accuracy of Edinburgh postnatal depression scale assessment during pregnancy for the risk of developing postpartum depressive symptoms: a prospective cohort study. BJOG : an international journal of obstetrics and gynaecology 2014; 121(13): 1604-10.*

368. *Meltzer-Brody S, Boschloo L, Jones I, Sullivan PF, Penninx BW. The EPDS-Lifetime: assessment of lifetime prevalence and risk factors for perinatal depression in a large cohort of depressed women. Archives of women's mental health 2013; 16(6): 465-73.*

369. *Dmitrovic BK, Dugalic MG, Balkoski GN, Dmitrovic A, Soldatovic I. Frequency of perinatal depression in Serbia and associated risk factors. The International journal of social psychiatry 2014; 60(6): 528-32.*

370. *Kerstis B, Engstrom G, Sundquist K, Widarsson M, Rosenblad A. The association between perceived relationship discord at childbirth and parental postpartum depressive symptoms: a comparison of mothers and fathers in Sweden. Upsala journal of medical sciences 2012; 117(4): 430-8.*

371. *Grote V, Vik T, von Kries R, et al. Maternal postnatal depression and child growth: a European cohort study. BMC pediatrics 2010; 10: 14.*

372. *McMahon CA, Boivin J, Gibson FL, Hammarberg K, Wynter K, Fisher JR. Older maternal age and major depressive episodes in the first two years after birth: findings from the Parental Age and Transition to Parenthood Australia (PATPA) study. Journal of affective disorders 2015; 175: 454-62.*

373. *Woolhouse H, Gartland D, Perlen S, Donath S, Brown SJ. Physical health after childbirth and maternal depression in the first 12 months post partum: results of an Australian nulliparous pregnancy cohort study. Midwifery 2014; 30(3): 378-84.*

374. *Petrosyan D, Armenian HK, Arzoumanian K. Interaction of maternal age and mode of delivery in the development of postpartum depression in Yerevan, Armenia. Journal of affective disorders 2011; 135(1-3): 77-81.*

375. *Wu M, Li X, Feng B, Wu H, Qiu C, Zhang W. Poor sleep quality of third-trimester pregnancy is a risk factor for postpartum depression. Medical science monitor : international medical journal of experimental and clinical research 2014; 20: 2740-5.*

376. *Ngai FW, Ngu SF. Predictors of maternal and paternal depressive symptoms at postpartum. Journal of psychosomatic research 2015; 78(2): 156-61.*

377. Lau Y, Wong DF, Chan KS. The utility of screening for perinatal depression in the second trimester among Chinese: a three-wave prospective longitudinal study. Archives of women's mental health 2010; 13(2): 153-64.

378. Taherifard P, Delpisheh A, Shirali R, Afkhamzadeh A, Veisani Y. Socioeconomic, psychiatric and materiality determinants and risk of postpartum depression in border city of ilam, Western iran. Depression research and treatment 2013; 2013: 653471.

379. Glasser S, Tanous M, Shihab S, Goldman N, Ziv A, Kaplan G. Perinatal depressive symptoms among Arab women in northern Israel. Maternal and child health journal 2012; 16(6): 1197-205.

380. Pollock JI, Manaseki-Holland S, Patel V. Depression in Mongolian women over the first 2 months after childbirth: prevalence and risk factors. Journal of affective disorders 2009; 116(1-2): 126-33.

381. Husain N, Parveen A, Husain M, et al. Prevalence and psychosocial correlates of perinatal depression: a cohort study from urban Pakistan. Archives of women's mental health 2011; 14(5): 395-403.

382. Cankorur VS, Abas M, Berksun O, Stewart R. Social support and the incidence and persistence of depression between antenatal and postnatal examinations in Turkey: a cohort study. BMJ open 2015; 5(4): e006456.

383. Annagur A, Annagur BB, Sahin A, Ors R, Kara F. Is maternal depressive symptomatology effective on success of exclusive breastfeeding during postpartum 6 weeks? Breastfeeding medicine : the official journal of the Academy of Breastfeeding Medicine 2013; 8(1): 53-7.

384. Pocan AG, Aki OE, Parlakgumus AH, Gereklioglu C, Dolgun AB. The incidence of and risk factors for postpartum depression at an urban maternity clinic in Turkey. International journal of psychiatry in medicine 2013; 46(2): 179-94.

385. Akyuz A, Seven M, Devran A, Demiralp M. Infertility history: is it a risk factor for postpartum depression in Turkish women? The Journal of perinatal & neonatal nursing 2010; 24(2): 137-45.

386. Beydoun HA, Al-Sahab B, Beydoun MA, Tamim H. Intimate partner violence as a risk factor for postpartum depression among Canadian women in the Maternity Experience Survey. Annals of epidemiology 2010; 20(8): 575-83.

387. Salm Ward T, Kanu FA, Robb SW. Prevalence of stressful life events during pregnancy and its association with postpartum depressive symptoms. Archives of women's mental health 2017; 20(1): 161-71.

388. Daoud N, O'Brien K, O'Campo P, et al. Postpartum depression prevalence and risk factors among Indigenous, non-Indigenous and immigrant women in Canada. Canadian journal of public health = Revue canadienne de sante publique 2019; 110(4): 440-52.

389. Miyake Y, Tanaka K, Arakawa M. Associations of job type, income, and education with postpartum depressive symptoms: The Kyushu Okinawa Maternal and Child Health Study. Psychiatry research 2020; 291: 113224.

390. Miller RL, Pallant JF, Negri LM. Anxiety and stress in the postpartum: is there more to postnatal distress than depression? BMC psychiatry 2006; 6: 12.

391. Sutter-Dallay AL, Giaconne-Marcesche V, Glatigny-Dallay E, Verdoux H. Women with anxiety disorders during pregnancy are at increased risk of intense postnatal depressive symptoms: a prospective survey of the MATQUID cohort. European psychiatry : the journal of the Association of European Psychiatrists 2004; 19(8): 459-63.

392. Edvinsson A, Skalkidou A, Hellgren C, et al. Different patterns of attentional bias in antenatal and postpartum depression. Brain and behavior 2017; 7(11): e00844.

393. Aydin N, Inandi T, Yigit A, Hodoglugil NN. Validation of the Turkish version of the Edinburgh Postnatal Depression Scale among women within their first postpartum year. Social psychiatry and psychiatric epidemiology 2004; 39(6): 483-6.

394. Ege E, Timur S, Zincir H, Geckil E, Sunar-Reeder B. Social support and symptoms of postpartum depression among new mothers in Eastern Turkey. The journal of obstetrics and gynaecology research 2008; 34(4): 585-93.

395. Ekuklu G, Tokuc B, Eskiocak M, Berberoglu U, Saltik A. Prevalence of postpartum depression in Edirne, Turkey, and related factors. The Journal of reproductive medicine 2004; 49(11): 908-14.

396. Mishra K, Mohapatra I, Rout RN. An epidemiological study on depression among women during postpartum period in an urban slum of Bhubaneswar. Journal of family medicine and primary care 2020; 9(9): 4736-40.

397.Kaya L, Cigdem Z. The relationship between mode of delivery and postpartum depression. Journal of education and health promotion 2019; 8: 5.

398. Liu S, Yan Y, Gao X, et al. Risk factors for postpartum depression among Chinese women: path model analysis. BMC pregnancy and childbirth 2017; 17(1): 133.

399. Sylven SM, Thomopoulos TP, Kollia N, Jonsson M, Skalkidou A. Correlates of postpartum depression in first time mothers without previous psychiatric contact. European psychiatry : the journal of the Association of European Psychiatrists 2017; 40: 4-12.

400. Cirik DA, Yerebasmaz N, Kotan VO, et al. The impact of prenatal psychologic and obstetric parameters on postpartum depression in late-term pregnancies: A preliminary study. Taiwanese journal of obstetrics & gynecology 2016; 55(3): 374-8.

401. Radesky JS, Zuckerman B, Silverstein M, et al. Inconsolable infant crying and maternal postpartum depressive symptoms. Pediatrics 2013; 131(6): e1857-64.

402. Nicklas JM, Miller LJ, Zera CA, Davis RB, Levkoff SE, Seely EW. Factors associated with depressive symptoms in the early postpartum period among women with recent gestational diabetes mellitus. Maternal and child health journal 2013; 17(9): 1665-72.

403. Eisenach JC, Pan PH, Smiley R, Lavand'homme P, Landau R, Houle TT. Severity of acute pain after childbirth, but not type of delivery, predicts persistent pain and postpartum depression. Pain 2008; 140(1): 87-94.

404. Gulseren L, Erol A, Gulseren S, Kuey L, Kilic B, Ergor G. From antepartum to postpartum: a prospective study on the prevalence of peripartum depression in a semiurban Turkish community. The Journal of reproductive medicine 2006; 51(12): 955-60.

405. Aris-Meijer J, Bockting C, Stolk R, et al. What If Pregnancy Is Not Seventh Heaven? The Influence of Specific Life Events during Pregnancy and Delivery on the Transition of Antenatal into Postpartum Anxiety and Depression. International journal of environmental research and public health 2019; 16(16).

406. Edge D, Baker D, Rogers A. Perinatal depression among black Caribbean women. Health & social care in the community 2004; 12(5): 430-8.

407. Morrell CJ, Slade P, Warner R, et al. Clinical effectiveness of health visitor training in psychologically informed approaches for depression in postnatal women: pragmatic cluster randomised trial in primary care. Bmj 2009; 338: a3045.

408. Nakano M, Sourander A, Luntamo T, Chudal R, Skokauskas N, Kaneko H. Early risk factors for postpartum depression: A longitudinal Japanese population-based study. Journal of affective disorders 2020; 269: 148-53.

409. Oladeji BD, Bello T, Kola L, Araya R, Zelkowitz P, Gureje O. Exploring Differences Between Adolescents and Adults With Perinatal Depression-Data From the Expanding Care for Perinatal Women With Depression Trial in Nigeria. Frontiers in psychiatry 2019; 10: 761.

410. Certain HE, Mueller M, Jagodzinski T, Fleming M. Domestic abuse during the previous year in a sample of postpartum women. Journal of obstetric, gynecologic, and neonatal nursing : JOGNN 2008; 37(1): 35-41.

411. van der Zee-van den Berg AI, Boere-Boonekamp MM, Groothuis-Oudshoorn CGM, MJ IJ, Haasnoot-Smallegange RME, Reijneveld SA. Post-Up Study: Postpartum Depression Screening in Well-Child Care and Maternal Outcomes. Pediatrics 2017; 140(4).

412. Mishina H, Hayashino Y, Fukuhara S. Test performance of two-question screening for postpartum depressive symptoms. Pediatrics international : official journal of the Japan Pediatric Society 2009; 51(1): 48-53.

413. Dagher RK, McGovern PM, Alexander BH, Dowd BE, Ukestad LK, McCaffrey DJ. The psychosocial work environment and maternal postpartum depression. International journal of behavioral medicine 2009; 16(4): 339-46.

414. Ersek JL, Brunner Huber LR. Physical activity prior to and during pregnancy and risk of postpartum depressive symptoms. Journal of obstetric, gynecologic, and neonatal nursing : JOGNN 2009; 38(5): 556-66.

415. Gaffney KF, Kitsantas P, Brito A, Swamidoss CS. Postpartum depression, infant feeding practices, and infant weight gain at six months of age. Journal of pediatric health care : official publication of National Association of Pediatric Nurse Associates & Practitioners 2014; 28(1): 43-50.

416. Glynn LM, Sandman CA. Evaluation of the association between placental corticotrophin-releasing hormone and postpartum depressive symptoms. Psychosomatic medicine 2014; 76(5): 355-62.

417. Howell EA, Balbierz A, Wang J, Parides M, Zlotnick C, Leventhal H. Reducing postpartum depressive symptoms among black and Latina mothers: a randomized controlled trial. Obstetrics and gynecology 2012; 119(5): 942-9.

418. Faisal-Cury A, Menezes PR, Quayle J, Matijasevich A. Unplanned pregnancy and risk of maternal depression: secondary data analysis from a prospective pregnancy cohort. Psychology, health & medicine 2017; 22(1): 65-74.

419. Knights JE, Salvatore ML, Simpkins G, Hunter K, Khandelwal M. In search of best practice for postpartum depression screening: is once enough? European journal of obstetrics, gynecology, and reproductive biology 2016; 206: 99-104.

420. Velloza J, Njoroge J, Ngure K, et al. Cognitive testing of the PHQ-9 for depression screening among pregnant and postpartum women in Kenya. BMC psychiatry 2020; 20(1): 31.

421. Avilla JC, Giugliani C, Bizon A, Martins ACM, Senna AFK, Giugliani ERJ. Association between maternal satisfaction with breastfeeding and postpartum depression symptoms. PloS one 2020; 15(11): e0242333.

422. Al Rawahi A, Al Kiyumi MH, Al Kimyani R, et al. The Effect of Antepartum Depression on the Outcomes of Pregnancy and Development of Postpartum Depression: A prospective cohort study of Omani women. Sultan Qaboos University medical journal 2020; 20(2): e179-e86.

423. Mott SL, Schiller CE, Richards JG, O'Hara MW, Stuart S. Depression and anxiety among postpartum and adoptive mothers. Archives of women's mental health 2011; 14(4): 335-43.

424. Silverman ME, Loudon H. Antenatal reports of pre-pregnancy abuse is associated with symptoms of depression in the postpartum period. Archives of women's mental health 2010; 13(5): 411-5.

425. Watkins S, Meltzer-Brody S, Zolnoun D, Stuebe A. Early breastfeeding experiences and postpartum depression. Obstetrics and gynecology 2011; 118(2 Pt 1): 214-21.

426. Sun L. The association between postpartum depression and early childhood caries. Acta odontologica Scandinavica 2020; 78(5): 352-7.

427. Riazanova OV, Alexandrovich YS, Ioscovich AM. The relationship between labor pain management, cortisol level and risk of postpartum depression development: a prospective nonrandomized observational monocentric trial. Romanian journal of anaesthesia and intensive care 2018; 25(2): 123-30.

428. Lupattelli A, Twigg MJ, Zagorodnikova K, et al. Self-reported perinatal depressive symptoms and postnatal symptom severity after treatment with antidepressants in pregnancy: a cross-sectional study across 12 European countries using the Edinburgh Postnatal Depression Scale. Clinical epidemiology 2018; 10: 655-69.

429. Wesselhoeft R, Madsen FK, Lichtenstein MB, et al. Postnatal depressive symptoms display marked similarities across continents. Journal of affective disorders 2020; 261: 58-66.

430. Tho Nhi T, Hanh NTT, Hinh ND, et al. Intimate Partner Violence among Pregnant Women and Postpartum Depression in Vietnam: A Longitudinal Study. BioMed research international 2019; 2019: 4717485.

431. Do TKL, Nguyen TTH, Pham TTH. Postpartum Depression and Risk Factors among Vietnamese Women. BioMed research international 2018; 2018: 4028913.

432. Lee LC, Hung CH. Predictors of post-partum stress in Vietnamese immigrant women in Taiwan. Japan journal of nursing science : JJNS 2016; 13(1): 38-45.

433. Ing H, Fellmeth G, White J, Stein A, Simpson JA, McGready R. Validation of the Edinburgh Postnatal Depression Scale (EPDS) on the Thai-Myanmar border. Tropical doctor 2017; 47(4): 339-47.

434. Roomruangwong C, Withayavanitchai S, Maes M. Antenatal and postnatal risk factors of postpartum depression symptoms in Thai women: A case-control study. Sexual & reproductive healthcare : official journal of the Swedish Association of Midwives 2016; 10: 25-31.

435. Panyayong B. Postpartum depression among Thai women: a national survey. Journal of the Medical Association of Thailand = Chotmaihet thangphaet 2013; 96(7): 761-7.

436. Tomlinson M, Chaudhery D, Ahmadzai H, et al. Identifying and treating maternal mental health difficulties in Afghanistan: A feasibility study. International journal of mental health systems 2020; 14: 75.

437. Takacs L, Smolik F, Putnam S. Assessing longitudinal pathways between maternal depressive symptoms, parenting self-esteem and infant temperament. PloS one 2019; 14(8): e0220633.

438. Coo S, Garcia MI, Mira A, Valdes V. The Role of Perinatal Anxiety and Depression in Breastfeeding Practices. Breastfeeding medicine : the official journal of the Academy of Breastfeeding Medicine 2020; 15(8): 495-500.

439. Amemiya A, Fujiwara T. Association between maternal intimate partner violence victimization during pregnancy and maternal abusive behavior towards infants at 4 months of age in Japan. Child abuse & neglect 2016; 55: 32-9.

440. Kabir ZN, Nasreen HE, Edhborg M. Intimate partner violence and its association with maternal depressive symptoms 6-8 months after childbirth in rural Bangladesh. Global health action 2014; 7: 24725.

441. Miura A, Fujiwara T. Intimate Partner Violence during Pregnancy and Postpartum Depression in Japan: A Cross-sectional Study. Frontiers in public health 2017; 5: 81.

442. *Tho Tran N, Nguyen HTT, Nguyen HD, et al. Emotional violence exerted by intimate partners and postnatal depressive symptoms among women in Vietnam: A prospective cohort study. PloS one 2018; 13(11): e0207108.*

443. *Silove D, Rees S, Tay AK, et al. Pathways to perinatal depressive symptoms after mass conflict in Timor-Leste: a modelling analysis using cross-sectional data. The lancet Psychiatry 2015; 2(2): 161-7.*

444. *Faisal-Cury A, Menezes PR, d'Oliveira AF, Schraiber LB, Lopes CS. Temporal relationship between intimate partner violence and postpartum depression in a sample of low income women. Maternal and child health journal 2013; 17(7): 1297-303.*

445. *Lobato G, Moraes CL, Dias AS, Reichenheim ME. Alcohol misuse among partners: a potential effect modifier in the relationship between physical intimate partner violence and postpartum depression. Social psychiatry and psychiatric epidemiology 2012; 47(3): 427-38.*

446. *Sunnqvist C, Sjostrom K, Finnbogadottir H. Depressive symptoms during pregnancy and postpartum in women and use of antidepressant treatment - a longitudinal cohort study. International journal of women's health 2019; 11: 109-17.*

447. *Wei G, Greaver LB, Marson SM, Herndon CH, Rogers J, Robeson Healthcare C. Postpartum depression: racial differences and ethnic disparities in a tri-racial and bi-ethnic population. Maternal and child health journal 2008; 12(6): 699-707.*

448. *Silva CS, Lima MC, Sequeira-de-Andrade LAS, et al. Association between postpartum depression and the practice of exclusive breastfeeding in the first three months of life. Jornal de pediatria 2017; 93(4): 356-64.*

449. *Sha T, Gao X, Chen C, et al. A prospective study of maternal postnatal depressive symptoms with infant-feeding practices in a Chinese birth cohort. BMC pregnancy and childbirth 2019; 19(1): 388.*

450. *Gregory EF, Butz AM, Ghazarian SR, Gross SM, Johnson SB. Are unmet breastfeeding expectations associated with maternal depressive symptoms? Academic pediatrics 2015; 15(3): 319-25.*

451. *Woolhouse H, James J, Gartland D, McDonald E, Brown SJ. Maternal depressive symptoms at three months postpartum and breastfeeding rates at six months postpartum: Implications for primary care in a prospective cohort study of primiparous women in Australia. Women and birth : journal of the Australian College of Midwives 2016; 29(4): 381-7.*

452. *Woolhouse H, Gartland D, Mensah F, Brown SJ. Maternal depression from early pregnancy to 4 years postpartum in a prospective pregnancy cohort study: implications for primary health care. BJOG : an international journal of obstetrics and gynaecology 2015; 122(3): 312-21.*

453. *Woolhouse H, Gartland D, Hegarty K, Donath S, Brown SJ. Depressive symptoms and intimate partner violence in the 12 months after childbirth: a prospective pregnancy cohort study. BJOG : an international journal of obstetrics and gynaecology 2012; 119(3): 315-23.*

454. *Nakamura Y, Okada T, Morikawa M, et al. Perinatal depression and anxiety of primipara is higher than that of multipara in Japanese women. Scientific reports 2020; 10(1): 17060.*

455. Iwata H, Mori E, Sakajo A, Aoki K, Maehara K, Tamakoshi K. Prevalence of postpartum depressive symptoms during the first 6 months postpartum: Association with maternal age and parity. Journal of affective disorders 2016; 203: 227-32.

456. Takehara K, Tachibana Y, Yoshida K, Mori R, Kakee N, Kubo T. Prevalence trends of pre- and postnatal depression in Japanese women: A population-based longitudinal study. Journal of affective disorders 2018; 225: 389-94.

457. Iranpour S, Kheirabadi GR, Esmaillzadeh A, Heidari-Beni M, Maracy MR. Association between sleep quality and postpartum depression. Journal of research in medical sciences : the official journal of Isfahan University of Medical Sciences 2016; 21: 110.

458. Emerson BL, Bradley ER, Riera A, Mayes L, Bechtel K. Postpartum depression screening in the pediatric emergency department. Pediatric emergency care 2014; 30(11): 788-92.

459. Birmingham MC, Chou KJ, Crain EF. Screening for postpartum depression in a pediatric emergency department. Pediatric emergency care 2011; 27(9): 795-800.

460. Gong W, Jin X, Cheng KK, Caine ED, Lehman R, Xu DR. Chinese Women's Acceptance and Uptake of Referral after Screening for Perinatal Depression. International journal of environmental research and public health 2020; 17(22).

461. Kothari CL, Liepman MR, Shama Tareen R, et al. Intimate Partner Violence Associated with Postpartum Depression, Regardless of Socioeconomic Status. Maternal and child health journal 2016; 20(6): 1237-46.

462. Li Q, Yang S, Xie M, et al. Impact of some social and clinical factors on the development of postpartum depression in Chinese women. BMC pregnancy and childbirth 2020; 20(1): 226.

463. Liu Y, Guo N, Li T, Zhuang W, Jiang H. Prevalence and Associated Factors of Postpartum Anxiety and Depression Symptoms Among Women in Shanghai, China. Journal of affective disorders 2020; 274: 848-56.

464. Cao L, Liu Y, Liang X, et al. Association between dietary patterns during the third trimester and the risk of postpartum depression in China. Journal of affective disorders 2020; 264: 370-5.

465. Chen L, Ding L, Qi M, Jiang C, Mao XM, Cai WZ. Incidence of and social-demographic and obstetric factors associated with postpartum depression: differences among ethnic Han and Kazak women of Northwestern China. PeerJ 2018; 6: e4335.

466. Liang P, Wang Y, Shi S, Liu Y, Xiong R. Prevalence and factors associated with postpartum depression during the COVID-19 pandemic among women in Guangzhou, China: a cross-sectional study. BMC psychiatry 2020; 20(1): 557.

467. Ding G, Niu L, Vinturache A, et al. "Doing the month" and postpartum depression among Chinese women: A Shanghai prospective cohort study. Women and birth : journal of the Australian College of Midwives 2020; 33(2): e151-e8.

468. Lu L, Duan Z, Wang Y, et al. Mental health outcomes among Chinese prenatal and postpartum women after the implementation of universal two-child policy. Journal of affective disorders 2020; 264: 187-92.

469. Jiang W, Mo M, Li M, et al. The relationship of dietary diversity score with depression and anxiety among prenatal and post-partum women. The journal of obstetrics and gynaecology research 2018; 44(10): 1929-36.

470. Shi X, Ying Y, Yu Z, et al. Risk factors for postpartum depression in Chinese women: A cross-sectional study at 6 weeks postpartum. Journal of psychosomatic research 2021; 140: 110295.

471. Zheng X, Morrell J, Watts K. Changes in maternal self-efficacy, postnatal depression symptoms and social support among Chinese primiparous women during the initial postpartum period: A longitudinal study. Midwifery 2018; 62: 151-60.

472. Wan EY, Moyer CA, Harlow SD, Fan Z, Jie Y, Yang H. Postpartum depression and traditional postpartum care in China: role of zuoyuezi. International journal of gynaecology and obstetrics: the official organ of the International Federation of Gynaecology and Obstetrics 2009; 104(3): 209-13.

473. Luo SC, Duan KM, Fang C, et al. Correlations Between SIRT Genetic Polymorphisms and Postpartum Depressive Symptoms in Chinese Parturients Who Had Undergone Cesarean Section. Neuropsychiatric disease and treatment 2020; 16: 3225-38.

474. Quan C, Wang S, Duan K, et al. The role of kynurenine pathway and kynurenic aminotransferase alleles in postpartum depression following cesarean section in Chinese women. Brain and behavior 2020; 10(4): e01566.

475. Fu CW, Liu JT, Tu WJ, Yang JQ, Cao Y. Association between serum 25-hydroxyvitamin D levels measured 24 hours after delivery and postpartum depression. BJOG : an international journal of obstetrics and gynaecology 2015; 122(12): 1688-94.

476. Lin PY, Chiu TH, Ho M, Pei-Chen Chang J, Hui-Chih Chang C, Su KP. Major depressive episodes during pregnancy and after childbirth: A prospective longitudinal study in Taiwan. Journal of the Formosan Medical Association = Taiwan yi zhi 2019; 118(11): 1551-9.

477. Liu H, Zhang Y, Gao Y, Zhang Z. Elevated levels of Hs-CRP and IL-6 after delivery are associated with depression during the 6 months post partum. Psychiatry research 2016; 243: 43-8.

478. Deng CM, Ding T, Li S, et al. Neuraxial labor analgesia is associated with a reduced risk of postpartum depression: A multicenter prospective cohort study with propensity score matching. Journal of affective disorders 2021; 281: 342-50.

479. Gao X, Wang J, Yao H, Cai Y, Cheng R. Serum BDNF concentration after delivery is associated with development of postpartum depression: A 3-month follow up study. Journal of affective disorders 2016; 200: 25-30.

480. Liu ZH, He ST, Deng CM, et al. Neuraxial labour analgesia is associated with a reduced risk of maternal depression at 2 years after childbirth: A multicentre, prospective, longitudinal study. European journal of anaesthesiology 2019; 36(10): 745-54.

481. Sun J, Xiao Y, Zou L, et al. Epidural Labor Analgesia Is Associated with a Decreased Risk of the Edinburgh Postnatal Depression Scale in Trial of Labor after Cesarean: A Multicenter, Prospective Cohort Study. BioMed research international 2020; 2020: 2408063.

482. Ding T, Wang DX, Qu Y, Chen Q, Zhu SN. Epidural labor analgesia is associated with a decreased risk of postpartum depression: a prospective cohort study. Anesthesia and analgesia 2014; 119(2): 383-92.

483. Pham D, Cormick G, Amyx MM, et al. Factors associated with postpartum depression in women from low socioeconomic level in Argentina: A hierarchical model approach. Journal of affective disorders 2018; 227: 731-8.

484. Fraga A, Theme-Filha MM. Pregestational overweight and obesity and symptoms of postpartum depression: Data from the Birth in Brazil Study. Journal of affective disorders 2020; 277: 463-9.

485. Roomruangwong C, Kanchanatawan B, Sirivichayakul S, Maes M. Antenatal depression and hematocrit levels as predictors of postpartum depression and anxiety symptoms. Psychiatry research 2016; 238: 211-7.

486. Faisal-Cury A. Postpartum Depression and Early Predictors of Lower Maternal Confidence at 12 to 15 Months after Delivery. The Psychiatric quarterly 2020.

487. Silveira MF, Mesenburg MA, Bertoldi AD, et al. The association between disrespect and abuse of women during childbirth and postpartum depression: Findings from the 2015 Pelotas birth cohort study. Journal of affective disorders 2019; 256: 441-7.

488. Costa VPP, Correa MB, Goettems ML, Pinheiro RT, Demarco FF. Maternal depression and anxiety associated with dental fear in children: a cohort of adolescent mothers in Southern Brazil. Brazilian oral research 2017; 31: e85.

489. Faisal-Cury A, Bertazzi Levy R, Kontos A, Tabb K, Matijasevich A. Postpartum bonding at the beginning of the second year of child's life: the role of postpartum depression and early bonding impairment. Journal of psychosomatic obstetrics and gynaecology 2020; 41(3): 224-30.

490. Brito CN, Alves SV, Ludermir AB, Araujo TV. Postpartum depression among women with unintended pregnancy. Revista de saude publica 2015; 49: 33.

491. Araujo IS, Aquino KS, Fagundes LKA, Santos VC. Postpartum Depression: Epidemiological Clinical Profile of Patients Attended In a Reference Public Maternity in Salvador-BA. Revista brasileira de ginecologia e obstetricia : revista da Federacao Brasileira das Sociedades de Ginecologia e Obstetricia 2019; 41(3): 155-63.

492. Callo-Quinte G, Del-Ponte B, Ruivo ACO, et al. Maternal depression symptoms and use of child health-care services at The Pelotas 2004 Birth Cohort. Journal of affective disorders 2019; 253: 303-7.

493. Zaconeta AM, Queiroz IF, Amato AA, Motta LD, Casulari LA. Depression with postpartum onset: a prospective cohort study in women undergoing elective cesarean section in Brasilia, Brazil. Revista brasileira de ginecologia e obstetricia : revista da Federacao Brasileira das Sociedades de Ginecologia e Obstetricia 2013; 35(3): 130-5.

494. Tannous L, Gigante LP, Fuchs SC, Busnello ED. Postnatal depression in Southern Brazil: prevalence and its demographic and socioeconomic determinants. BMC psychiatry 2008; 8: 1.

495. Faisal-Cury A, Tabb K, Matijasevich A. Partner relationship quality predicts later postpartum depression independently of the chronicity of depressive symptoms. Revista brasileira de psiquiatria 2020.

496. Pinheiro RT, Magalhaes PV, Horta BL, Pinheiro KA, da Silva RA, Pinto RH. Is paternal postpartum depression associated with maternal postpartum depression? Population-based study in Brazil. Acta psychiatrica Scandinavica 2006; 113(3): 230-2.

497. Valdes M, Hanchey A, Munoz MP, Baumert B, Iglesias V. Low-level arsenic exposure during pregnancy and its association with postpartum depression: A cohort study of women from Arica, Chile. Revue d'epidemiologie et de sante publique 2017; 65(6): 427-35.

498. Bauman BL, Ko JY, Cox S, et al. Vital Signs: Postpartum Depressive Symptoms and Provider Discussions About Perinatal Depression - United States, 2018. MMWR Morbidity and mortality weekly report 2020; 69(19): 575-81.

499. Wissart J, Parshad O, Kulkarni S. Prevalence of pre- and postpartum depression in Jamaican women. BMC pregnancy and childbirth 2005; 5: 15.

500. Youn H, Lee S, Han SW, et al. Obstetric risk factors for depression during the postpartum period in South Korea: a nationwide study. Journal of psychosomatic research 2017; 102: 15-20.

501. Choi SK, Park YG, Park IY, Ko HS, Shin JC. Impact of antenatal depression on perinatal outcomes and postpartum depression in Korean women. Journal of research in medical sciences : the official journal of Isfahan University of Medical Sciences 2014; 19(9): 807-12.

502. Kim K, Hong JP, Cho MJ, et al. Loss of sexual interest and premenstrual mood change in women with postpartum versus non-postpartum depression: A nationwide community sample of Korean adults. Journal of affective disorders 2016; 191: 222-9.

503. Suhitharan T, Pham TP, Chen H, et al. Investigating analgesic and psychological factors associated with risk of postpartum depression development: a case-control study. Neuropsychiatric disease and treatment 2016; 12: 1333-9.

504. Alves S, Fonseca A, Canavarro MC, Pereira M. Preliminary Psychometric Testing of the Postpartum Depression Predictors Inventory-Revised (PDPI-R) in Portuguese Women. Maternal and child health journal 2018; 22(4): 571-8.

505. Gutierrez-Zotes A, Diaz-Pena R, Costas J, et al. Interaction between the functional SNP rs2070951 in NR3C2 gene and high levels of plasma corticotropin-releasing hormone associates to postpartum depression. Archives of women's mental health 2020; 23(3): 413-20.

506. *Ikeda M, Kamibeppu K. Measuring the risk factors for postpartum depression: development of the Japanese version of the Postpartum Depression Predictors Inventory-Revised (PDPI-R-J). BMC pregnancy and childbirth 2013; 13: 112.*

507. *Fellmeth G, Plugge E, Fazel M, et al. Prevalence and determinants of perinatal depression among labour migrant and refugee women on the Thai-Myanmar border: a cohort study. BMC psychiatry 2020; 20(1): 168.*

508. *Hossain SJ, Roy BR, Hossain AT, et al. Prevalence of Maternal Postpartum Depression, Health-Seeking Behavior and Out of Pocket Payment for Physical Illness and Cost Coping Mechanism of the Poor Families in Bangladesh: A Rural Community-Based Study. International journal of environmental research and public health 2020; 17(13).*

509. *Nasreen HE, Kabir ZN, Forsell Y, Edhborg M. Impact of maternal depressive symptoms and infant temperament on early infant growth and motor development: results from a population based study in Bangladesh. Journal of affective disorders 2013; 146(2): 254-61.*

510. *Salehi-Pourmehr H, Mohammad-Alizadeh S, Jafarilar-Agdam N, Rafiee S, Farshbaf-Khalili A. The association between pre-pregnancy obesity and screening results of depression for all trimesters of pregnancy, postpartum and 1 year after birth: a cohort study. Journal of perinatal medicine 2018; 46(1): 87-95.*

511. *Dayan F, Javadifar N, Tadayon M, Malehi AS, Komeili Sani H. The Relationship between Gestational Weight Gain and Postpartum Depression in Normal and Overweight Pregnant Women. Journal of pregnancy 2018; 2018: 9315320.*

512. *Ezzeddin N, Jahanihashemi H, Zavoshy R, Noroozi M. The Prevalence of Postpartum Depression and Its Association with Food Insecurity among Mothers Referring to Community Health Centers. Iranian journal of psychiatry 2018; 13(4): 280-7.*

513. *Ahmed A, Bowen A, Feng CX. Maternal depression in Syrian refugee women recently moved to Canada: a preliminary study. BMC pregnancy and childbirth 2017; 17(1): 240.*

514. *Safadi RR, Abushaikha LA, Ahmad MM. Demographic, maternal, and infant health correlates of post-partum depression in Jordan. Nursing & health sciences 2016; 18(3): 306-13.*

515. *Yehia DB, Callister LC, Hamdan-Mansour A. Prevalence and predictors of postpartum depression among Arabic Muslim Jordanian women serving in the military. The Journal of perinatal & neonatal nursing 2013; 27(1): 25-33; quiz 4-5.*

516. *Shwartz N, O'Rourke N, Daoud N. Pathways Linking Intimate Partner Violence and Postpartum Depression Among Jewish and Arab Women in Israel. Journal of interpersonal violence 2020: 886260520908022.*

517. *Molmen Lichter M, Peled Y, Levy S, Wiznitzer A, Krissi H, Handelzalts JE. The associations between insecure attachment, rooming-in, and postpartum depression: A 2 months' longitudinal study. Infant mental health journal 2021; 42(1): 74-86.*

518. *Freedman SA, Reshef S, Weiniger CF. Post-traumatic stress disorder and postpartum depression and their reported association with recent labor and delivery: a questionnaire survey cohort. International journal of obstetric anesthesia 2020; 43: 18-24.*

519. *Goren G, Sarid O, Philippou P, Taylor A. Sense of Coherence Mediates the Links between Job Status Prior to Birth and Postpartum Depression: A Structured Equation Modeling Approach. International journal of environmental research and public health 2020; 17(17).*

520. *Orbach-Zinger S, Landau R, Harousch AB, et al. The Relationship Between Women's Intention to Request a Labor Epidural Analgesia, Actually Delivering With Labor Epidural Analgesia, and Postpartum Depression at 6 Weeks: A Prospective Observational Study. Anesthesia and analgesia 2018; 126(5): 1590-7.*

521. *Bina R. Seeking help for postpartum depression in the Israeli Jewish orthodox community: factors associated with use of professional and informal help. Women & health 2014; 54(5): 455-73.*

522. *Alzahrani AD. Risk Factors for Postnatal Depression among Primipara Mothers. The Spanish journal of psychology 2019; 22: E35.*

523. *Haight SC, Ko JY, Yogman MW, Farr SL. Postpartum Depressive Symptoms and Screening Opportunities at Health Care Encounters. Journal of women's health 2020.*

524. *Farr SL, Denk CE, Dahms EW, Dietz PM. Evaluating universal education and screening for postpartum depression using population-based data. Journal of women's health 2014; 23(8): 657-63.*

525. *Farr SL, Dietz PM, O'Hara MW, Burley K, Ko JY. Postpartum anxiety and comorbid depression in a population-based sample of women. Journal of women's health 2014; 23(2): 120-8.*

526. *Mark TE, Latulipe RJ, Anto-Ocrah M, Mlongoti G, Adler D, Lanning JW. Seasonality, Food Insecurity, and Clinical Depression in Post-Partum Women in a Rural Malawi Setting. Maternal and child health journal 2020.*

527. *Raisanen S, Lehto SM, Nielsen HS, Gissler M, Kramer MR, Heinonen S. Fear of childbirth predicts postpartum depression: a population-based analysis of 511 422 singleton births in Finland. BMJ open 2013; 3(11): e004047.*

528. *Demirchyan A, Petrosyan D, Armenian HK. Rate and predictors of postpartum depression in a 22-year follow-up of a cohort of earthquake survivors in Armenia. Archives of women's mental health 2014; 17(3): 229-37.*

529. *Eckerdal P, Kollia N, Lofblad J, et al. Delineating the Association between Heavy Postpartum Haemorrhage and Postpartum Depression. PloS one 2016; 11(1): e0144274.*

530. *Falah-Hassani K, Shiri R, Dennis CL. Prevalence and risk factors for comorbid postpartum depressive symptomatology and anxiety. Journal of affective disorders 2016; 198: 142-7.*

531. Kiviruusu O, Pietikainen JT, Kylliainen A, et al. Trajectories of mothers' and fathers' depressive symptoms from pregnancy to 24 months postpartum. Journal of affective disorders 2020; 260: 629-37.

532. Sarberg M, Bladh M, Svanborg E, Josefsson A. Postpartum depressive symptoms and its association to daytime sleepiness and restless legs during pregnancy. BMC pregnancy and childbirth 2016; 16(1): 137.

533. Muchanga SMJ, Eitoku M, Mbelambela EP, et al. Association between nausea and vomiting of pregnancy and postpartum depression: the Japan Environment and Children's Study. Journal of psychosomatic obstetrics and gynaecology 2020: 1-9.

534. Asif S, Mulic-Lutvica A, Axfors C, et al. Severe obstetric lacerations associated with postpartum depression among women with low resilience - a Swedish birth cohort study. BJOG : an international journal of obstetrics and gynaecology 2020; 127(11): 1382-90.

535. Rosander M, Berlin A, Forslund Frykedal K, Barimani M. Maternal depression symptoms during the first 21 months after giving birth. Scandinavian journal of public health 2020: 1403494820977969.

536. Holm-Larsen CE, Madsen FK, Rogathi JJ, et al. Postpartum depression and child growth in Tanzania: a cohort study. BJOG : an international journal of obstetrics and gynaecology 2019; 126(5): 590-8.

537. Maliszewska K, Bidzan M, Swiatkowska-Freund M, Preis K. Medical and psychosocial determinants of risk of postpartum depression: a cross-sectional study. Acta neuropsychiatrica 2017; 29(6): 347-55.

538. Maliszewska K, Swiatkowska-Freund M, Bidzan M, Krzysztof P. Screening for maternal postpartum depression and associationswith personality traits and social support. A Polish follow-upstudy 4 weeks and 3 months after delivery. Psychiatria polska 2017; 51(5): 889-98.

539. Gray PH, Edwards DM, O'Callaghan MJ, Cuskelly M, Gibbons K. Parenting stress in mothers of very preterm infants -- influence of development, temperament and maternal depression. Early human development 2013; 89(9): 625-9.

540. Shah S, Lonergan B. Frequency of postpartum depression and its association with breastfeeding: A cross-sectional survey at immunization clinics in Islamabad, Pakistan. JPMA The Journal of the Pakistan Medical Association 2017; 67(8): 1151-6.

541. Fellmeth G, Opondo C, Henderson J, et al. Identifying postnatal depression: Comparison of a self-reported depression item with Edinburgh Postnatal Depression Scale scores at three months postpartum. Journal of affective disorders 2019; 251: 8-14.

542. Cruise SM, Layte R, Stevenson M, O'Reilly D. Prevalence and factors associated with depression and depression-related healthcare access in mothers of 9-month-old infants in the Republic of Ireland. Epidemiology and psychiatric sciences 2018; 27(5): 468-78.

543. Wesseloo R, Kamperman AM, Bergink V, Pop VJM. Thyroid peroxidase antibodies during early gestation and the subsequent risk of first-onset postpartum depression: A prospective cohort study. Journal of affective disorders 2018; 225: 399-403.

544. Fritel X, Tsegan YE, Pierre F, Saurel-Cubizolles MJ, Group EM-CCS. Association of postpartum depressive symptoms and urinary incontinence. A cohort study. European journal of obstetrics, gynecology, and reproductive biology 2016; 198: 62-7.

545.Binda V, Figueroa-Leigh F, Olhaberry M. Antenatal and postnatal depressive symptoms: Association with quality of mother-infant interaction. Infant behavior & development 2019; 57: 101386.

546. Leite TH, Pereira APE, Leal MDC, da Silva AAM. Disrespect and abuse towards women during childbirth and postpartum depression: findings from Birth in Brazil Study. Journal of affective disorders 2020; 273: 391-401.

547. Ferrari B, Mesiano L, Benacchio L, Ciulli B, Donolato A, Riolo R. Prevalence and risk factors of postpartum depression and adjustment disorder during puerperium - a retrospective research. Journal of reproductive and infant psychology 2020: 1-13.

548. Epifanio MS, Genna V, De Luca C, Roccella M, La Grutta S. Paternal and Maternal Transition to Parenthood: The Risk of Postpartum Depression and Parenting Stress. Pediatric reports 2015; 7(2): 5872.

549. Vismara L, Rolle L, Agostini F, et al. Perinatal Parenting Stress, Anxiety, and Depression Outcomes in First-Time Mothers and Fathers: A 3- to 6-Months Postpartum Follow-Up Study. Frontiers in psychology 2016; 7: 938.

550. Clavenna A, Seletti E, Cartabia M, et al. Postnatal depression screening in a paediatric primary care setting in Italy. BMC psychiatry 2017; 17(1): 42.

551. Palumbo G, Mirabella F, Gigantesco A. Positive screening and risk factors for postpartum depression. European psychiatry : the journal of the Association of European Psychiatrists 2017; 42: 77-85.

552. Gremigni P, Mariani L, Marracino V, Tranquilli AL, Turi A. Partner support and postpartum depressive symptoms. Journal of psychosomatic obstetrics and gynaecology 2011; 32(3): 135-40.

553. Turner K, Piazzini A, Franza A, et al. Postpartum depression in women with epilepsy versus women without epilepsy. Epilepsy & behavior : E&B 2006; 9(2): 293-7.

554. Albacar G, Sans T, Martin-Santos R, et al. An association between plasma ferritin concentrations measured 48 h after delivery and postpartum depression. Journal of affective disorders 2011; 131(1-3): 136-42.

555. Albacar G, Sans T, Martin-Santos R, et al. Thyroid function 48h after delivery as a marker for subsequent postpartum depression. Psychoneuroendocrinology 2010; 35(5): 738-42.

556. Jin Y, Coad J, Pond R, Kim N, Brough L. Selenium intake and status of postpartum women and postnatal depression during the first year after childbirth in New Zealand - Mother and Infant Nutrition Investigation (MINI) study. Journal of trace elements in medicine and biology : organ of the Society for Minerals and Trace Elements 2020; 61: 126503.

557. Chan JE, Samaranayaka A, Paterson H. Seasonal and gestational variation in perinatal depression in a prospective cohort in New Zealand. The Australian & New Zealand journal of obstetrics & gynaecology 2019; 59(4): 514-22.

558. Gould JF, Anderson AJ, Yelland LN, et al. Association of cord blood vitamin D at delivery with postpartum depression in Australian women. The Australian & New Zealand journal of obstetrics & gynaecology 2015; 55(5): 446-52.

559. Pingo J, van den Heuvel LL, Vythylingum B, Seedat S. Probable postpartum hypomania and depression in a South African cohort. Archives of women's mental health 2017; 20(3): 427-37.

560. Madeghe BA, Kimani VN, Vander Stoep A, Nicodimos S, Kumar M. Postpartum depression and infant feeding practices in a low income urban settlement in Nairobi-Kenya. BMC research notes 2016; 9(1): 506.

561. Xie, R.-H., Liao, S., Xie, H., Guo, Y., Walker, M., & Wen, S. W. (2010). Infant sex, family support and postpartum depression in a Chinese cohort. Journal of Epidemiology & Community Health, 65(8), 722–726.

562. De Tychey, C., Spitz, E., Briançon, S., Lighezzolo, J., Girvan, F., Rosati, A., … Vincent, S. (2005).Pre- and postnatal depression and coping: a comparative approach. Journal of Affective Disorders, 85(3), 323–326.

563. Alasoom LI, Koura MR. Predictors of postpartum depression in the eastern province capital of saudi arabia. J Family Med Prim Care. 2014 Apr;3(2):146-50.

564. Shivalli S, Gururaj N. Postnatal depression among rural women in South India: do socio-demographic, obstetric and pregnancy outcome have a role to play? PLoS One. 2015 Apr 7;10(4):e0122079.

565. Lambrinoudaki I, Rizos D, Armeni E, Pliatsika P, Leonardou A, Sygelou A, Argeitis J, Spentzou G, Hasiakos D, Zervas I, Papadias C. Thyroid function and postpartum mood disturbances in Greek women. J Affect Disord. 2010 Mar;121(3):278-82.
